# The translocator protein ligands as mitochondrial functional modulators for the potential anti-Alzheimer agents

**DOI:** 10.1080/14756366.2021.1900158

**Published:** 2021-03-23

**Authors:** TaeHun Kim, Mohammad N. Morshed, Ashwini M. Londhe, Ji W. Lim, Ha E. Lee, Suengmok Cho, Sung J. Cho, Hayoung Hwang, Sang M. Lim, Jae Y. Lee, Jiyoun Lee, Ae N. Pae

**Affiliations:** a Convergence Research Center for Diagnosis, Treatment and Care System of Dementia, Korea Institute of Science and Technology, Seoul, Republic of Korea; b Division of Bio-Medical Science and Technology, KIST School, Korea University of Science and Technology, Seoul, Republic of Korea; c Center for Advanced Research in Sciences (CARS), University of Dhaka, Dhaka, Bangladesh; d KHU-KIST Department of Converging Science and Technology, Kyung Hee University, Seoul, Republic of Korea; e Department of Food Science and Technology, Pukyong National University, Pusan, Republic of Korea; f New Drug Development Center, Daegu-Gyeongbuk Medical Innovation Foundation (DGMIF), Daegu, Republic of Korea; g Department of Global Medical Science, Sungshin University, Seoul, Republic of Korea

**Keywords:** Alzheimer’s disease, function modulators, translocator protein ligand

## Abstract

Small molecule modulators of mitochondrial function have been attracted much attention in recent years due to their potential therapeutic applications for neurodegenerative diseases. The mitochondrial translocator protein (TSPO) is a promising target for such compounds, given its involvement in the formation of the mitochondrial permeability transition pore in response to mitochondrial stress. In this study, we performed a ligand-based pharmacophore design and virtual screening, and identified a potent hit compound, **7** (**VH34**) as a TSPO ligand. After validating its biological activity against amyloid-β (Aβ) induced mitochondrial dysfunction and in acute and transgenic Alzheimer’s disease (AD) model mice, we developed a library of analogs, and we found two most active compounds, **31** and **44**, which restored the mitochondrial membrane potential, ATP production, and cell viability under Aβ-induced mitochondrial toxicity. These compounds recovered learning and memory function in acute AD model mice with improved pharmacokinetic properties.

## 1. Introduction

Alzheimer’s disease (AD) is a late-onset, progressive, age-dependent neurodegenerative disorder, characterised clinically by the impairment of cognitive functions and changes in behaviour and personality^1^. Among several hypothesis regarding the AD pathogenesis, mitochondrial dysfunction has been reported as an early event observed in AD^2^. Specifically, recent studies suggested that amyloid-β (Aβ) has deleterious effects on mitochondrial function and may contribute to respiratory chain impairment, neuronal apoptosis, and generation of reactive oxygen species (ROS) in AD^3^^,^^4^. While the mechanisms underlying Aβ-induced mitochondrial stress is still under debate, emerging evidences indicate that the maintenance of mitochondrial function may provide a potential treatment option for AD^5^^,^^6^.

As a therapeutic strategy against AD, several mitochondrial proteins have been highlighted as promising drug targets^7^^,^^8^. In particular, the 18 k*Da* translocator protein (TSPO) is an evolutionally conserved protein located in the outer mitochondrial membrane (OMM), and is known to play crucial roles in steroidogenesis, apoptosis, immune response, and Ca^2+^ homeostasis^9^^,^^10^. TSPO is also involved in mitochondrial functions such as ATP production, ROS generation, and mitochondrial polarisation (ΔΨ_m_)^11^. More importantly, several TSPO ligands showed neuroprotective effect by decreasing microglial activation in a rat model of neurodegeneration, suggesting that TSPO may serve as a potential therapeutic target^12^. In particular, it has been reported that TSPO ligands may act on the formation of the mitochondrial permeability transition pore (mPTP), which causes mitochondrial swelling and cell death by allowing the passage of ions and proteins between the mitochondria and the cytosol^13^^,^^14^. Although, the exact structure of the mPTP still remains elusive, many studies indicate that the mPTP is a multiprotein complex composed of cyclophilin D (CypD), the voltage-dependent anion channel 1(VDAC1), and TSPO. Compounds targeting CypD^15^^,^^16^ and TSPO^14^ have demonstrated neuroprotective activity by blocking the formation of the mPTP, specifically in Aβ-induced mitochondrial dysfunction^17^^,^^18^. In addition, the administration of TSPO ligands recovered ΔΨ_m_ and ATP production, disrupted by the treatment of Aβ *in vitro*^19^, but also improved cognitive function of transgenic AD model mice^20^. Another recent study also suggest that TSPO ligands may inhibit ROS generation by blocking the interaction between TSPO and VDAC1^21^, supporting that TSPO is a viable target for the treatment AD^22^^,^^23^.

**Figure 1.**
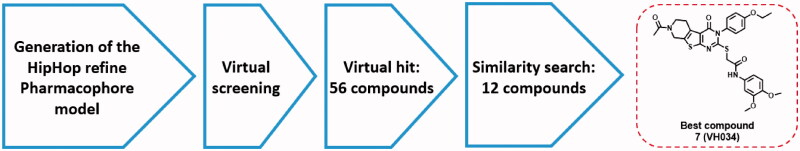
Virtual screening process to identify the best hit compound.

In this work, we aim to find novel TSPO ligands that can restore mitochondrial function in cellular and animal models of AD. Previously developed TSPO ligands mostly consist of either a benzodiazepine or a steroid scaffold, because TSPO was initially thought to be expressed only in the periphery with a propensity to bind benzodiazepines such as diazepam^24^^,^^25^. Given that the structures of the bacterial and mouse TSPO have been identified very recently^26^^,^^27^, we believe that this new structural information will allow us to find TSPO ligands with a novel scaffold that can be readily optimised for further therapeutic development. To this end, we performed a ligand-based pharmacophore modelling, followed by a virtual screening as described in Figure 1. We identified several hit compounds, and performed *in vitro* mitochondrial functional assays to evaluate biological activity. We further optimised these hit compounds via a similarity search and *in vitro* assays, and found two most active compounds, **7** (**VH034**) and **14** (**VH062**). Next, we designed and synthesised a library of TSPO ligands based on the structures of the two compounds. We evaluated biological activities of the synthesised ligands *in vitro* and *in vivo* to identify novel TSPO ligands that can recover neuronal cells from Aβ-induced mitochondrial dysfunction, but also improve cognitive impairment in AD model mice. We also performed pharmacokinetic analysis and docking studies to validate the activity of the identified ligands.

## 2. Materials and methods

### 2.1. Generation and validation of common feature pharmacophore model

Six TSPO ligands were collected from Integrity, and four TSPO ligands from the Integrity database of Prous (www.prous.com) were selected for HipHop pharmacophore analysis. The conformational models of selected compounds having up to 250 conformers were built using the “best conformer generation” method with a 20 kcal/mol energy cut-off. HipHop pharmacophore models were derived by comparing a set of conformational models and a number of three-dimensional configurations of chemical features shared among the training set molecules. The parameter settings of Maximum Omitted Features, Misses, and Complete Misses were varied to generate multiple hypotheses in which some compounds may or may not fit all features. Three chemical functions with hydrogen bonding acceptor, hydrogen bonding donor, and hydrophobic group were used as the pharmacophore features. The hypothesis generation process in Catalyst was returned 10 possible pharmacophore hypotheses having different arrangements of constituent features or ranking scores. After deleting the redundant hypotheses that have the same chemical characteristics and nearly the same distances between these functions, diverse configurations of hypotheses were selected according to ranking scores and fitting scores. The best hypothesis was determined by comparing the highest fit value of training set compounds.

### 2.2. Virtual screening using an external library

Virtual screening was carried out by combining two HipHop Refine models and one shape-added pharmacophore model to obtain new compounds with desired activity profiles. The commercial library Asinex (AsinexGold, 229,398 compounds, AsinexPlatinum, 125,231 compounds, Asinex, Moscow, Russia, www.asinex.com) and ChemDiv (693,042 compounds, ChemDiv, Inc., San Diego, CA, USA, www.chemdiv.com) have been utilised for virtual screening. The best pharmacophore model was used for the virtual screening experiment, by selecting the fast flexible database search option. From the initial screening, compounds with similar functional and spatial properties defined in 3D pharmacophore query were selected on the basis of their fit values. Compounds obtained by fit values were further filtered by visual inspection. The selected compounds were purchased for biological assays.

### 2.3. Synthesis

All reagents were obtained from commercial sources and used without further purification. All reactions were performed under a nitrogen atmosphere in oven-dried glassware. Reactions were monitored by analytical thin-layer chromatography (TLC) plates (Merck, catalog no. 1.05715) with spots visualised by UV light (λ = 254 nm) or using a KMnO_4_ solution. Solvents were evaporated using a rotary evaporator under a reduced pressure of ∼50 mBar. The reaction products were purified by flash column chromatography using silica gel 60 (Merck, catalog nos. 1.07734). Melting points were determined using an OptiMelt melting point apparatus (Stanford Research System, Inc.) in open capillary tube without correction. ^1^H (300 or 400 MHz) and ^13^C (75 or 100 MHz) NMR spectra were recorded using tetramethylsilane (TMS) as the internal standard. Chemical shifts (*δ*) are reported in parts per million (ppm) values relative to TMS, and the coupling constants (*J*) are reported in hertz (Hz). The purity (≥95%) of the samples was determined by analytical HPLC using a Waters E2695 system with SunFire C_18_ column (4.6 mm × 150 mm; 5 µm). HPLC data were recorded using the parameters as follows: H_2_O/MeCN, 90/10 → 0/100 in 20 min, +3 min isocratic, flow rate of 1.0 ml/min, λ = 254 and 280 nm. High–resolution mass spectra (HRMS) were recorded on an LTQ Orbitrap (Thermo Electron Corporation) instrument. Reaction yields are for purified products. Detailed experiment procedures and characterisation of all synthesised compounds can be found in the Supporting Information.

### 2.4. Cell culture

HT-22 (mouse hippocampal cells) cells were grown in Dulbecco’s Modified Eagle’s Medium (DMEM, GIBCO) supplemented with 10% (vol/vol) FBS and antibiotics (100 mg/mL penicillin/streptomycin mix) in a humidified atmosphere at 37 °C with 5% CO_2_.

### 2.5. Preparation of Aβ_1–42_

To prepare a homogenous solution of monomeric Aβ, Aβ_1–42_ peptide (American Peptide) was mixed with 1 ml of 1,1,1,3,3,3 hexafluoro-2-propanol (HFIP; Sigma–Aldrich) and vortexed gently. Then, the solution was aliquoted into microcentrifuge tubes and lyophilised to prepare the Aβ_1–42_ samples as dried peptide films. The prepared Aβ_1–42_ samples were stored at −80 °C and used immediately for cell-based assays to minimise possible aggregation of Aβ itself. Before the use of Aβ_1–42_ samples, the amount of protein was quantified by the BCA protein assay (Pierce BCA protein assay kit; Thermo Scientific). The prepared Aβ_1–42_ samples were suspended with anhydrous dimethyl sulfoxide (DMSO) to a concentration of 5 mM, and used for each cell-based assay.

### 2.6. JC-1 assay

Thirty thousand HT-22 cells per well were seeded into a clear 96-well plate (FALCON) at 200 µL per well one day prior to assay. 750 mM of JC-1 (Stratagene) in DMSO stock solution was dissolved into phenol red-free Opti-MEM (GIBCO) medium to make final concentration of 7.5 mM JC-1 per well. Medium was removed from the plate, and 100 µL per well of JC-1 was added. The sample plates were incubated for 1 h and 15 min at 37 °C and washed twice with 100 µL per well of PBS. Subsequently, cells were treated with a 25 µL solution of each compound at 10 µM in Opti-MEM and incubated at 37 °C for 10 min followed by addition of a 25 µL of Aβ (American peptide, 1-42) solution at 10 µM. Fluorescence was measured at every one hour for three hours at ex/em 530 nm/580 nm (“red”) and ex/em 485 nm/530 nm (“green”) by using the Flexstation^®^ 3 (Molecular Devices, USA) reader. The ratio of green to red fluorescence was calculated and normalised by taking the percent changes using vehicle control as 100%.

### 2.7. Luciferase-based ATP assay

Ten thousand HT-22 cells per well were seeded into a clear 96-well plate (FALCON) one day prior to assay. Medium was removed from the plate, and cells were treated with a 25 µL solution of each compound at 10 µM and incubated at 37 °C for 10 min followed by the addition of a 25 µL of Aβ (American peptide) solution at 10 µM. Cells were incubated at 37 °C for 7 h and washed twice with PBS. Cells were lysed by using 1% Triton-X 100 in TBST buffer solution and the protein concentrations were determined by using the BCA protein determination kit (Thermo scientific). An equal amount of cell lysates from each well was plated into a white 96-well plate (NUNC) and the amount of ATP generated in each sample was determined by using the ATP determination kit (Molecular Probes, USA) containing _D_-luciferin and luciferase. The % inhibition value was calculated by measuring luminescence from each sample (Flexstation^®^ 3, Molecular Devices, USA), and comparing the ATP levels of the vehicle control treated with Aβ as a negative control. Cell viability was also calculated based on the ATP levels of each sample without the treatment of Aβ solution.

### 2.8. MTTassay

To a clear 96-well plate (FALCON), the cultured HT-22 cells were seeded in a number of 5,000 per well 24 h prior to the assay. A solution of each test compound (5 µM, 25 µL) was added to each well. After 24 h of incubation at 37 °C, the cells were treated with a 10 µL of MTT solution (3–(4,5-dimethylthiazol-2-yl)-2,5-diphenyltetrazolium bromide, Sigma-Aldrich), incubated for 2 h at 37 °C, and were treated with 135 µL of MTT solubilising solution (10% Triton-X 100 in isopropanol with 0.1 M HCl) for 2 h at 37 °C. The % values of cell viability were determined by using the optical density (OD) values at 560 nm, and normalised by taking the vehicle control (5 µM of DMSO) as 0%.

### 2.9. CYP inhibition assay

Human CYP450 (CYP1A2, 2D6, 2C9, 2C19, and 3A4) activities were determined by using the Vivid^®^ CYP450 screening kit (Invitrogen, Madison, WI, USA) in a clear 96-well plate. Five positive controls were used as 10 mM solutions in MeCN for five different CYP450 enzymes: CYP1A2 (α-naphthoflavone), CYP2D6 (quinidine), CYP2C9 (sulfaphenazole), CYP2C19 (lansoprazole), and CYP3A4 (ketoconazole). Each sample (test compounds, positive inhibition controls, and solvent controls) was added into each well with the Master Pre-Mix [CYP450 BACULOSOMES^®^ Reagent (recombinant human CYP450 isozyme and rabbit NADPHP450 reductase) and Regeneration System (3.3 mM glucose-6-phosphate and 0.3 U/ml glucose-6-phosphate dehydrogenase in 100 mM potassium phosphate, pH 8.0)]. The mixture was incubated for 5 min at 37 °C, and the Vivid^®^ CYP substrates and 0.1 mM NADP^+^ buffer were added to initiate the CYP450 enzyme reaction. The % remaining activities of CYP450 were measured after 20 min by using a fluorescent plate reader (Flexstation^®^ 3, Molecular Devices, USA).

### 2.10. Measurement of liver microsomal stability

Liver microsomes (human, dog, rat, and mouse; 0.5 mg/mL) were incubated with 1 µM of each test compound in the presence of potassium phosphate buffer (PPB) for 5 min at 37 °C. A solution of the NADPH regeneration system (#44332000, Oriental Yeast Co., Japan) was added to begin metabolic reactions of liver microsomes. The resulting mixtures were incubated for 30 min at 37 °C, and the reaction was terminated by the addition of a cold MeCN solution containing an internal standard (chlorpropamide). The mixture was separated by centrifugation (14,000 rpm for 5 min at 4 °C), and the supernatant was analysed by LC-MS/MS (Shimadzu Nexera XR system and TSQ vantage; Thermo Scientific).

### 2.11. hERG inhibition assay

For automated patch-clamp NPC-16 Patchliner (Nanion Technologies, München, Germany) recordings, CHO-K1 Tet-On hERG cells (IonGate Biosciences GmbH, Frankfurt, Germany) were plated into 100-mm cell culture dishes. Whole-cell currents were recorded with the intracellular solution containing: 50 mM KCl, 60 mM KF, 10 mM NaCl, 2 mM MgCl_2_, 20 mM EGTA and 10 mM HEPES (pH 7.2), and with the extracellular solution containing: 140 mM NaCl, 4 mM KCl, 2 mM CaCl_2_, 1 mM MgCl_2_, 5 mM Glucose and 10 HEPES (pH 7.4). To assist stable seal formations, the seal enhancer containing: 80 mM NaCl, 3 mM KCl, 35 mM CaCl_2_, 10 mM MgCl_2_ and 10 mM HEPES (pH 7.4) was used only at the seal formation step. Prior to the whole-cell recordings, the external seal enhancing solution was exchanged to the extracellular solution described above. hERG channel currents were recorded using the parallel EPC-10 patch-clamp amplifiers (HEKA Elektronik, Lambrecht/Pfalz, Germany), and low-pass filtered (10 kHz) with a 4-pole Bessel filter. Cell suspension and patch solutions were automatically added onto the four recording wells in the microfabricated disposable chip (NPC-16 Chip, Nanion Technologies, München, Germany). To obtain the inhibitory constants, hERG tail currents were evoked by repolarizing steps to −50 mV for 500 ms preceded by a 500-ms depolarisation potential of +20 mV at a holding potential of −80 mV with a 20-s sweep interval. Whole-cell currents were acquired and digitised at 5 kHz using Patchmaster (HEKA Elektronik, Lambrecht/Pfalz, Germany). The extracellular solution was exchanged to the extracellular solution containing each blocker via four pipette tips of NPC-16 Patchliner using a 4-fold volume of solution (40 µL) with a speed of 4 µL/s, and the exchanged blocker solution was applied for 100–200 s to the patch-clamped cells until blocker binding had reached equilibrium by monitoring hERG tail currents. Whole-cell recordings were analysed using the Patchmaster/Fitmaster (HEKA Elektronik, Lambrecht/Pfalz, Germany), IGOR Pro (WaveMetrics Inc., Portland, OR, USA), and the GraphPad Prism 4.0 (GraphPad Software, Inc., La Jolla, CA, USA) software.

### 2.12. Pharmacokinetic study

Each test compound at a dose of 10 mg/kg was administered intravenously or orally to male Sprague-Dawley (SD) rats. Blood samples collected via carotid artery at several time points after administration of each compound were stored at −70 °C until LC-MS/MS analysis. To determine the plasma concentration of each compound, a 0.1 µg/mL of internal standard in 100 µL of MeCN was added to 50 µL of plasma samples. After vortex-shaking and centrifugation at 10,000 rpm for 10 min, 5 µL of the supernatant was analysed by LC-MS/MS [HP1100^®^ HPLC system (Agilent, Santa Clara, CA) combined with API3200^®^ triple-quadrupole mass spectrometer (Applied Biosystems-SCIEX, Concord, Canada)]. The HPLC chromatographic separation was performed on a reversed-phase Xterra^®^ C_18_ column (Waters Corporation, Milford, MA) using gradient elution consisted of 0.1% formic acid and 0.1% formic acid in 90% MeCN at a flow rate of 0.3 ml/min.

### 2.13. Animals

The acute AD mice model was prepared by administration of Aβ_1-42_ solution (10% DMSO/90% PBS) to male ICR mice (6 weeks old, 30 − 33 g) via intracerebroventricular (ICV) injection (Aβ_1-42_ dose: 500 pmol/mouse) as described previously^28^. The wild type mice (B6C3F1) and double APP/PS1 transgenic mice (Tg AD mice model; APPswe/PSEN1dE9) were purchased from Jackson Laboratory (Bar Harbour, ME, USA). The Tg AD model mice were 11 months old at the beginning of the behaviour test, and were housed for 1 month in a room under controlled temperature and fed *ad libitum*. The behaviour tests were conducted during daytime in air-controlled and soundproof experiment room. The animal experiments were abided by the guidelines of the Institutional Animal Care and Use Committee of Korea Institute of Science and Technology.

### 2.14. Y-Maze spontaneous alternation test

To the mice of the acute AD model (*n* = 7 per group), each test compound (test compound in 20% cyclodextrin, 30 mg/kg) was intravenously injected daily for 6 days. The Y-maze spontaneous alternation test was performed 1 day after the administration of each test compound. The Y maze was made of three equally spaced black plastic arms (40 L × 10 W × 12 H cm) positioned at an equal angle. Each mouse was placed at the end of one arm and allowed to move freely through the maze for 8 min, and the sequence of arm entries was recorded. The arm entry of the mouse was counted when the all four limbs were within an arm. A set of entries into all three arms is considered as a spontaneous alternation. The spontaneous alternation (%) was calculated by the following equation:

$$\text{Spontaneous alternation }\left(\%\right)=\text{1}00x\left[\frac{\text{number of alternations}}{\left(\text{total number of arm entries }–\text{ 2}\right)}\right]$$

### 2.15. Contextual fear conditioning test

To the Tg mice of the AD model (*n* = 7 per group), each test compound (30 mg/kg) was orally administrated daily for 1 month. Each mouse was placed in a fear-conditioning chamber (Courlbourn, USA) for 90 s before giving an electric shock. Trials of training were performed using the chamber equipped with a fear conditioning system (FreActimetrics, USA). The training was performed by giving a conditional stimulus (CS) of 75 dB sound for 20 s followed by an unconditional stimulus (US) of electric foot shock (0.5 mA) for the last 2 s in CS. After an additional stay for 1 min, the mouse was returned to its home cage. Fear conditioning test was conducted 24 h after the training. The mouse was placed in the same chamber for 5 min without presentation of CS. Freezing behaviour was considered as the complete absence of any movement except for respiration and heartbeat. Freezing response was measured by the fear conditioning system without application of CS or US.

### 2.16. Surface plasmon resonance (SPR) measurements

To determine the binding affinities of the selected compounds, the *K*_D_ values were measured by using Biacore T200 optical biosensor system (GE Healthcare). The recombinant human TSPO (18 kDa, Origene Technologies, Inc., No.: TP320107) was covalently immobilised on a CM5 chip (GE Healthcare) at 25 °C *via* standard amine-coupling protocols in 10 mM sodium acetate (pH 4.5) at a flow rate of 10 µL/min for 1500s (immobilised protein densities: 5,000–6,000 response unit (RU)). The tested compounds were initially prepared at 10 mM in DMSO, and serially diluted in PBS-P buffer (10 mM NaH_2_PO_4_, 150 mM NaCl, pH 7.4) to 1% DMSO. For each tested compound, the kinetic values were measured using a five-point concentration series, 0.001, 0.01, 0.5, 1, and 5 µM. Multiple blank samples of running buffer alone (0 µM of tested compounds) were incorporated in every measurement. The filtered PBS-P buffer containing 1% DMSO was used as a running buffer during the SPR experiments. To measure the kinetic values, a series of different concentrations were injected over the immobilised chip at a flow rate of 50 µL/min (contact time: 120 s, dissociation time: 300 s), followed by the regeneration process at a flow rate of 50 µL/min (contact time: 120 s, stabilisation time: 10 s). The *K*_D_ value of each compound was calculated by Biacore T200 evaluation software (GE Healthcare) after the standard solvent correction process.

### 2.17. Docking study

A homology model of human TSPO was prepared using Discovery studio 2017 R2 client software1. The human TSPO sequence (UniProt ID: P30536) was taken from UniProt database. The high-resolution crystal structure of the translocator protein 18 kDa (RCSB ID: 4UC1) from Rhodobacter sphaeroides (Sequence Identity: 33.7%, sequence similarity: 49.7%) was used as a template for the homology model generation. Docking studies were performed with XP docking methodology implemented in Maestro, Schrödinger software2. Ligands were prepared for the energy-minimised structures using OPLS_2005 force field. Proteins were prepared using the protein preparation wizard. Hydrogens were added and water molecules beyond 5 Å from hetero groups were removed. The prepared ligands were docked using the extra precision (XP) method and flexible ligand sampling. Docking images were prepared using Discovery studio software.

## 3. Results and discussion

### 3.1. Generation of a pharmacophore model

**Figure 2.**
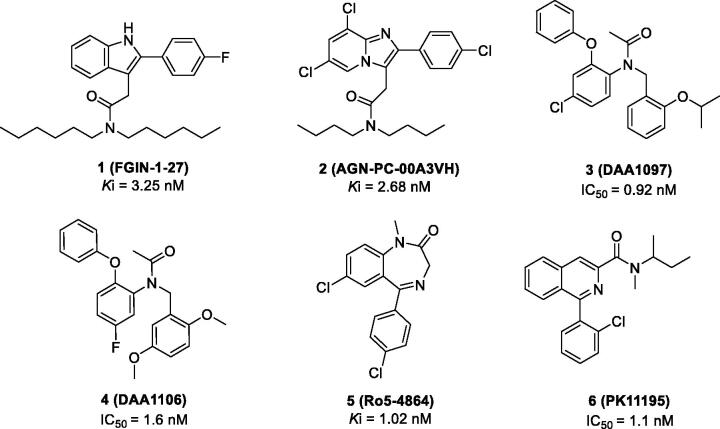
Representative TSPO ligands used as a training set to generate the pharmacophore model.

We generated common feature pharmacophore models by using six known TSPO ligands (compounds **1**–**6**) from the Integrity^®^ database of Prous (Figure 2). These compounds showed potent TSPO binding affinities within the nanomolar range: 2–(2-(4-fluorophenyl)-1*H*-indol-3-yl)-*N*,*N*-dihexylacetamide (**FGIN-1–27**, *K*i = 3.25 nM)^29^, *N,N*-dibutyl-2–(6,8-dichloro-2–(4-chloro-phenyl)imidazo[1,2-*a*]pyridin-3-yl)acetamide (**AGN-PC-00A3VH**, *K*i = 2.68 nM)^30^, *N*-(4-chloro-2-phenoxyphenyl)-*N*-(2-isopropoxybenzyl)acetamide (**DAA1097**, IC_50_ = 0.92 nM)^31^, *N*-(2,5-dimethoxybenzyl)-*N*-(5-fluoro-2-phenoxyphenyl)acetamide (**DAA1106**, IC_50_ = 1.6 nM)^31^, 7-chloro-5–(4-chlorophenyl)-1-methyl-1,3-dihydro-2H-benzo[*e*][1,4]diazepin-2-one (**Ro5-4864**, *K*i = 1.02 nM)^32^, and *N*-(*sec*-butyl)-1–(2-chlorophenyl)-*N*-methylisoquinoline-3-carboxamide (**PK-11195**, IC_50_ = 1.1 nM)^33^.

**Figure 3.**
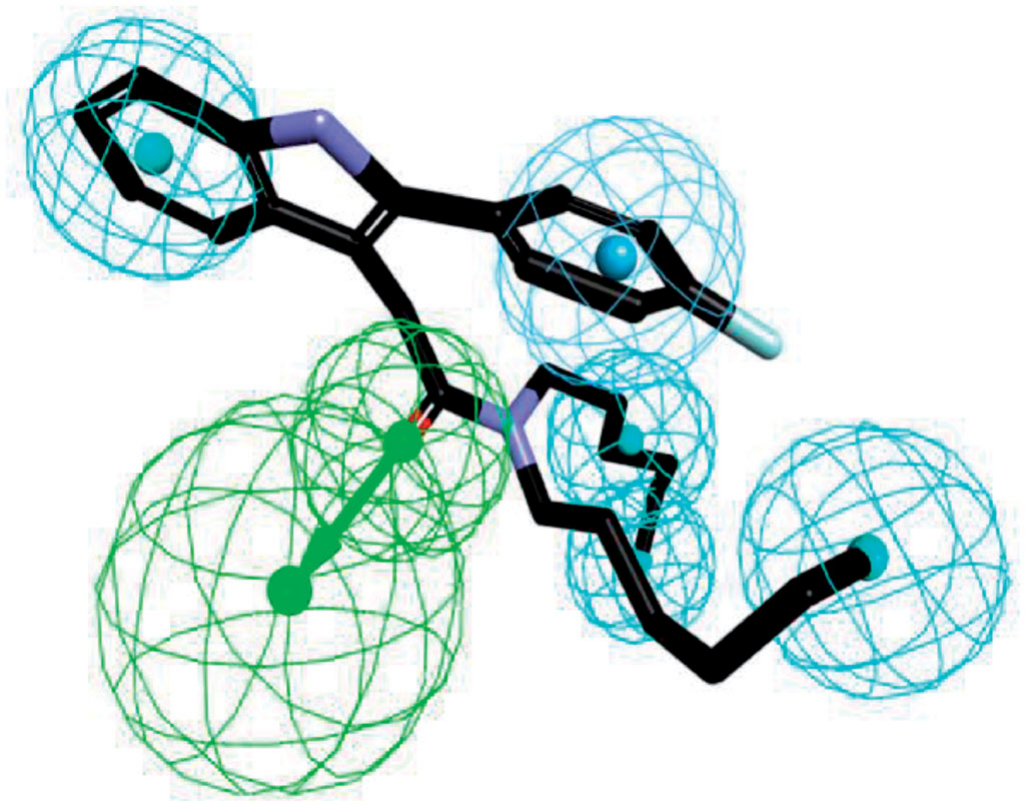
The ligand-based pharmacophore model mapping with FGIN-1–27 (**1**). (C atom, black; N atom, blue; O atom, red; F atom, cyan). The pharmacophore features are drawn as colour-coded spheres: H-bond acceptor, green; hydrophobic feature, light blue; and ring aromatic feature, blue.

We applied HipHop pharmacophore module^34^^,^^35^ which does not use any activity data of the training set compounds; instead, it generates hypotheses by taking each training set compound as a reference to accommodate structural features of all the compounds. Since this method assumes that all the compounds in the training set are equally important, all of the chemical features in each compound will be considered to build a hypothesis space. Next, in order to get more common features, the pharmacophore models were refined by removing one training set compound in each run. The final pharmacophore model was generated by differing the aromatic ring and hydrophobic feature options as described in Figure 3. The final pharmacophore contains a hydrogen bond acceptor, an aromatic ring and four hydrophobic features. Compound **FGIN-1–27** overlapped with the model, showing the oxygen atom map with a hydrogen bond acceptor feature, the fluoro-benzene part with a hydrophobic aromatic feature, and the benzene of the indole and *n*-alkyl chains of amide part with four hydrophobic features.

### 3.2. Virtual screening and *in vitro* assays

We screened commercially available libraries from Asinex (AsinexGold, 229,398 compounds; AsinexPlatinum, 125,231 compounds) and ChemDiv (693,042 compounds) against our pharmacophore model (Figure 3). Through the BEST flexible search of the databases, 47,268 compounds were found as initial hits from the HipHop pharmacophore model by the fit value (3.50 out of 6.00). Among them, 56 compounds (**VH001**–**VH056**; Supplementary Table S1) were manually selected on the basis of their structural diversity and functional groups that are crucial for activity such as the number of heteroatoms, aromatic/non-aromatic ring, *n* (*n* = 5, 6, 7)-membered ring, polycyclic ring, alkyl substituent, and their positions.

The selected 56 compounds were purchased and tested by *in vitro* cell-based assays. Four different cell-based assays (JC-1, MTT, ATP production, and cellular ROS assays) were carried out sequentially. First, the JC-1 assay was performed to screen all 56 compounds, **VH001**–**VH056** (Supplementary Table S1). Since mitochondrial membrane depolarisation is one of the distinct signs of mPTP opening, the mitochondrial uptake of a fluorescent JC-1 dye (5,59,6,69-tetrachloro-1,19,3,39-tetraethylbenzimidazolocarbocyanine iodide) was measured to evaluate the effects of each compound on the mitochondrial membrane potential (ΔΨ_m_). HT22 cells, a mouse hippocampal cell line, were treated with 5 µM of Aβ, incubated with each compound at the same concentration of 5 µM, and subsequently treated with JC-1. On the basis of changes in the red/green fluorescence intensity ratio between the cells treated with Aβ and control, the percent recovery of ΔΨ_m_ for each compound was calculated and normalised to the ΔΨ_m_ of healthy cells as 100%. Two neuroprotective compounds, piracetam and cyclosporine A (CsA), were also tested for comparison, because these compounds have demonstrated neuroprotective activity by restoring Aβ-induced mitochondrial dysfunction^36^ or by inhibiting prolonged opening of the mPTP^37^.

Based on the JC-1 assay results measured from piracetam (60% at 5 µM) and CsA (55% at 5 µM), we selected compounds exhibiting greater than 50% of the percent recovery values for additional *in vitro* assays. Among 56 compounds, 23 compounds were selected and further tested for the luciferase-based ATP assay. It should be noted that the two reference compounds, piracetam and CsA, appeared to demonstrate differing activity, 127% for piracetam and −46% for CsA; it is not clear whether the effect on ΔΨ_m_ can be correlated with ATP production. Compounds **7 (VH034)** and **8 (VH050)** showed the most potent recovery activities (>40%) (Table 1). Thus, we chose these two compounds and further evaluated their biological activity by measuring cell viability and reactive oxygen species (ROS) production. The effects on cell viability of each compound under Aβ-induced cytotoxicity were determined by measuring the metabolic activity of NAD(P)H-dependent cellular oxidoreductases using the 3–(4,5-dimethylthiazol-2-yl)-2,5-diphenyl-tetrazolium bromide (MTT) dye. To measure cellular ROS levels, we used a fluorescence indicator, 2′,7′-dichlorofluorescein diacetate (DCFDA), and calculated the percent inhibition of ROS generation by applying the same method used for MTT assays. Compound **7** inhibited Aβ-induced ROS generation (97% at 5 µM) almost to the level of the untreated control (Table 1). On the other hand, compound **8** did not appear to reduce ROS generation, which is likely due to the toxicity of the compound itself (34% of cell viability from the MTT assay). On the basis of the *in vitro* activity, we selected compound **7** as the best hit from the initial screening library.

**Table 1.** *In vitro* cell-based assay results of **7** and **8**. 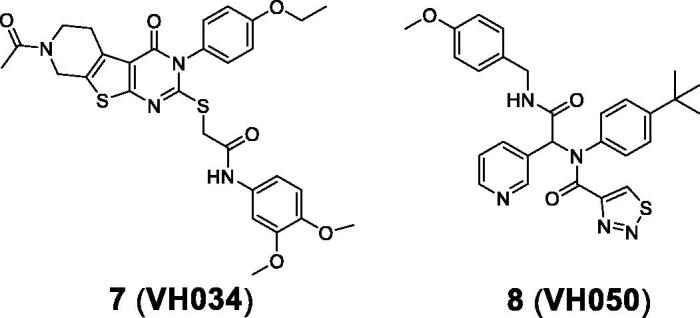

Compound	Recovery of ΔΨ_m_ (%)^a^	ATP Recovery (%)^a^	MTT		ROS Inhibition (%)
			Recovery (%)^a^	Viability (%)^b^	
**7 (VH034)**	78%	42%	28%	95%	97%
**8 (VH050)**	92%	45%	−184%	34%	−10%
**Piracetam**	60%	127%	29%	132%	129%
**CsA**	55%	−46%	61%	90%	ND

^a^Recovery percent values were calculated by normalising each measured value against the untreated control; for example, 0% (Aβ-induced damaged condition) and 100% (normal condition in the absence of Aβ).

^b^HT22 cell viability after 24 h of the treatment with 5 μM of each compound.

ND: Not determined.

### 3.3. Additional similarity search and *in vitro* activity of the identified hit compounds

To explore biological activities of structurally similar compounds with **7**, a similarity search was carried out by using **7** as a structural template. A total of 12 compounds (**9**–2**0**; Table 2) were selected from a commercial database and purchased for *in vitro* assays. These newly selected compounds possess the same tricyclic thienopyrimidine core; compounds **9**–**12** contain an alkyl chain connected via the sulphur atom of the tricyclic core ring, while compound **13** bears a polar amide group at the same position. Compounds **14**–**16** contain a lipophilic aromatic group at the same sulphur atom. In compounds **17**–**20**, the sulphur side chain is replaced with a carbonyl group, and both the two nitrogens in the pyrimidine core have aromatic substituents, adding additional hydrophobic groups compared with compounds **9**–**16**. Compounds with a thioalkyl group (**9**–**12**) are less effective in recovering ΔΨ_m_ than compound **7**, whereas compounds with an additional aromatic side chain with no sulphur atom in the core (**17**–**20**) have no ATP recovering effect, suggesting that the aromatic side chain connected through the sulphur atom is important for mitochondrial function. Overall, **14 (VH062)** is the most potent compound in four different cell-based assays without any noticeable cytotoxicity (Table 2). Specifically, compound **14** appeared to be highly effective in recovering ATP production and metabolic function measured by MTT assays, comparable to the levels of untreated cells. Considering that **14** has a smaller R_1_ substituent (methoxy versus ethoxy group), and highly electron-withdrawing aromatic substituents in the R_2_ benzamide ring (difluoro versus dimethoxy group) compared with **7**, the SAR at these two positions need to be investigated more thoroughly. We will describe the additional SAR in the later section (*3.6*, compound **27**–**62**).

**Table 2.** *In vitro* cell-based assay results of **9–20**. 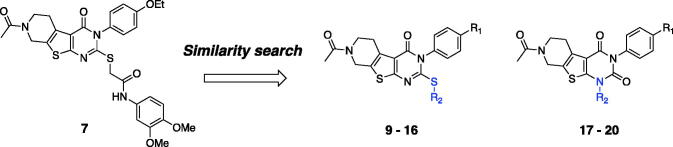

Compound	R_1_	R_2_	Recovery of ΔΨ_m_ (%)	ATP Recovery (%)	MTT		ROS Inhibition (%)
					Recovery (%)	Viability (%)	
**7 (VH034)**			78%	42%	28%	95%	97%
**9 (VH057)**	OMe	Me	30%	ND	ND	ND	ND
**10** (**VH058**)	OEt	Me	52%	49%	45%	87%	−177%
**11** (**VH059**)	OEt	*i*-Bu	59%	ND	ND	ND	−234%
**12** (**VH060**)	OEt	*n*-Bu	1%	ND	ND	ND	ND
**13** (**VH061**)	OMe		66%	14%	ND	ND	ND
**14 (VH062)**	OMe		77%	122%	92%	120%	136%
**15** (**VH063**)	OEt		86%	31%	59%	139%	−4%
**16** (**VH064**)	OMe		74%	38%	32%	135%	112%
**17** (**VH065**)			72%	−105%	ND	ND	ND
**18** (**VH066**)			53%	−18%	ND	ND	ND
**19** (**VH067**)			73%	−77%	ND	ND	ND
**20** (**VH068**)			50%	−55%	ND	ND	ND
**Piracetam**			60%	127%	29%	132%	129%
**CsA**			55%	−46%	61%	90%	NDa

ND: Not determined.

### 3.4. *In vivo* activity of the most active hit compounds, 7 (VH034) and 14 (VH062)

**Figure 4.**
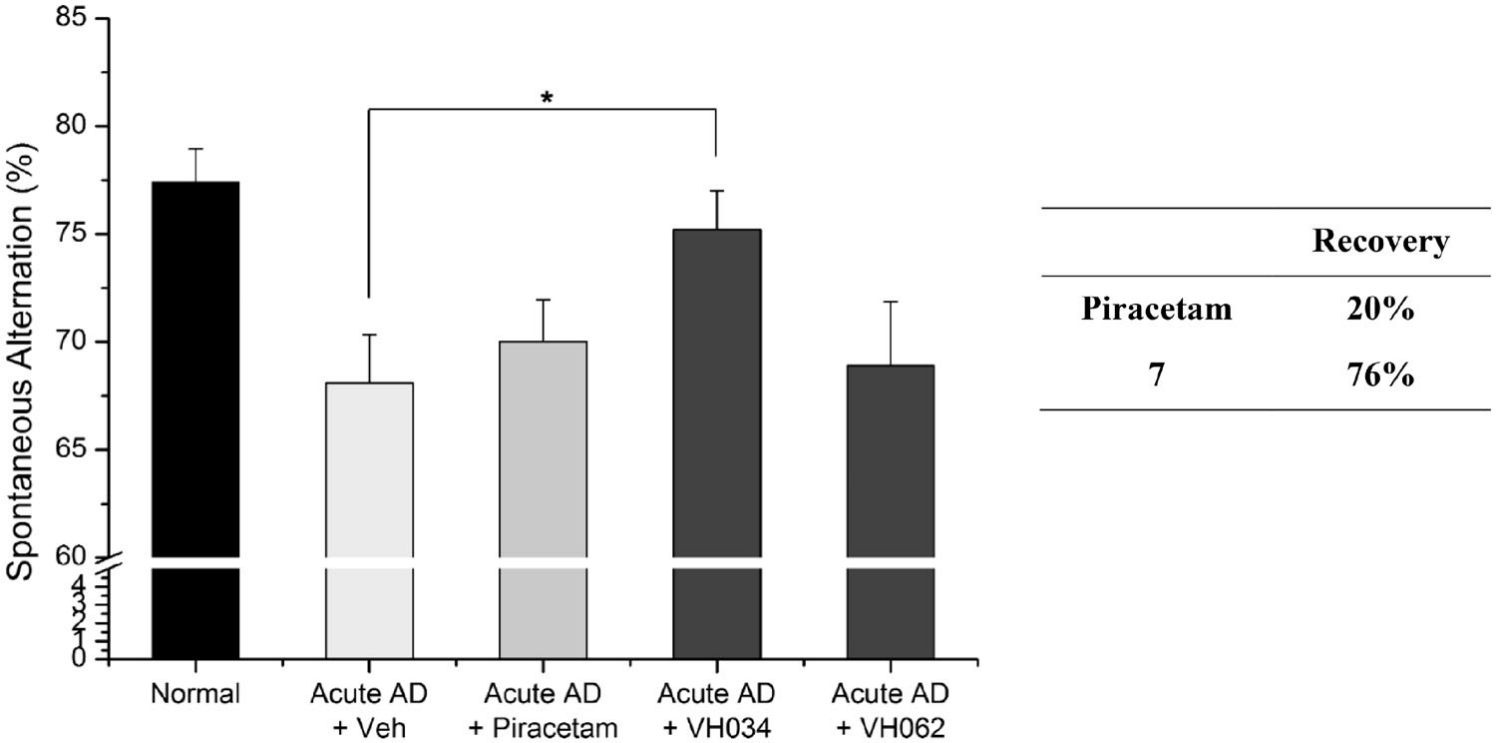
Recovery of Y-maze spontaneous alternations in an acute AD model by the treatment of compounds **7** or **14** (30 mg/kg daily, 6 days, i.p.) Each group was evaluated by comparing with the piracetam-treated group (30 mg/kg daily, 6 days, i.p.). Data are expressed as a mean ± SEM (*n* = 7 per group): *p < 0.05.

We evaluated *in vivo* activity of **7** and **14** in an acute AD mouse model. The acute AD model mice were generated by the administration of Aβ_1-42_ peptide (500 pmol) according to the previously reported method^28^, and each compound (30 mg/kg daily) was administered intraperitoneally for 6 days. Piracetam (30 mg/kg daily) was also administered in parallel for comparison. The Y-maze spontaneous alternation tests were performed to measure the recovery of learning and memory deficit in acute AD model mice^38^^,^^39^. After 6 days of administration, we found that compound **7** alleviated spatial learning and memory deficits significantly, restoring 76% of Aβ-induced memory impairments in acute AD model mice (Figure 4). In contrast, compound **14** did not show any comparable activity, and the piracetam-treated mice only recovered 20% of spatial learning and memory deficits. We believe that the treatment of **7** was beneficial for ameliorating Aβ-induced cognitive impairments.

**Figure 5.**
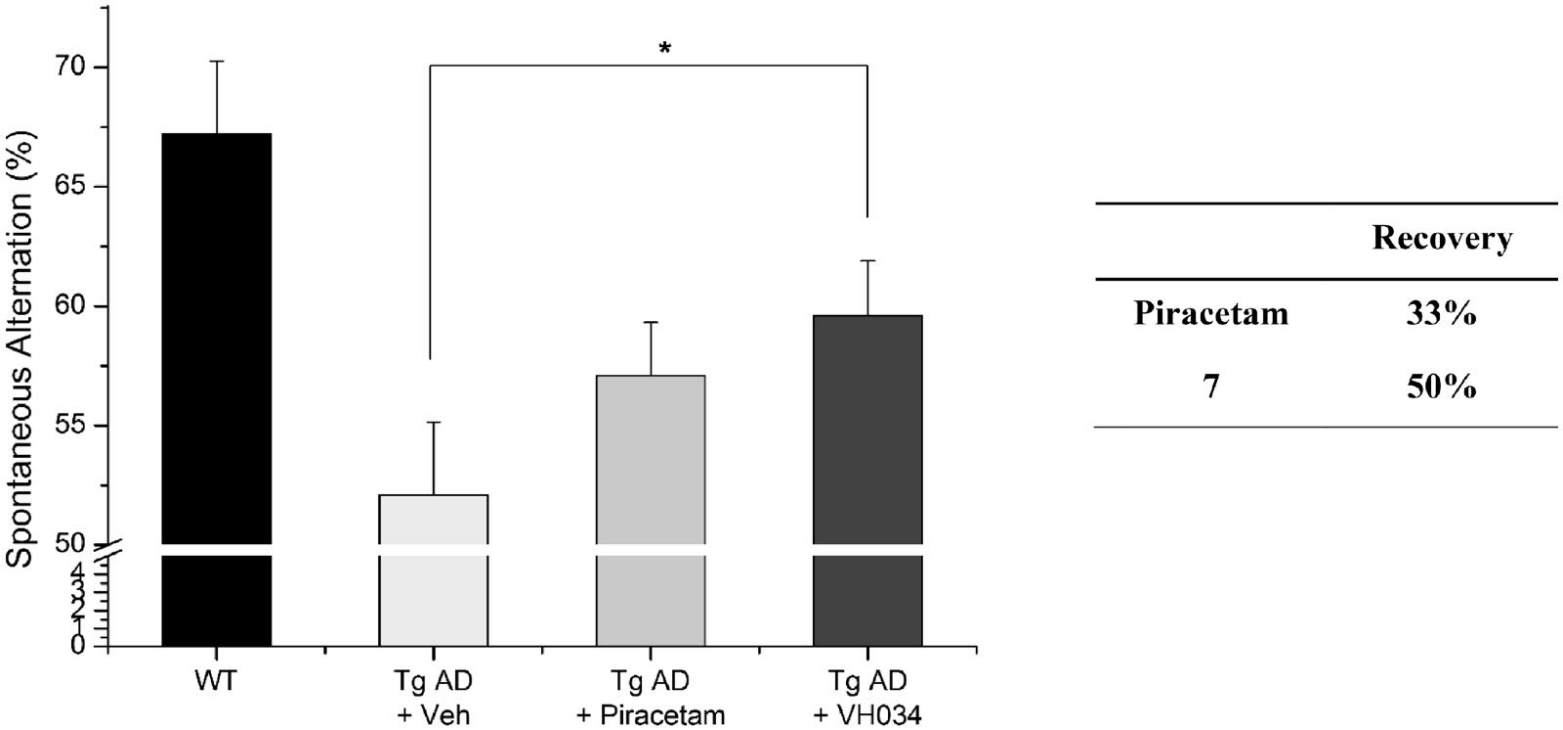
Recovery of Y-maze spontaneous alternations in transgenic (Tg; APPswe/PSEN1dE9 2x TG) AD model mice by the treatment of **7**. Each group was evaluated by comparing with the piracetam-treated group (30 mg/kg daily, 1 month, PO). Data are expressed as a mean ± SEM (*n* = 7 per group): *p < 0.05.

To further validate the therapeutic effects of compound **7**, we performed the Y-maze tests by using a transgenic (Tg) mouse model of AD (APPswe/PSEN1dE9 2xTG) aged 11 months. Compound **7** was administrated to the Tg AD model mice for 1 month (30 mg/kg daily) and the Y-maze tests were performed at 12 months of age. As shown in Figures 5 and 7 restored 50% of spatial learning and memory deficits, whereas piracetam recovered 33% of the deficits. Overall, based on the results of the Y-maze behaviour tests, compound **7** showed greater recovery than piracetam in both transgenic and acute AD model mice.

**Figure 6.**
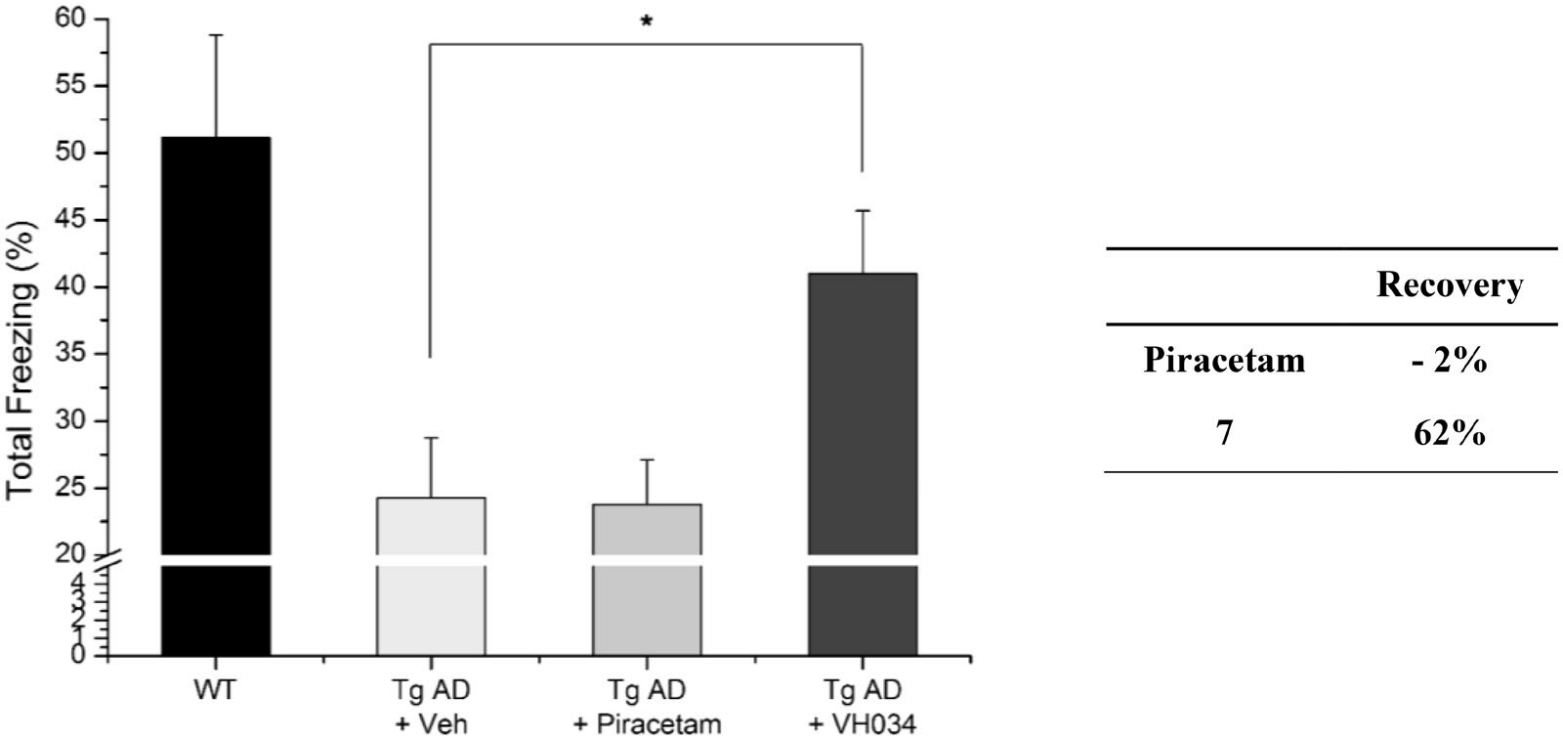
Contextual fear conditioning tests in wildtype and transgenic AD model mice. Each group was evaluated by comparing with the piracetam-treated group (30 mg/kg daily, 1 month, PO). Data are expressed as a mean ± SEM (*n* = 7 per group): *p < 0.05.

In addition, we assessed recovery effects of compound **7** by performing contextual fear conditioning tests.^40^ Healthy mice show higher number of total freezing, which is an index of fear learning and memory of rodents; therefore, the number of total freezing responses under fear-relevant stimuli was measured with the compound-treated Tg AD model mice. As shown in Figure 6, the treatment of **7** restored cognitive function more effectively compared to piracetam-treated mice, which corresponds to the observations from the Y-maze tests. Specifically, the piracetam-treated Tg mice were not affected by the treatment (percent recovery = −2%), whereas **7**–treated mice demonstrated a remarkable improvement in cognitive function (62%). Given the excellent *in vivo* activity of compound **7**, as well as the significant *in vivo* recovery effect, we decided to design new derivatives based on the core structure of compounds **7** and **14** to optimise the biological activity.

### 3.5. Synthesis of the designed ligands

**Scheme 1.**
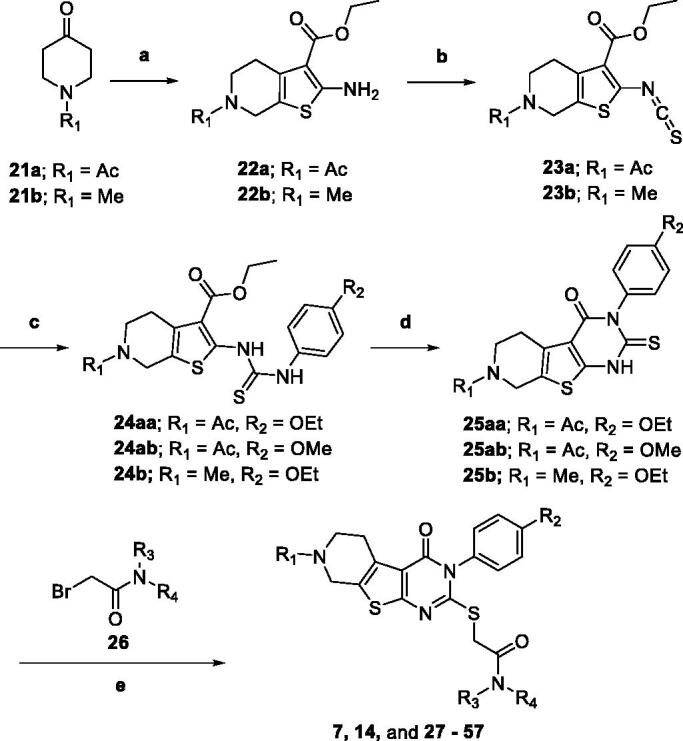
Synthesis of 5,6,7,8-tetrahydropyrido[4',3':4,5]thieno[2,3-*d*]pyrimidin-4(3*H*)-one derivatives **7**, **14**, and **27–57**. Reagents and conditions: (a) ethyl 2-cyanoacetate, Sulphur, Et_3_N, EtOH, reflux, 4 h, 96–100%, (b) 1,1'-thiocarbonylbis(pyridin-2(1*H*)-one), THF, reflux, 4 h, 80–86%, (c) substituted aniline, EtOH, reflux, 12 h, 84–93%, (d) (i) 10% NaOH/MeOH (1:3), reflux 3 h, (ii) 2 N HCl, 93–98%, (e) 2-bromo-*N*-substitued acetamide **26**, Et_3_N, MeCN, reflux, 5 h, 33–98%.

**Scheme 2.**
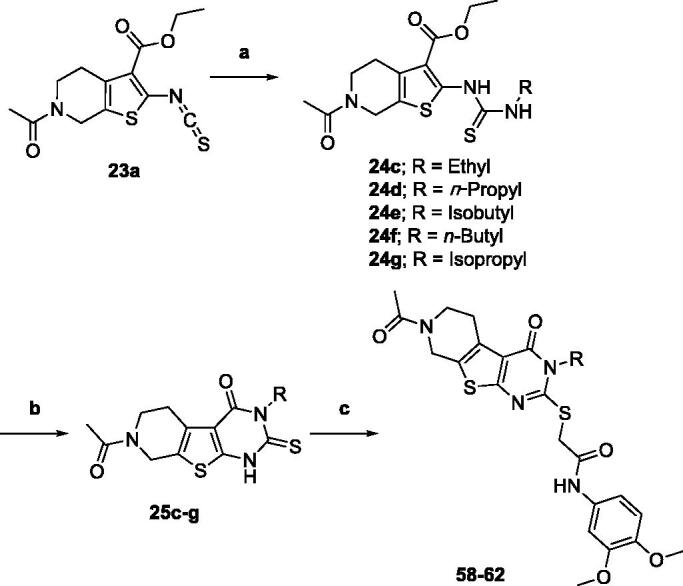
Synthesis of 5,6,7,8-tetrahydropyrido[4',3':4,5]thieno[2,3-*d*]pyrimidin-4(3*H*)-one derivatives **58–62**. Reagents and conditions: (a) substituted amine, EtOH, reflux, 12 h, 57–99%, (b) (i) 10% NaOH/MeOH (1:3), reflux 3 h, (ii) 2 N HCl, 56–96%, (c) 2-bromo-*N*-(3,4-dimethoxyphenyl)acetamide **26**, Et_3_N, MeCN, reflux, 5 h, 50–90%.

Compounds **7**, **14** and their derivatives were prepared as shown in Scheme 1. Starting from commercially available piperidone compounds **21a-b**, the tetrahydrothieno pyridine compounds **22a-b** were generated *via* Gewald reaction^41^. Subsequently, the amino group of compounds **22a-b** were converted to an isothiocyanate group^42^^,^^43^. The addition of *p*-alkoxy anilines to the isothiocyanate, followed by a cyclisation reaction yielded compounds **25aa**-**b**^44^. Finally, compounds **7**, **14** and their derivatives were obtained *via* S*_N_*2 reaction of compounds **25aa-b** with 2-bromo-*N*-substitued acetamide **26**. Additionally, to study SAR of different functional groups (R) at the position of a pendent alkoxy phenyl ring of **7** and **14**, compounds **58**–**62** were designed and synthesised as illustrated in Scheme 2. To synthesise compounds **58**–**62**, we used various alkylamines to diversify R groups (**24c-g**) instead of using the alkoxy anilines.

### 3.6. *In vitro* activity of compounds 27–62

On the basis of our initial cell-based assays (Table 2), we found that the sulphur-linked benzamide group is particularly important for *in vitro* activity while an alkoxy substituent in the aromatic side chain also affected biological activity to some extent. To further explore SAR of these specific positions, we have designed and synthesised additional compounds and tested for *in vitro* activity. Specifically, we fixed the aromatic alkoxy substituent as either a methoxy (**27**) or an ethoxy (**28**–**47**) group; we added an additional substituent (R_2_) at the amide nitrogen while diversified the benzamide substituents (R_3_) as described in Table 3.

**Table 3.** In vitro assay results of compounds **27**–**47**. 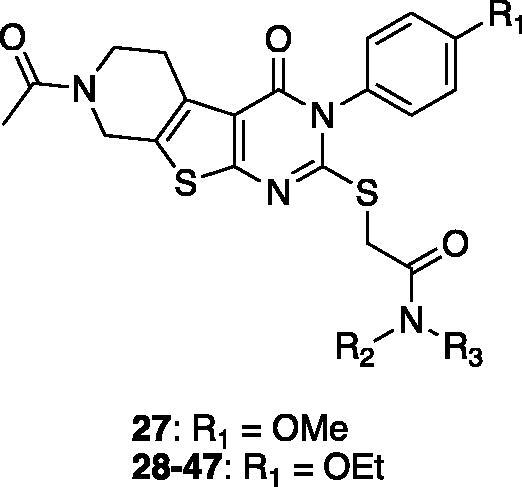

Compound	R_2_	R_3_	Recovery of ΔΨ_m_	ATP recovery	MTT	
					Recovery	Viability
**27**	H	3,4-difluorophenyl	73%	−15%	−4%	92%
**28**	H	2-(Piperidin-1-yl)ethyl	78%	−10%	−22%	71%
**29**	H	3-(Piperidin-1-yl)propyl	69%	−22%	−10%	89%
**30**	H	1-Methylpiperidin-4-yl	68%	60%	6%	98%
**31**	H	Benzyl	81%	59%	18%	109%
**32**	Methyl	Benzyl	83%	7%	8%	88%
**33**	Ethyl	Benzyl	86%	7%	35%	104%
**34**	H	4-Chlorobenzyl	82%	40%	20%	97%
**35**	H	4-Chlorophenyl	82%	38%	18%	107%
**36**	H	4-Methylbenzyl	82%	35%	2%	96%
**37**	Methyl	Methyl	45%	ND	ND	ND
**38**	Ethyl	Ethyl	65%	12%	5%	77%
**39**	*n*-Propyl	*n*-Propyl	79%	15%	26%	99%
**40**	*n*-Butyl	*n*-Butyl	80%	33%	32%	107%
**41**	H	*n*-Propyl	51%	ND	ND	ND
**42**	H	Isopropyl	51%	ND	ND	ND
**43**	H	*n*-Butyl	73%	1%	−27%	79%
**44**	4-Phenylpiperazin-1-yl		83%	63%	9%	97%
**45**	4-Benzylpiperazin-1-yl		78%	54%	−31%	113%
**46**	4-Phenylpiperidin-1-yl		82%	80%	28%	86%
**47**	4-Benzylpiperidin-1-yl		81%	86%	31%	94%
**7**			73%	42%	28%	95%

ND: Not determined.

**Table 4.** *In vitro* assay results of compounds **48**–**57**. 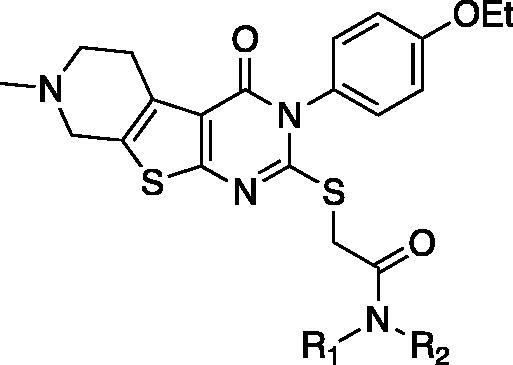

Compound	R_1_	R_2_	Recovery of ΔΨ_m_	ATP Recovery	MTT	
					Recovery	Viability
**48**	H	3,4-Dimethoxyphenyl	55%	62%	35%	106%
**49**	H	Benzyl	61%	36%	42%	99%
**50**	Methyl	Benzyl	78%	32%	33%	95%
**51**	Ethyl	Benzyl	74%	22%	39%	107%
**52**	H	4-Chlorobenzyl	67%	30%	19%	80%
**53**	H	4-Chlorophenyl	65%	10%	−21%	89%
**54**	H	4-Methylbenzyl	57%	1%	2%	107%
**55**	H	4-Methylphenyl	66%	56%	45%	127%
**56**	*n*-Propyl	*n*-Propyl	69%	50%	16%	108%
**57**	*n*-Butyl	*n*-Butyl	73%	50%	66%	110%
**7**			73%	42%	28%	95%

Most derivatives showed excellent ΔΨ_m_ percent recovery values close to or greater than 70%, except compounds **37**, **41**, and **42**. These compounds with low activity have relatively small sized (less than three carbons) alkyl side chains or a single alkyl chain at the amide nitrogen, suggesting that the size and hydrophobicity of the substituent at this position is crucial for cellular activity. Compounds with an aromatic ring (**31**–**36**, **44**–**47**) and a longer alkyl chain (**39**–**40**) at the same position generally showed better activity than compounds with a piperidine group (**28**–**30**), also indicating that overall hydrophobicity of the molecule is important for membrane-associated activity. The percent recovery values of ΔΨ_m_ were enhanced with increasing the length of alkyl chain, in the order of methyl (compound **37**; 45%) < ethyl (compound **38**; 65%) < *n*-propyl (compound **39**; 79%) < *n*-butyl (compound **40**; 80%). In addition, the di-alkyl substituted (R_2_ and R_3_) compounds **37**–**40** showed greater recovery than mono-alkyl substituted (R_3_ only) compounds **41**–**43** from the JC-1 assay. When these R_2_ and R_3_ alkyl groups were replaced with a constrained heterocycle (compounds **44–47**), they demonstrated consistently potent activity (78–83%), indicating that a bulky hydrophobic residue at this specific position is important for recovering Aβ-induced mitochondrial membrane depolarisation.

We further assessed *in vitro* activity of the compounds demonstrated over 60% of recovery in the JC-1 assays by performing the MTT assay and the ATP production assay. Six compounds (**30**–**31**, **44**–**47**) showed superior recovery activities in ATP production compared to **7**. Compounds without aromatic substituents (**28**–**29**, **37**–**43**) generally showed little to no effects on both assays except compounds **30** and **40** which has relatively bulky side chains. We think that the aromatic substituents are likely to act as a place holder or a steric blocker for specific interactions. Compound **27**, which only differs the positions of difluoro-substituents from compound **14**, did not show any effect on ATP recovery or MTT assays, suggesting that the difluoro-substituents might be involved in hydrogen-bonding or hydrogen-bonding induced conformational changes. Compounds with a constrained heterocycle connecting the R_2_ and R_3_ positions (**44**–**47**) appeared to restore ATP production significantly, while piperidinyl analogs (**46**, **47**) are more effective in recovering both ATP production and metabolic activity than piperazinyl analogs (**44**–**45**), again suggesting that hydrogen-bonding interactions might be involved in this binding region.

Next, we tested the second set of compounds, **48–57**, that were designed to have an *N*-methyl group replacing the acetamide group at the 7-position of the tetrahydropyrido moiety in **7**. As described in Table 4, compounds in this series showed slightly less of ΔΨ_m_ recovery, while demonstrated better results in ATP recovery and MTT assay, compared to their acetamide counterparts. We think that the replacement of the acetamide to the methyl may have affected electron-density of the nitrogen with little effect on overall hydrophobicity or conformation. The ATP production assay and the MTT assay results indicated that compounds in this group generally showed favourable recovery activity in both assays except compounds **53** and **54**.

The final group of compounds contained an alkyl chain in place of the 4-ethoxyphenyl side chain in compound **7** (compounds **58 − 62**). As shown in Table 5, compounds in this series demonstrated comparable recovery of ΔΨ_m_ (60–79%) likely due to their hydrophobicity; however, they generally showed moderate to poor recovery of ATP production (−7 to 23%) and cell viability (0–5%), suggesting that the additional lipophilic substituent may help sustain the membrane potential temporarily, but does not seem to affect overall mitochondrial function.

**Table 5.** *In vitro* assay results of compounds **58**–**62**. 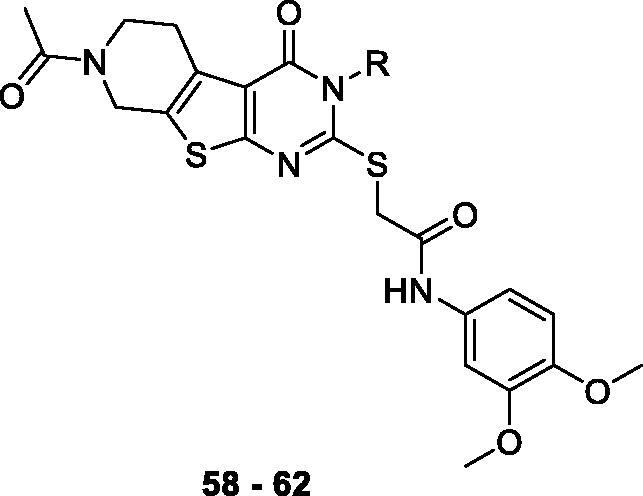

Compound	R	Recovery of ΔΨ_m_	ATP Recovery	MTT	
				Recovery	Viability
**58**	Ethyl	60%	23%	4%	99%
**59**	*n*-Propyl	79%	−1%	5%	112%
**60**	Isobutyl	66%	14%	1%	96%
**61**	*n*-Butyl	71%	−7%	0%	97%
**62**	Isopropyl	68%	15%	0%	94%
**7**		73%	42%	28%	95%

### 3.7. Assessment of drug-like properties of the selected compounds

On the basis of the *in vitro* mitochondrial functional assays, we selected a total number of 20 compounds with excellent recovery effects. We determined inhibition of CYP450 isozyme, inhibition of the hERG, and human liver microsomal stability to evaluate drug-like properties of the selected compounds (Table 6). Compounds **31**–**32**, **34**, **36**, **45**–**48, 52, 55**, and **56** showed relatively high inhibition (<75%) against at least one CYP450 isozyme. Compounds with bi-substituted *N*-alkyl chains (Table 3, **32, 33, 39, 40, 45–47, 56**) demonstrated poor stability in human liver microsome, showing less than 10% of the compound remaining intact after 30 min. Compounds **45–48** also demonstrated low water solubility, thus the IC_50_ values of the hERG inhibition could not be determined. Compounds **35** and **36** showed moderate hERG inhibition, implying a potential toxicity issue. Compounds **50** and **51** are similar in their structures, *in vitro* activity and drug-like properties, therefore we selected compound **50** as a representative compound. Based on this toxicity profile, we selected compounds **30**, **31**, **44**, **49** and **50**, which demonstrated low to moderate CYP inhibition against all tested isoforms, low hERG inhibition, with moderate to high liver microsomal stability, for further *in vivo* studies.

**Table 6.** CYP450, hERG liability, and human liver microsomal stability profiles of the selected compounds.

Compound	CYP450 (% remaining Activity at 10 μM)					hERG (IC_50_, μM)	Human Liver Microsomal Stability (% remaining after 30 min)
	CYP1A2	CYP2D6	CYP2C9	CYP3A4	CYP2C19		
**30**	119.7	118.5	93.0	31.0	107.6	12.30 ± 1.25	99.1
**31**	143.1	88.1	51.1	18.3	70.0	8.80 ± 2.36	32.7
**32**	117.3	91.7	30.9	49.3	23.3	6.53 ± 2.46	8.1
**33**	150.5	82.0	32.8	56.6	35.7	2.77 ± 1.18	9.6
**34**	160.6	92.6	32.9	15.0	56.8	4.13 ± 1.39	0.0
**35**	120.0	113.8	64.0	70.7	72.0	30.00 ± 10.2	0.1
**36**	123.6	98.9	50.4	17.3	92.7	22.00 ± 12.7	0.0
**39**	143.1	95.1	61.0	51.8	64.0	5.87 ± 1.18	0.0
**40**	103.0	79.8	33.6	38.9	57.4	2.43 ± 0.48	1.5
**44**	92.0	117.3	84.4	72.2	28.3	8.90 ± 0.22	15.3
**45**	110.5	107.0	14.9	89.1	23.2	ND	4.3
**46**	84.4	72.3	6.7	81.2	16.5	ND	6.6
**47**	81.5	64.0	15.6	66.2	15.0	ND	2.2
**48**	35.8	57.8	33.7	20.7	20.3	ND	63.4
**49**	95.3	43.1	99.6	28.1	90.0	15.00 ± 4.24	87.9
**50**	97.3	30.0	66.4	36.5	47.9	10.60 ± 0.97	73.1
**51**	98.7	61.4	105.8	44.1	52.5	10.00 ± 3.60	82.0
**52**	117.2	36.6	86.9	42.6	16.1	8.60 ± 0.83	67.6
**55**	69.2	59.2	43.0	23.0	31.2	5.63 ± 1.75	49.1
**56**	71.9	89.2	44.5	30.9	30.5	12.90 ± 3.03	1.3

ND: not determined (due to the low solubility).

### 3.8. *In vivo* activity of the selected compounds

**Figure 7.**
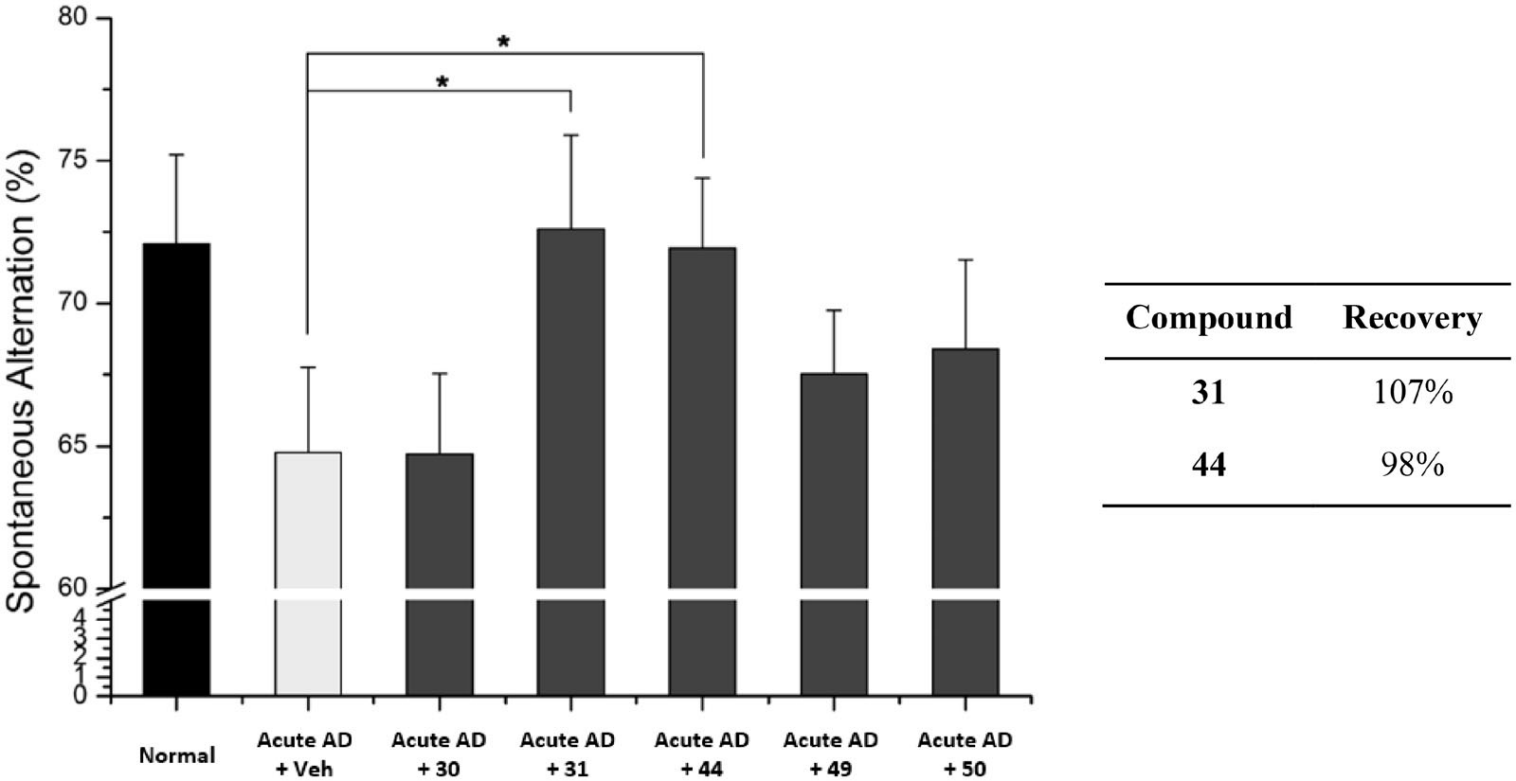
Recovery of Y-maze spontaneous alternations in acute AD model mice by treating each compound. Each group was evaluated by comparing with the piracetam-treated group (30 mg/kg daily, 6 days, i.p.). Data are expressed as a mean ± SEM (*n* = 7 per group): *p < 0.05.

To assess the *in vivo* activity of the selected compounds, **30**, **31**, **44**, **49** and **50**, we administered each compound (30 mg/kg, daily) to the acute AD model mice for 6 days. After the administration, we evaluated the cognitive function of the mice by performing the Y-maze spontaneous alternation test. As shown in Figure 7, compounds **31** and **44** significantly recovered learning and memory deficits in the acute AD model mice. Both compounds **31** and **44** improved Aβ-induced memory deficits almost to the level of normal mice, demonstrating the recovery percent of 107% and 98%, respectively. These percent values are greater than those of **7** (76%) and piracetam (20%) (Figure 4). In contrast, compounds **30**, **49**, and **50** did not show significant activity. We concluded that compounds **31** and **44** are the most active compounds based on their *in vitro* and *in vivo* protective effect against Aβ-induced mitochondrial dysfunction.

### 3.9. Pharmacokinetic properties of compounds 31 and 44

We evaluated the pharmacokinetic profiles of the two most active compounds **31** and **44** using SD male rats. As shown in Table 7, compound **31** showed low bioavailability (8.0%) whereas compound **44** demonstrated much higher bioavailability (78.4%). However, given its relatively low liver microsomal stability (15.3%) and short plasma half-life (80 min), compound **44** needs further optimisation for oral administration. It is notable that the presence of a constrained phenylpiperazine group at the amide bond region (R_3_ and R_4_) improved bioavailability while reduced liver microsomal stability compared to the initial hit compound **7** (Supplementary Tables S3 and S4).

**Table 7.** Pharmacokinetic parameters (mean ± SD) of compounds **31** and **44** after intravenous (*n* = 3) and oral (*n* = 3) administration (10 mg/kg) to SD male rats.^a^

	31		44	
	Intravenous	Oral	Intravenous	Oral
AUC_0–∞_ (mg min/mL)	330.66 ± 93.61	26.59 ± 22.32	1070.23 ± 114.39	614.11 ± 120.15
AUC_last_ (mg min/mL)	273.60 ± 64.45	11.67 ± 5.99	1058.16 ± 109.82	487.49 ± 87.29
Terminal half-life (min)	173.83 ± 32.38	448.10 ± 27.60	80.17 ± 9.34	199.34 ± 134.70
C_max_ (mg/mL)	–	0.04 ± 0.02	–	2.11 ± 0.91
T_max_ (min)	–	48 (30 ∼ 60)	–	72 (60 ∼ 120)
CL (mL/mim/kg)	32.53 ± 10.08	–	9.43 ± 1.05	–
V_ss_ (mL/kg)	7736.8 ± 1114.3	–	785.49 ± 39.64	–
*F* (%)	8.0		78.4	

^a^AUC_0–∞_, total area under the plasma concentration–time curve from time zero to time infinity; AUC_last_, total area under the plasma concentration–time curve from time zero to last measured time; *C*_max_, peak plasma concentration; *T*_max_, time to reach *C*_max_; CL, time-averaged total body clearance; *V*_ss_, apparent volume of distribution at steady state; *F*, bioavailability.

### 3.10. TSPO binding affinity measured by SPR

The direct binding affinities of the initial hit compounds **7** and **14** as well as compounds **31** and **44** were determined against recombinant human TSPO by performing surface plasmon resonance (SPR) studies. As shown in Table 8 and Supplementary Figure S2, all four compounds demonstrated specific binding in a dose-dependent manner, having the *K*_D_ values in a low micromolar range. Although **PK11195**, a well-known TSPO ligand, seemed to be much more potent (*K*_D_ = 1.1 ± 0.45 nM) than our newly identified ligands, given the size and structural differences, we believe that these compounds can serve as a good lead compound with a novel scaffold for future optimisation through SAR studies. These results supported that these compounds specifically interact with TSPO to exert their biological activity.

**Table 8.** TSPO binding affinity.^a^

Compound	*K*_D_
**7**	0.63 ± 0.33 μM
**14**	1.69 ± 0.40 μM
**31**	1.25 ± 0.40 μM
**44**	2.74 ± 0.66 μM
**PK11195**	1.1 ± 0.45 nM

^a^The *K*_D_ values are expressed as the mean ± SD from three independent measurements.

### 3.11. Docking study

**Figure 8.**
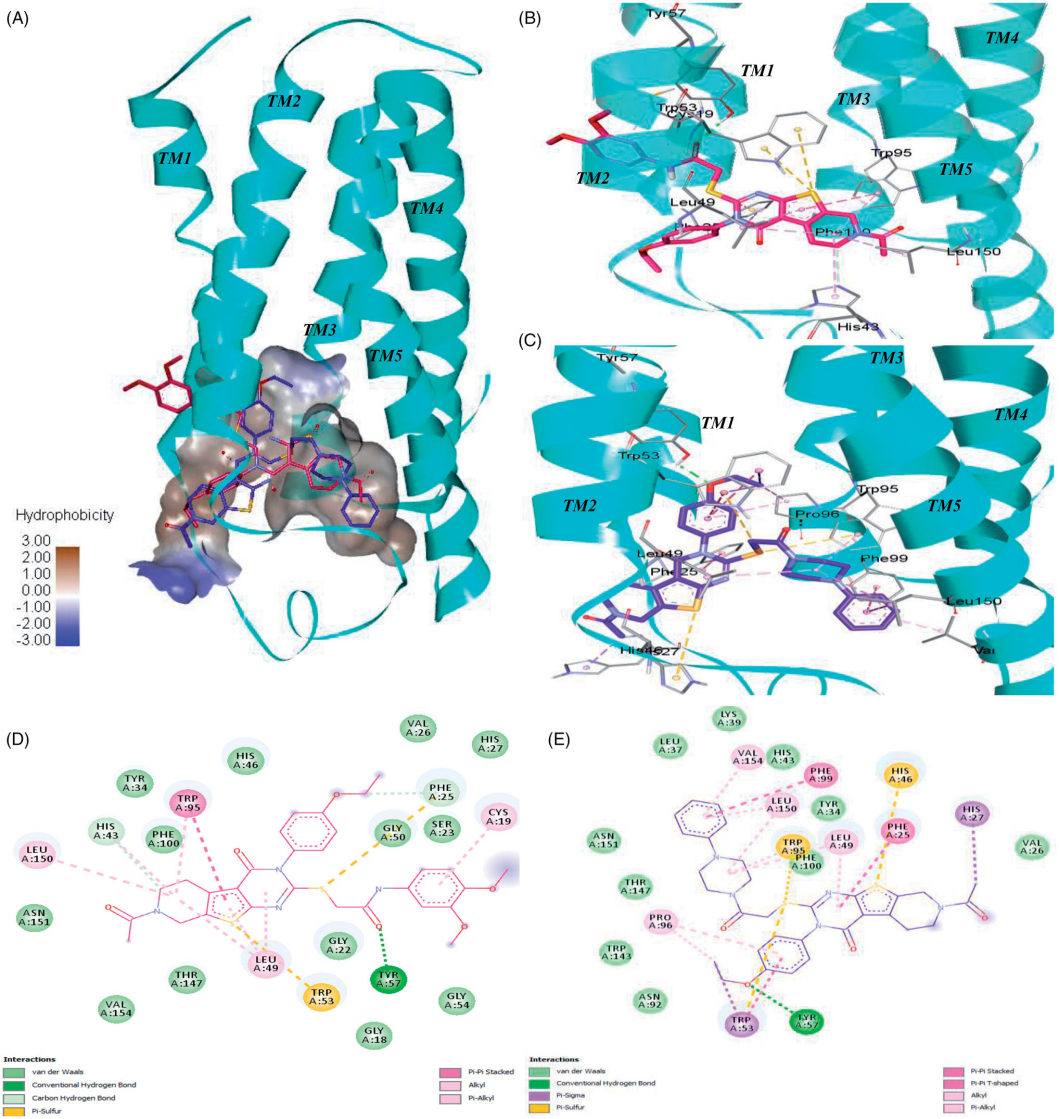
Interactions of **7** and compound **44** with the human TSPO homology model. (A) Surface depiction of the TSPO binding site with the binding orientation overlapped with **7** (red stick) and compound **44** (blue stick). Detailed view of the interactions between **7** (B) and compound **44** (C) with the human TSPO. TSPO is represented with cyan helices and the interacting amino acids are depicted with grey sticks. Corresponding 2D interactions of **7** (D) and compound **44** (E).

To further investigate the binding mode of the selected compounds, we performed docking studies for the two most active compounds **7** and **44**. We generated the human TSPO homology model based on 4UC1 x-ray crystal structure as a template. The binding orientation of compounds **7** and **44** appeared to overlap inside the central pocket of the TSPO model as shown in Figure 8(A). The amide group of **7** (glide XP score= −11.27) formed a hydrogen bond with Tyr57 from TM2 as shown in Figure 8(B). The dimethoxy phenyl ring inserted between TM1 and TM2 showed a pi-alkyl bond with Cys19 from TM1. The tricyclic fused core ring compactly packed inside the binding pocket via strong pi stacked interactions of Trp95 from TM3 and pi-alkyl interactions of Leu49, Leu150, and His43. Two Pi-sulphur bonds were also observed between Trp53 and the sulphur atom of the thienopiperidinone ring. The sulphur atom from the alkyl chain also exhibited pi-sulphur interactions with Phe25 as shown in Figure 8(D).

In contrast, compound **44** (glide XP score= −11.20) fitted in reverse orientation inside the binding cavity (Figure 8(A)) which explains slightly lower binding affinity of **44** than **7**. Because of the presence of a bulky phenylpiperazine group, the tricyclic core ring of compound **44** moved towards the space between intracellular loop1 residues such as Phe25, His46, and Leu49 interacting via strong pi stacked, pi-sulphur and pi-alkyl contacts respectively (Figure 8(C)). The phenylpiperazine side chain is pointed towards TM5 while interacts with Phe99 from TM3 via a strong pi-stacked interaction. The presence of pi-stacking interactions corroborates the superior activity observed from compounds with an aromatic ring compared to compounds with no aromatic side chains. Several pi-alkyl interactions with Val154, Leu150, Leu49, and Trp95 were also observed. The sulphur atom from the alkyl chain showed two pi-sulphur bonds with Trp53 and Trp95. The oxygen atom of the ethoxy group formed a hydrogen bond with Tyr57. Trp53 showed pi stacked and pi sigma contacts with the ethoxy phenyl ring as described in Figure 8(E).

Overall, **7** and **44** have a similar scaffold and share the same binding cavity. In addition, both molecules appeared to form a hydrogen bond with Tyr57 residue. Docking score reveals that they have the equal binding potential with TSPO protein, but they may bind in the reverse orientation. The binding region between TM2, TM3, and TM5 is hydrophobic due to the presence of residues like Leu49, Trp95, Phe99, Leu150, and Val154. This hydrophobic region is preferable for binding of the phenylpiperazine group. Therefore, in spite of having similar scaffolds **7** and **44** demonstrate an opposite binding orientation in the docking study.

## 4. Conclusion

In this study, we synthesized a series of TSPO ligands derived from a ligand-based pharmacophore. We evaluated their *in vitro* and *in vivo* activity of mitochondrial functional recovery and discovered two initial hit compounds from virtual screening and *in vitro* assays, and further synthesised a library of analogs. Two most active compounds **31** and **44** demonstrated excellent recovery of ΔΨ_m_, ATP production, and cell viability upon Aβ-induced cytotoxicity. These compounds also demonstrated neuroprotective effects in AD model mice, improving learning and memory impairment. Our SPR data and the docking study results indicated that **7** and **44** directly interact with the TSPO binding site, while they may occupy the cavity in reverse orientation to each other despite their structural similarities. Taken together, our findings suggest that these compounds exert neuroprotective effects *in vitro* and *in vivo* by restoring mitochondrial function through the specific interaction with TSPO in the presence of Aβ. We believe that these novel TSPO ligands can serve as a promising lead for further therapeutic development, and may provide an alternative strategy to the current treatment options for AD.

## Supplementary Material

Supplemental Material
