# Supplementary material for: The translocator protein ligands as mitochondrial functional modulators for the potential anti-Alzheimer agents
Source: J Enzyme Inhib Med Chem. 2021 Mar 23;36(1):831–46. doi: 10.1080/14756366.2021.1900158 (PMC7996082; doi:10.1080/14756366.2021.1900158)
Supplement: Supplemental Material [file IENZ_A_1900158_SM9817.pdf]

Supplemental Material

The Translocator Protein Ligands as Mitochondrial Functional Modulators for the Potential Anti-Alzheimer Agents

TaeHun Kim,[‡,a,b] Mohammad Neaz Morshed,[‡ a,b,c] Ashwini M. Londhe,[‡a,b] Ji Woong Lim, [‡a,d] Ha Eun Lee, [a,b] Suengmok Cho,[e] Sung Jin Cho,[f] Hayoung Hwang,[f] Sang Min Lim,[a,b] Jae Yeol Lee, [a,d] Jiyoun Lee[*],[g] and Ae Nim Pae[,a,b,d]*

CONTENTS

- **Table S1-S4.** *In vitro* and *in vivo* assay results for virtual screening library
- **Figure S1**. SPR measurements of 7,14,31, and 44
- **Figure S2-S118**. ^{1}H NMR and ^{13}C NMR spectra of all compounds
- Detailed synthetic procedures and additional characterization data

Table S1. The JC-1 assay results of the selected 56 compounds (**VH001-VH056**).

Compd	Structure	Recovery of $\Delta\Psi_m$ at 5 μM[a]	Compd	Structure	Recovery of $\Delta\Psi_m$ at 5 μM[a]
**VH001**	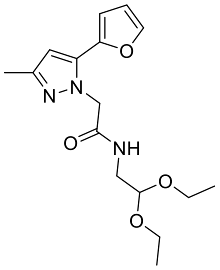	-11%	**VH002**	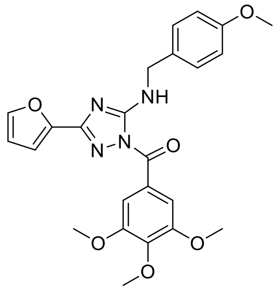	11%
**VH003**	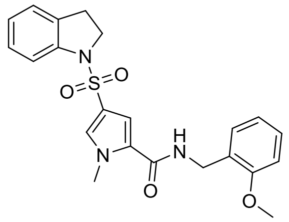	52%	**VH004**	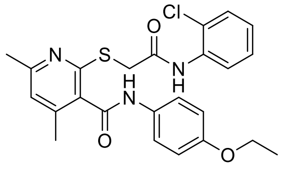	24%
**VH005**	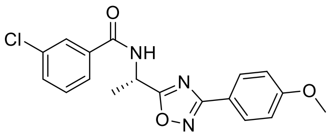	13%	**VH006**	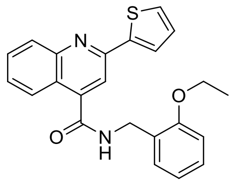	49%
**VH007**	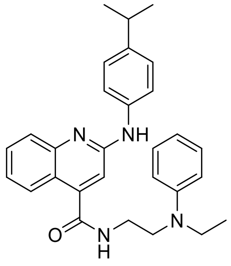	30%	**VH008**	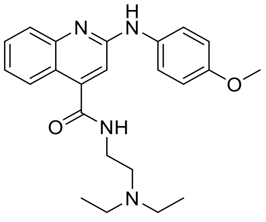	58%
**VH009**	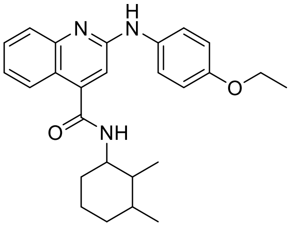	47%	**VH010**	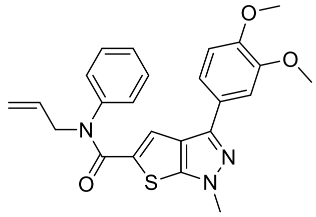	32%

Compd	Structure	Recovery of $\Delta\Psi_m$ at 5 μM[a]	Compd	Structure	Recovery of $\Delta\Psi_m$ at 5 μM[a]
**VH011**	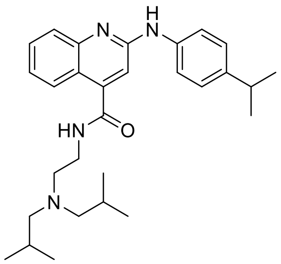	47%	**VH012**	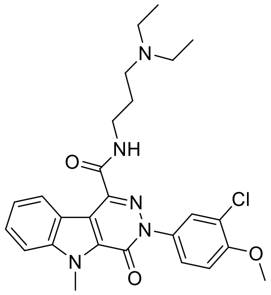	61%
**VH013**	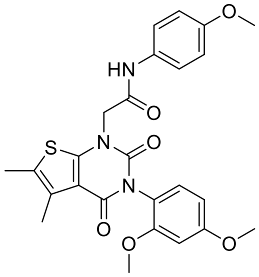	-5%	**VH014**	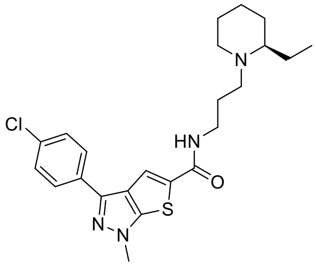	44%
**VH015**	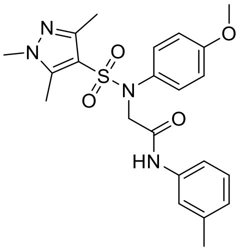	13%	**VH016**	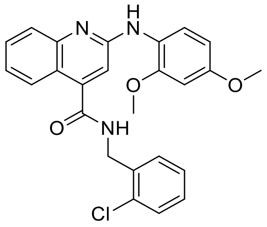	59%
**VH017**	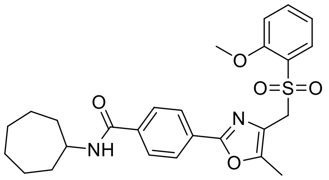	18%	**VH018**	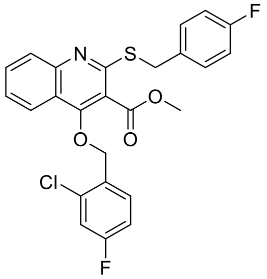	-93%
**VH019**	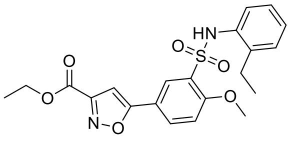	-45%	**VH020**	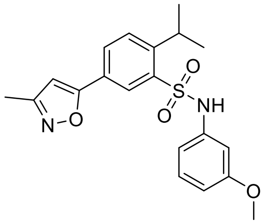	27%

**Compd**	Structure	Recovery of $\Delta\Psi_m$ at 5 μM[a]	Compd	Structure	Recovery of $\Delta\Psi_m$ at 5 μM[a]
**VH021**	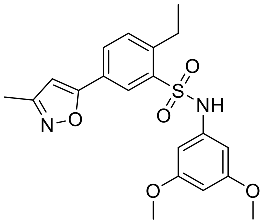	42%	**VH022**	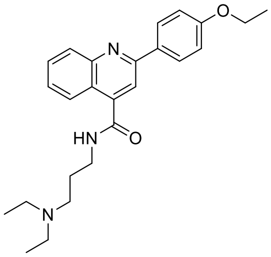	36%
**VH023**	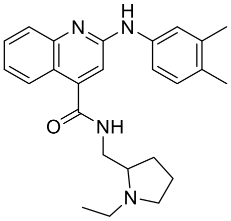	62%	**VH024**	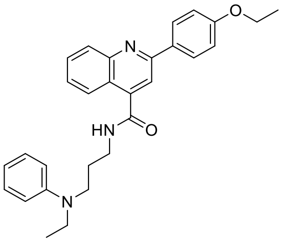	74%
**VH025**	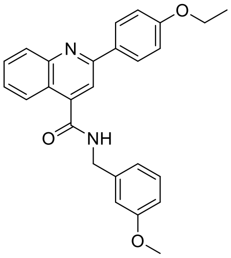	12%	**VH026**	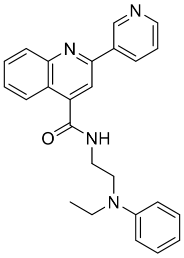	49%
**VH027**	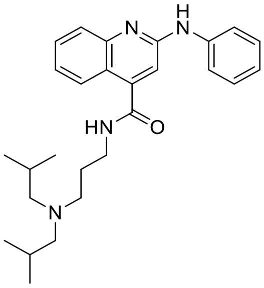	25%	**VH028**	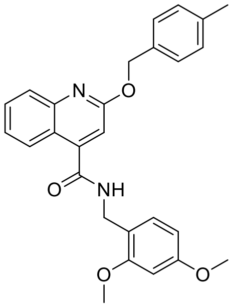	51%
**VH029**	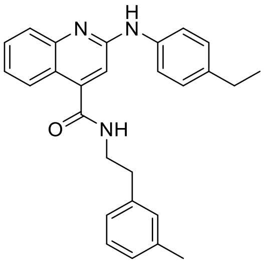	18%	**VH030**	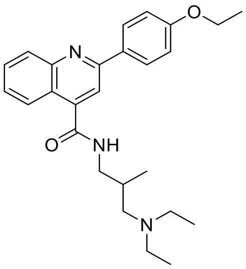	60%

**Compd**	**Structure**	Recovery of $\Delta\Psi_m$ at 5 μM[a]	**Compd**	**Structure**	Recovery of $\Delta\Psi_m$ at 5 μM[a]
**VH031**	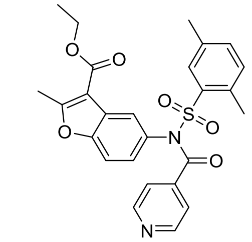	40%	**VH032**	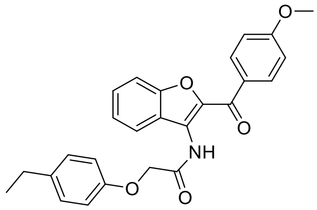	27%
**VH033**	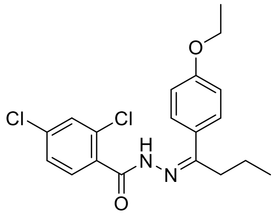	-30%	**7** (**VH034**)	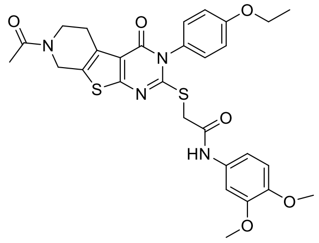	78%
**VH035**	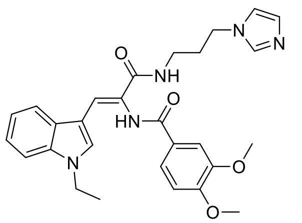	17%	**VH036**	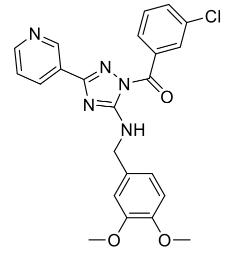	36%
**VH037**	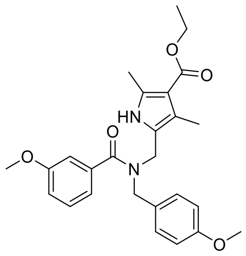	44%	**VH038**	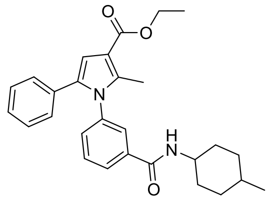	46%
**VH039**	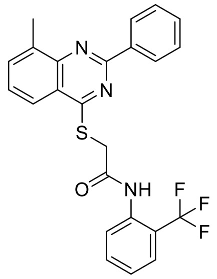	-86%	**VH040**	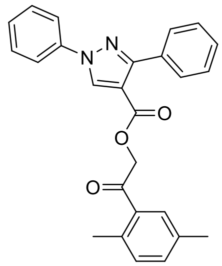	81%

Compd	Structure	Recovery of $\Delta\Psi_m$ at 5 μM[a]	Compd	Structure	Recovery of $\Delta\Psi_m$ at 5 μM[a]
**VH041**	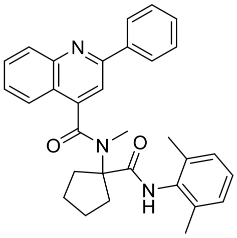	87%	**VH042**	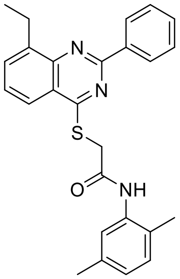	-44%
**VH043**	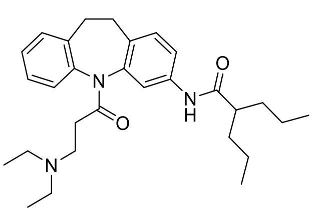	69%	**VH044**	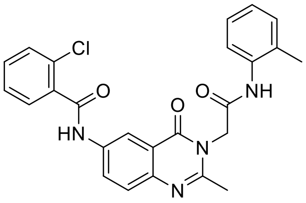	64%
**VH045**	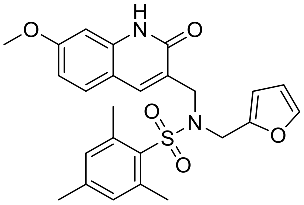	78%	**VH046**	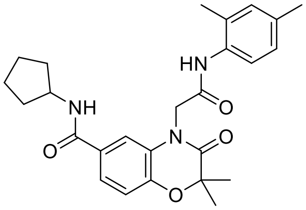	-27%
**VH047**	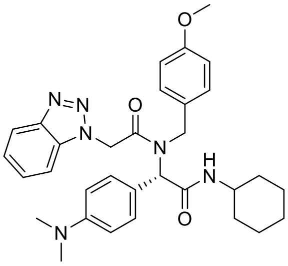	68%	**VH048**	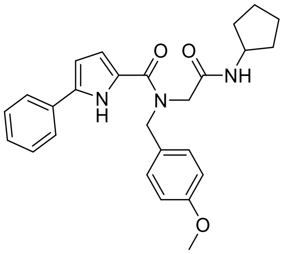	47%
**VH049**	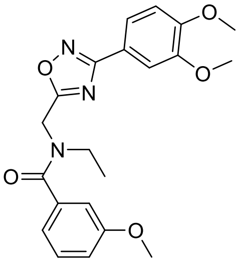	18%	**8** (**VH050**)	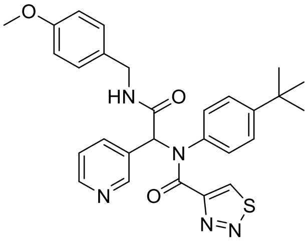	92%

Compd	Structure	Recovery of $\Delta\Psi_m$ at 5 μM[a]	Compd	Structure	Recovery of $\Delta\Psi_m$ at 5 μM[a] [a]
**VH051**	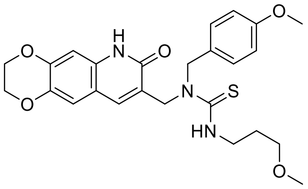	-37%	**VH052**	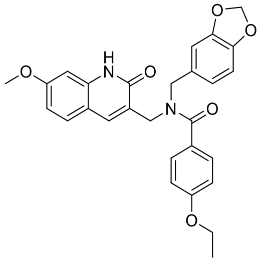	50%
**VH053**	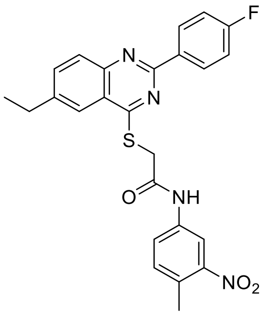	78%	**VH054**	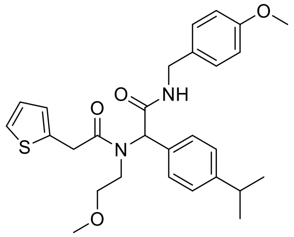	-22%
**VH055**	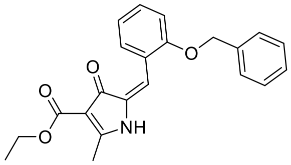	22%	**VH056**	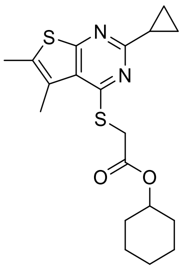	2%
	**Piracetam**	60%		**CsA**	55%

[a] After the treatment of each compound (5 μM) and Aβ (5 μM) in HT22 cell, the increase of fluorescence ratio (green/red) was measured and normalized by taking the change of the ratio between 0% (Aβ-induced damaged condition) and 100% (normal condition in the absence of Aβ).

Table S2. *In vitro* ATP production recovery activities of the 23 compounds selected from the JC-1 assay.

Compd	ATP recovery (%) [a]	Compd	ATP recovery (%) [a]
**VH003**	28%	**VH008**	-2%
**VH009**	-62%	**VH011**	22%
**VH012**	-17%	**VH016**	-18%
**VH023**	20%	**VH024**	13%
**VH026**	-6%	**VH028**	28%
**VH030**	-14%	**7** (**VH034**)	42%
**VH038**	33%	**VH040**	-159%
**VH041**	-11%	**VH043**	0%
**VH044**	-66%	**VH045**	-53%
**VH047**	-68%	**VH048**	0%
**8** (**VH050**)	45%	**VH052**	0%
**VH053**	16%		
**CsA**	- 46%	**Piracetam**	127%

[a] Recovery of ATP production at a concentration of 5 μM of each test compound against Aβ-induced mitochondrial ATP reduction.

Table S3. Mean (± SD) pharmacokinetic parameters[a] after intravenous (n = 5) and oral (n = 5) administration (10 mg/kg) of **7** and **14** to SD male rats.

	**7**		**14**	
	**Intravenous**	**Oral**	**Intravenous**	**Oral**
$AUC_{0–\infty}$ (mg min/ml)	361.71±88.82	135.03±15.37	330.66±93.61	26.59±22.32
AUC_{last} (mg min/ml)	360.07±88.83	115.57±28.01	273.60±64.45	11.67±5.99
Terminal half-life (min)	62.49±4.71	140.77±62.19	173.83±32.38	448.10±27.60
C_{max} (mg/ml)	-	0.43±0.22	-	0.04±0.02
T_{max} (min)	-	132 (60~240)	-	48 (30~60)
CL (ml/mim/kg)	28.95±7.09	-	32.525±10.08	-
V_{ss} (ml/kg)	1737.04±498.80	-	7736.86±1114.29	-
F (%)	37.3		8.0	

[a]$AUC_{0–\infty}$, total area under the plasma concentration–time curve from time zero to time infinity; AUC_{last}, total area under the plasma concentration–time curve from time zero to last measured time; C_{max}, peak plasma concentration; T_{max}, time to reach C_{max}; CL, time-averaged total body clearance; MRT, mean residence time; V_{ss}, apparent volume of distribution at steady state; *A*e, Excreted amount; *F*, bioavailability.

Table S4. CYP450, hERG liability, and human microsomal stability profiles of **7** and **14**.

**Compd**	**CYP450 (% remaining Activity @ 10 μM)**				**hERG (IC_{50}, μM)[a]**	**Microsomal stability (% remaining after 30 min)**
	CYP1A2	CYP2D6	CYP2C9	CYP3A4		
**7**	69.0	83.3	72.5	69.9	19.5 ± 4.43	49.6
**14**	92.0	98.9	63.8	158.6	4.23 ± 0.48	62.9

[a] IC_{50} values (±SD) were obtained from a dose-response curve.

Figure S1. Representative surface plasmon resonance (SPR) sensorgrams of **7 (VH034)** (**A**), **14 (VH062)** (**B**), compound **31** (**C**) and compound **44** (**D**). The K_D values are expressed as mean ± SD from three independent measurements.

A

7 (VH034), $K_D = 0.63 \pm 0.33$ µM

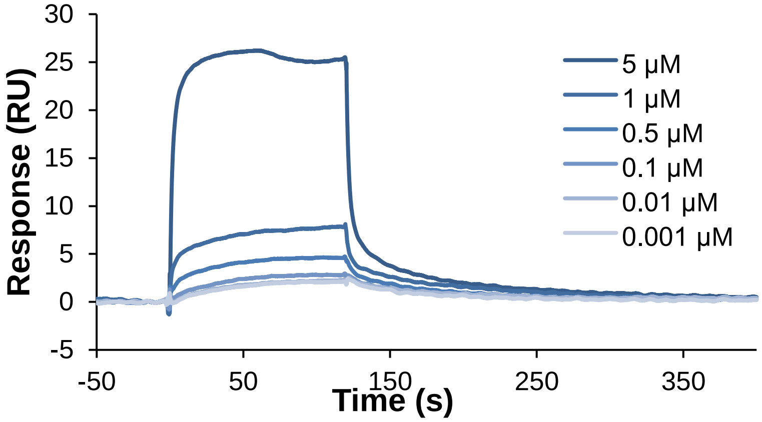

B

14 (VH062), $K_D = 1.69 \pm 0.40$ µM

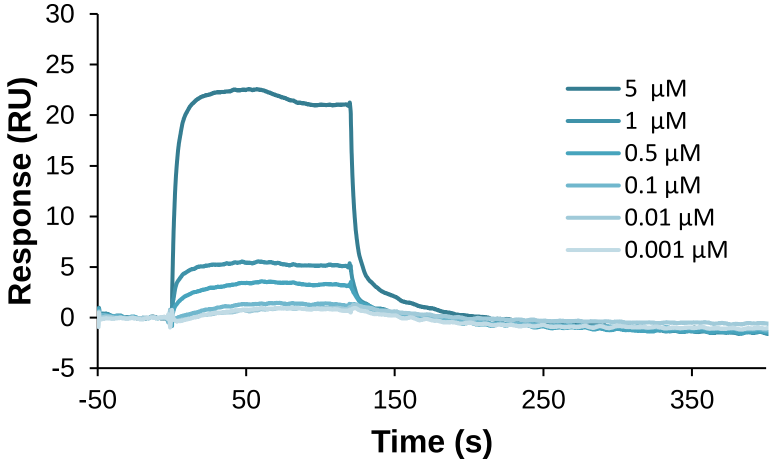

C

Compound **31**, $K_D = 1.25 \pm 0.79$ μM

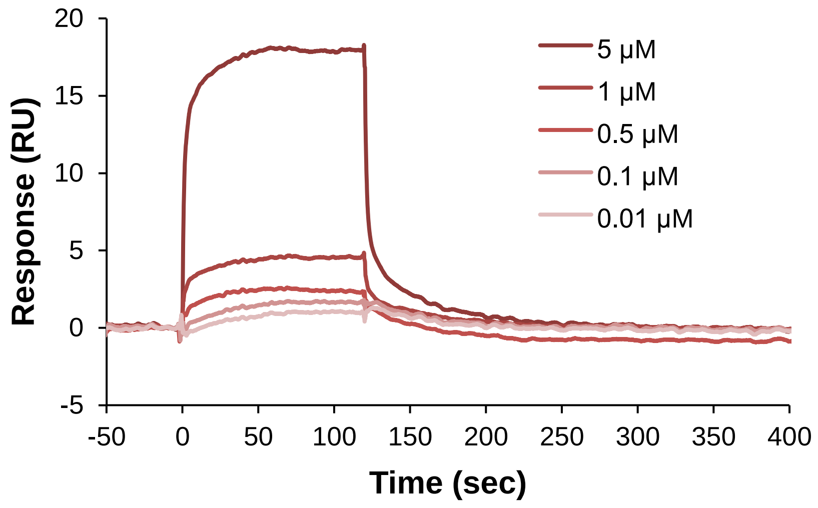

D

Compound **44**, $K_D = 2.74 \pm 0.66$ μM

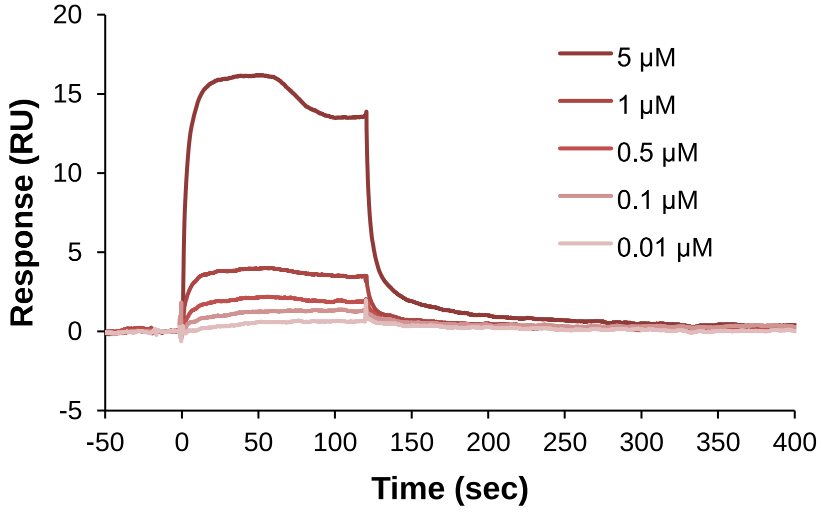

Figure S2. ^{1}H NMR Spectrum of **23a** in $CDCl_3$

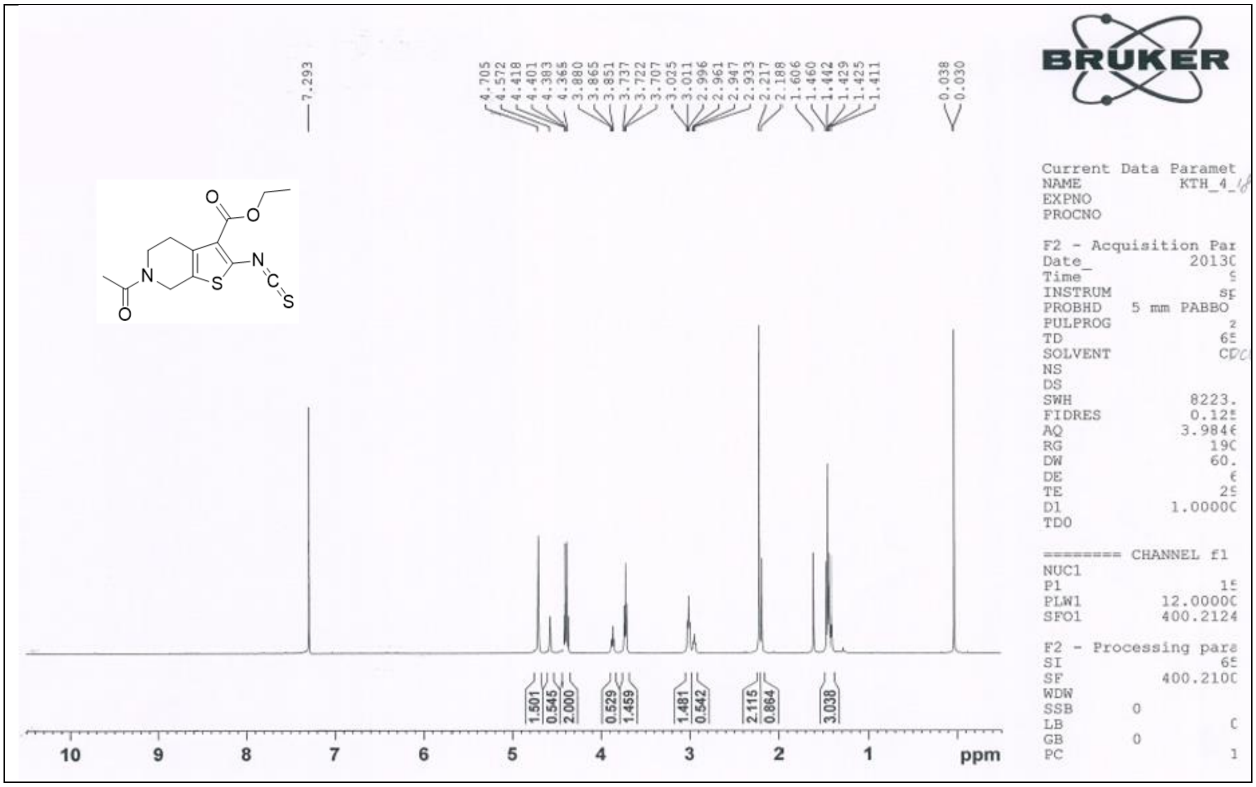

Figure S3. ^{13}C NMR Spectrum of **23a** in $CDCl_3$

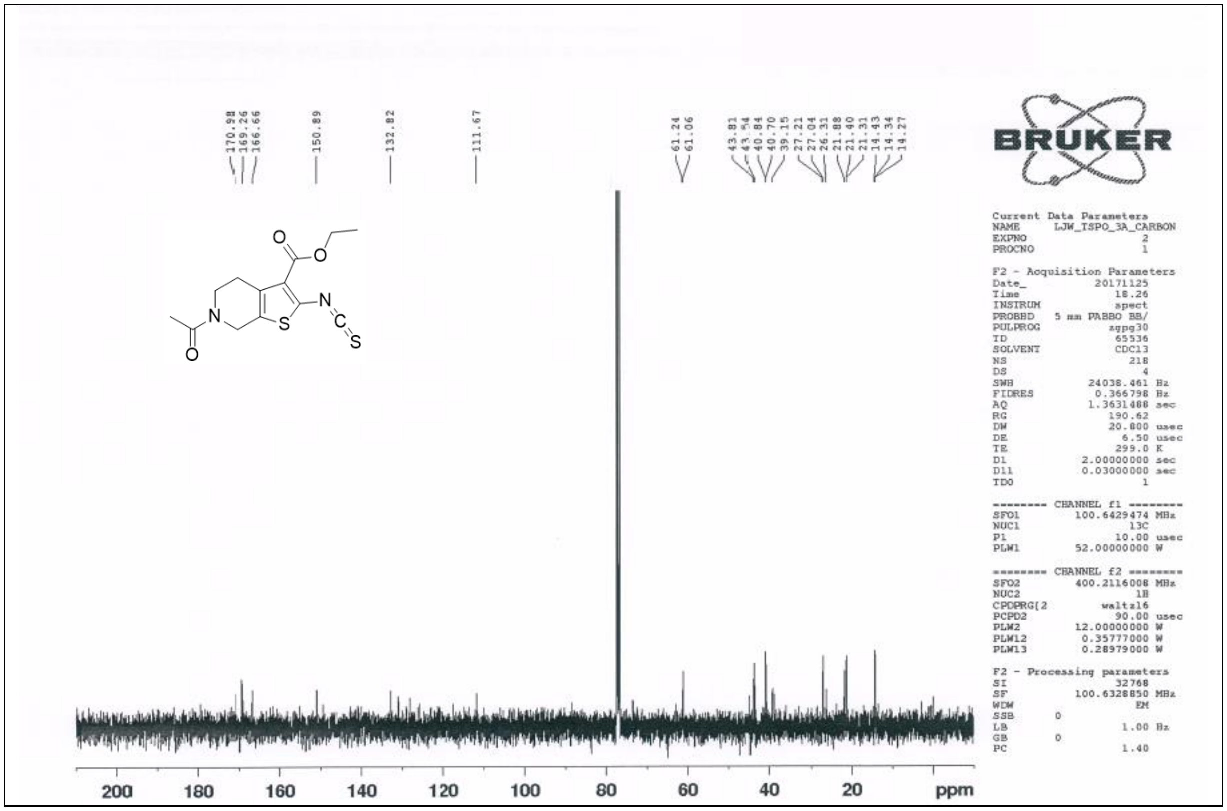

Figure S4. ^{1}H NMR Spectrum of **23b** in $CDCl_3$

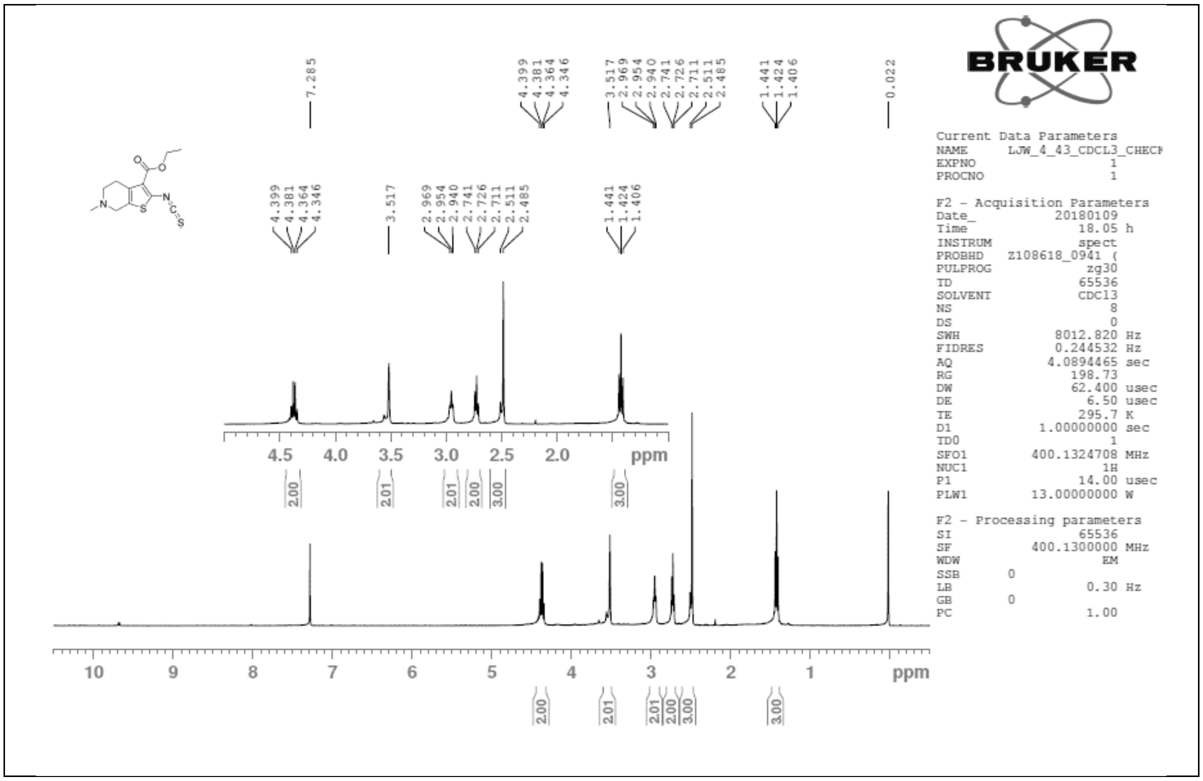

Figure S5. ^{13}C NMR Spectrum of **23b** in $CDCl_3$

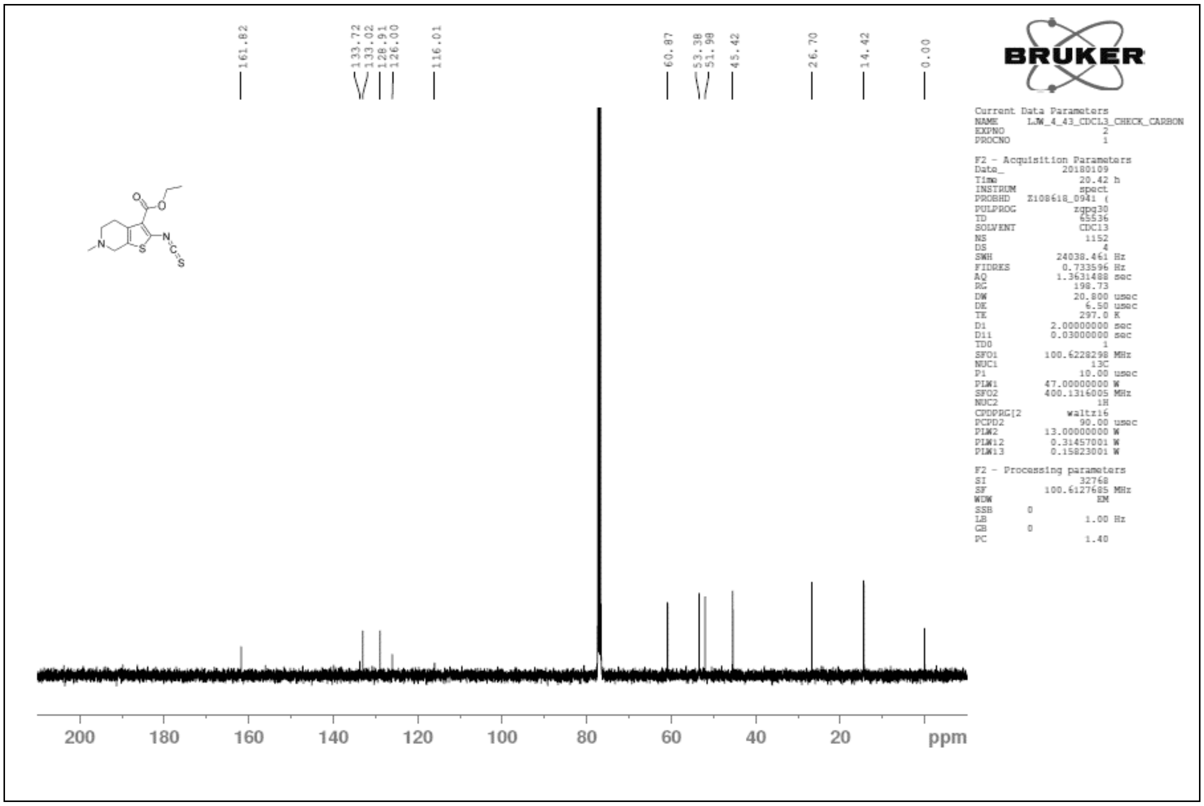

Figure S6. ^{1}H NMR Spectrum of **24aa** in $CDCl_3$

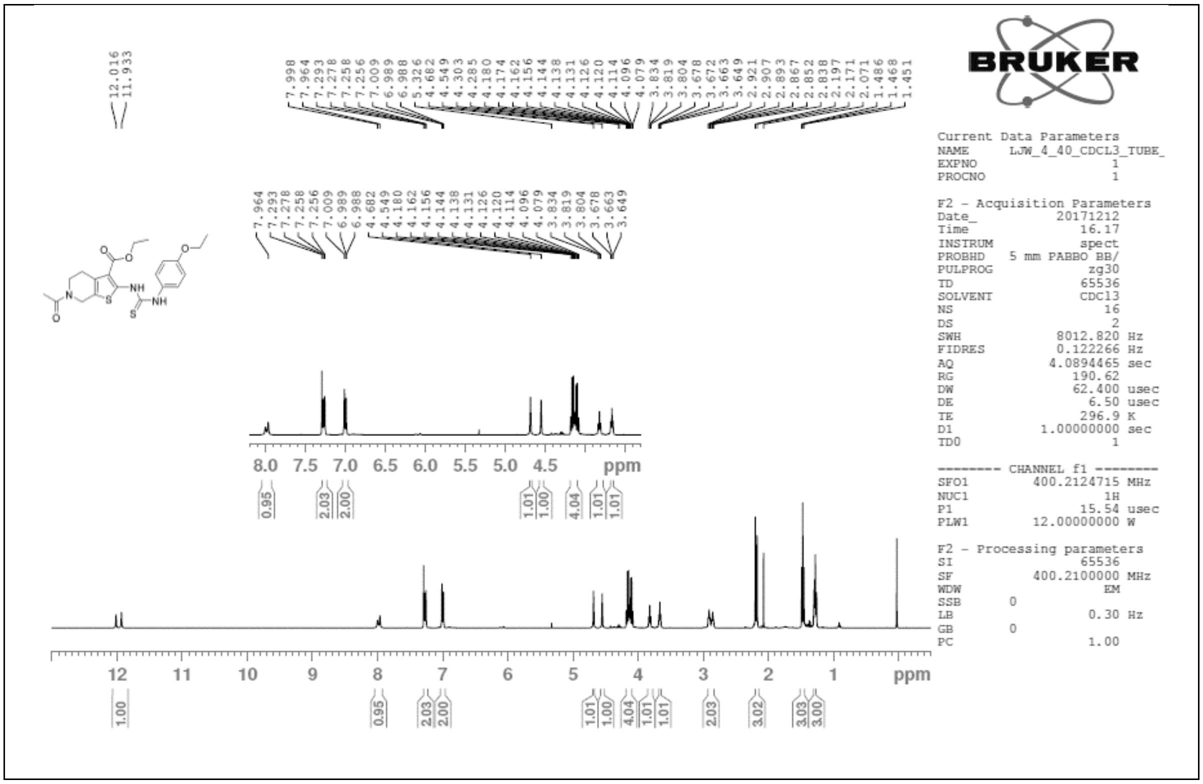

Figure S7. ^{13}C NMR Spectrum of **24aa** in $CDCl_3$

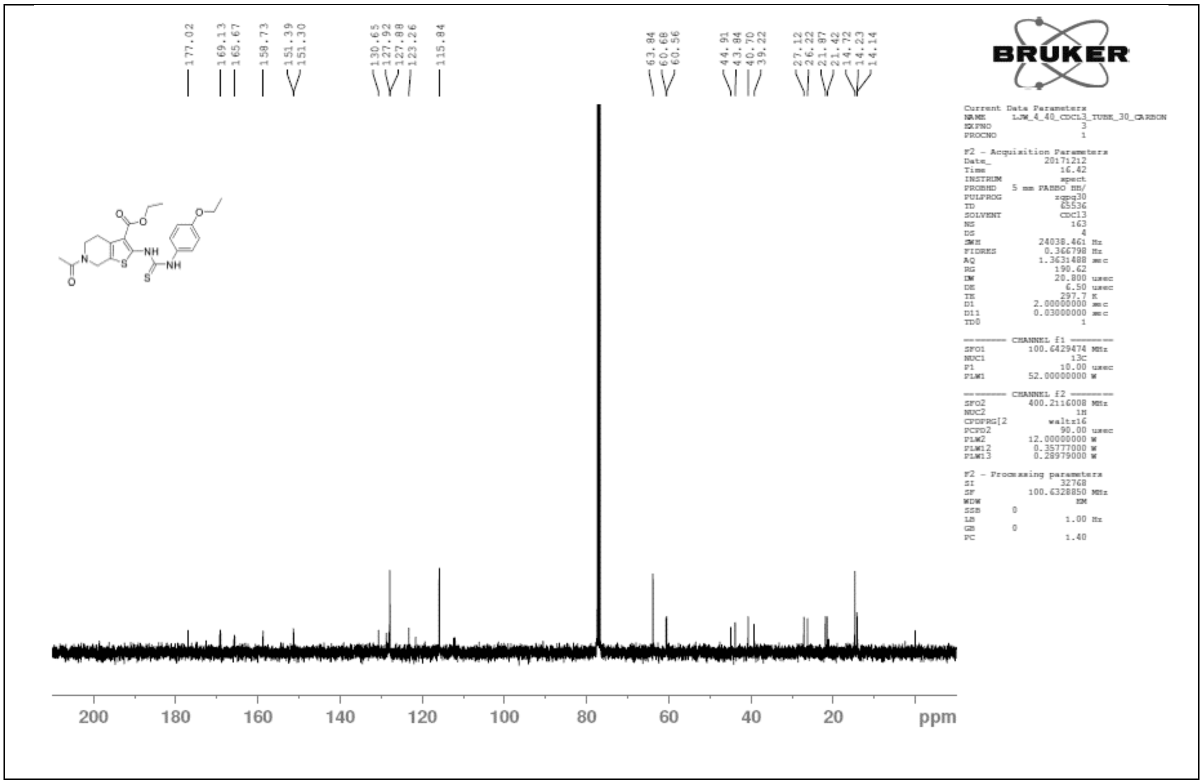

Figure S8. ^{1}H NMR Spectrum of **24ab** in $CDCl_3$

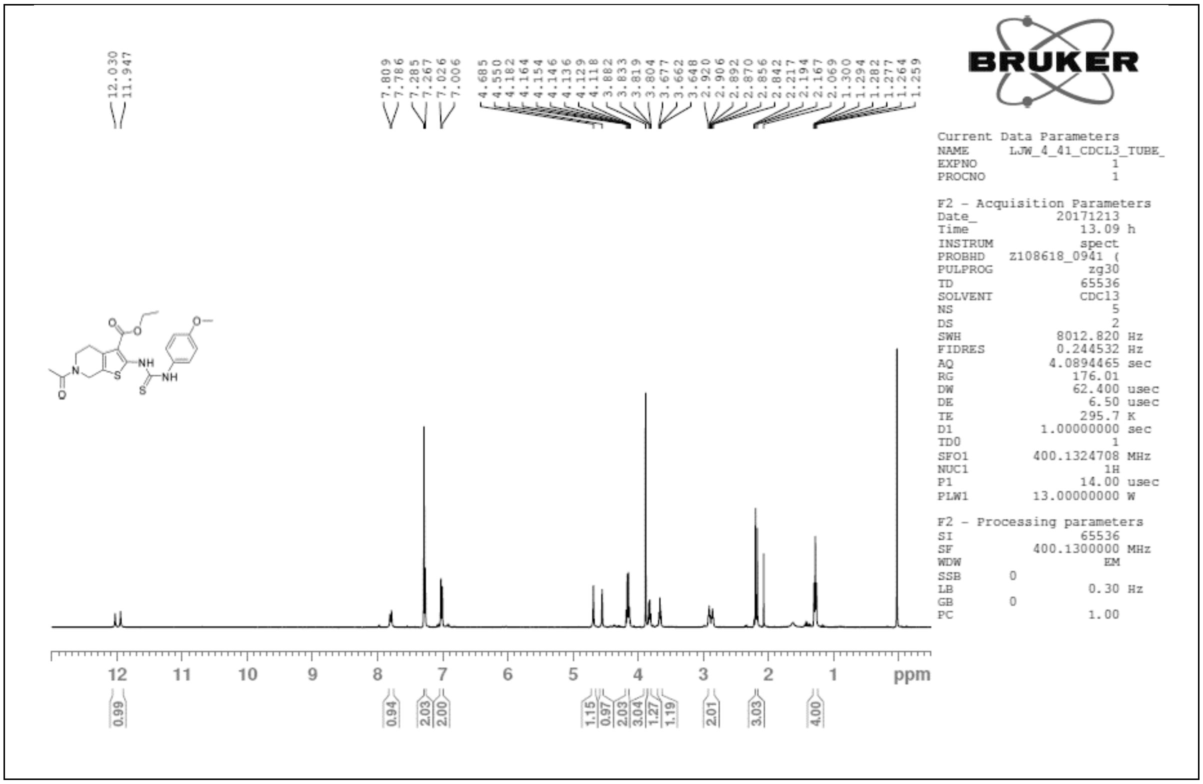

Figure S9. ^{13}C NMR Spectrum of **24ab** in $CDCl_3$

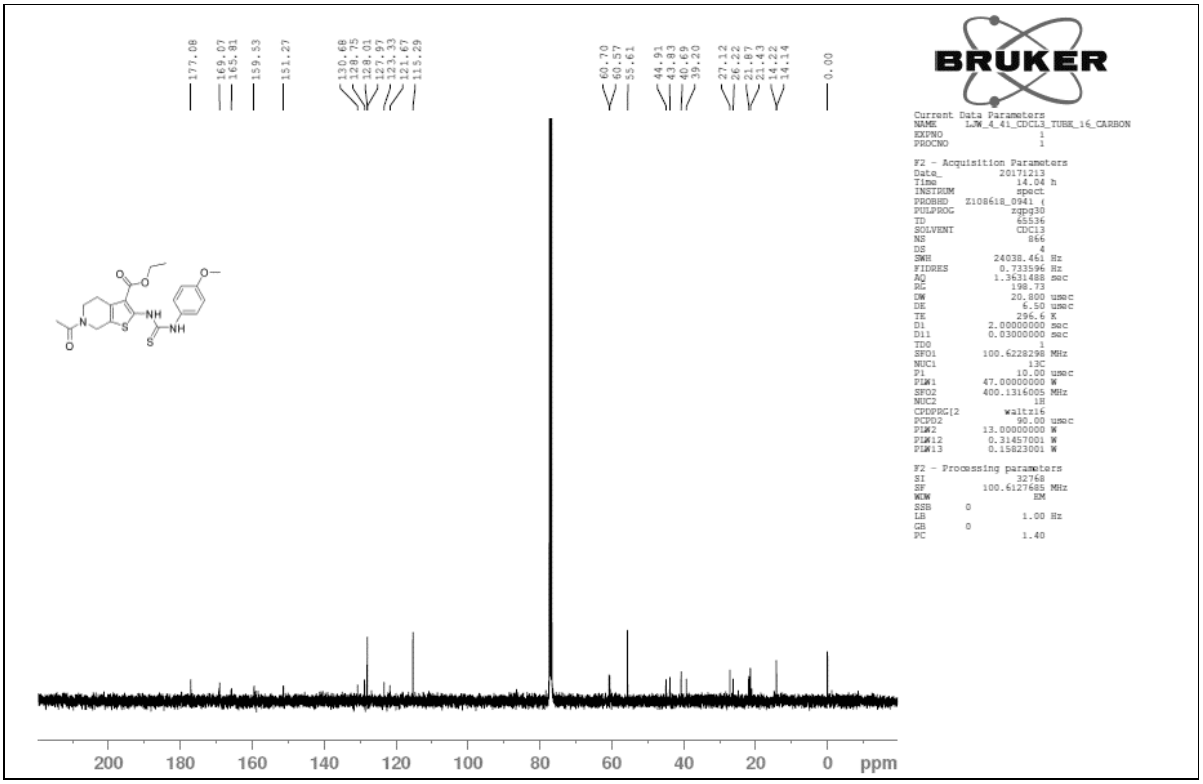

Figure S10. ^{1}H NMR Spectrum of **24c** in $CDCl_3$

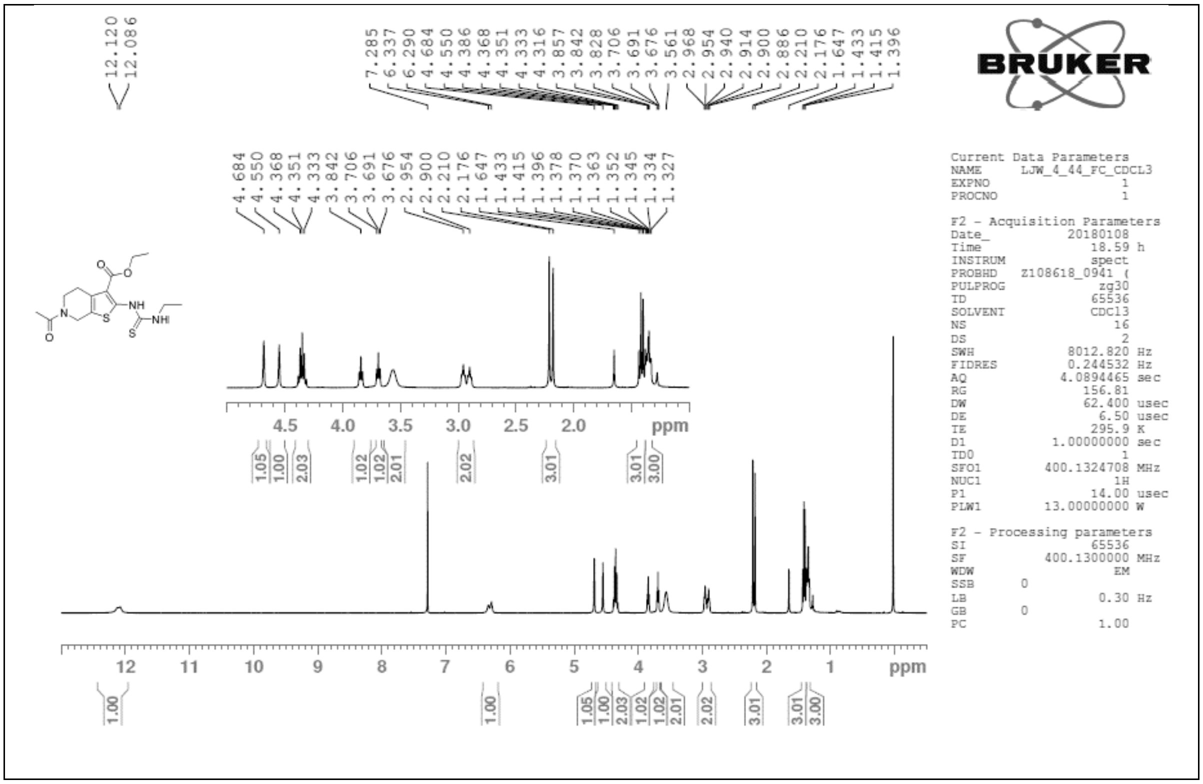

Figure S11. ^{13}C NMR Spectrum of **24c** in $CDCl_3$

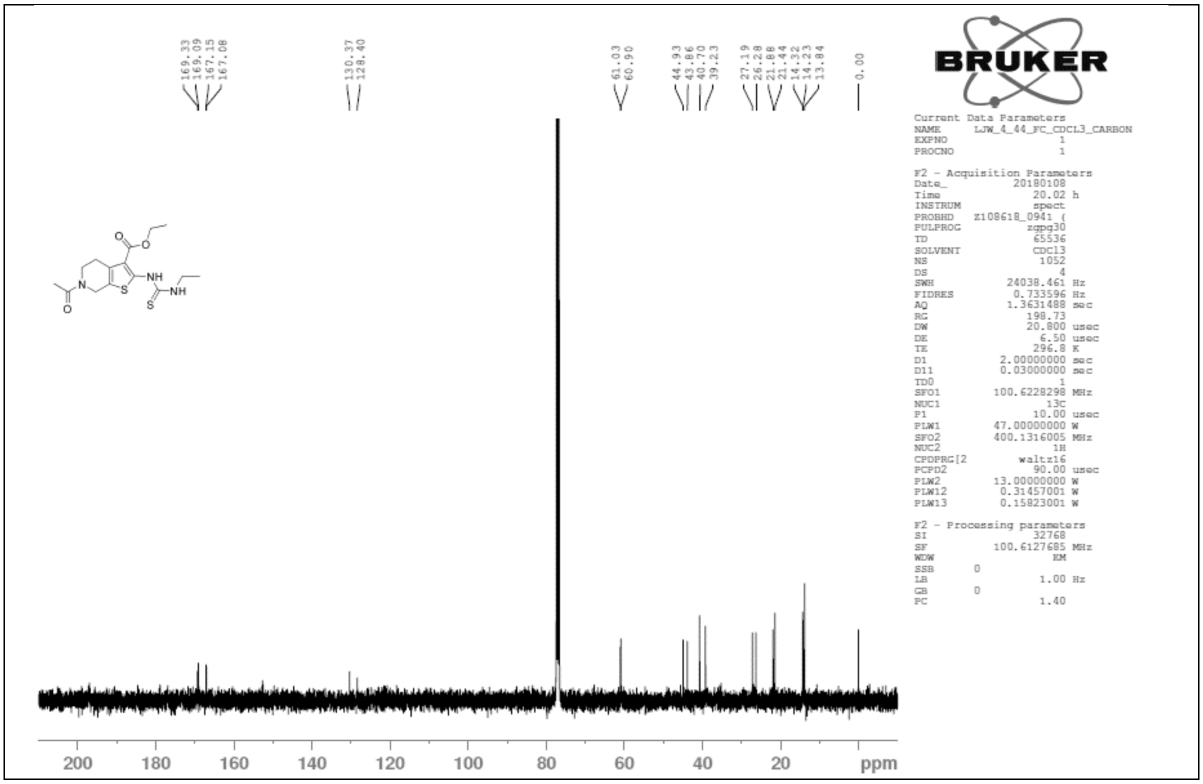

Figure S12. ^{1}H NMR Spectrum of **24d** in $CDCl_3$

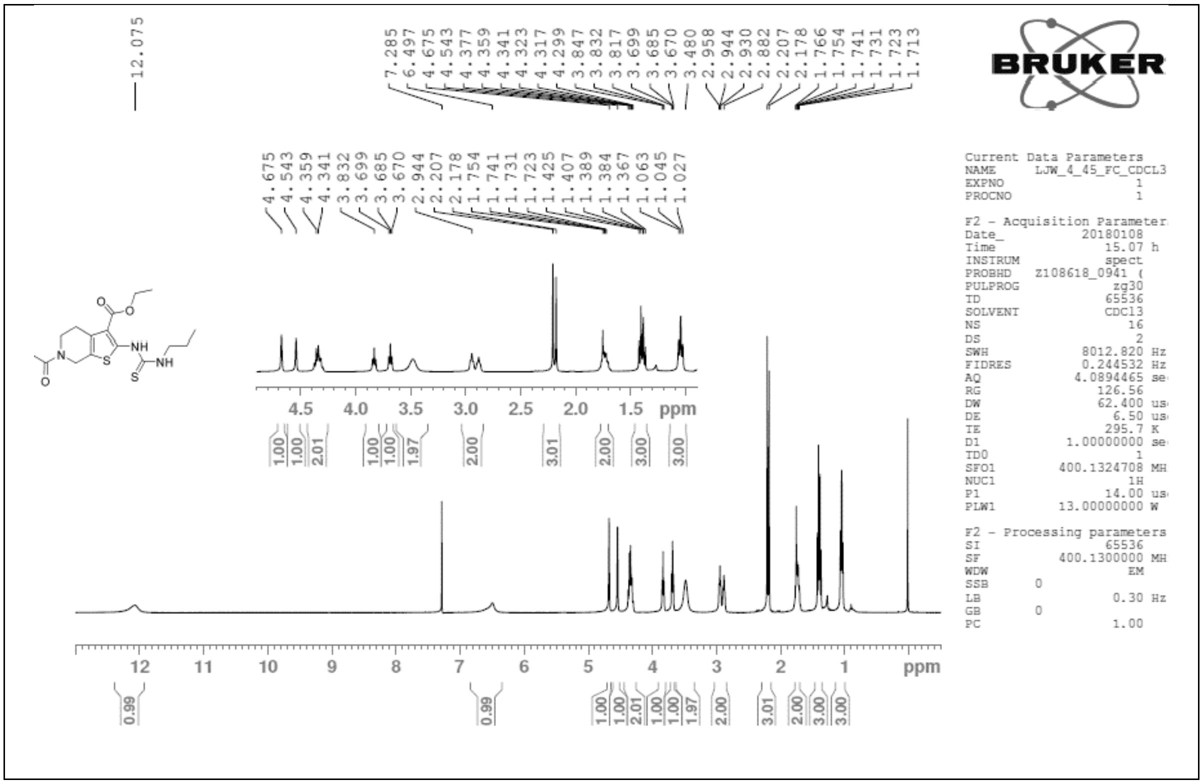

Figure S13. ^{13}C NMR Spectrum of **24d** in $CDCl_3$

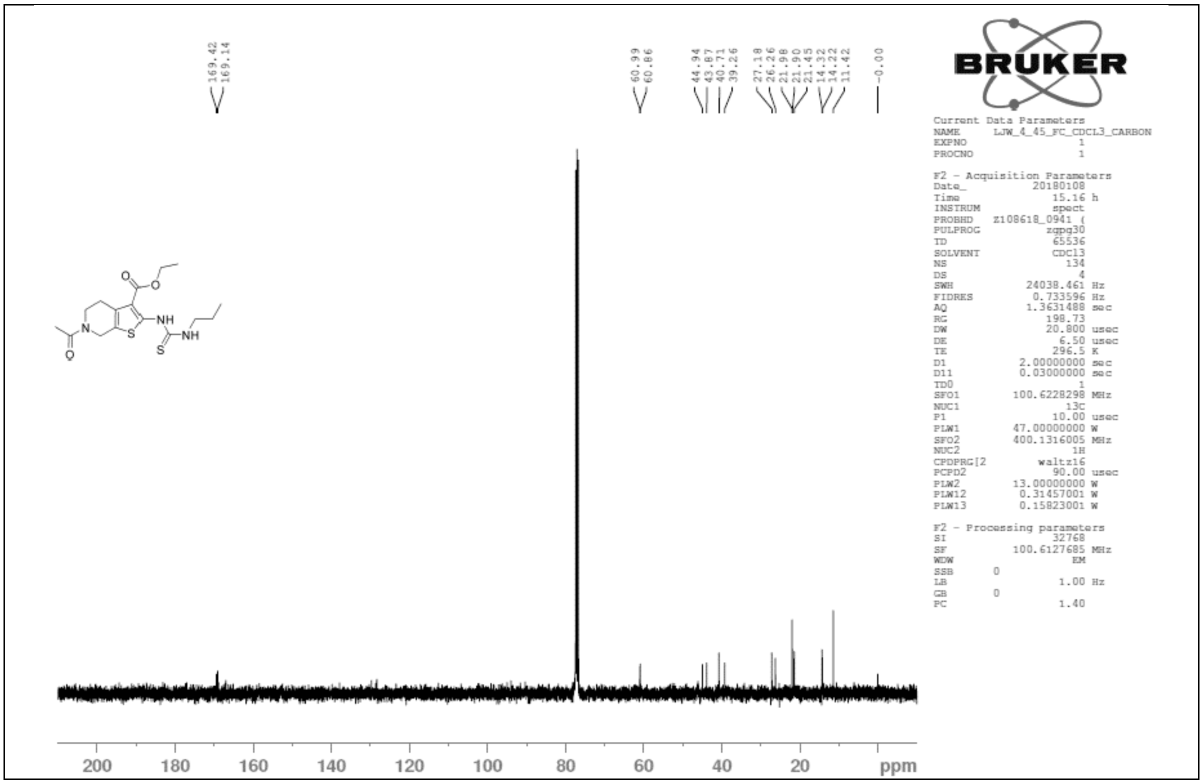

Figure S14. ^{1}H NMR Spectrum of **24e** in $CDCl_3$

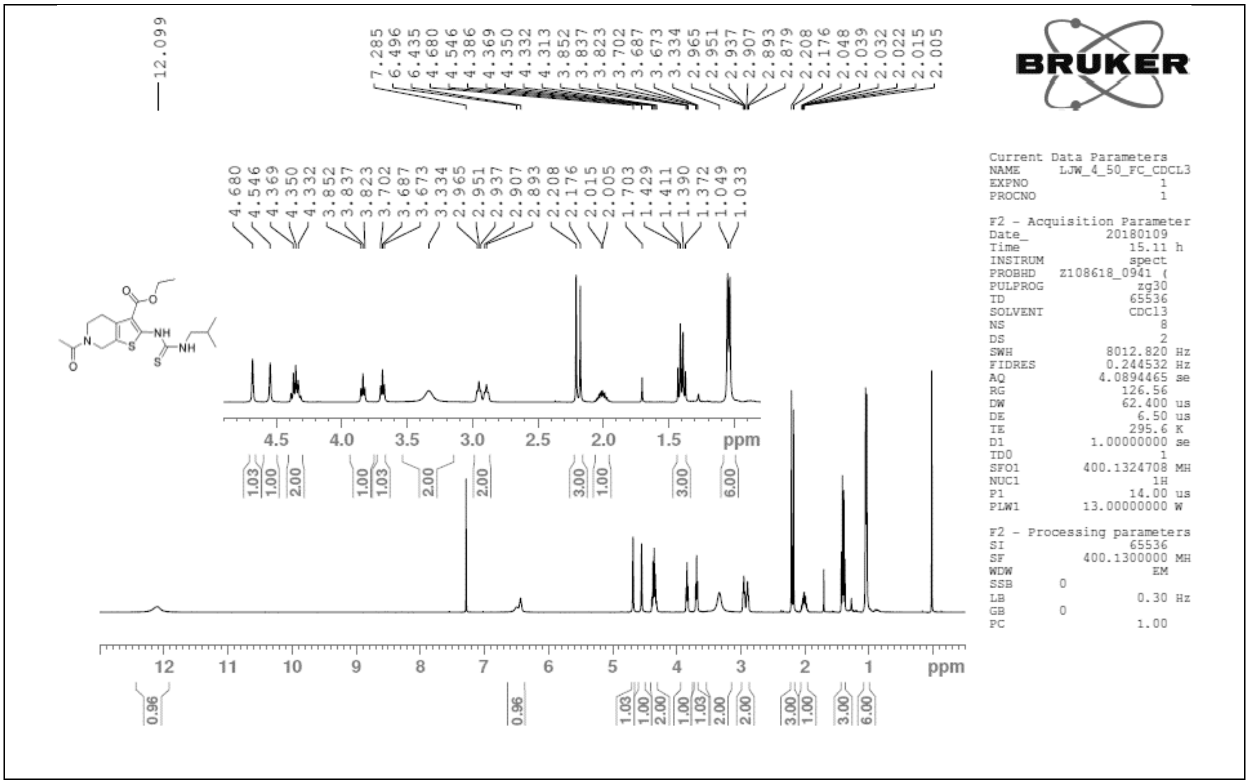

Figure S15. ^{13}C NMR Spectrum of **24e** in $CDCl_3$

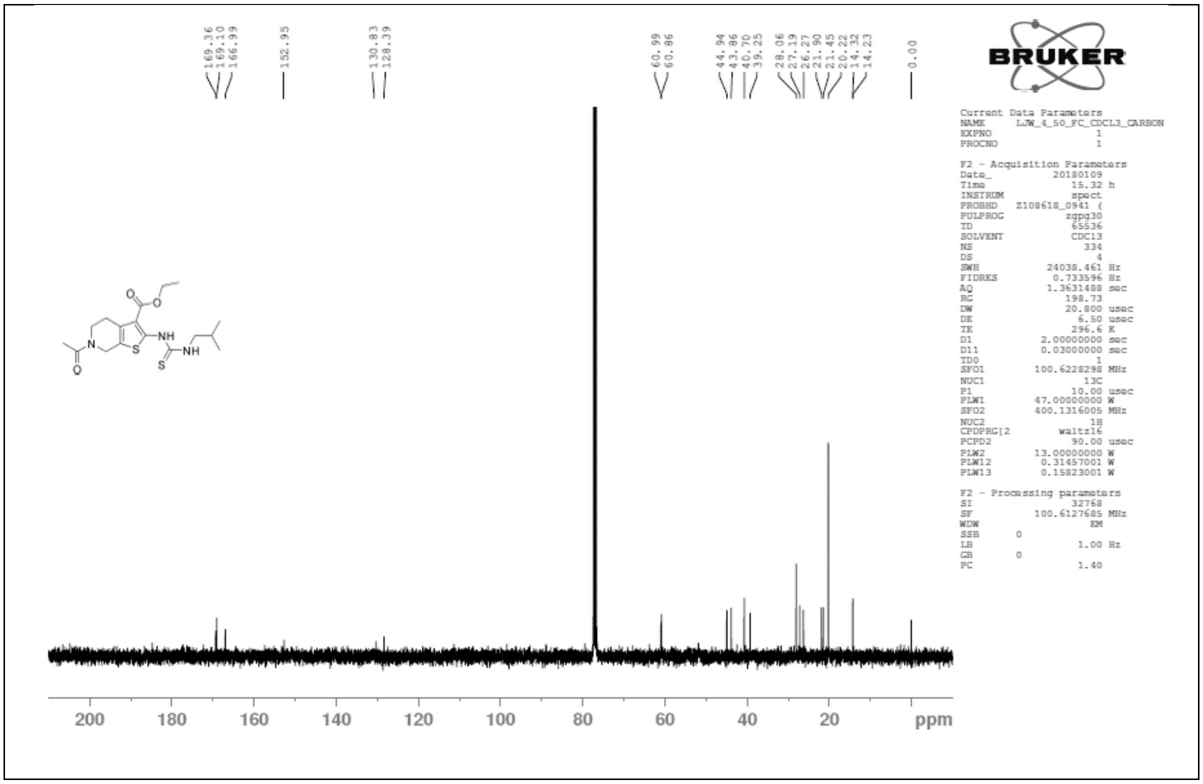

Figure S16. ^{1}H NMR Spectrum of **24f** in $CDCl_3$

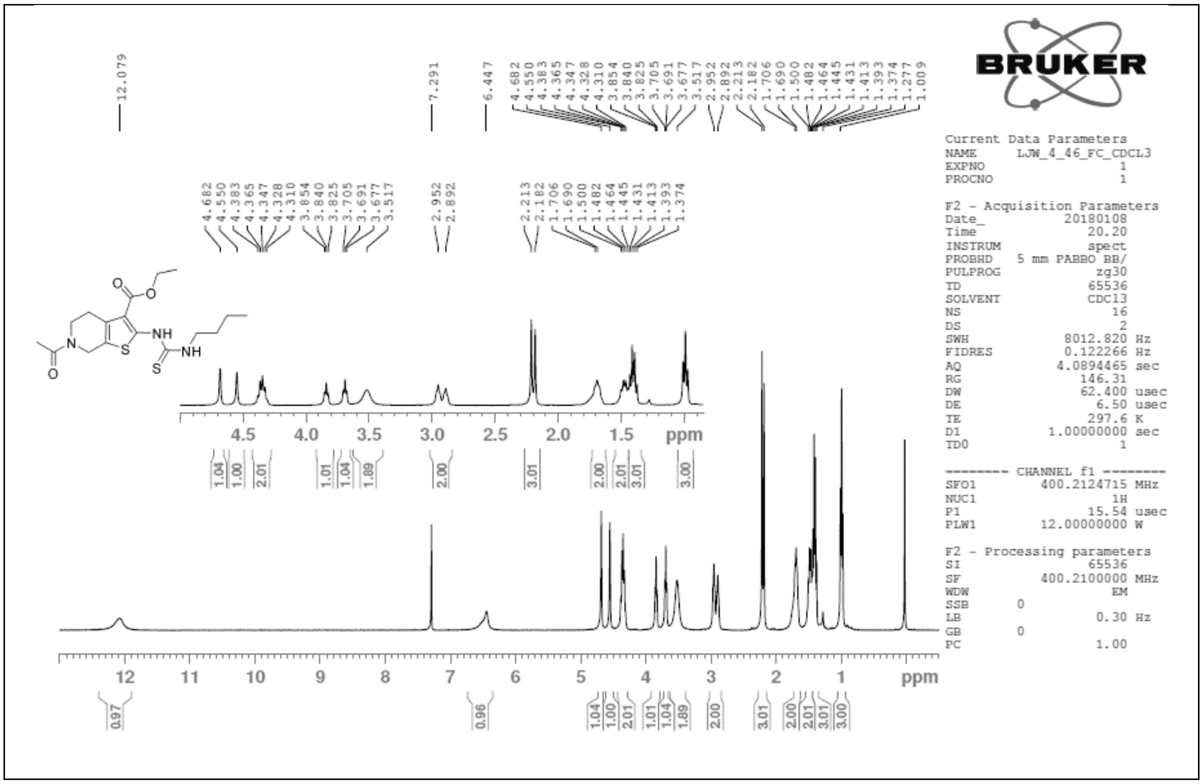

Figure S17. ^{13}C NMR Spectrum of **24f** in $CDCl_3$

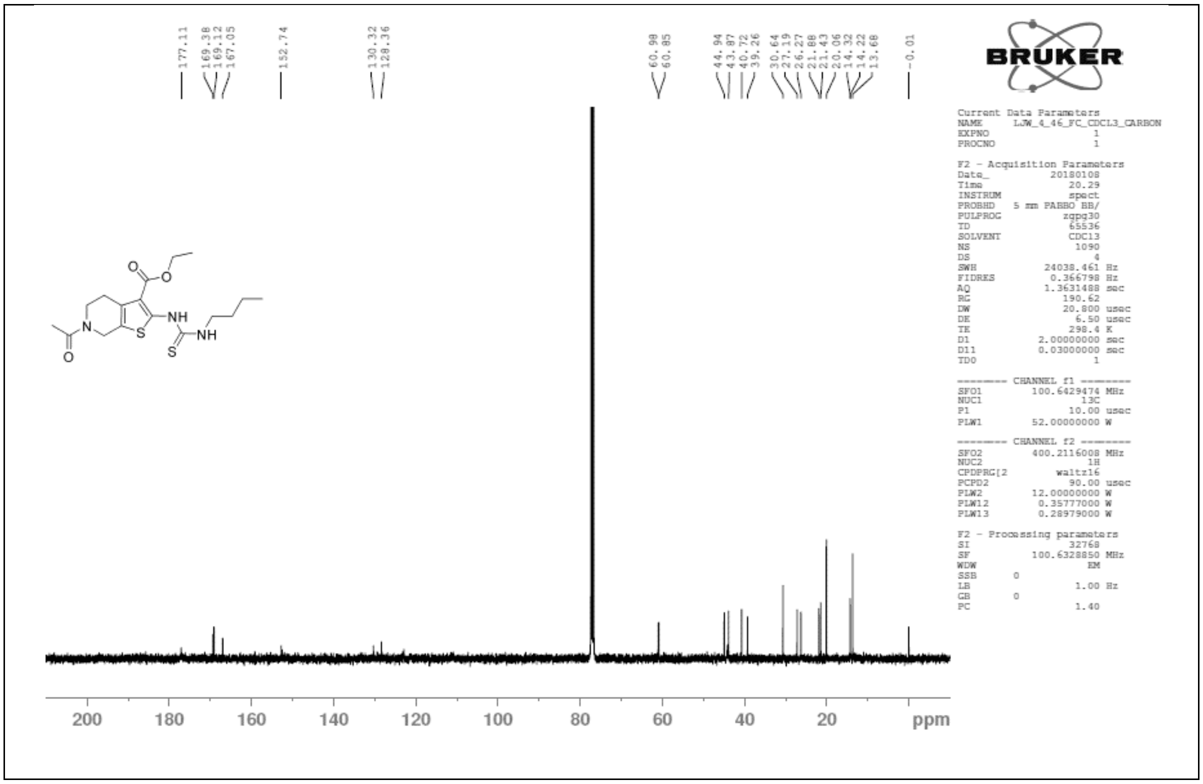

Figure S18. ^{1}H NMR Spectrum of **24g** in $CDCl_3$

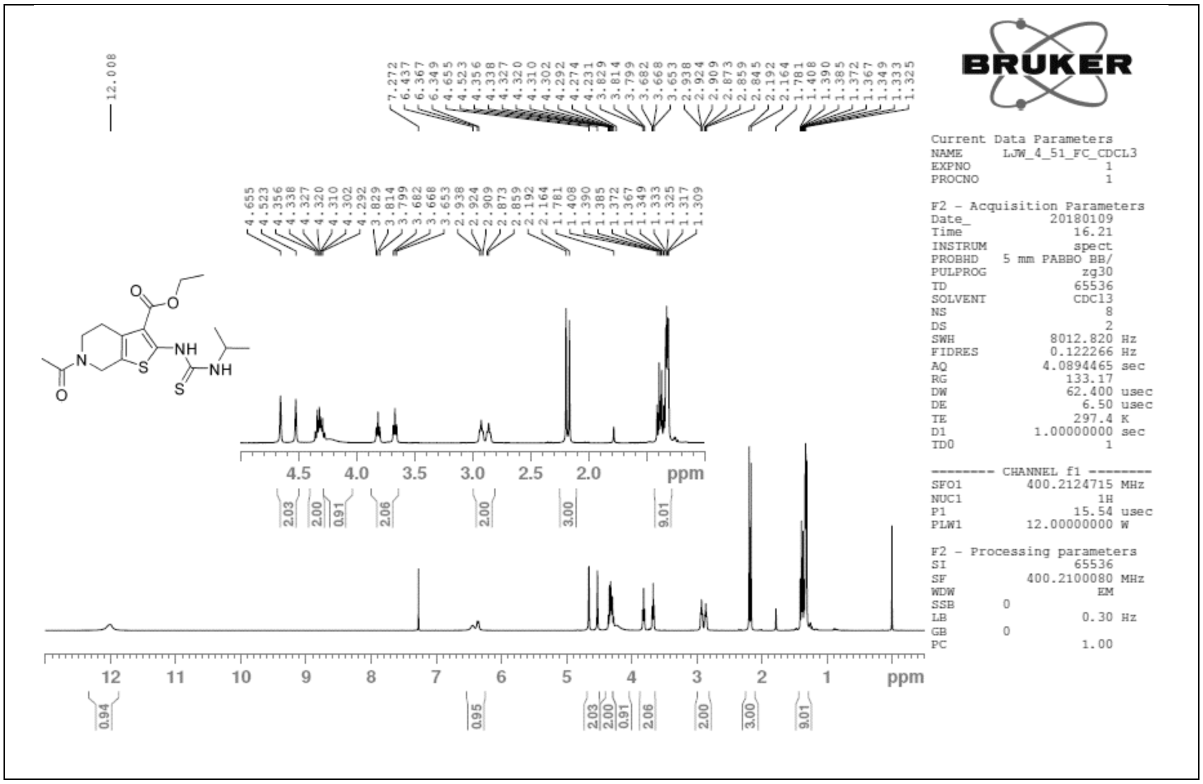

Figure S19. ^{13}C NMR Spectrum of **24g** in $CDCl_3$

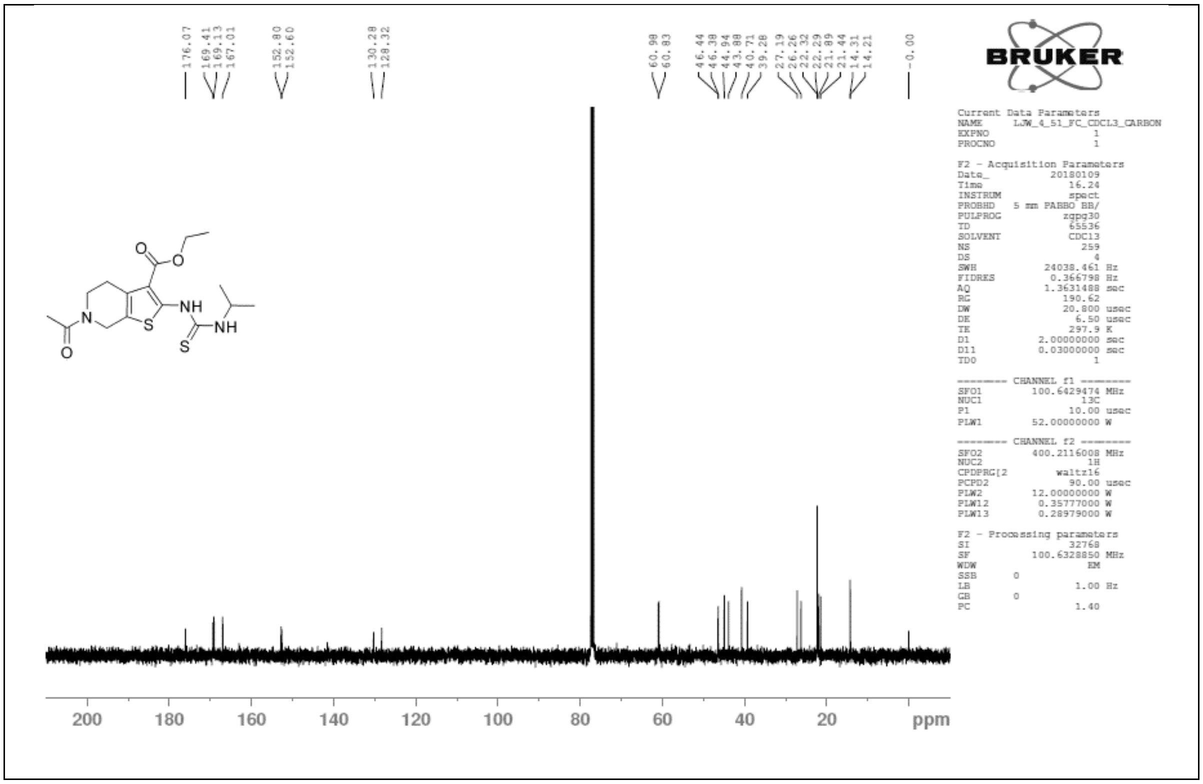

Figure S20. ^{1}H NMR Spectrum of **25aa** in DMSO-d_6

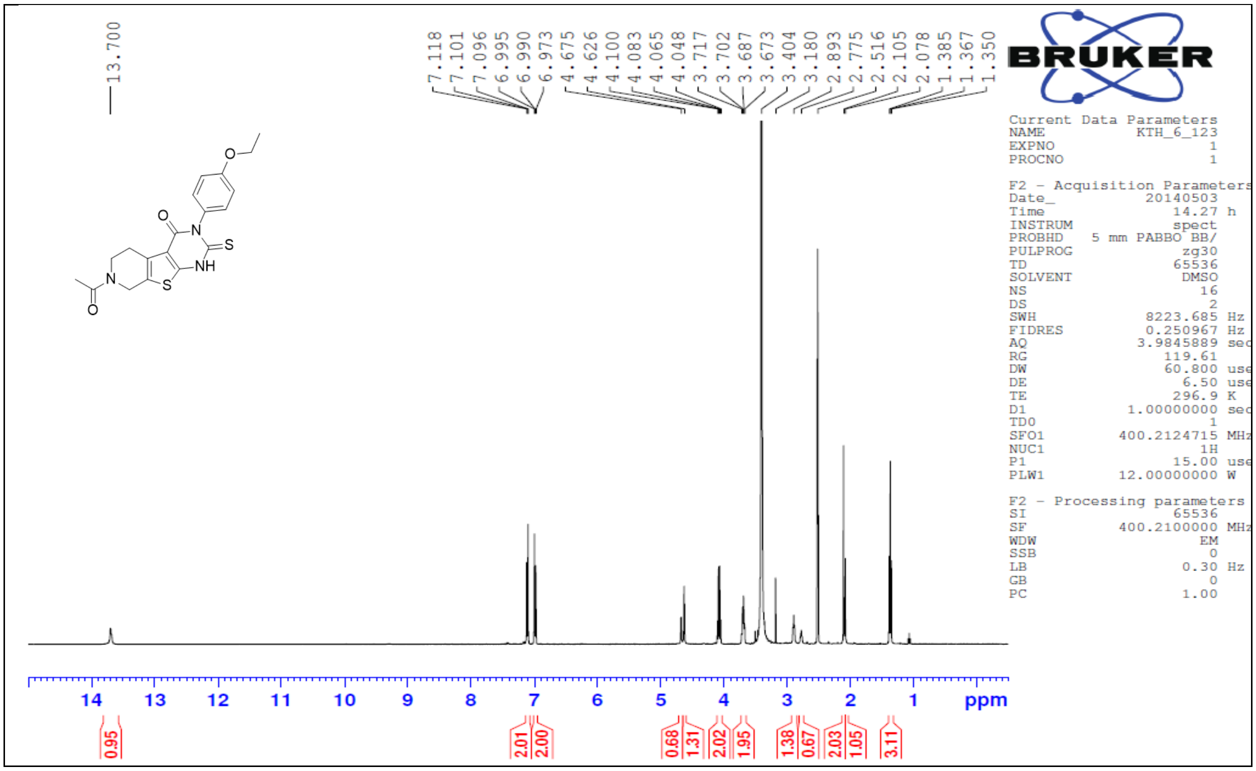

Figure S21. ^{13}C NMR Spectrum of **25aa** in DMSO-d_6

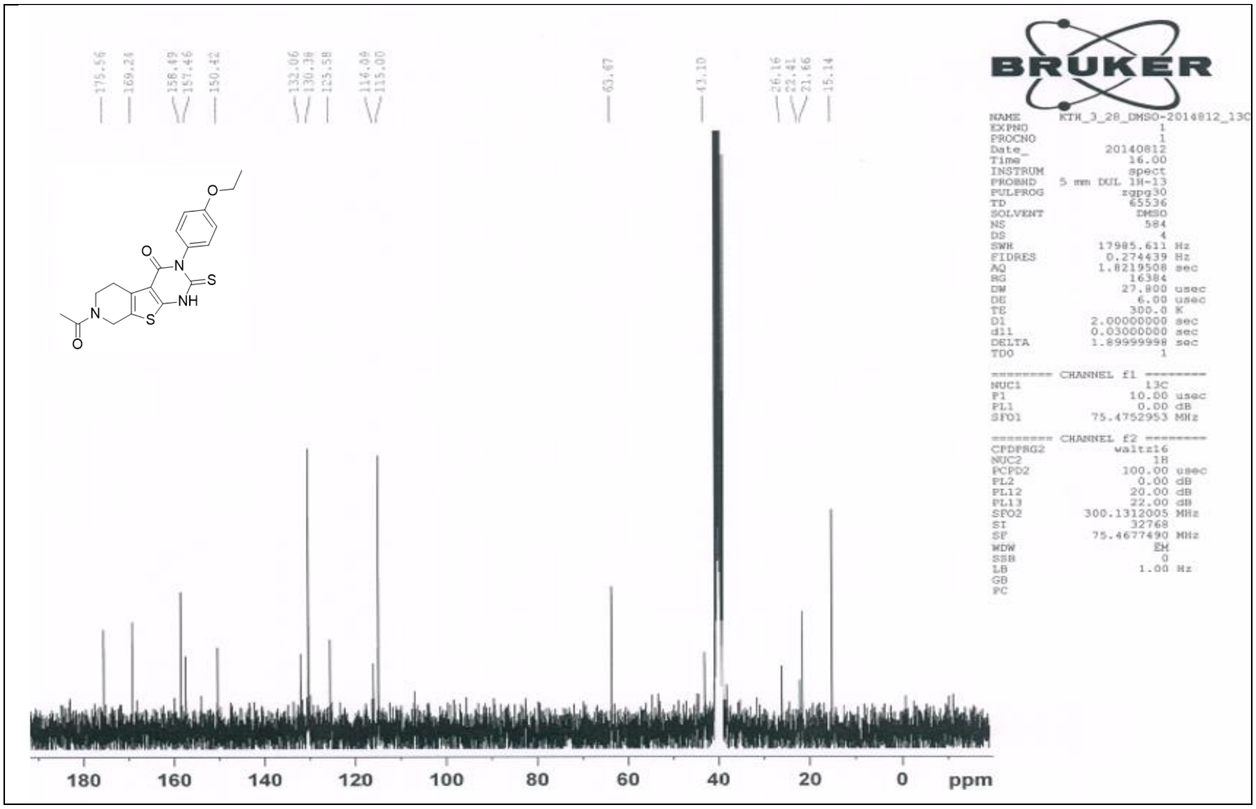

Figure S22. ^{1}H NMR Spectrum of **25ab** in DMSO-d$_6$

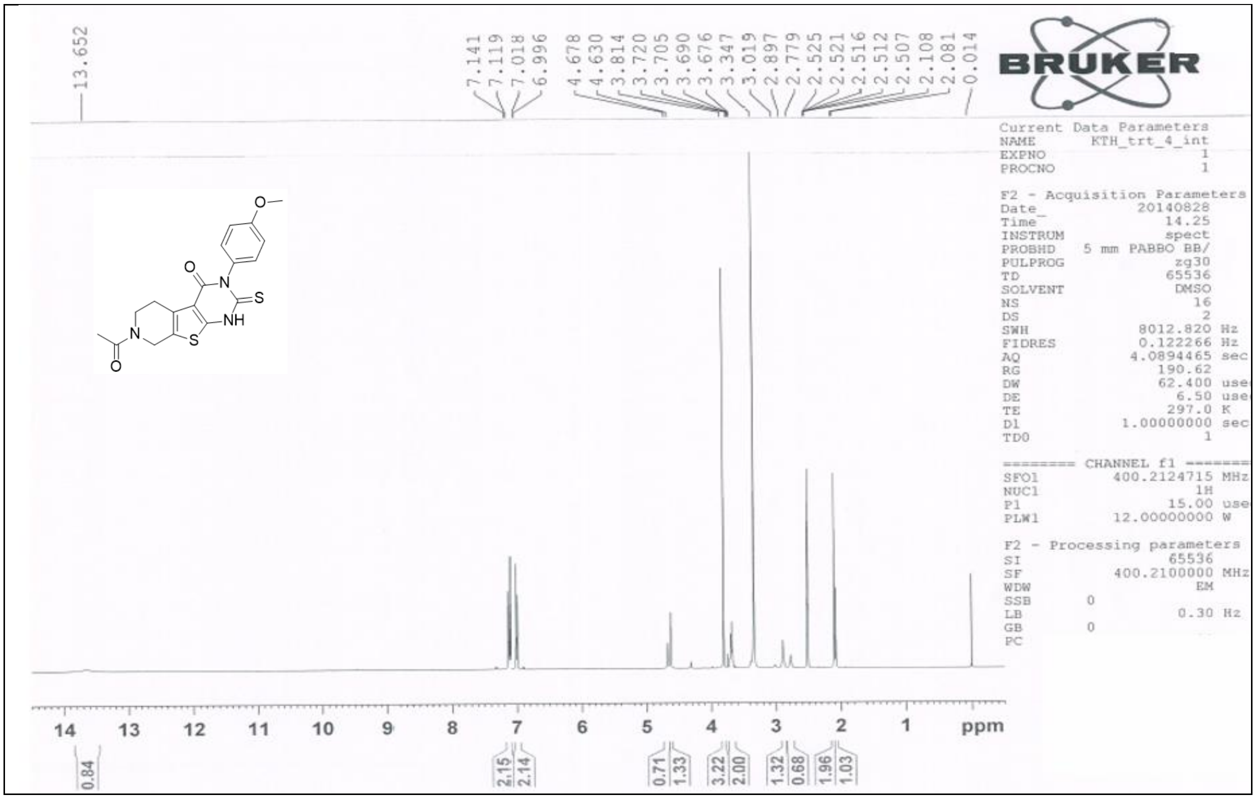

Figure S23. ^{13}C NMR Spectrum of **25ab** in DMSO-d$_6$

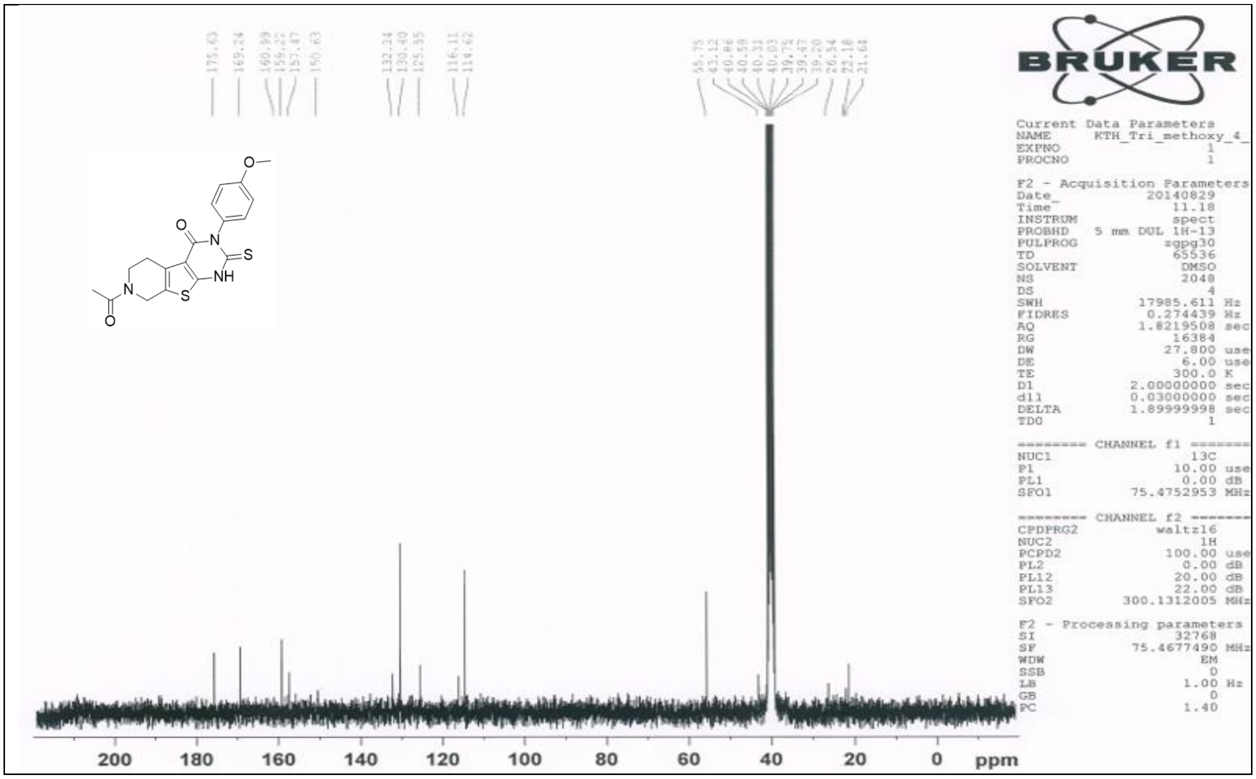

Figure S24. ^{1}H NMR Spectrum of **25b** in MeOD

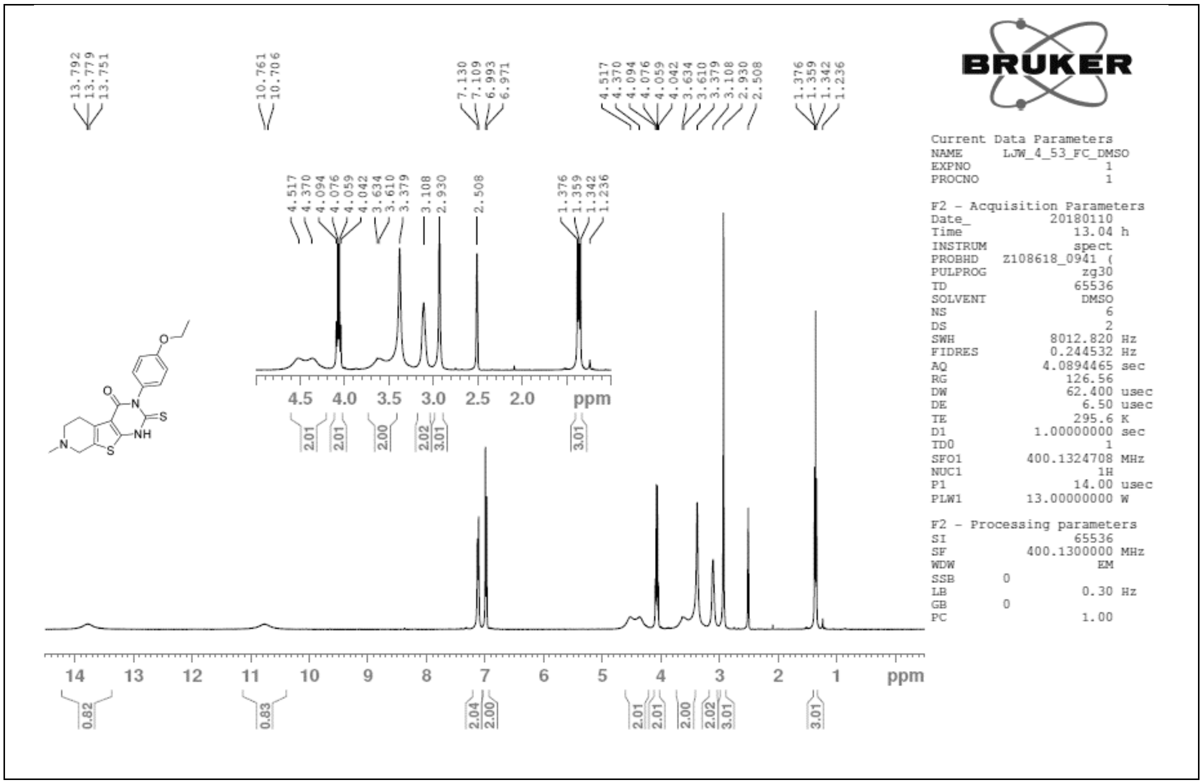

Figure S25. ^{13}C NMR Spectrum of **25b** in $CDCl_3$

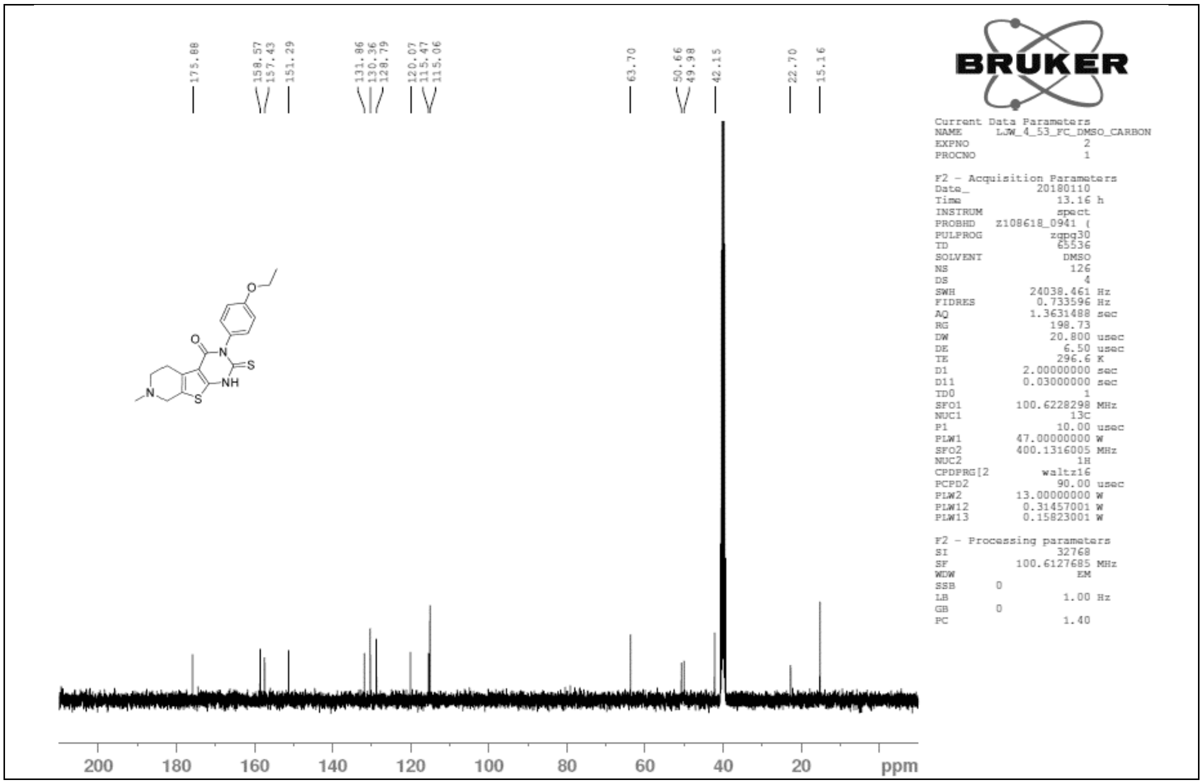

Figure S26. ^{1}H NMR Spectrum of **25c** in DMSO-d$_6$

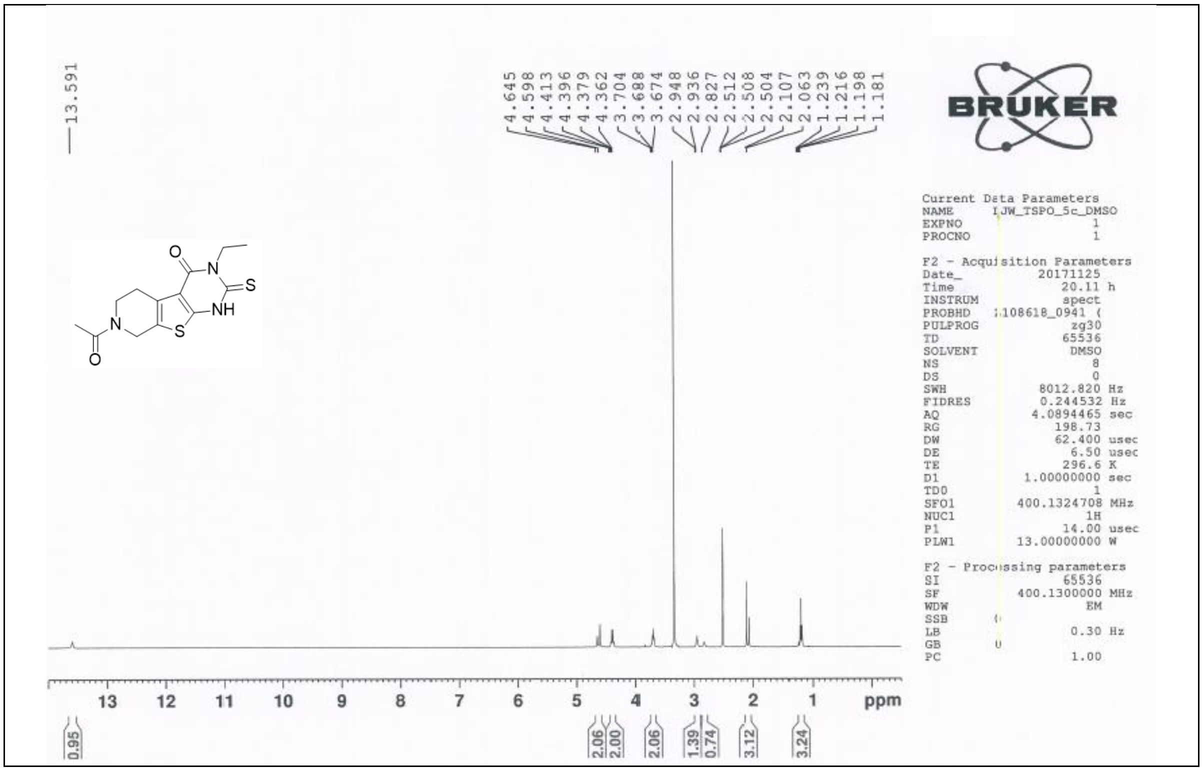

Figure S27. ^{13}C NMR Spectrum of **25c** in DMSO-d$_6$

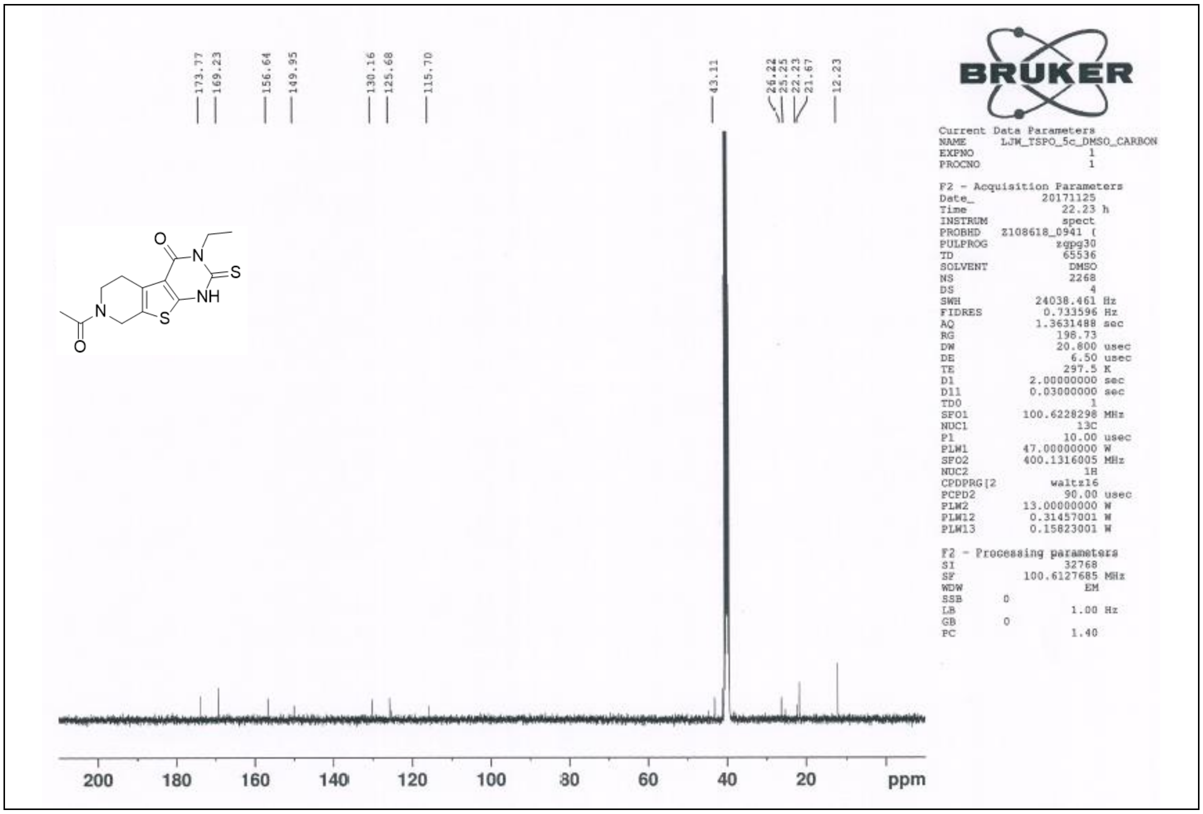

Figure S28. ^{1}H NMR Spectrum of **25d** in $CDCl_3$

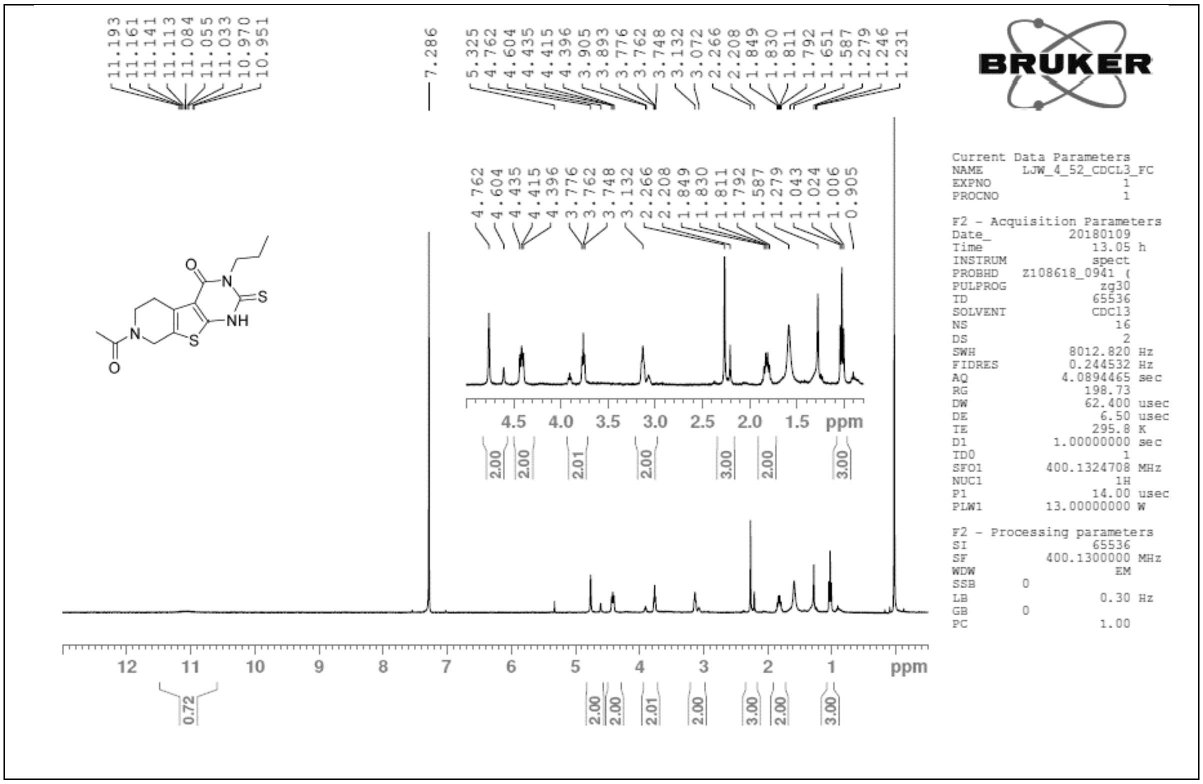

Figure S29. ^{13}C NMR Spectrum of **25d** in DMSO-d_6

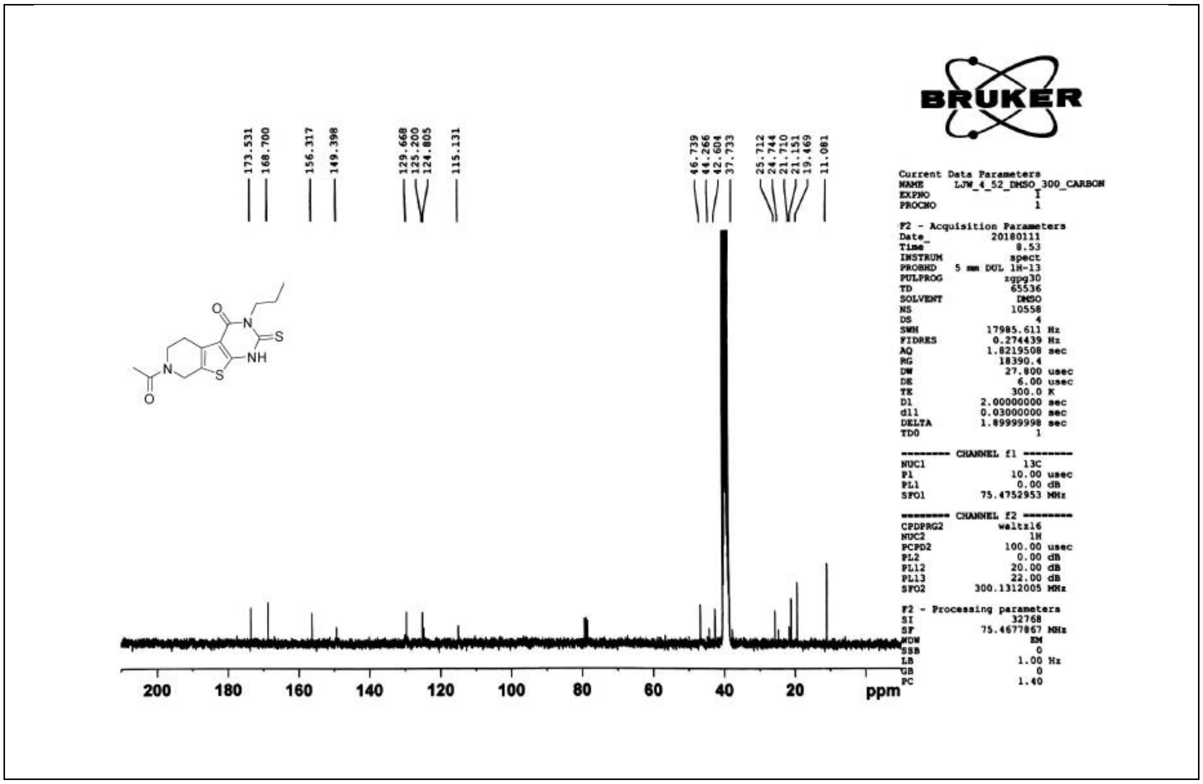

Figure S30. ^{1}H NMR Spectrum of **25e** in $CDCl_3$

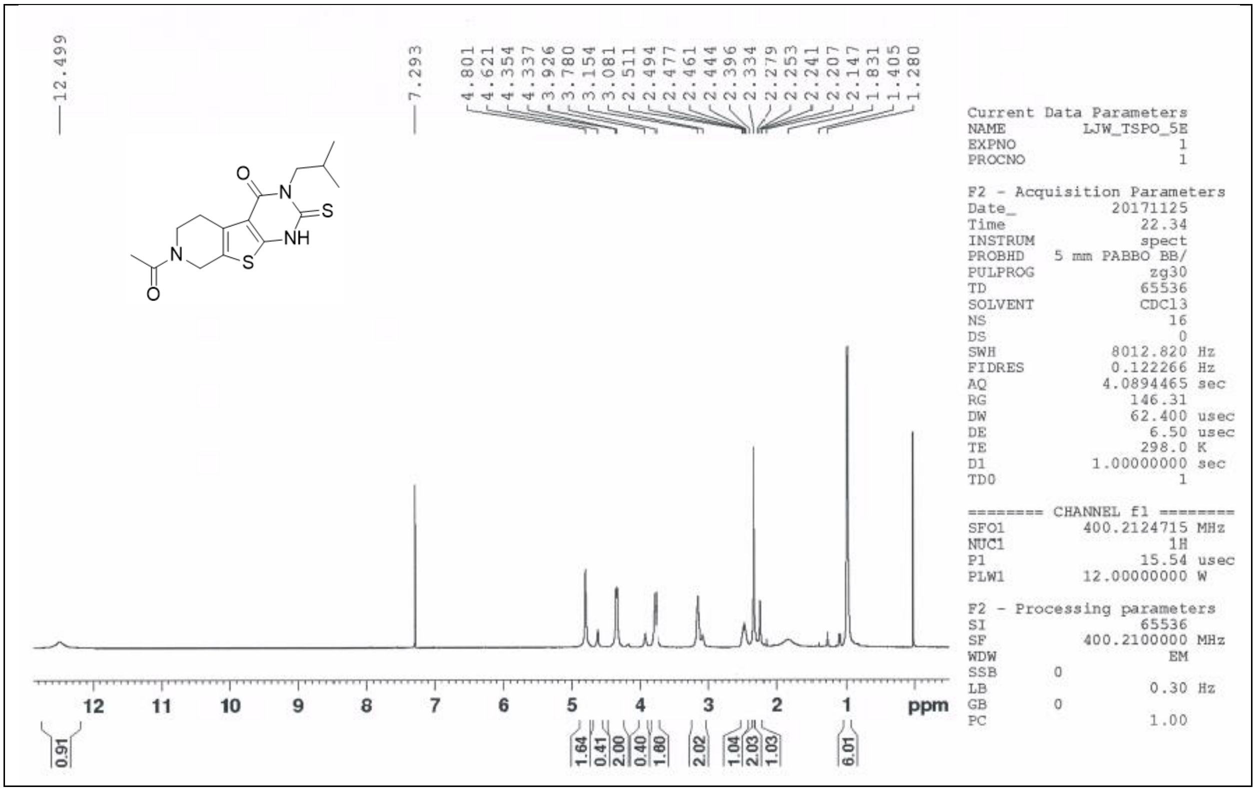

Figure S31. ^{13}C NMR Spectrum of **25e** in $CDCl_3$

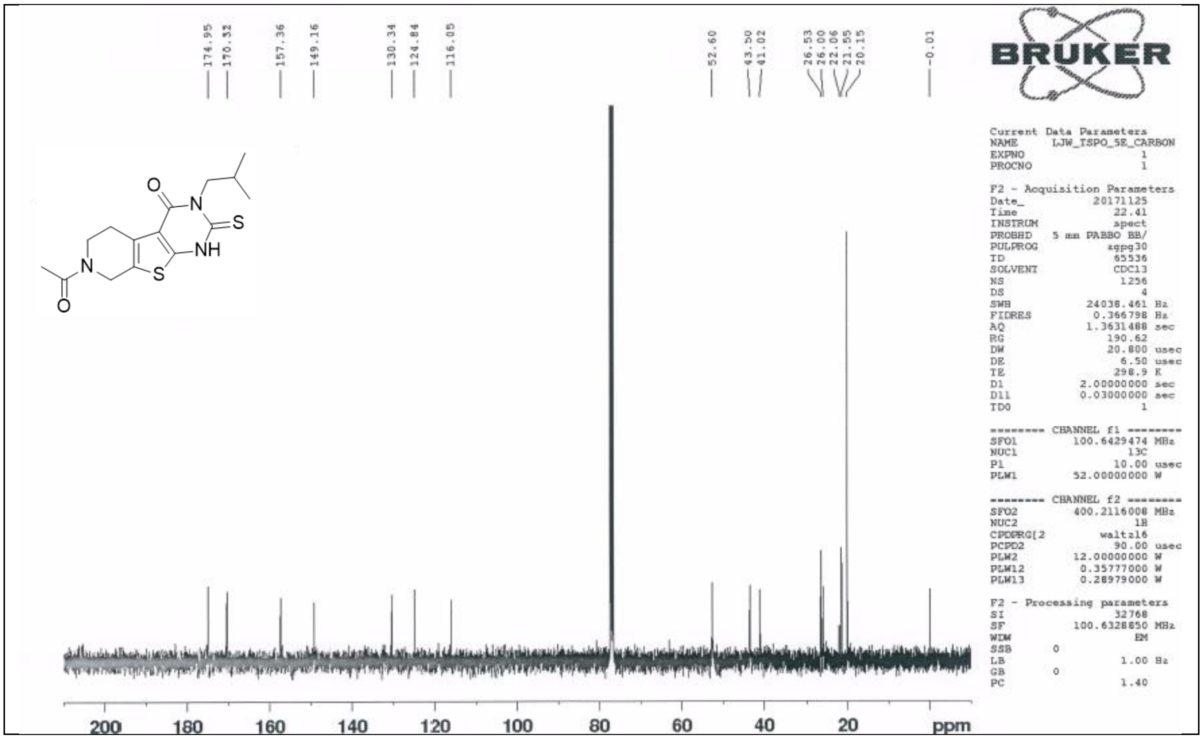

Figure S32. ^{1}H NMR Spectrum of **25f** in $CDCl_3$

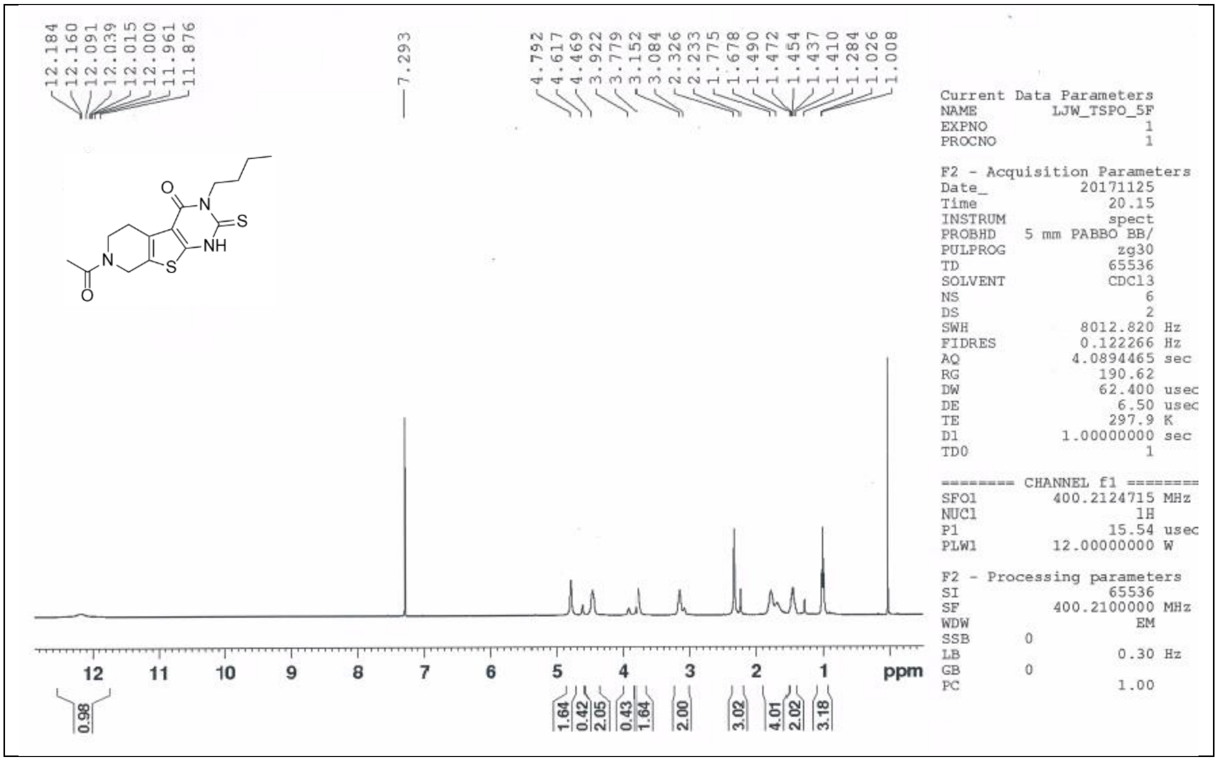

Figure S33. ^{13}C NMR Spectrum of **25f** in $CDCl_3$

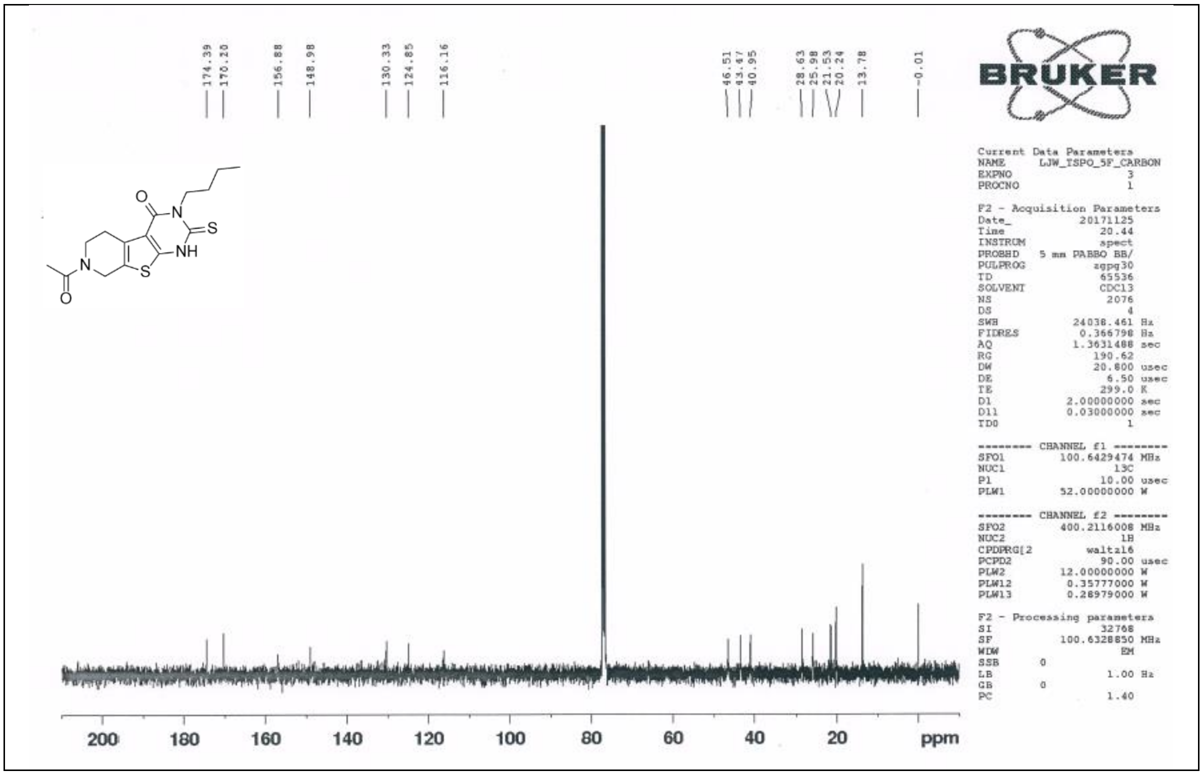

Figure S34. ^{1}H NMR Spectrum of **25g** in $CDCl_3$

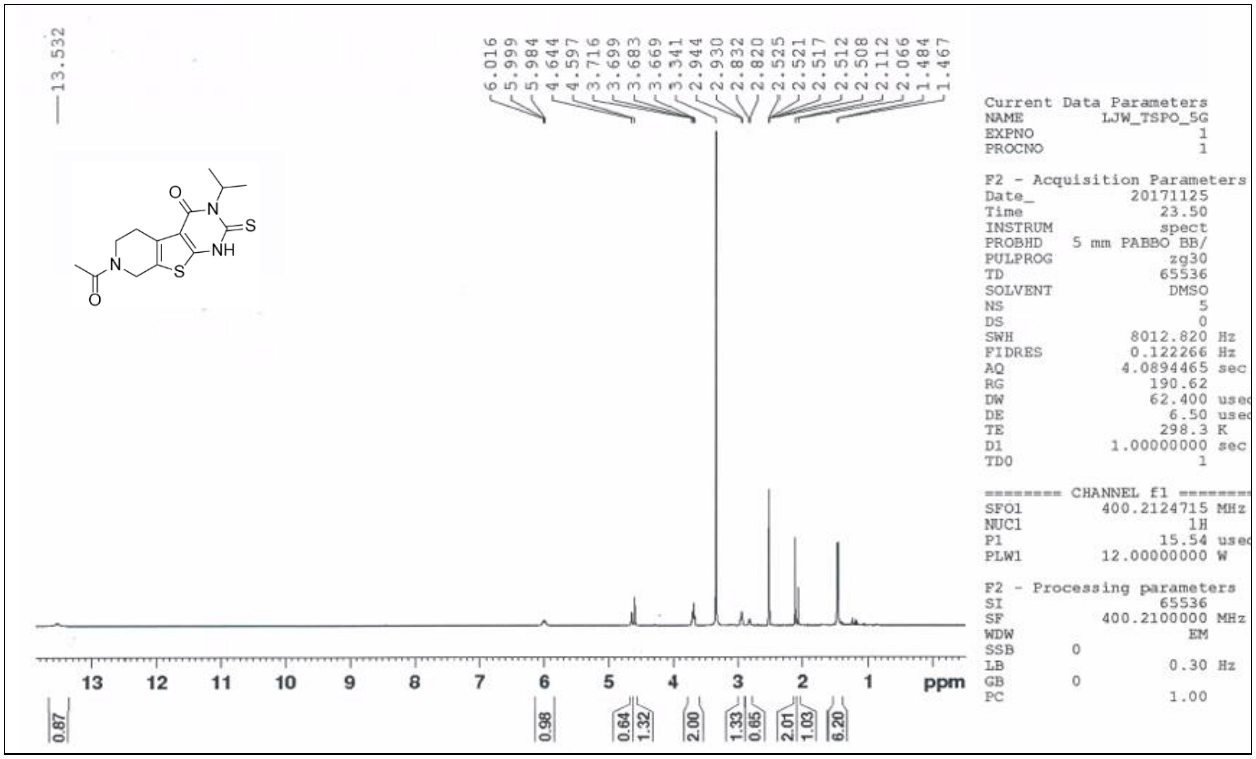

Figure S35. ^{13}C NMR Spectrum of **25g** in DMSO-d_6

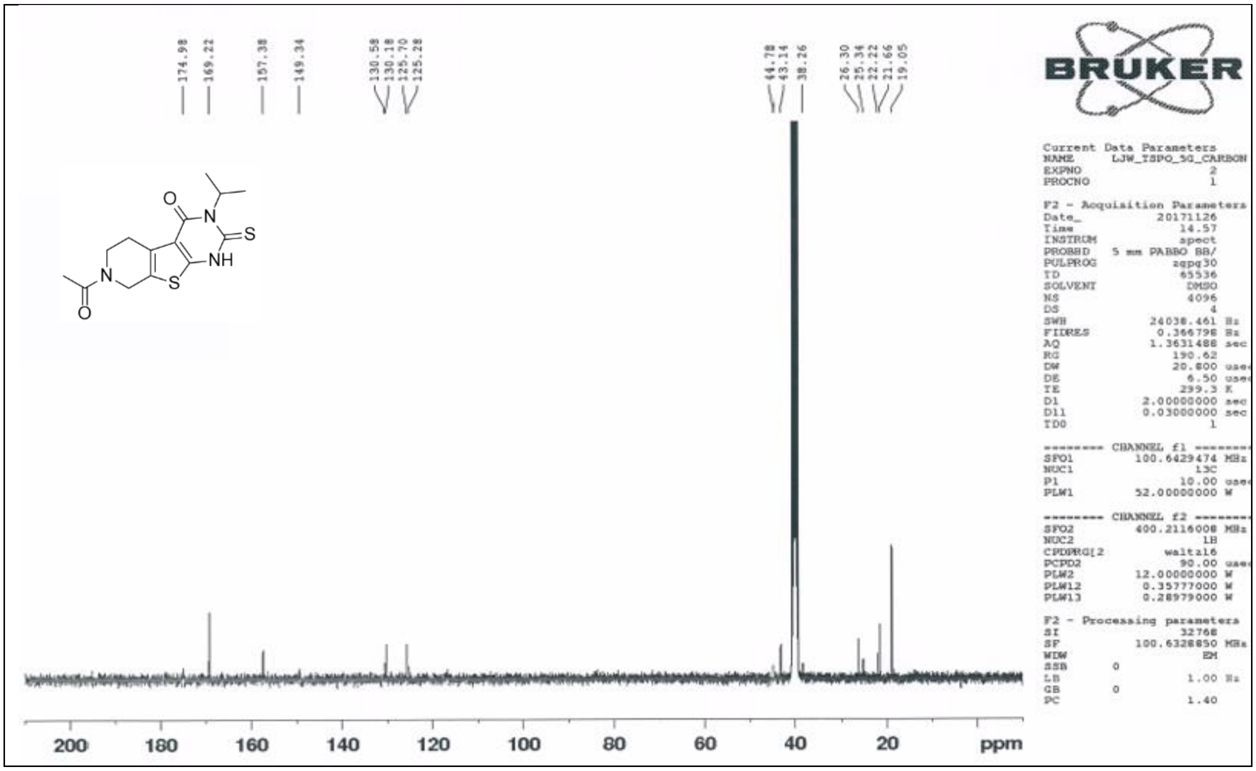

Figure S36. ^{1}H NMR Spectrum of **14** in $CDCl_3$

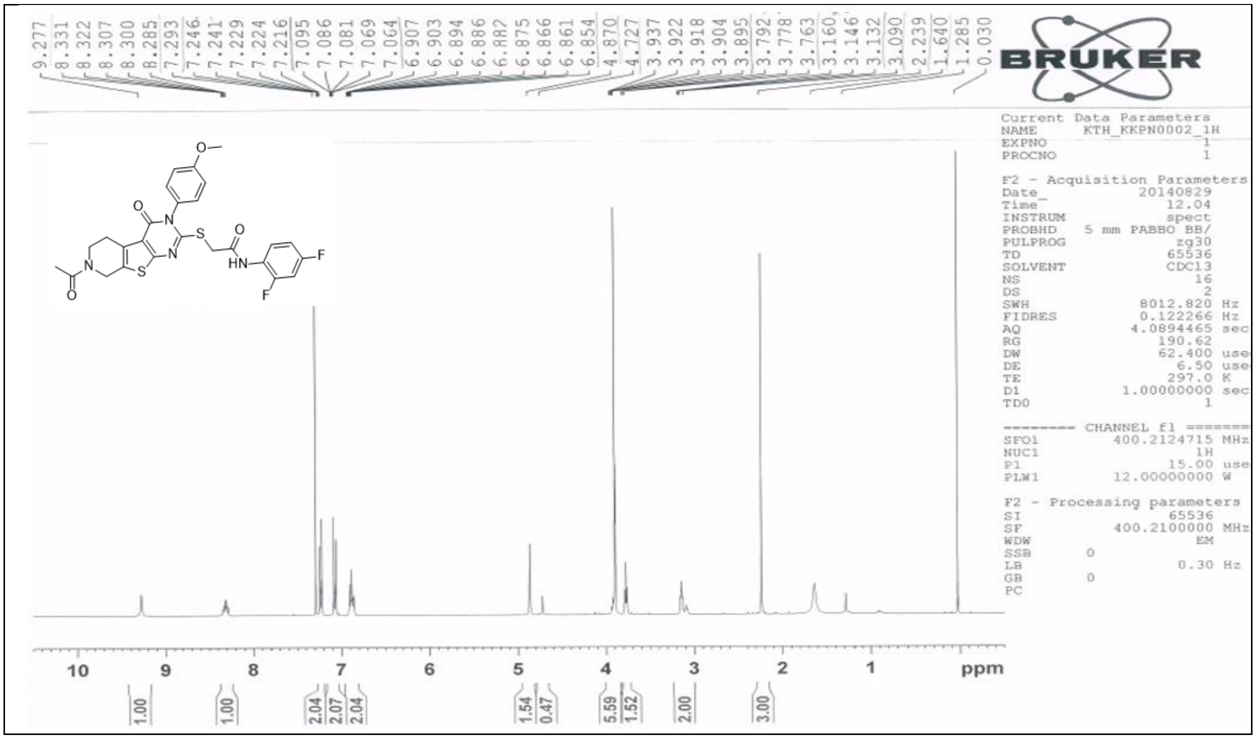

Figure S37. ^{13}C NMR Spectrum of **14** in $CDCl_3$

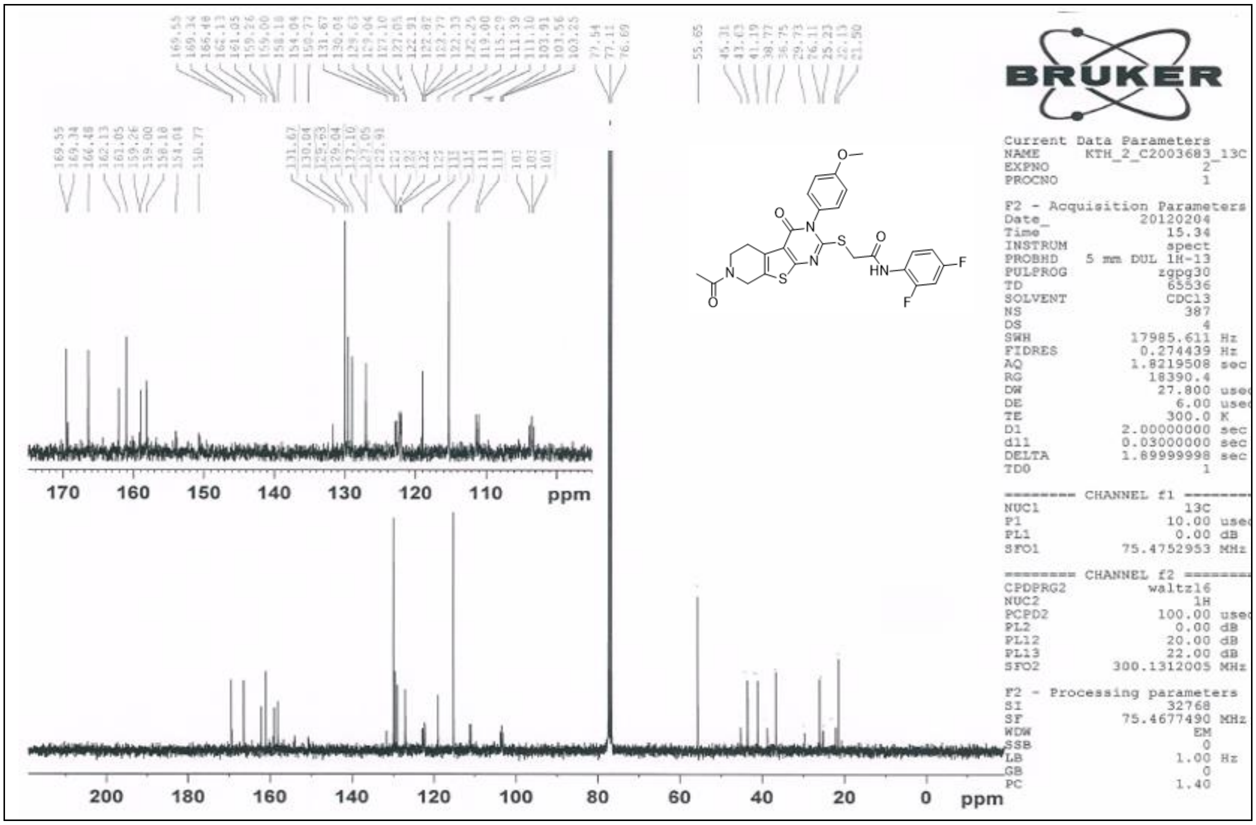

Figure S38. ^{1}H NMR Spectrum of **27** in $CDCl_3$

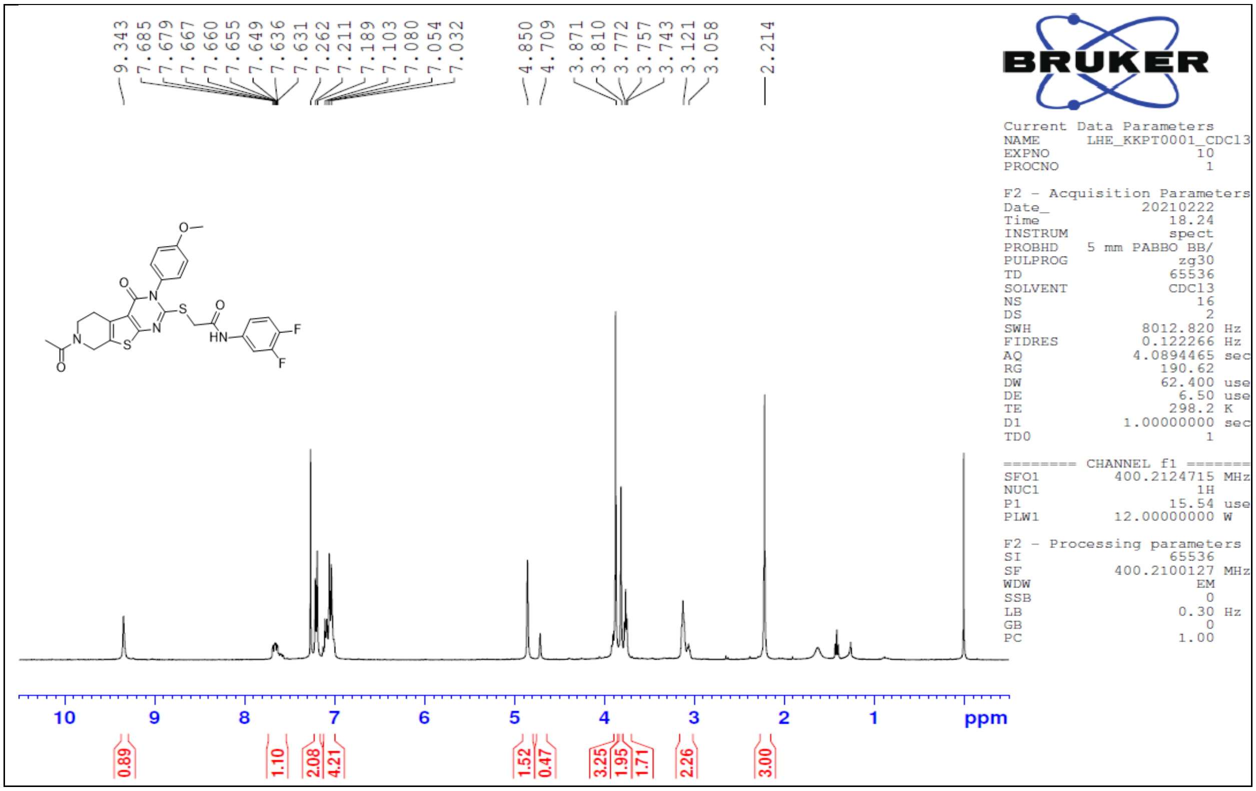

Figure S39. ^{13}C NMR Spectrum of **27** in $CDCl_3$

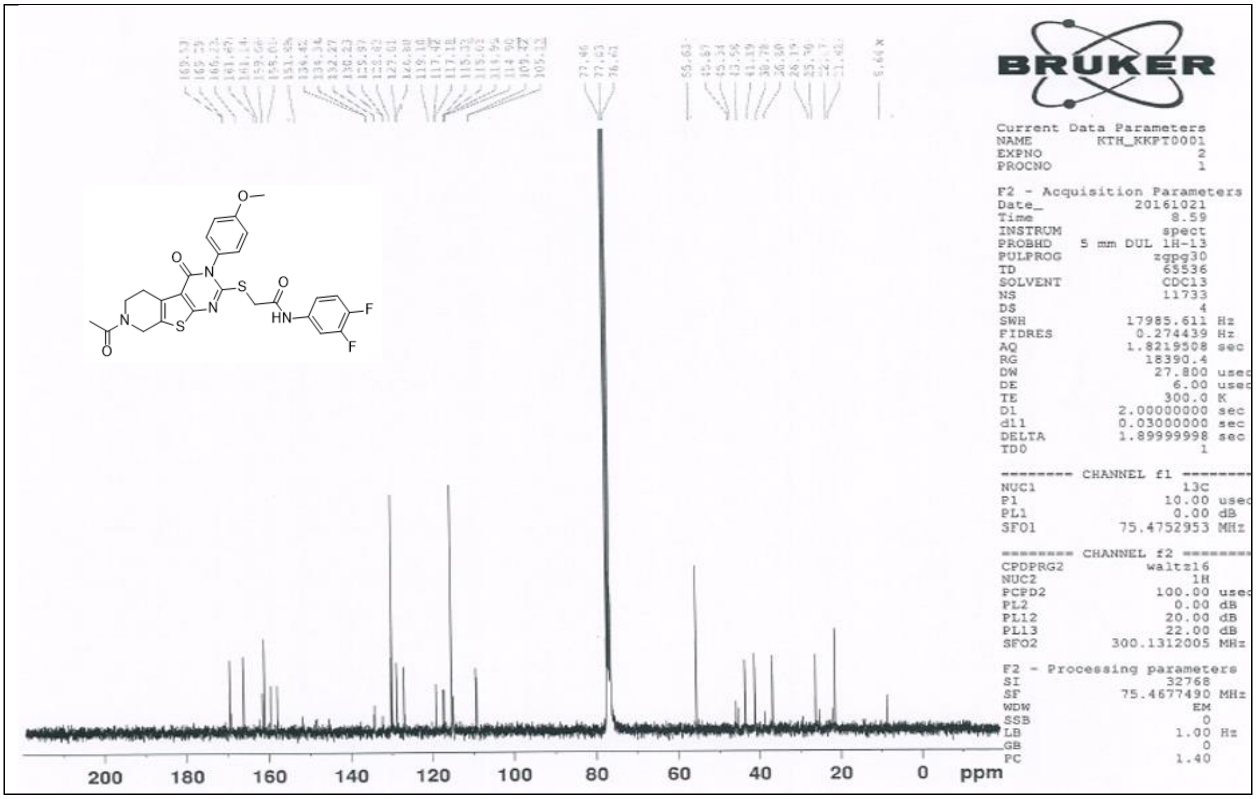

Figure S40. ^{1}H NMR Spectrum of **28** in $CDCl_3$

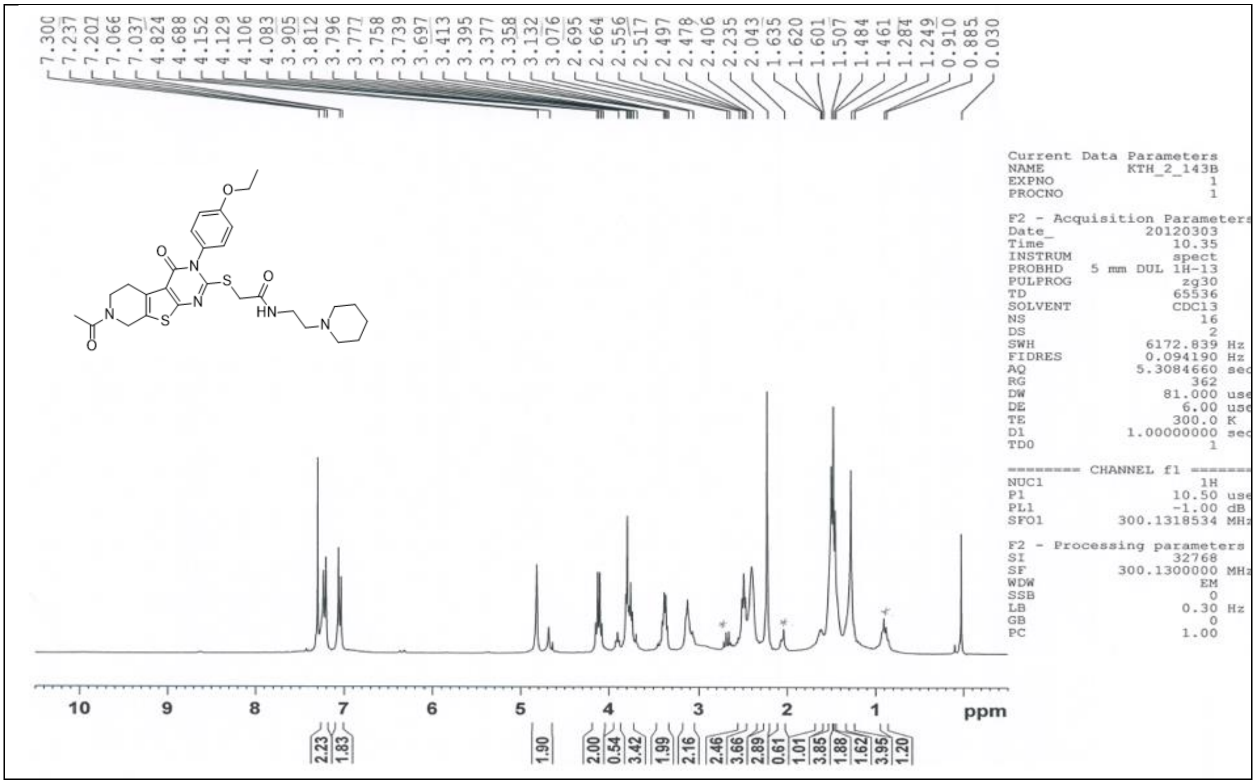

Figure S41. ^{13}C NMR Spectrum of **28** in $CDCl_3$

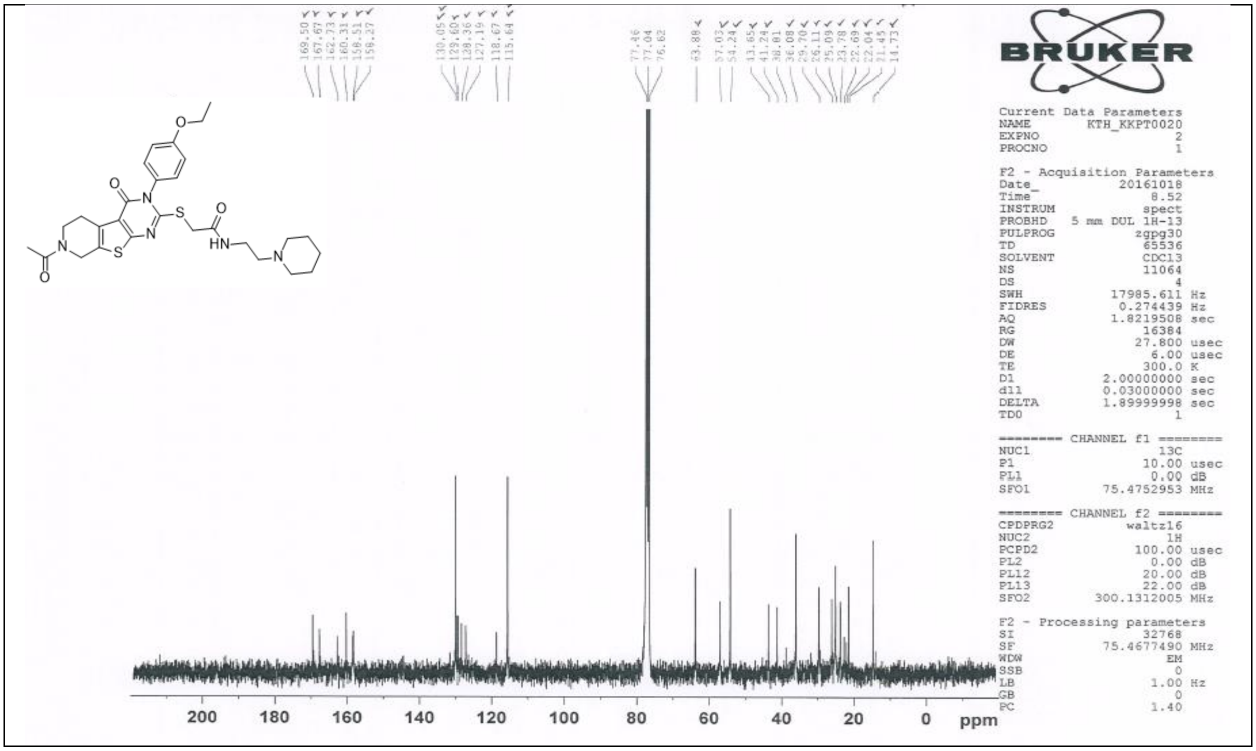

Figure S42. ^{1}H NMR Spectrum of **29** in $CDCl_3$

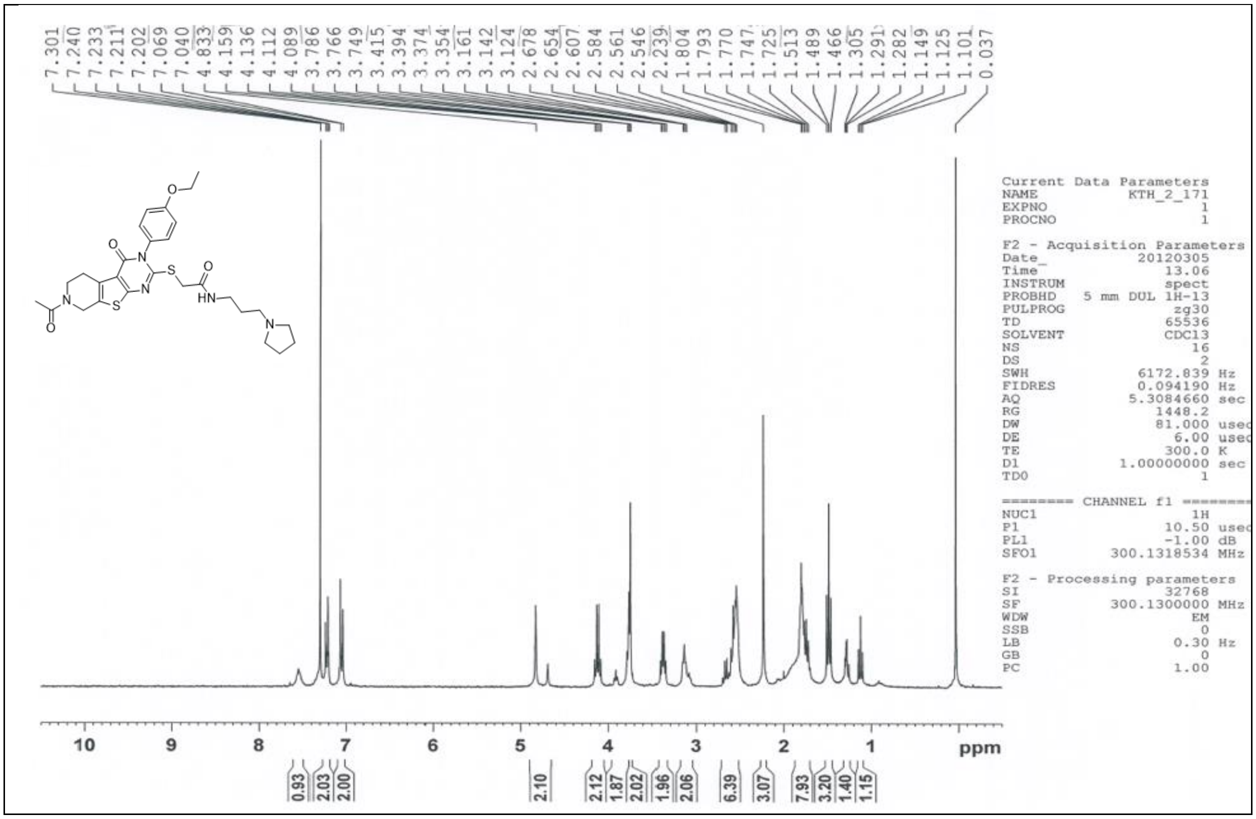

Figure S43. ^{13}C NMR Spectrum of **29** in $CDCl_3$

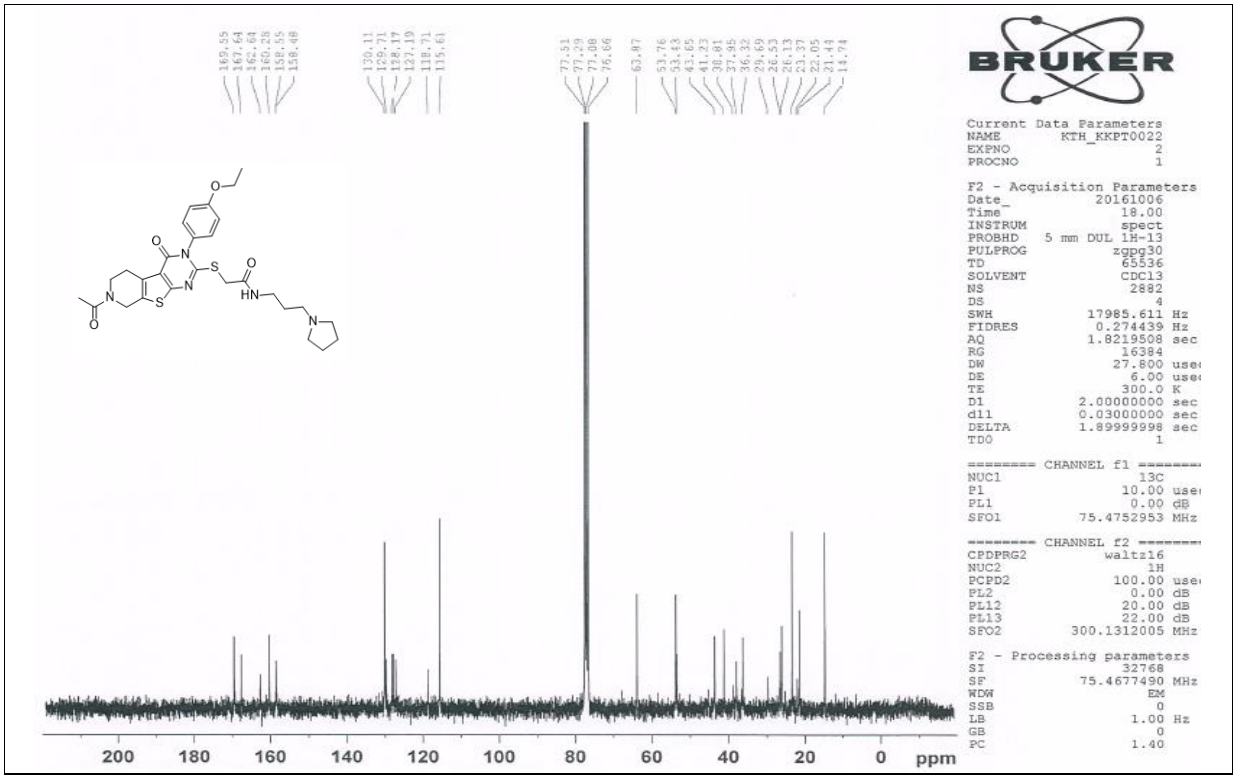

Figure S44. ^{1}H NMR Spectrum of **30** in $CDCl_3$

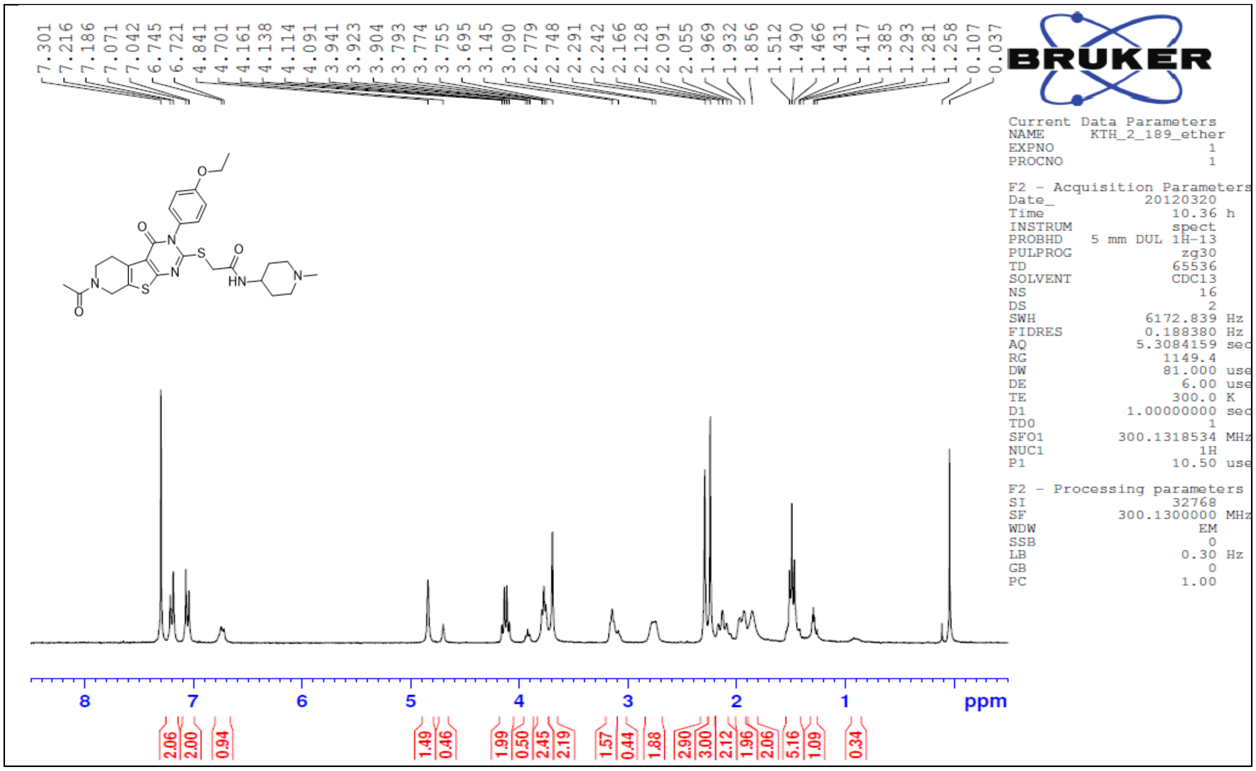

Figure S45. ^{13}C NMR Spectrum of **30** in $CDCl_3$

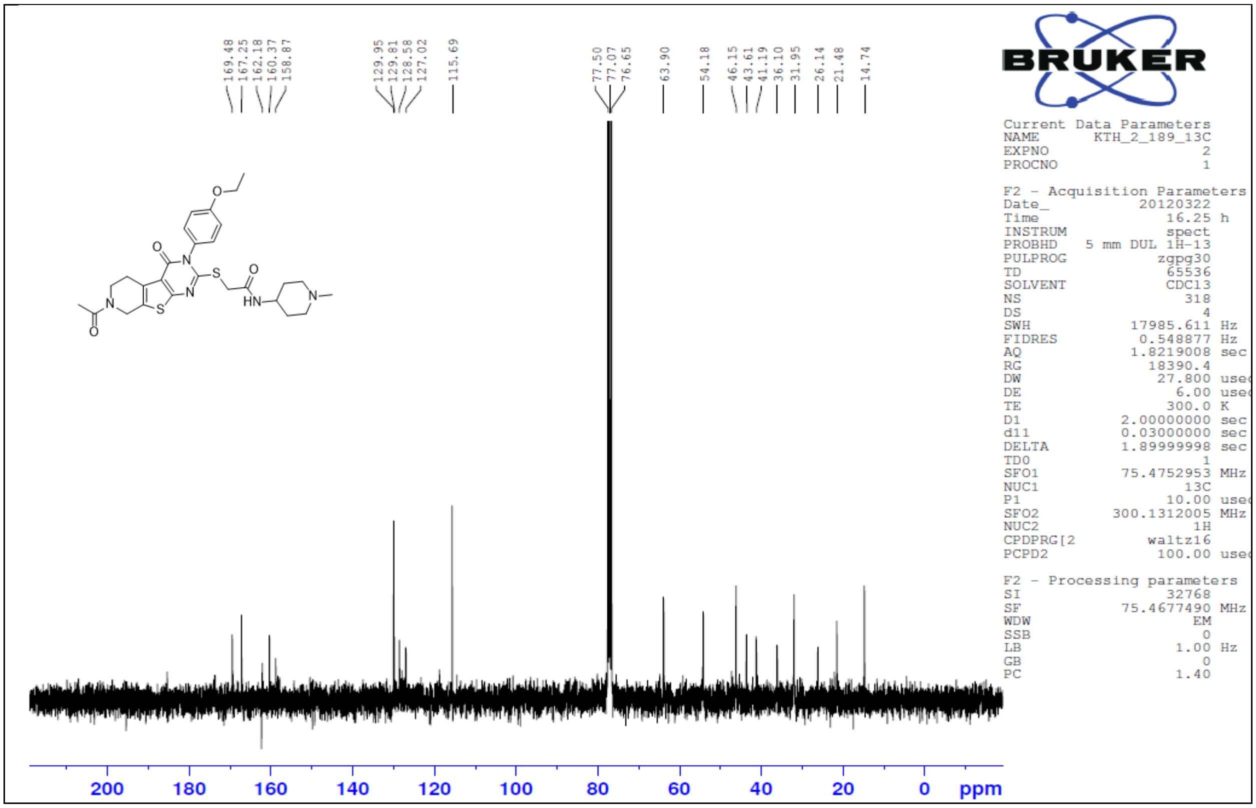

Figure S46. ^{1}H NMR Spectrum of **31** in $CDCl_3$

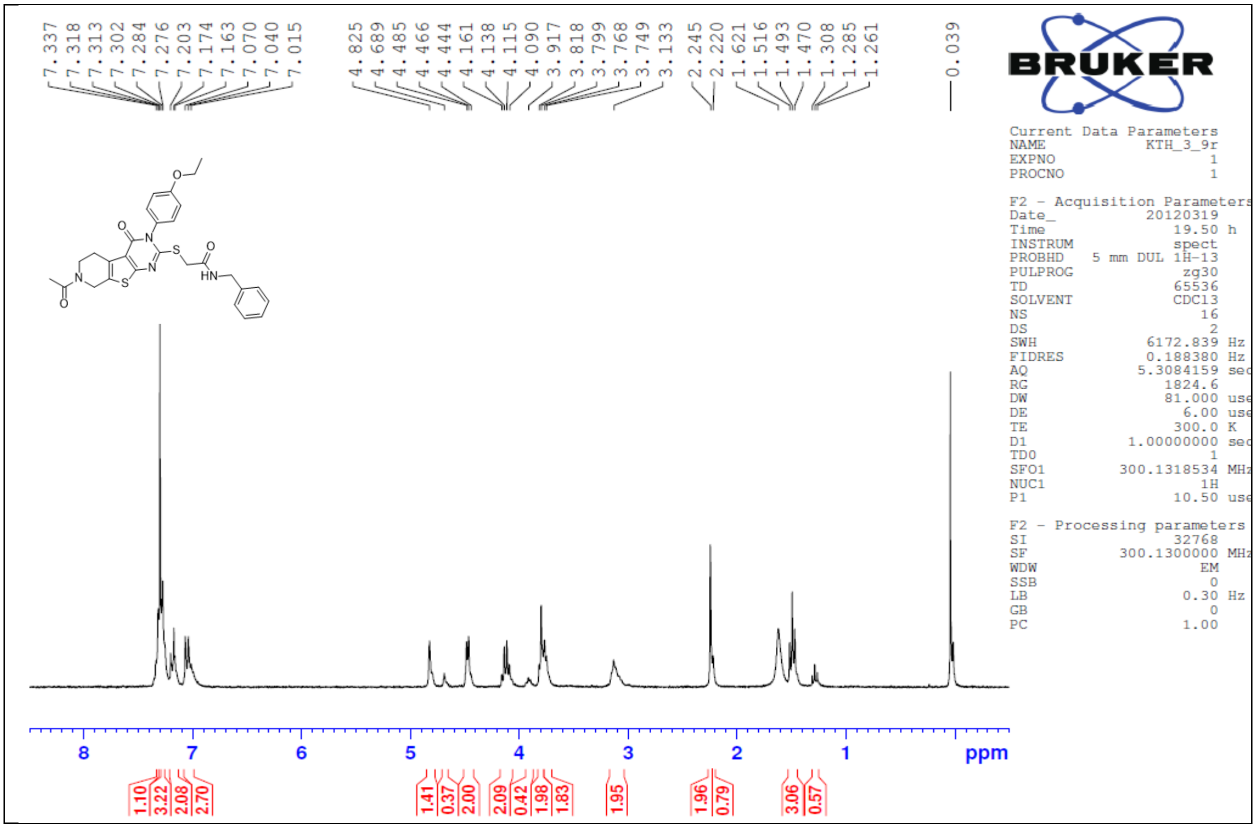

Figure S47. ^{13}C NMR Spectrum of **31** in $CDCl_3$

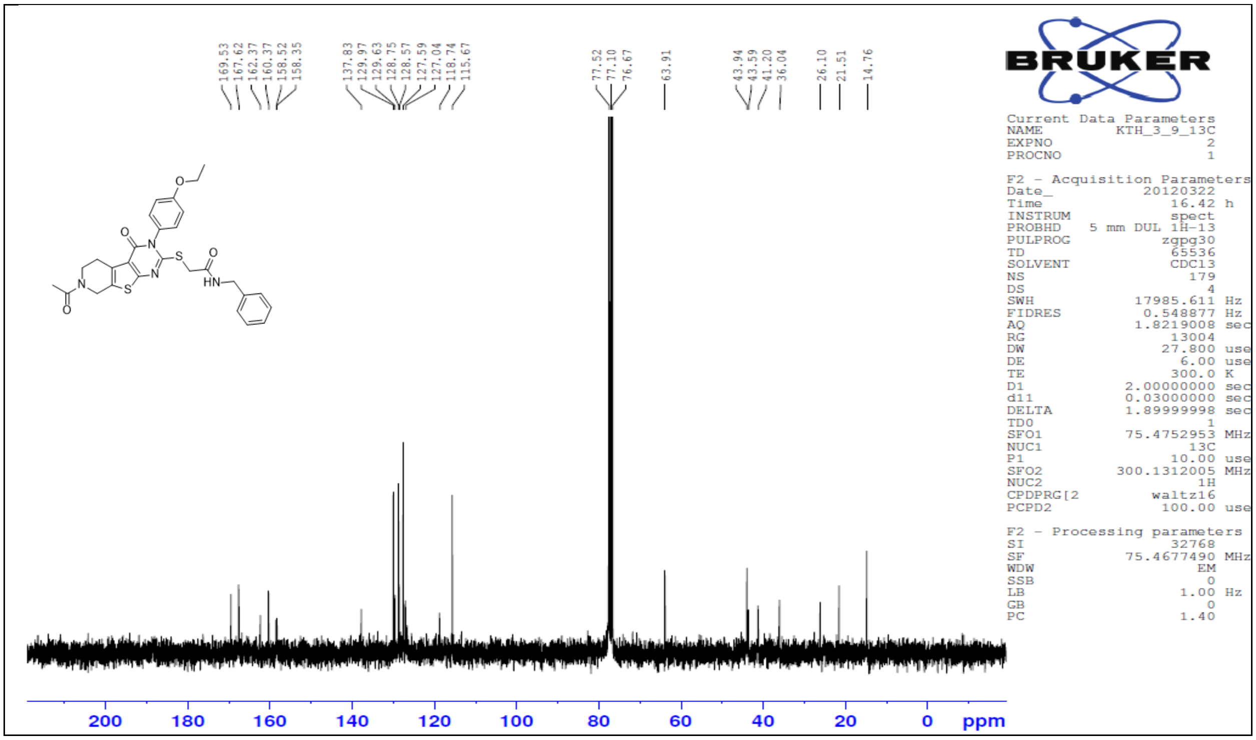

Figure S48. ^{1}H NMR Spectrum of **32** in $CDCl_3$

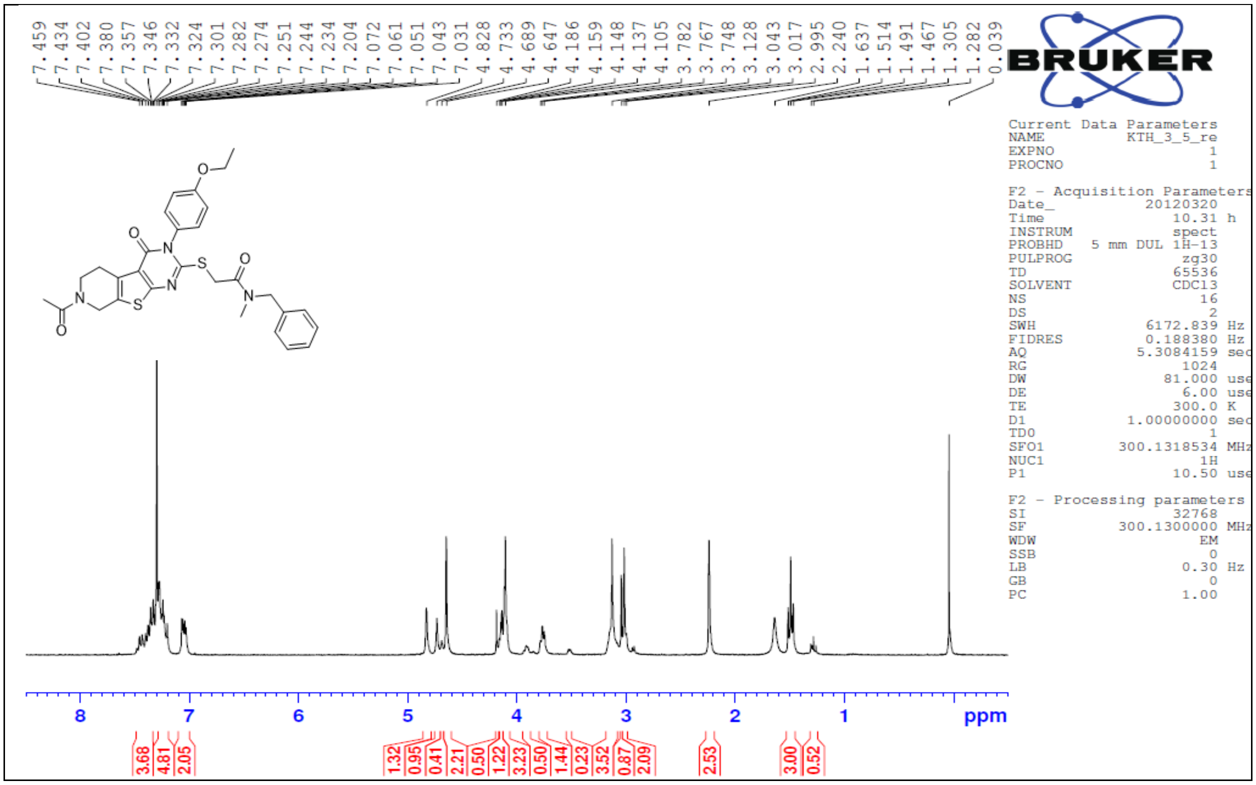

Figure S49. ^{13}C NMR Spectrum of **32** in $CDCl_3$

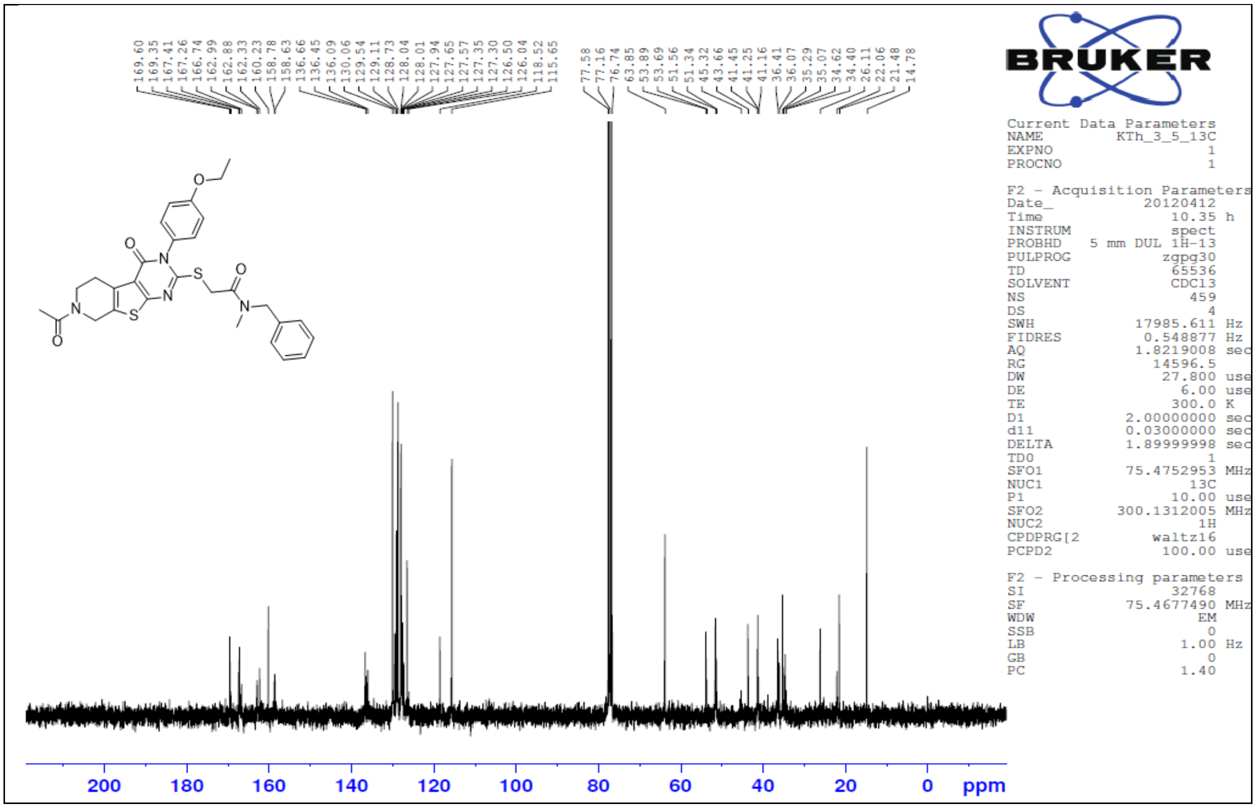

Figure S50. ^{1}H NMR Spectrum of **33** in $CDCl_3$

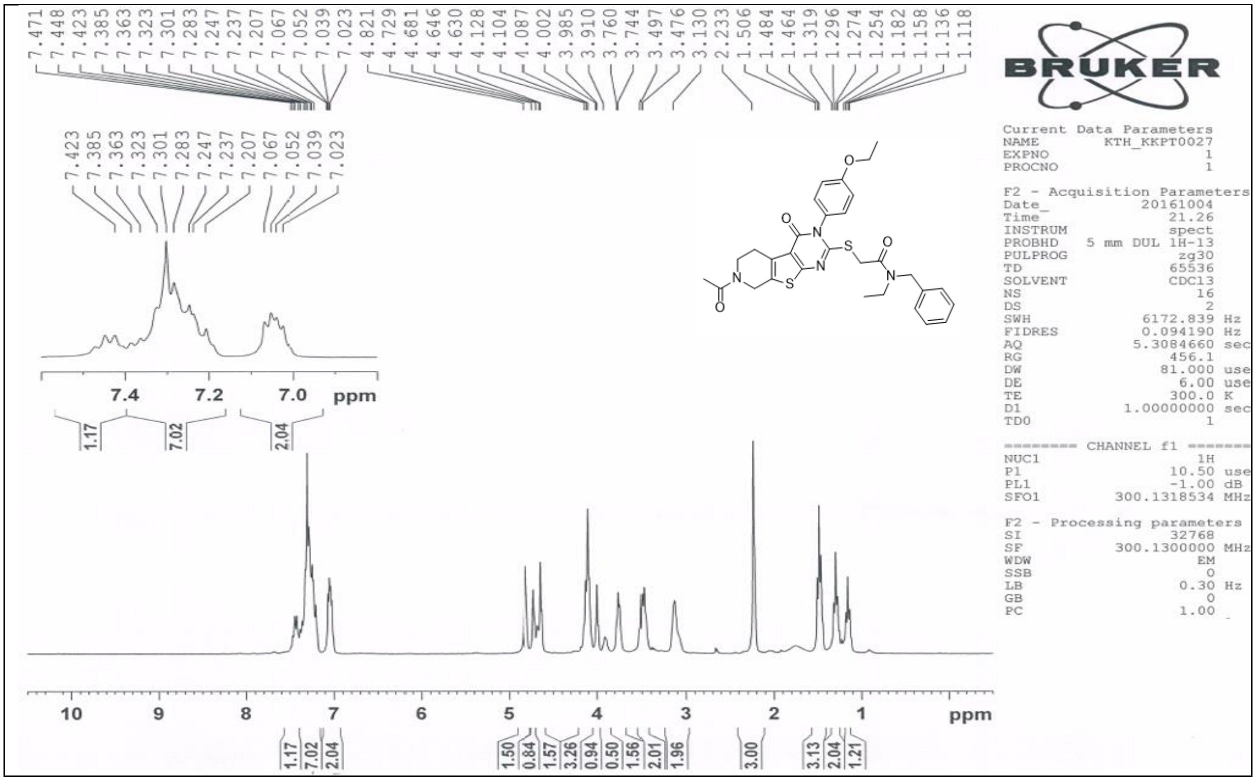

Figure S51. ^{13}C NMR Spectrum of **33** in $CDCl_3$

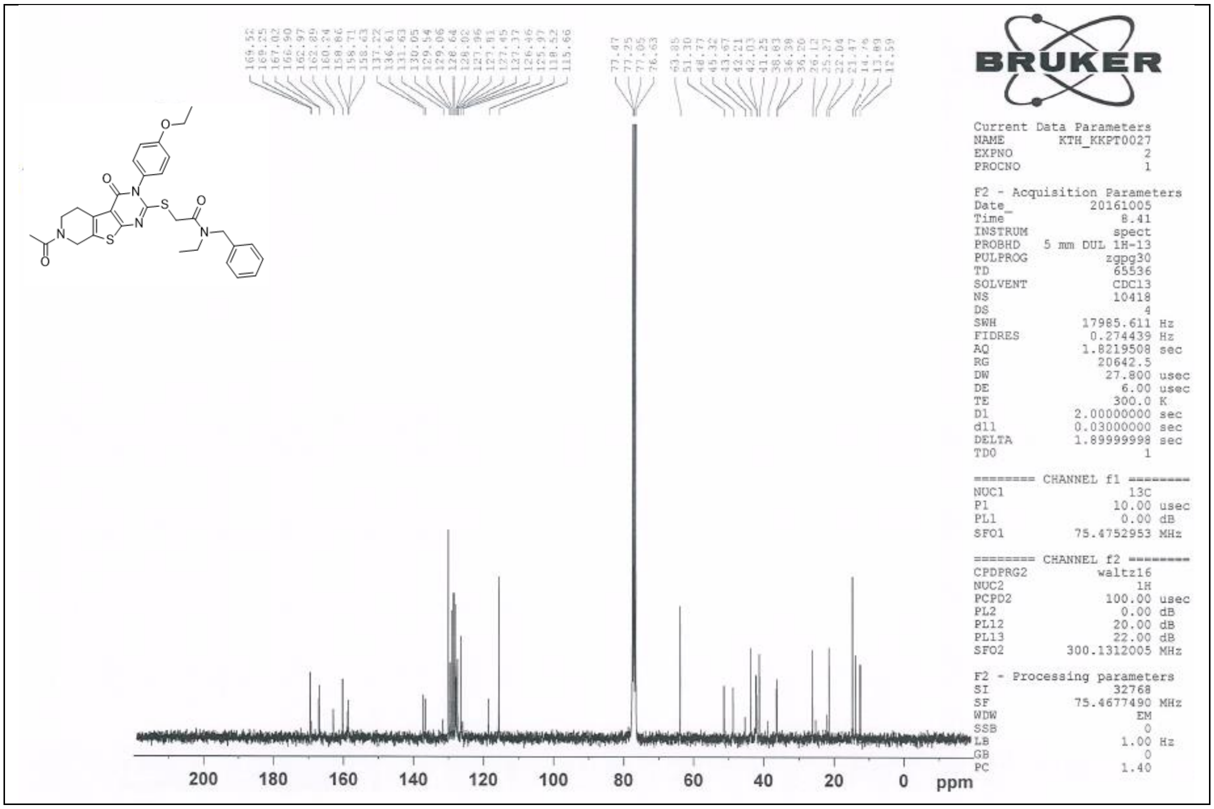

Figure S52. ^{1}H NMR Spectrum of **34** in $CDCl_3$

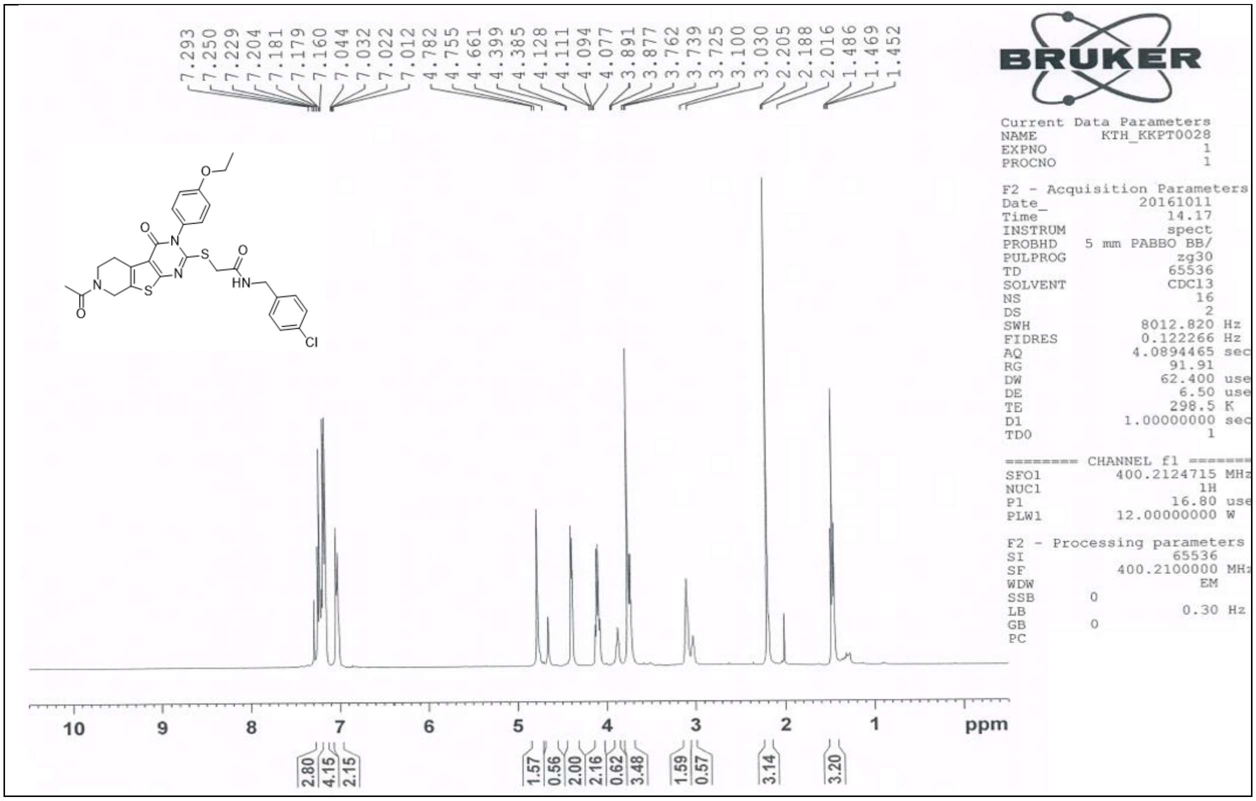

Figure S53. ^{13}C NMR Spectrum of **34** in $CDCl_3$

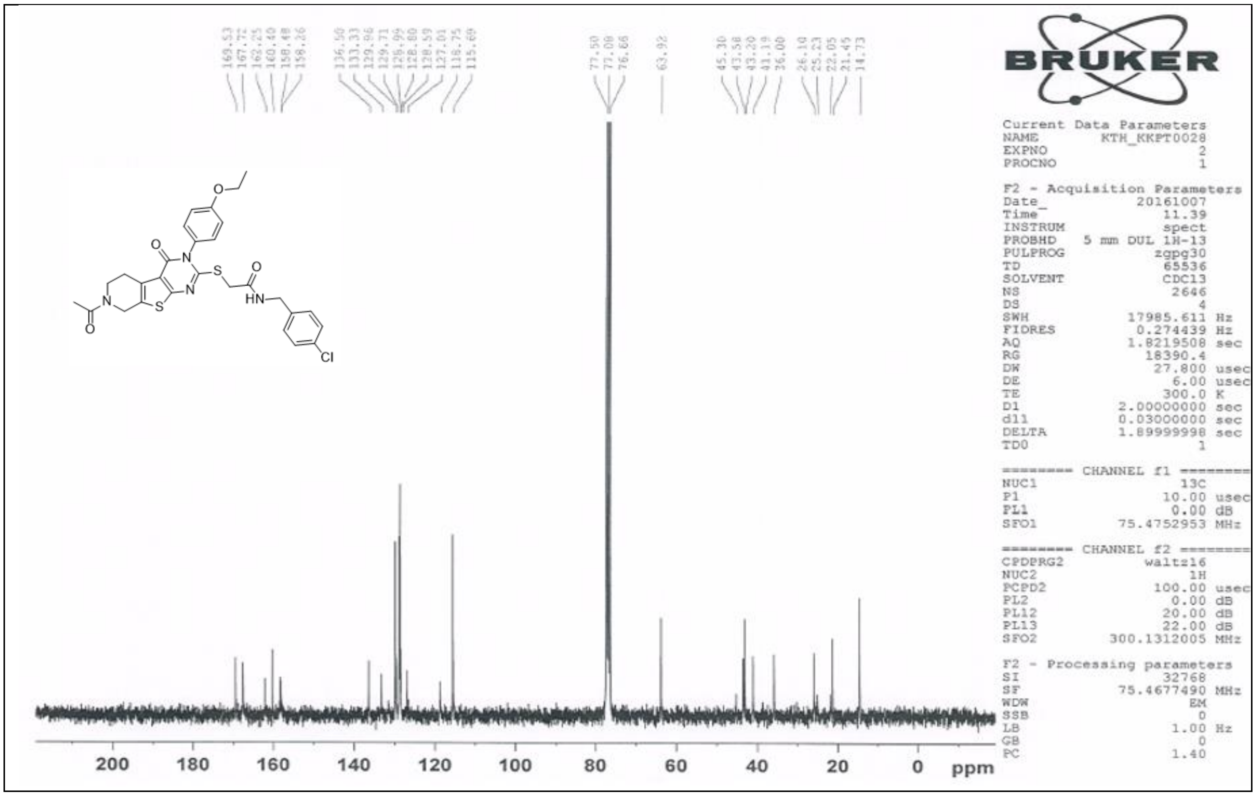

Figure S54. ^{1}H NMR Spectrum of **35** in $CDCl_3$

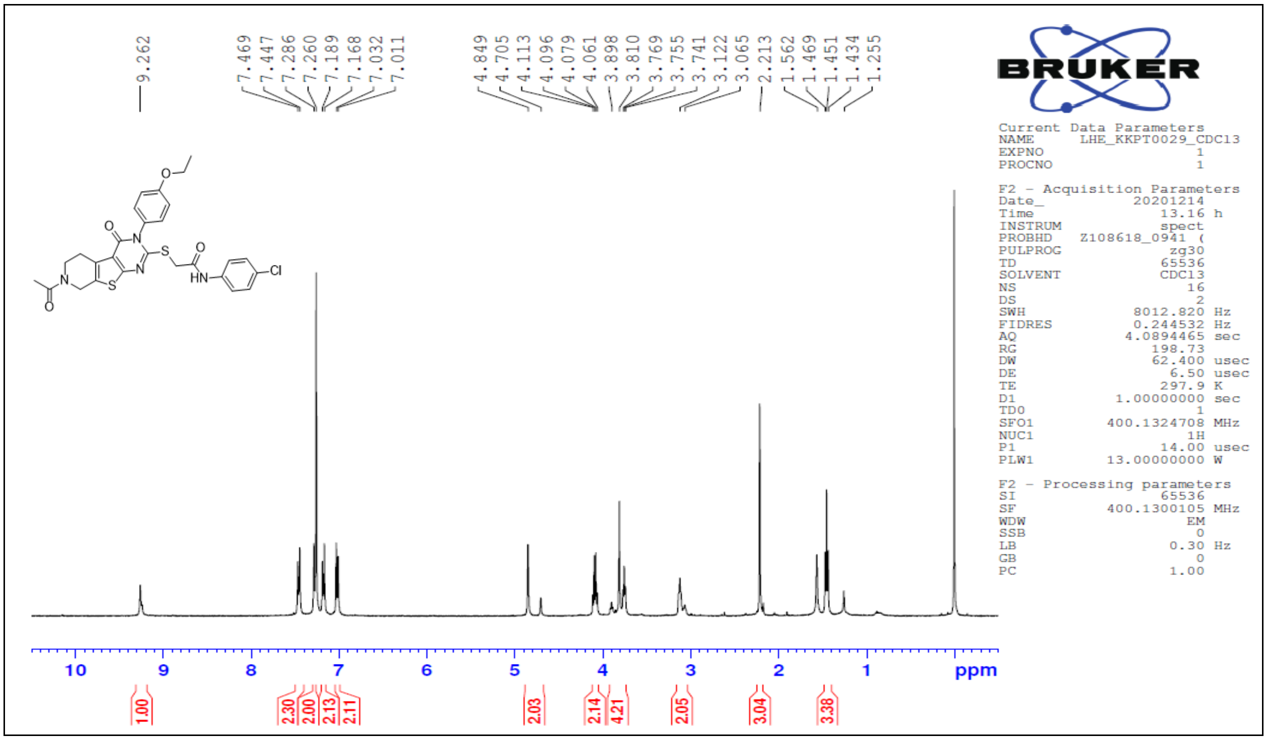

Figure S55. ^{13}C NMR Spectrum of **35** in $CDCl_3$

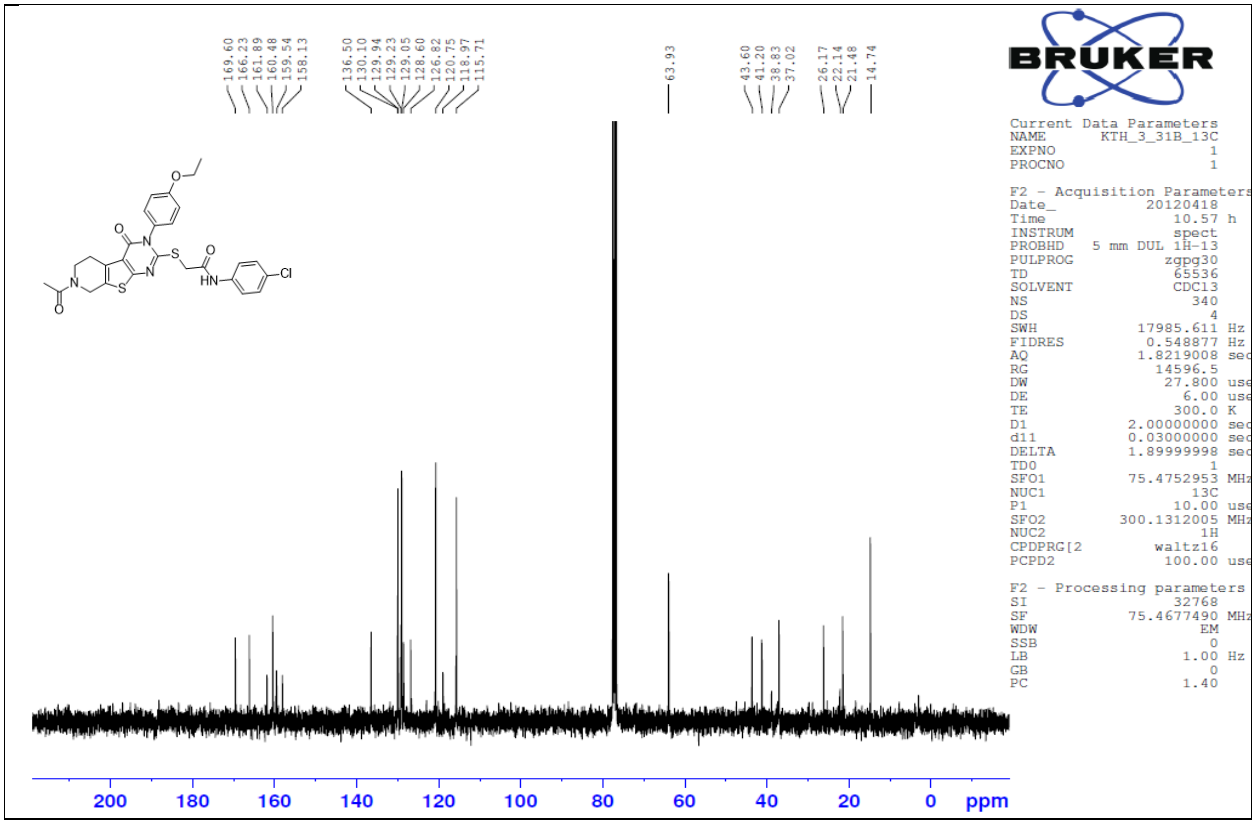

Figure S56. ^{1}H NMR Spectrum of **36** in $CDCl_3$

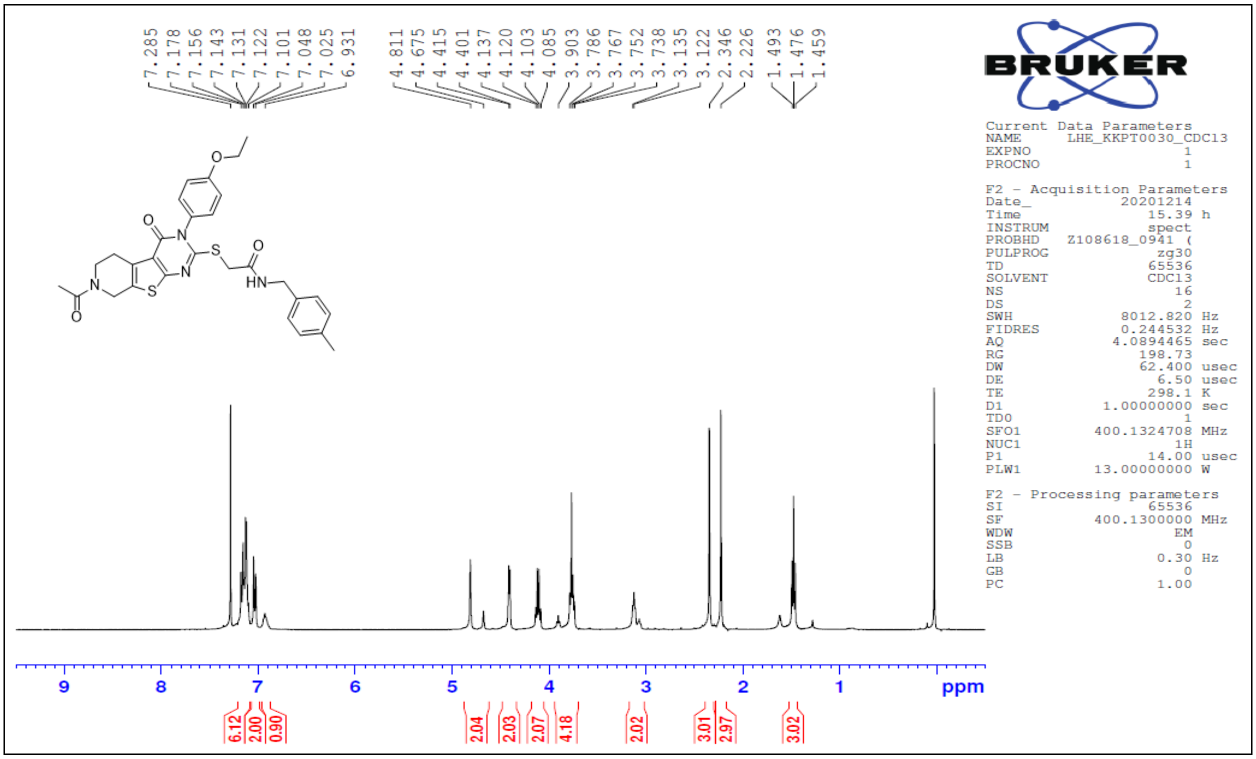

Figure S57. ^{13}C NMR Spectrum of **36** in $CDCl_3$

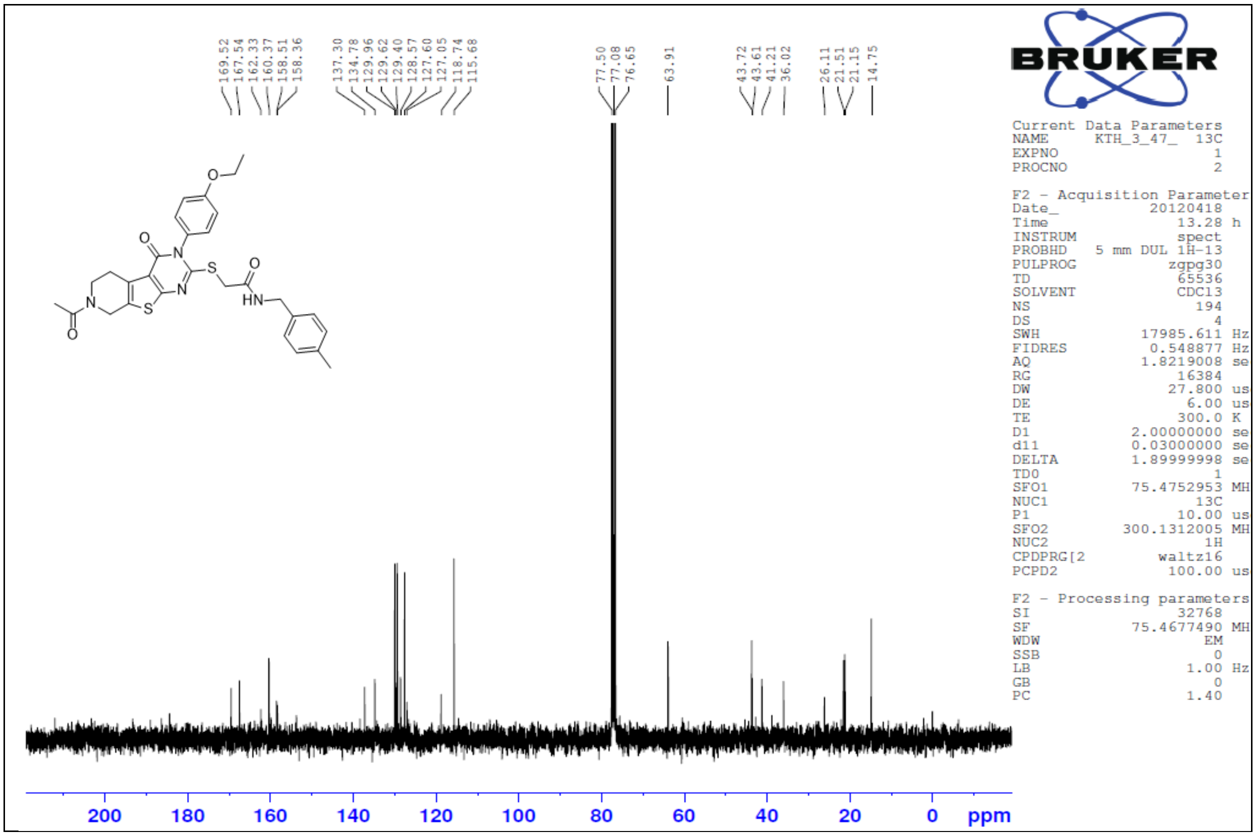

Figure S58. ^{1}H NMR Spectrum of **37** in $CDCl_3$

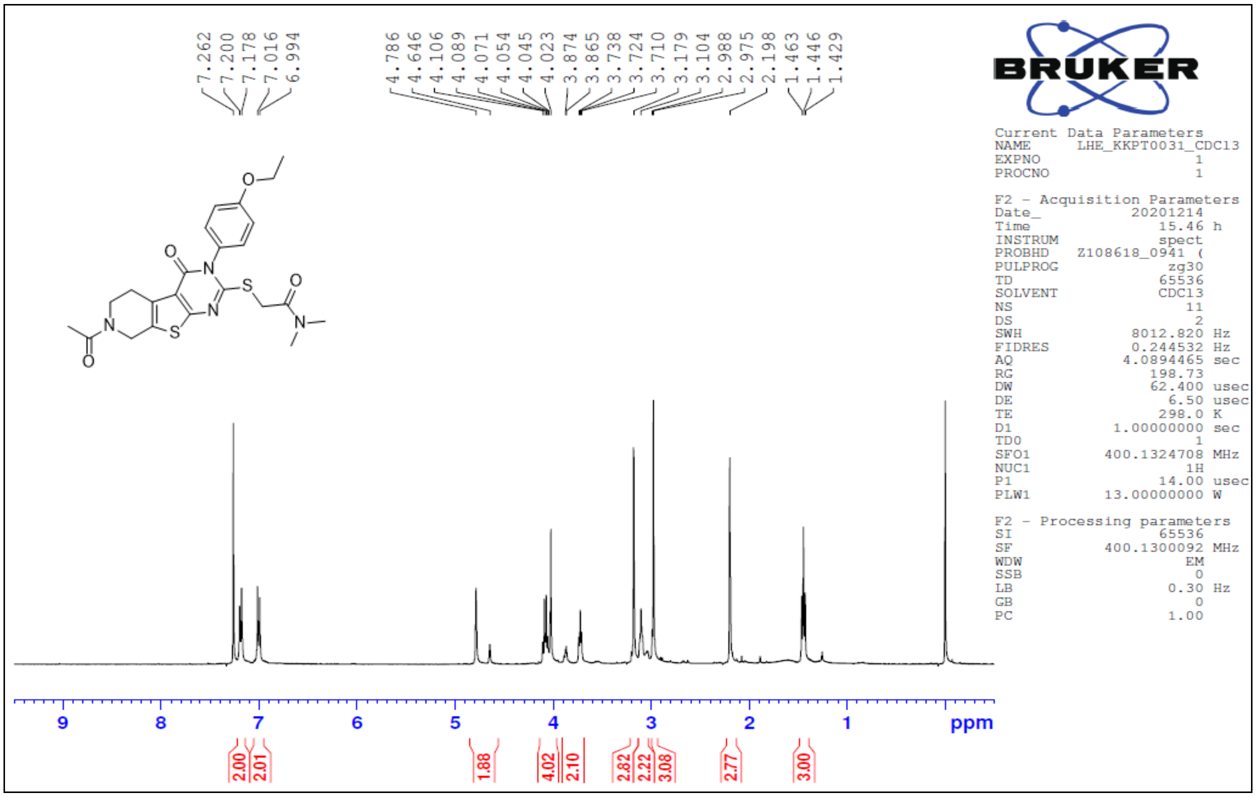

Figure S59. ^{13}C NMR Spectrum of **37** in $CDCl_3$

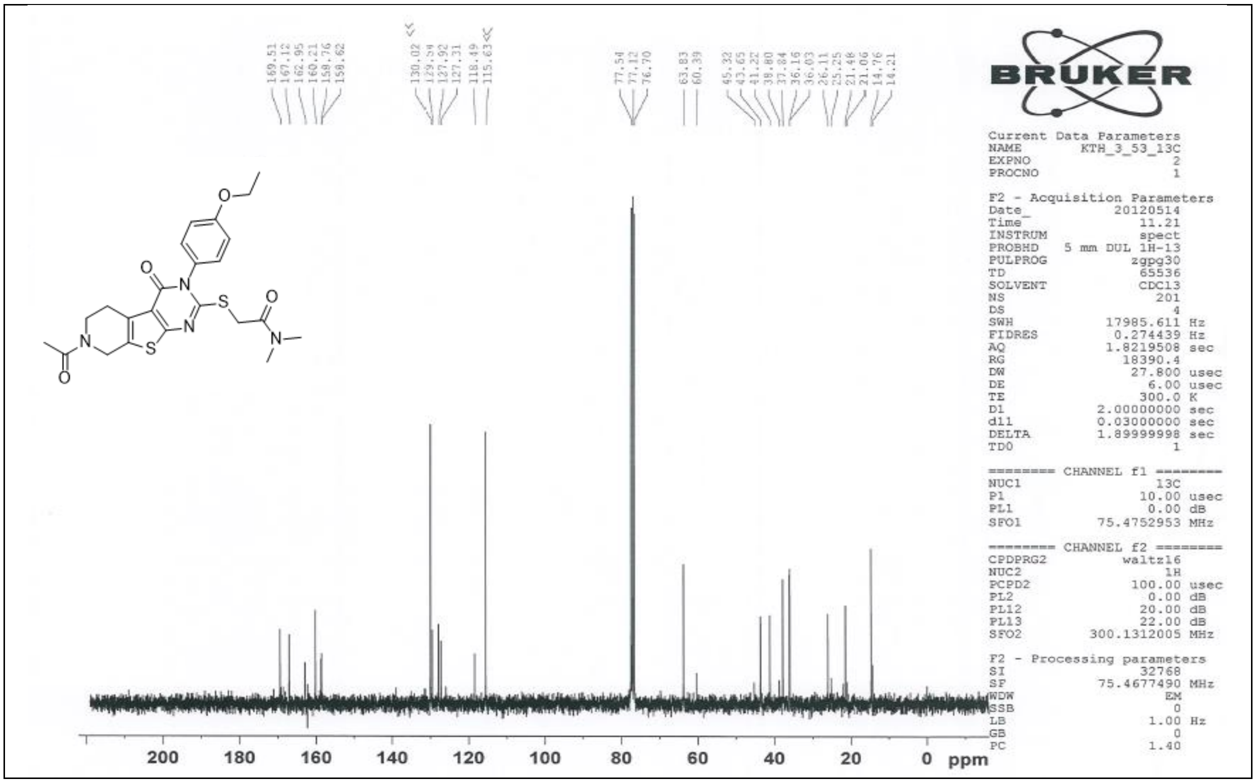

Figure S60. ^{1}H NMR Spectrum of **38** in $CDCl_3$

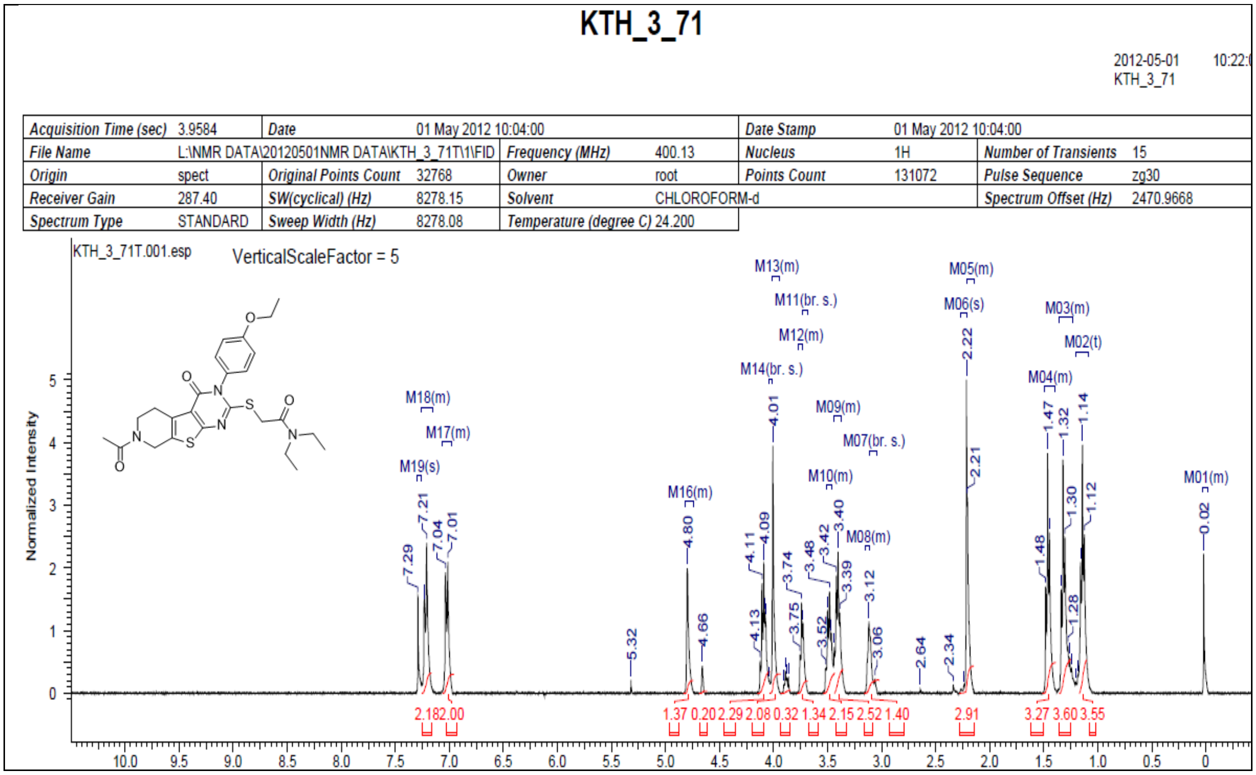

Figure S61. ^{13}C NMR Spectrum of **38** in $CDCl_3$

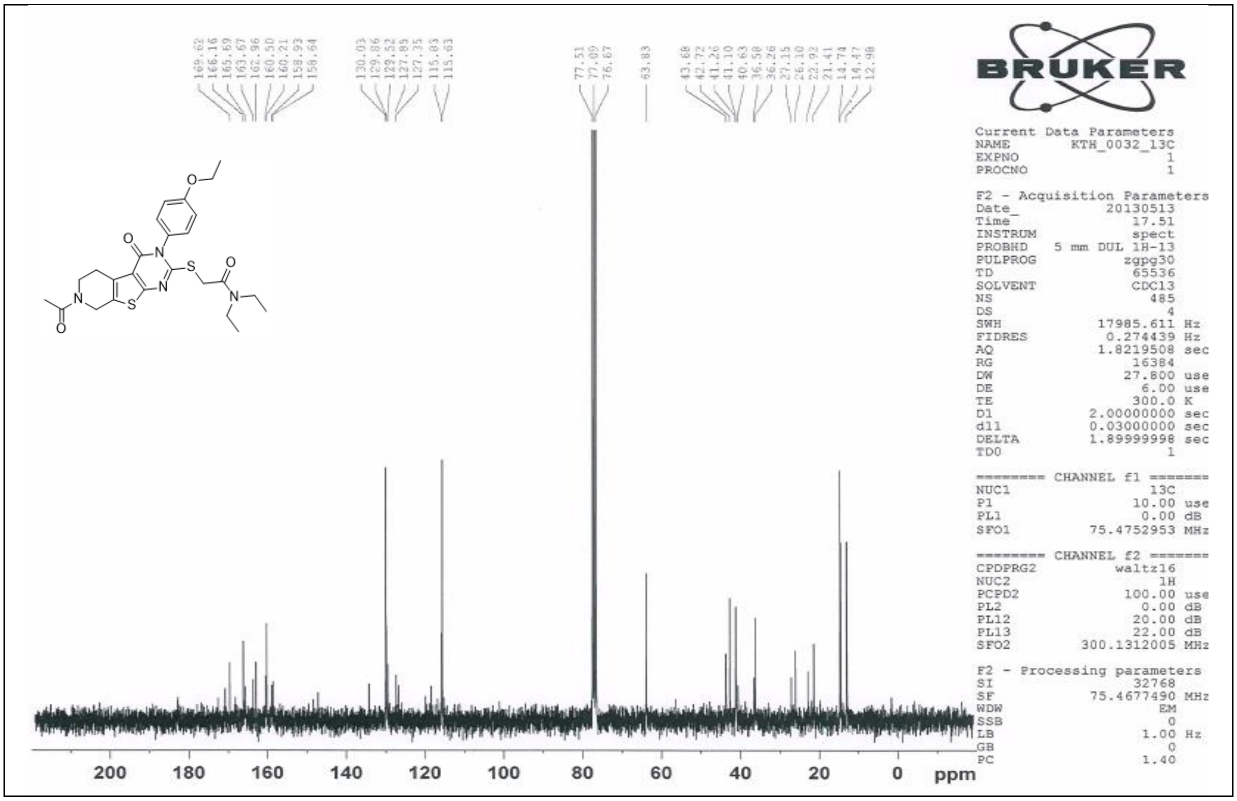

Figure S62. ^{1}H NMR Spectrum of **39** in $CDCl_3$

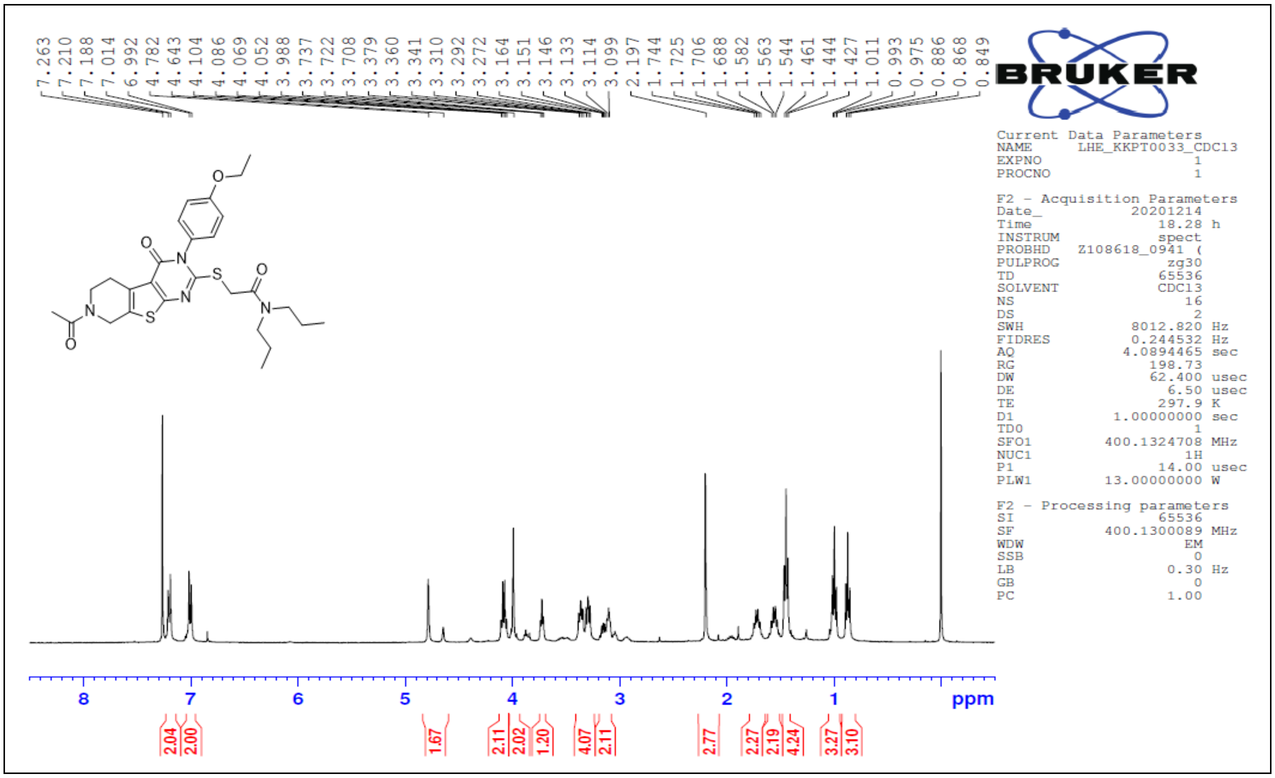

Figure S63. ^{13}C NMR Spectrum of **39** in $CDCl_3$

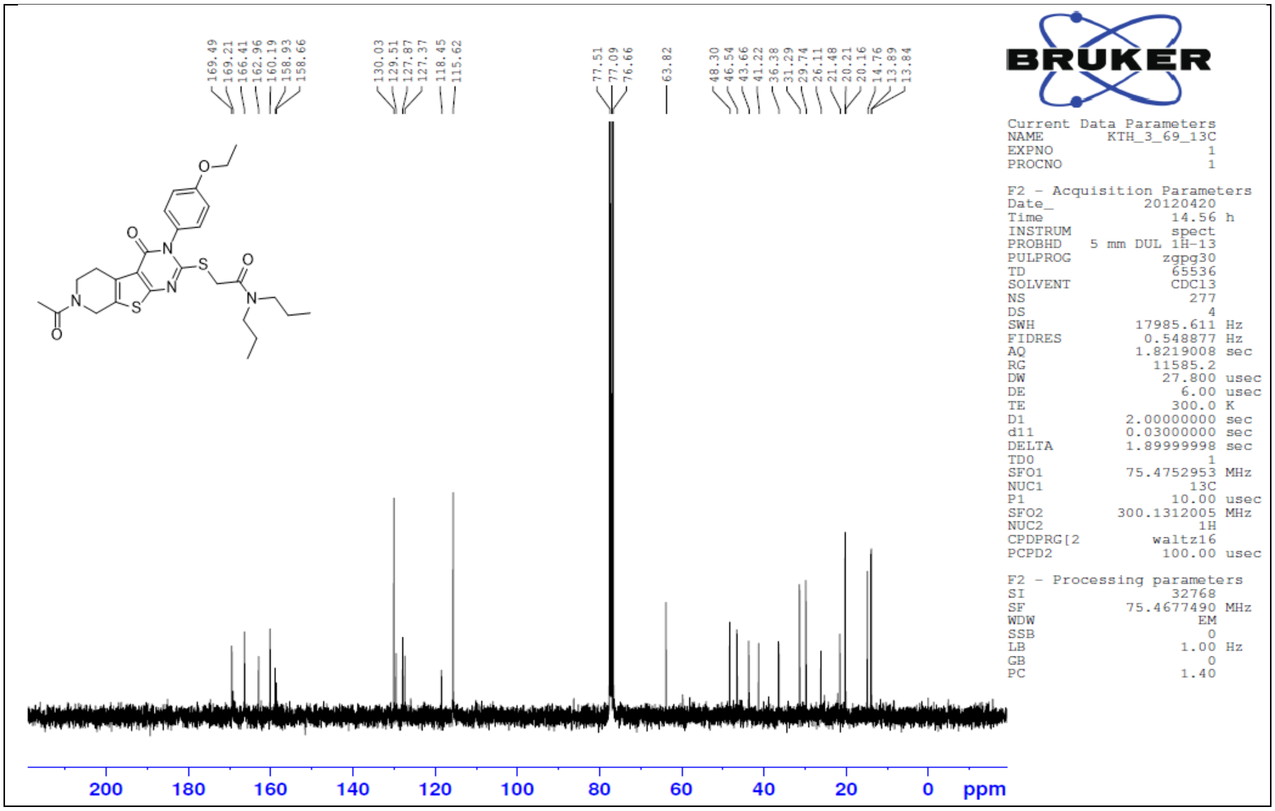

Figure S64. ^{1}H NMR Spectrum of **40** in $CDCl_3$

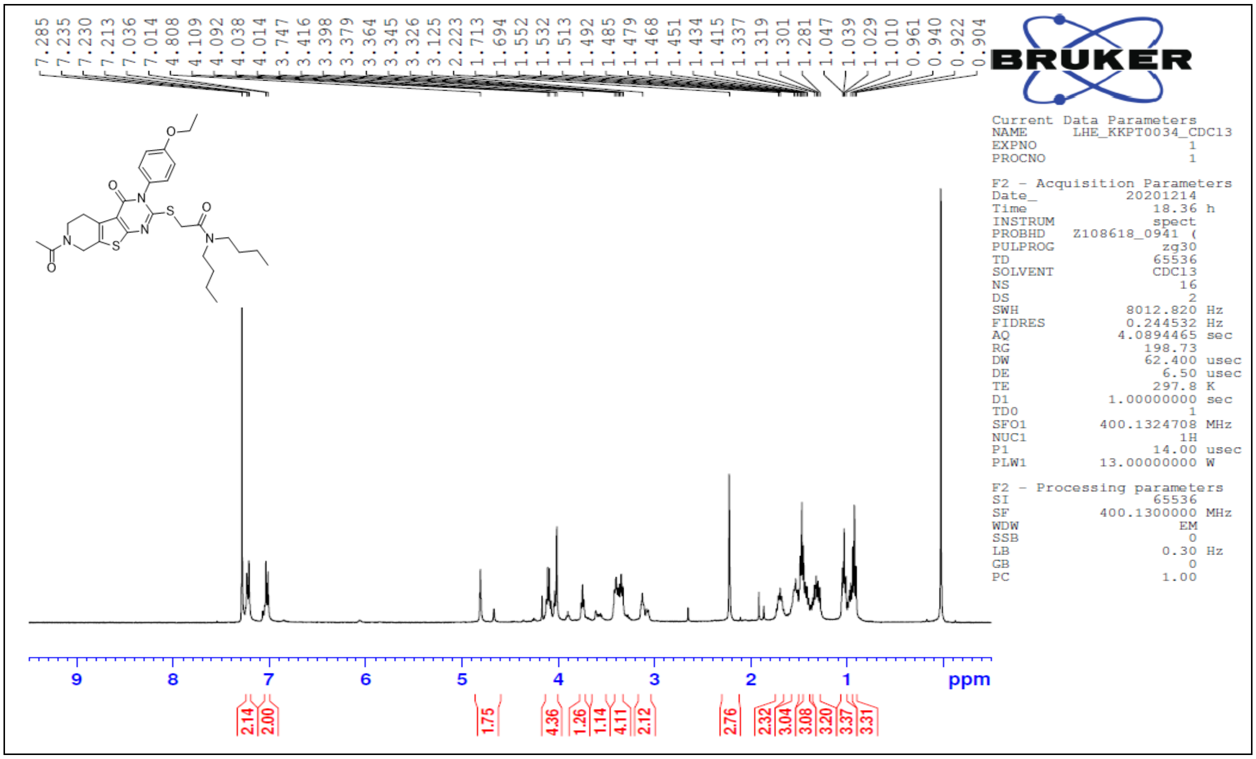

Figure S65. ^{13}C NMR Spectrum of **40** in $CDCl_3$

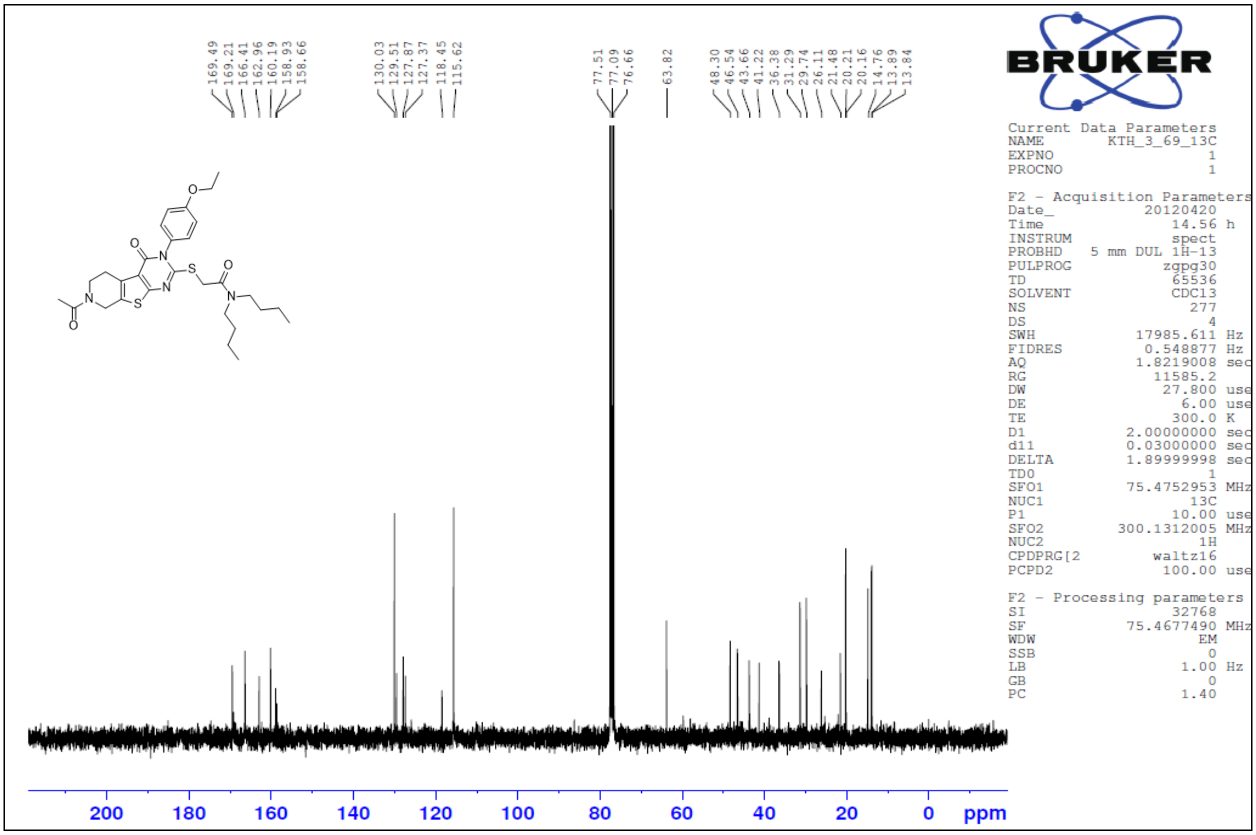

Figure S66. ^{1}H NMR Spectrum of **41** in CDCl3

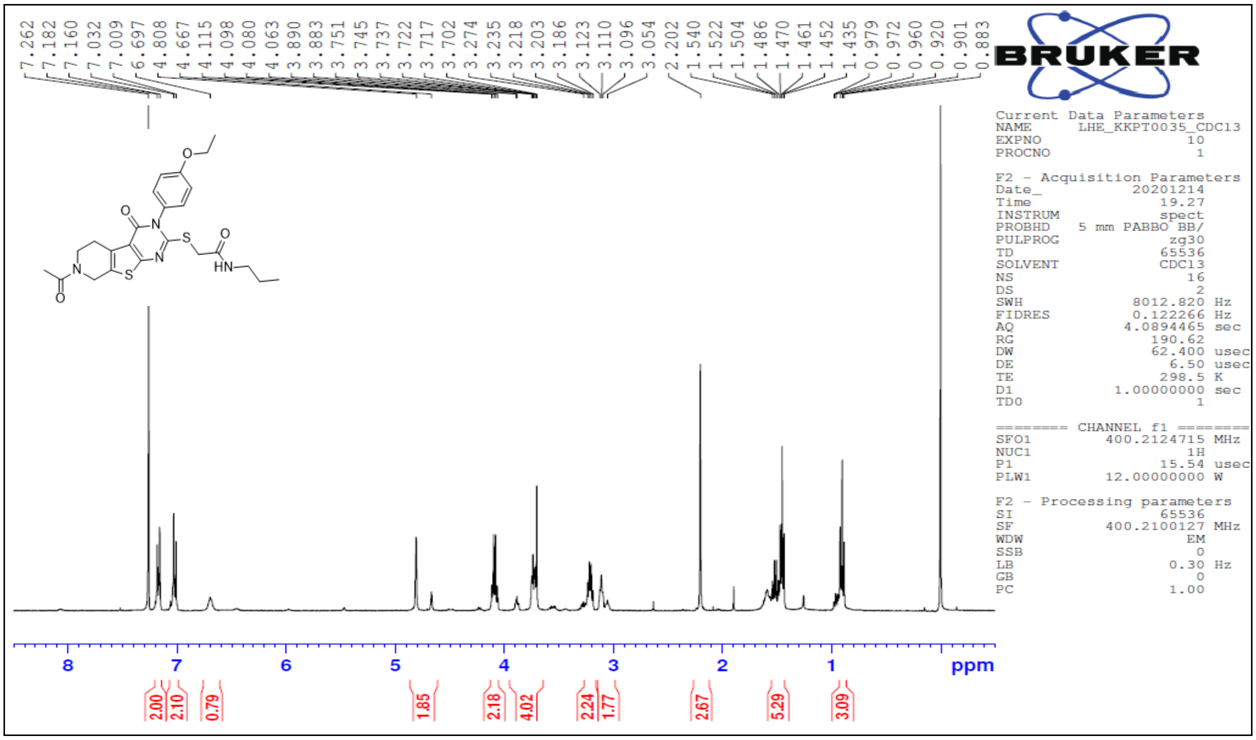

Figure S67. ^{13}C NMR Spectrum of **41** in CDCl3

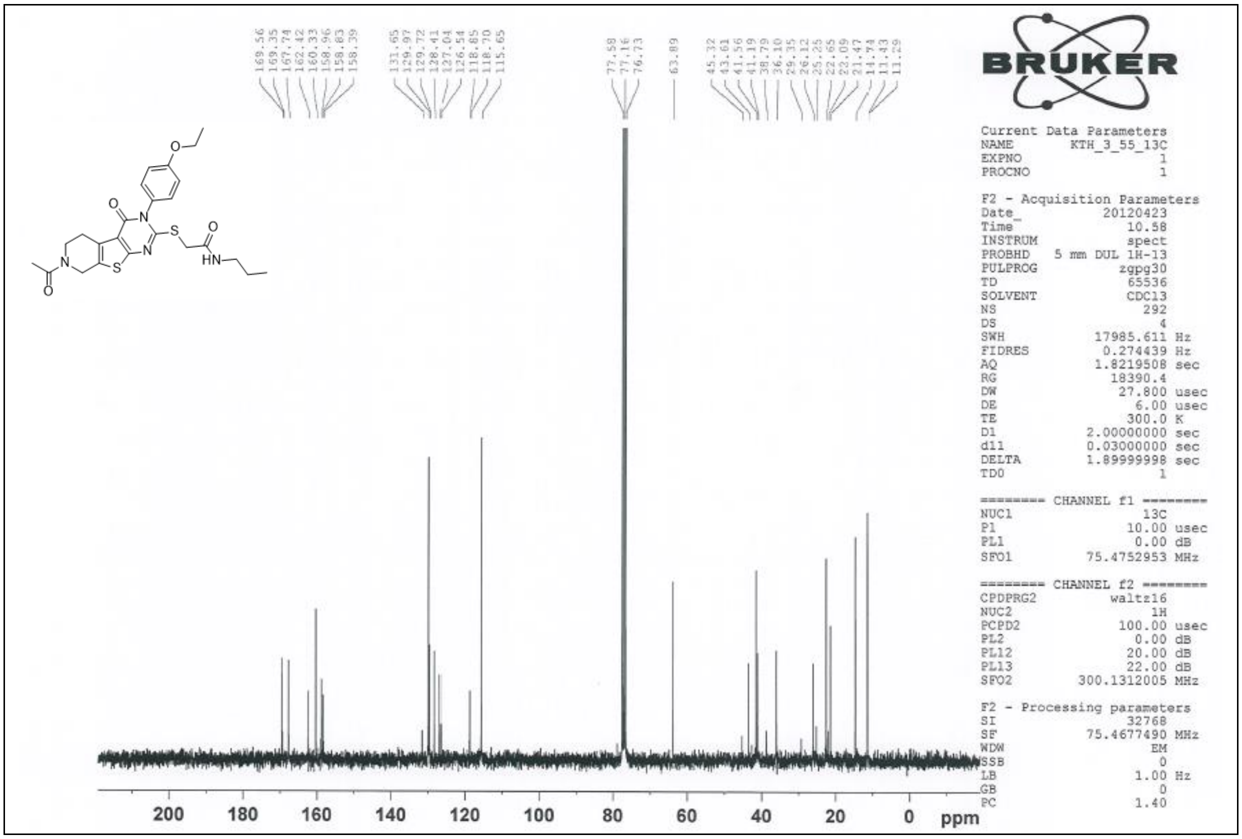

Figure S68. ^{1}H NMR Spectrum of **42** in $CDCl_3$

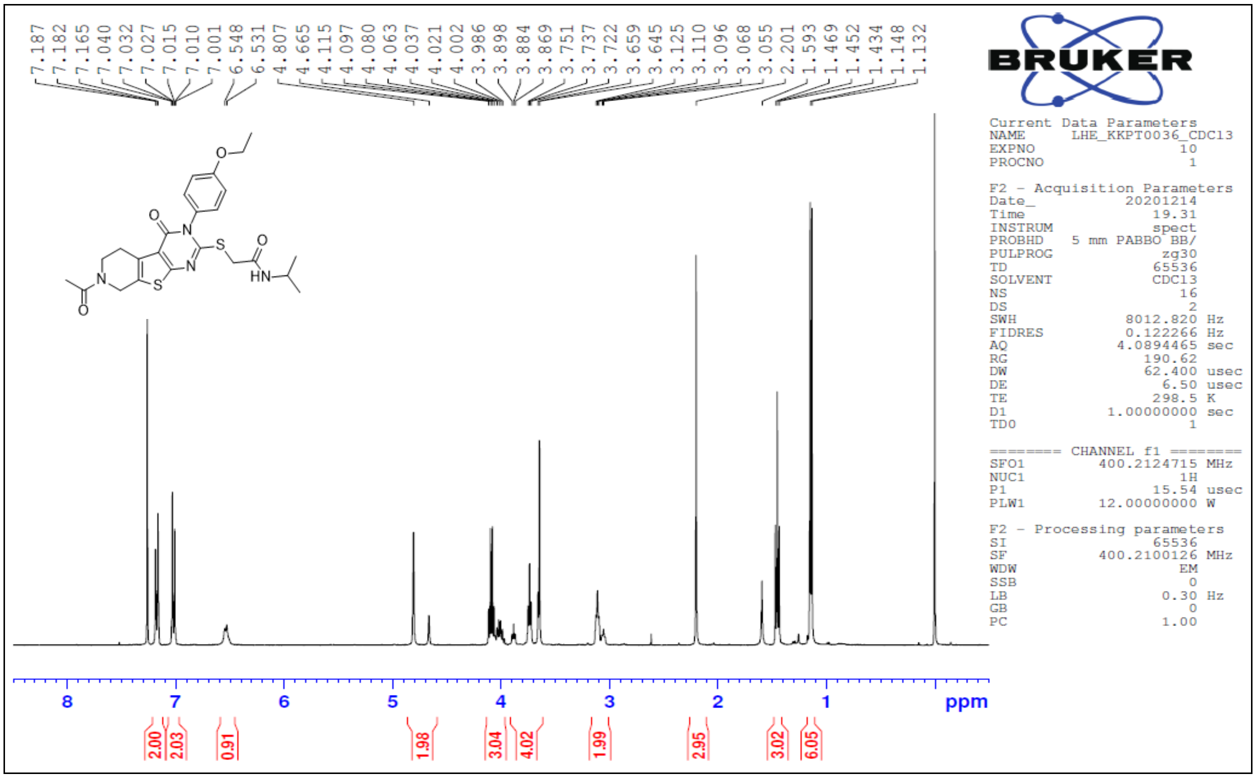

Figure S69. ^{13}C NMR Spectrum of **42** in $CDCl_3$

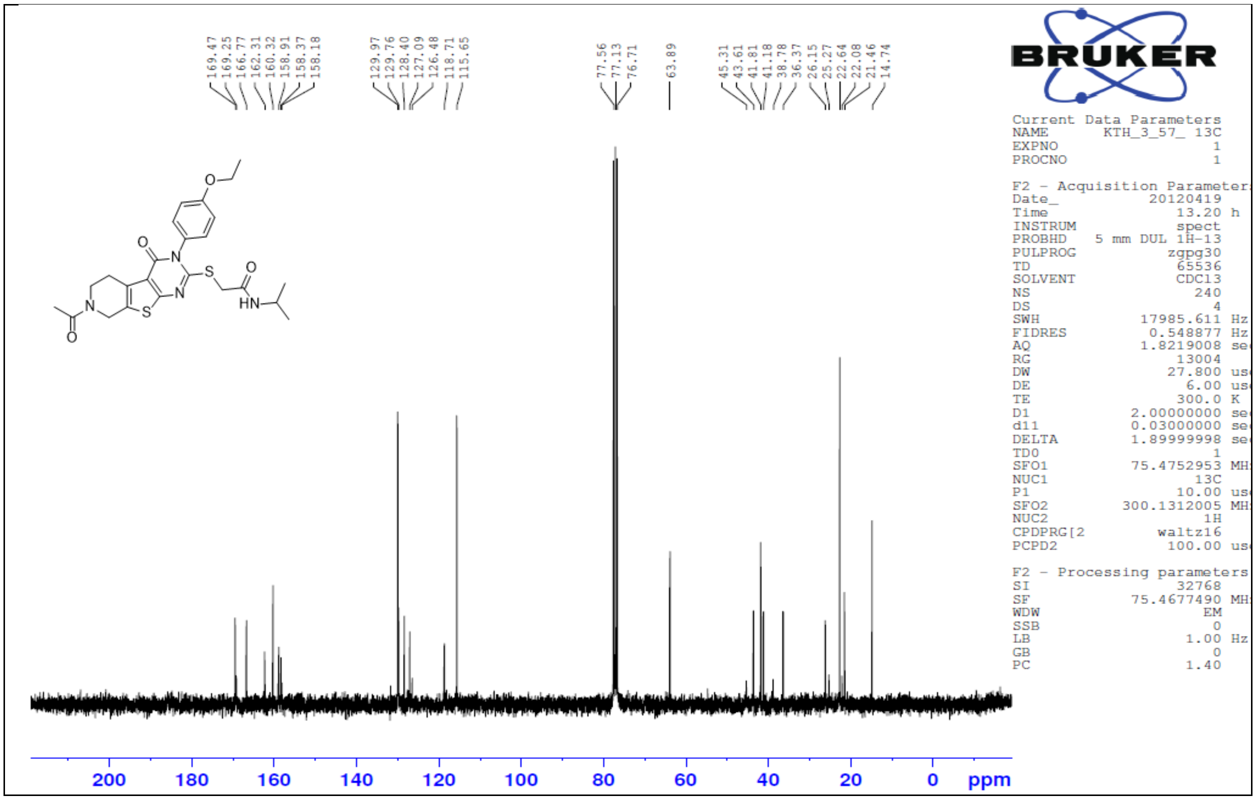

Figure S70. ^{1}H NMR Spectrum of **43** in $CDCl_3$

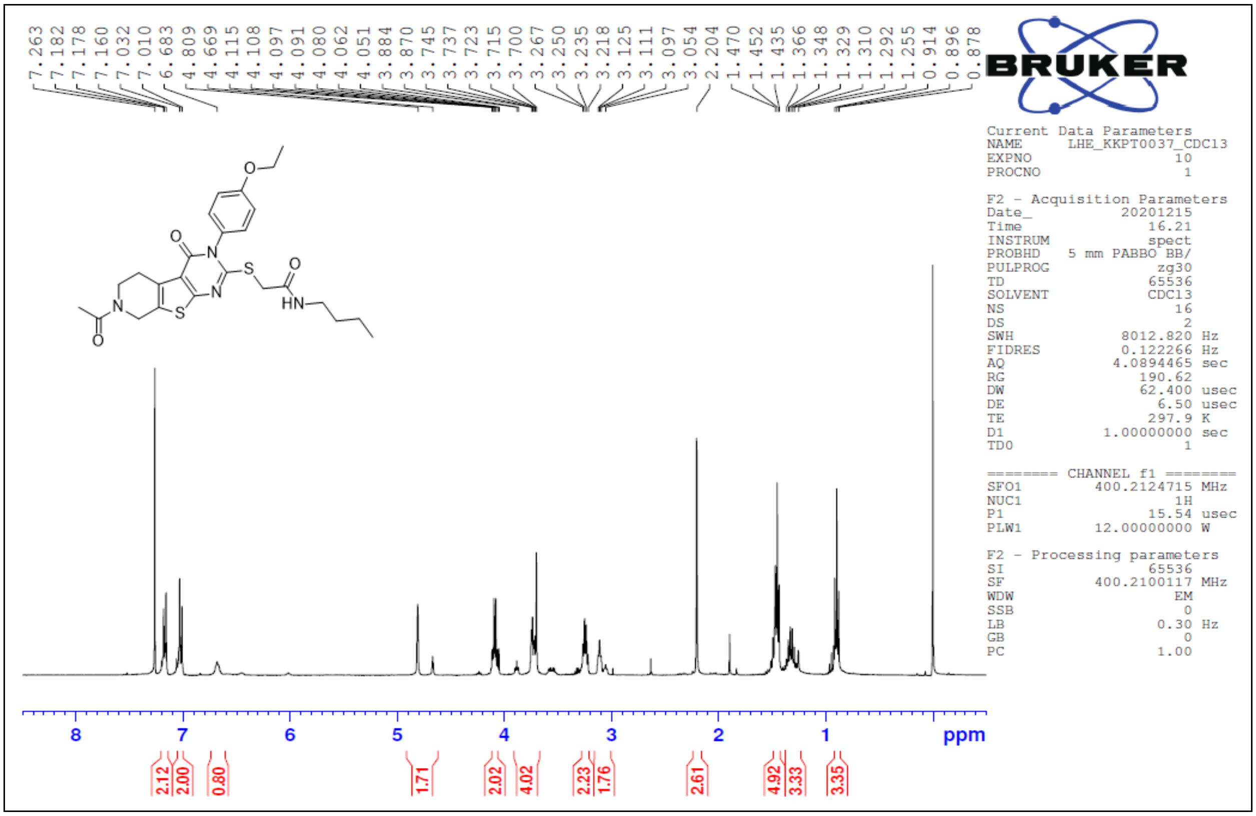

Figure S71. ^{13}C NMR Spectrum of **43** in $CDCl_3$

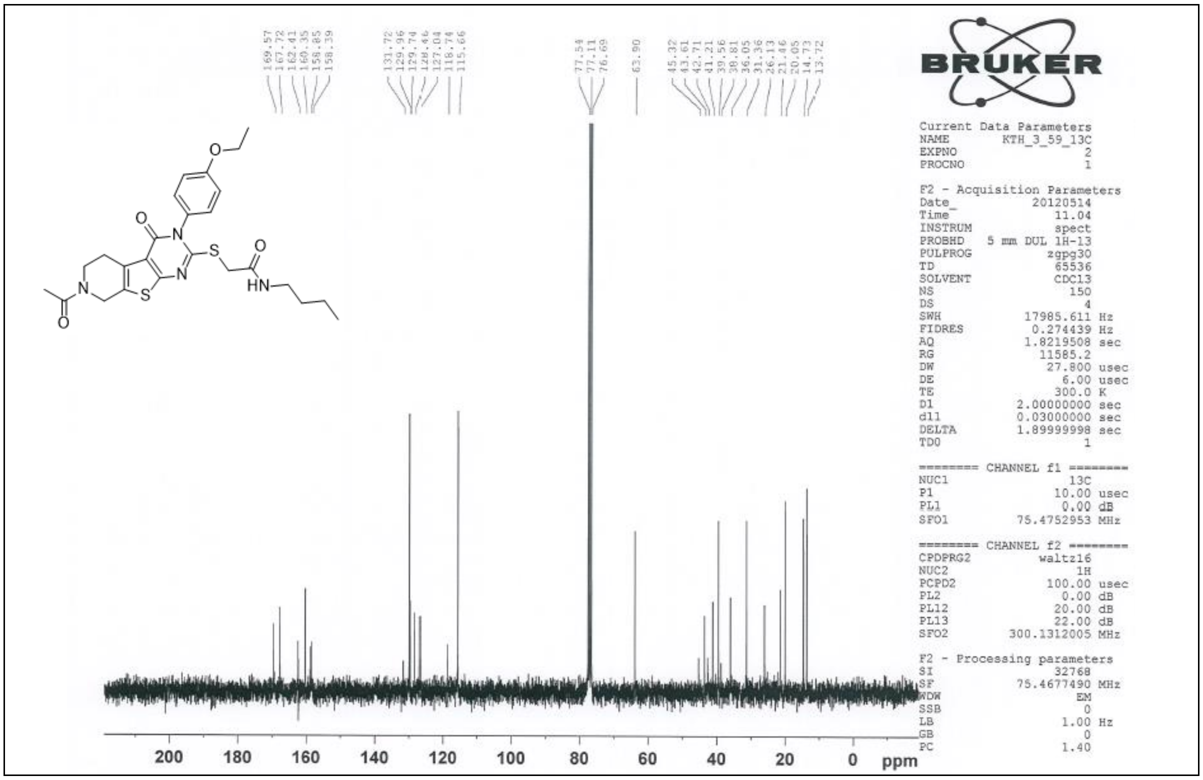

Figure S72. ^{1}H NMR Spectrum of **44** in $CDCl_3$

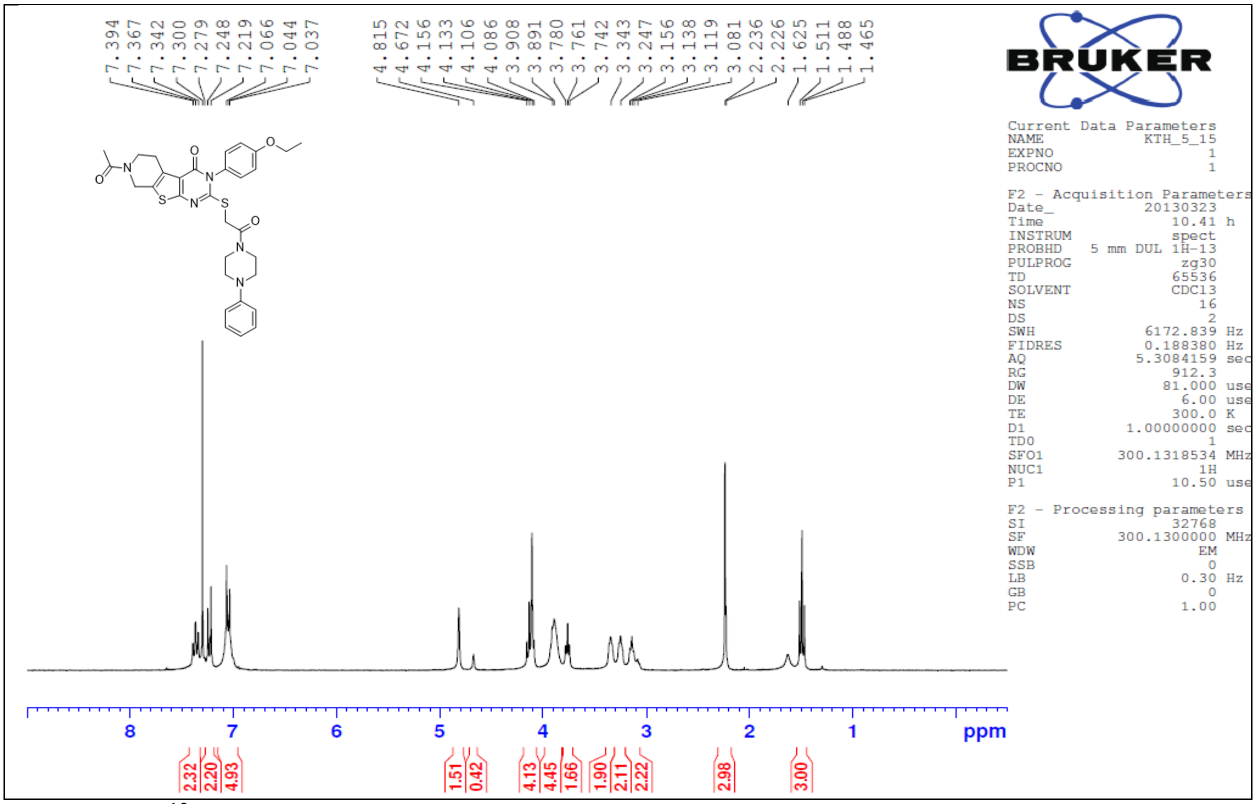

Figure S73. ^{13}C NMR Spectrum of **44** in $CDCl_3$

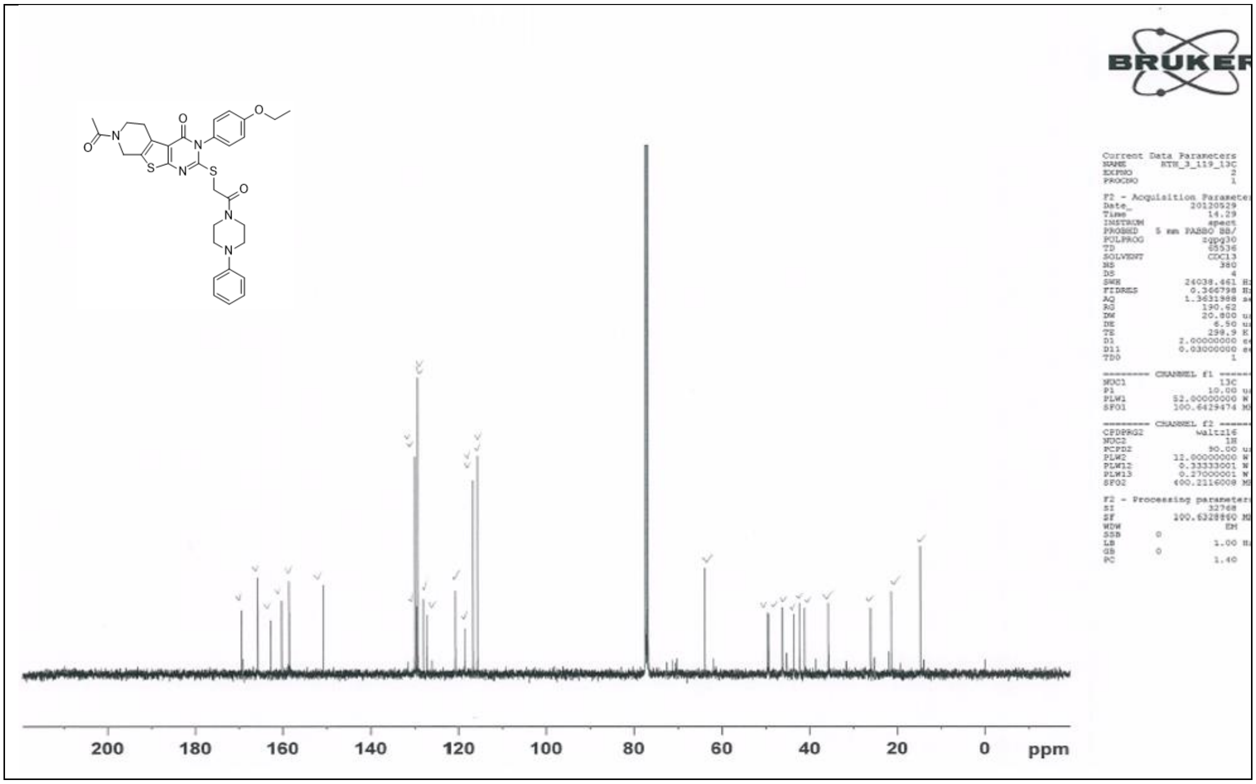

Figure S74. ^{1}H NMR Spectrum of **45** in $CDCl_3$

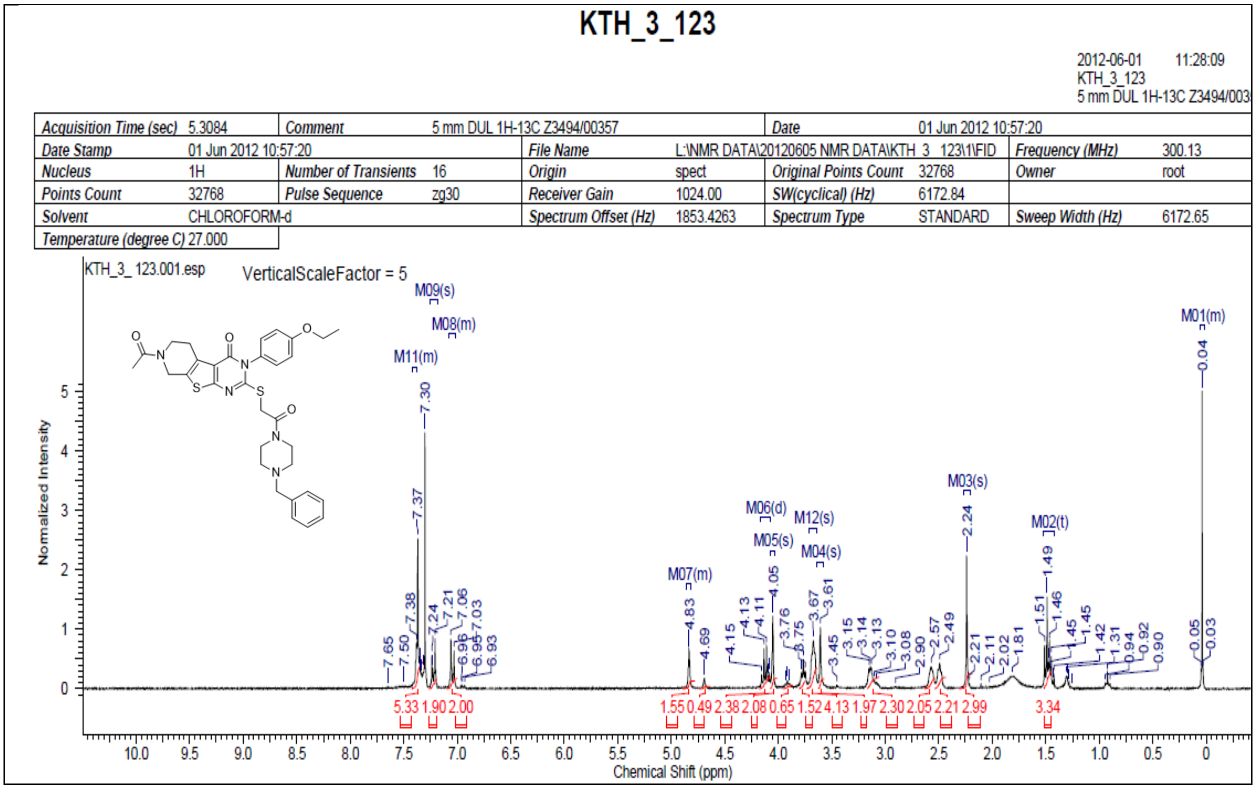

Figure S75. ^{13}C NMR Spectrum of **45** in $CDCl_3$

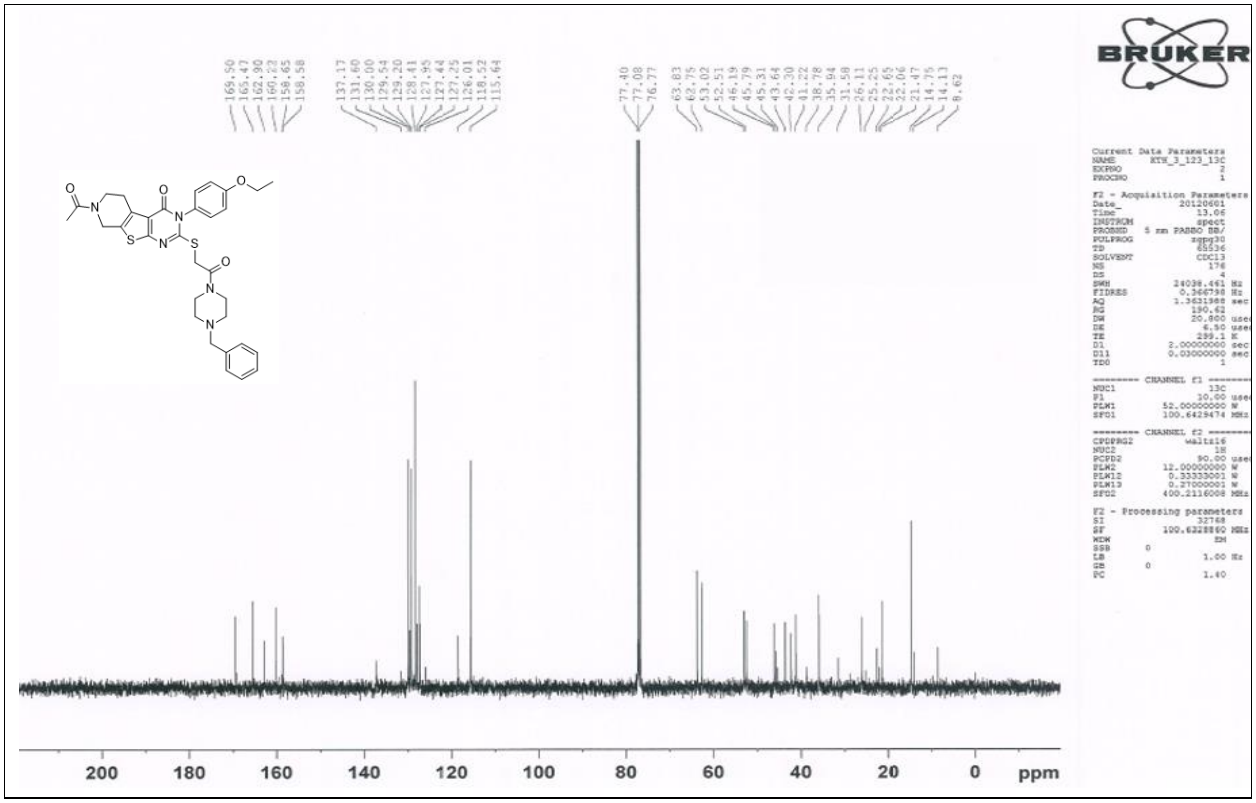

Figure S76. ^{1}H NMR Spectrum of **46** in $CDCl_3$

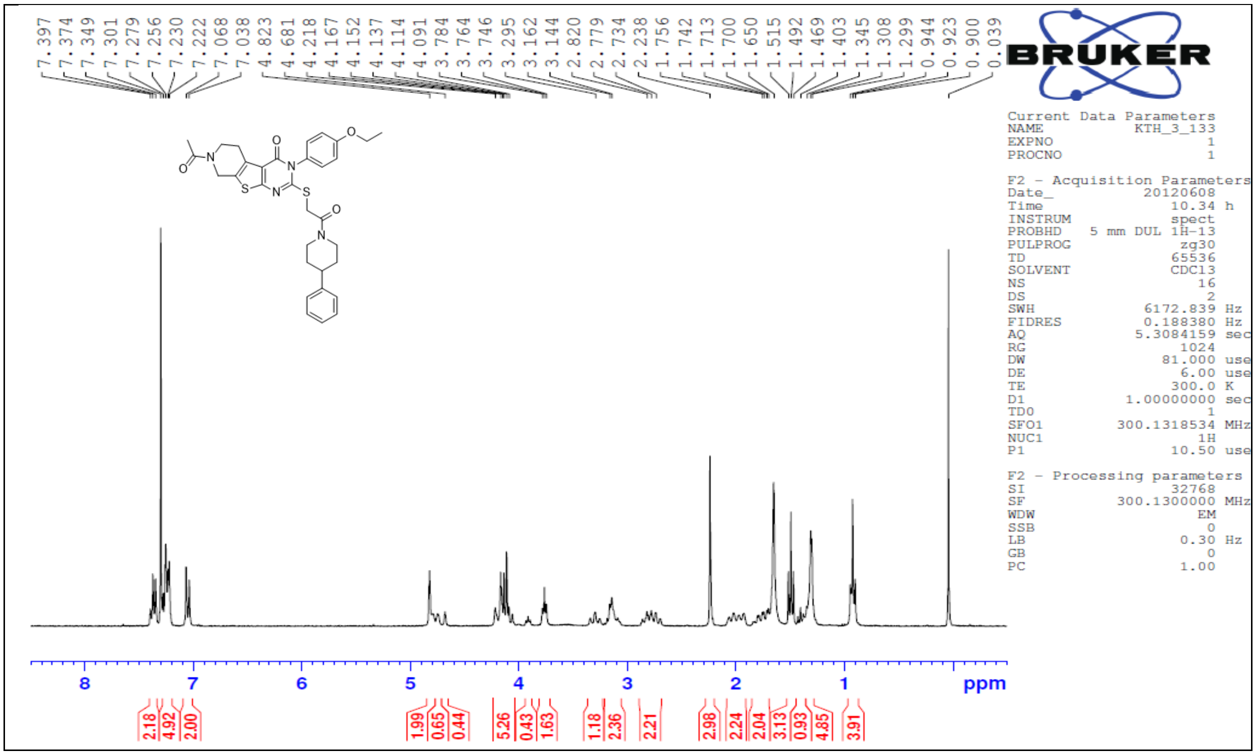

Figure S77. ^{13}C NMR Spectrum of **46** in $CDCl_3$

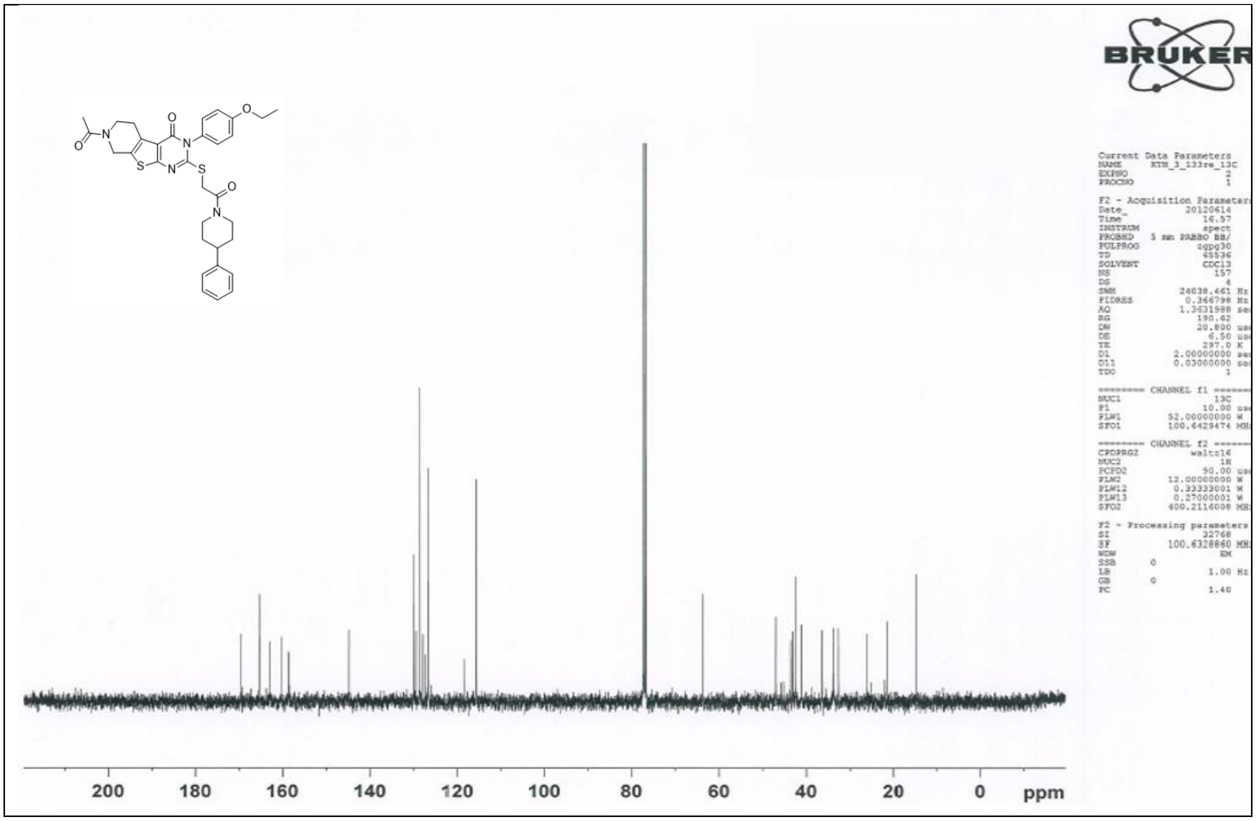

Figure S78. ^{1}H NMR Spectrum of **47** in $CDCl_3$

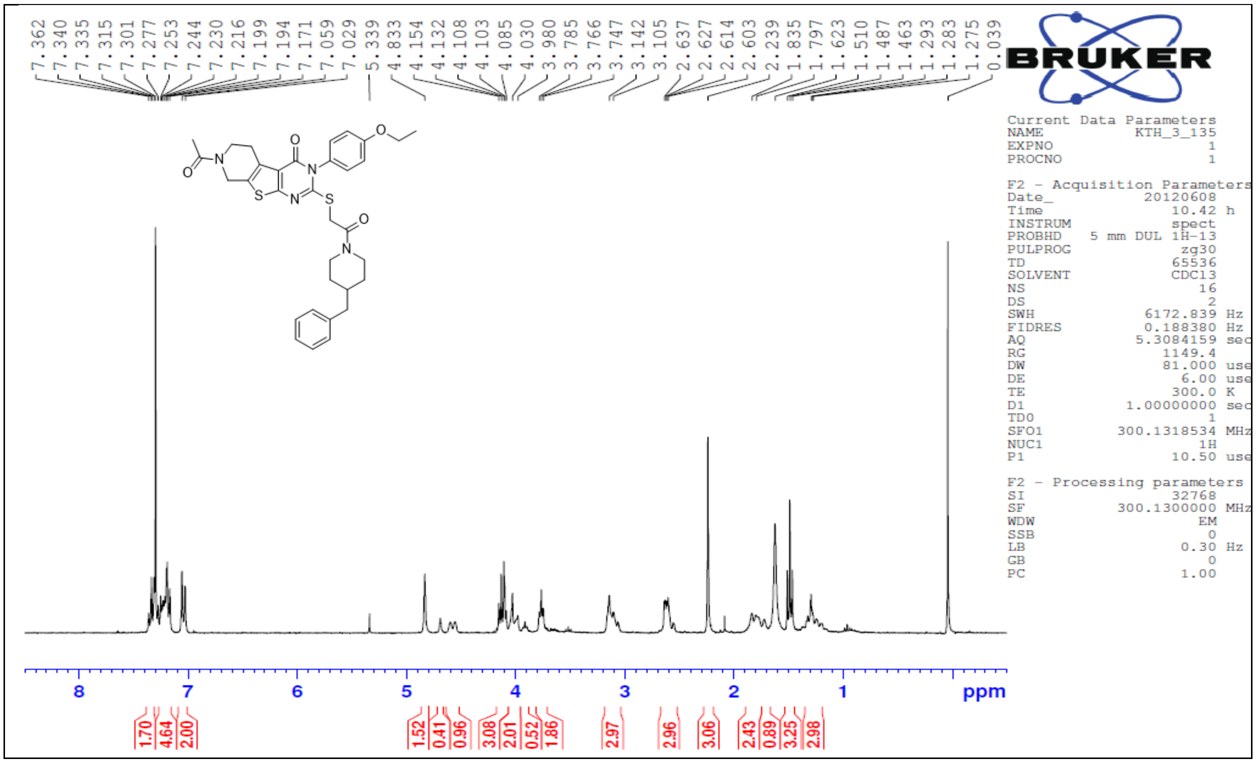

Figure S79. ^{13}C NMR Spectrum of **47** in $CDCl_3$

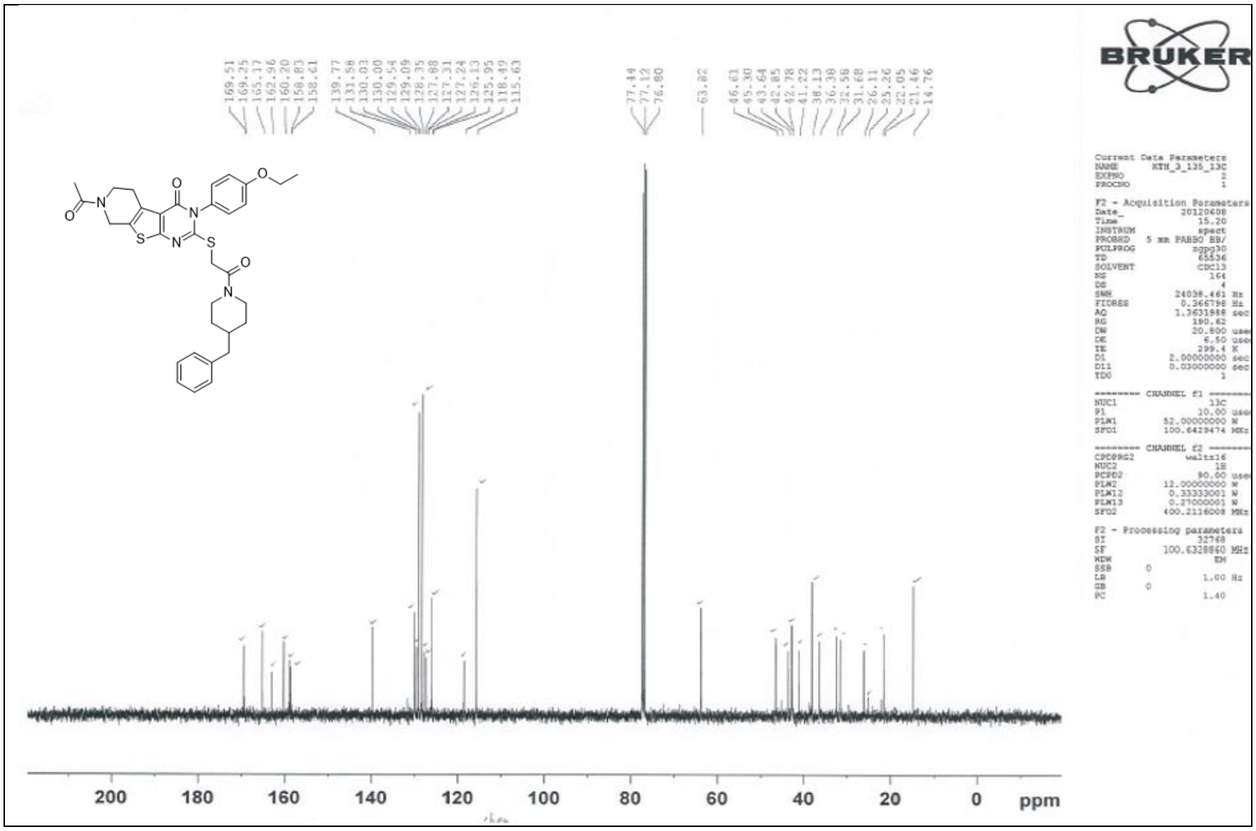

Figure S80. ^{1}H NMR Spectrum of **48** in $CDCl_3$

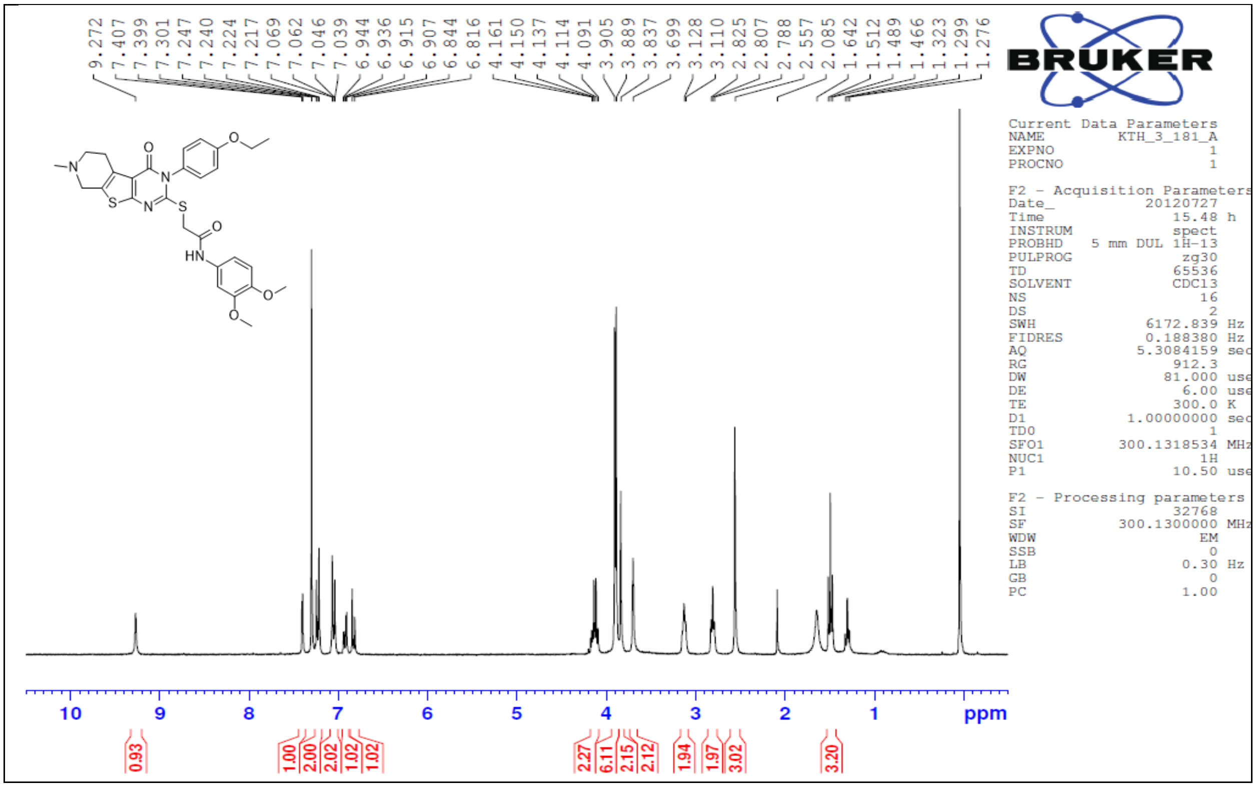

Figure S81. ^{13}C NMR Spectrum of **48** in $CDCl_3$

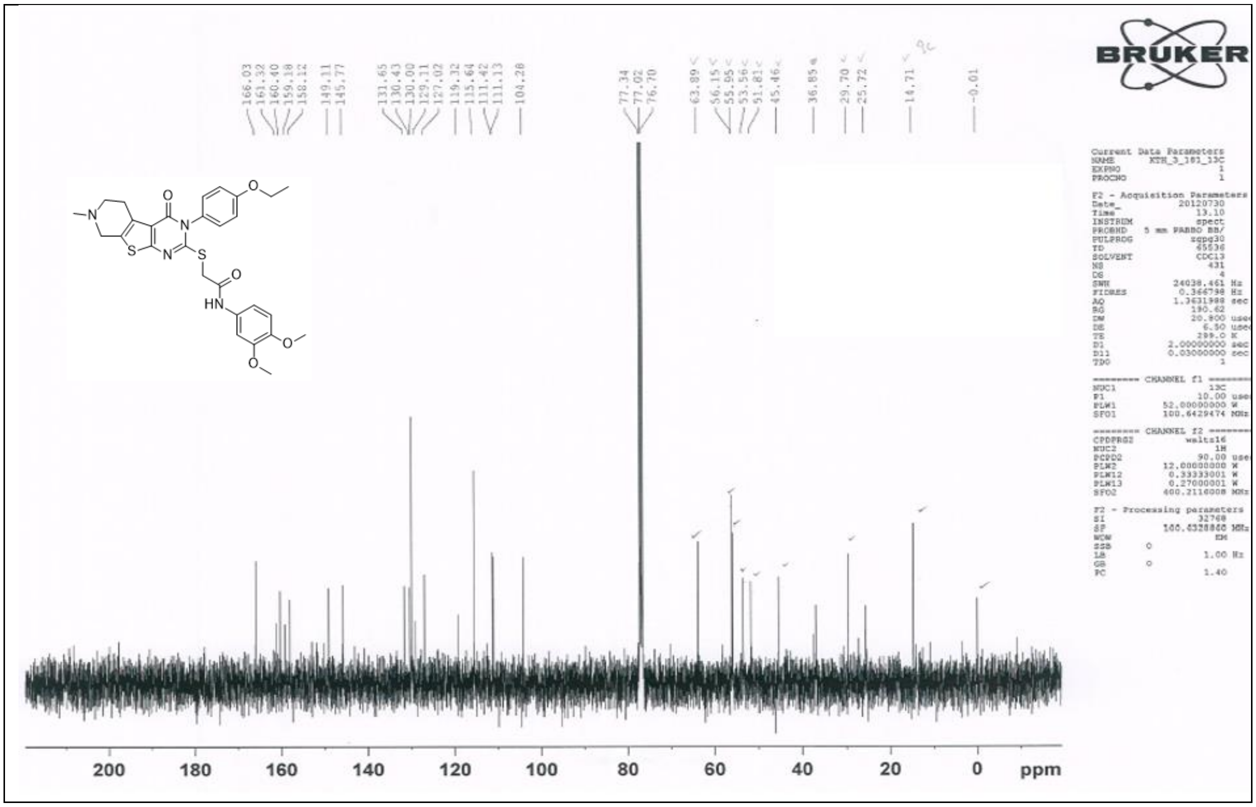

Figure S82. ^{1}H NMR Spectrum of **49** in $CDCl_3$

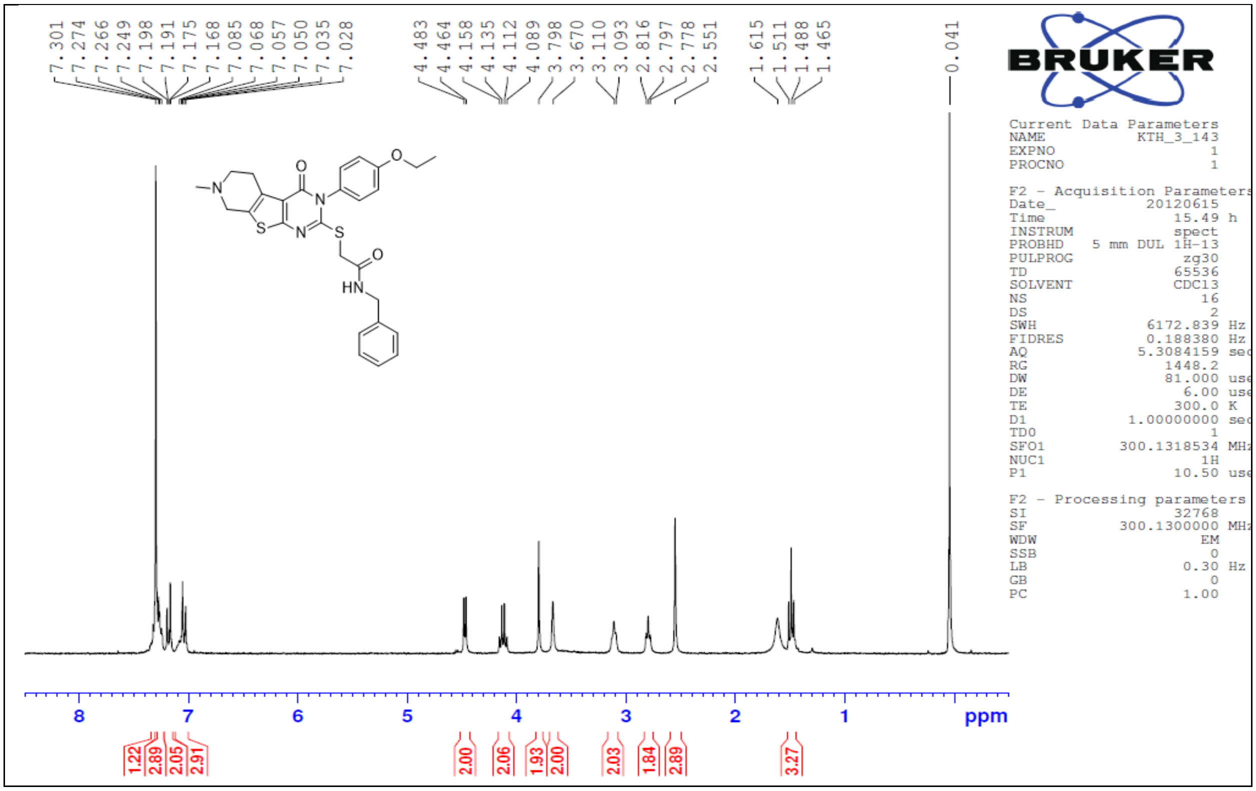

Figure S83. ^{13}C NMR Spectrum of **49** in $CDCl_3$

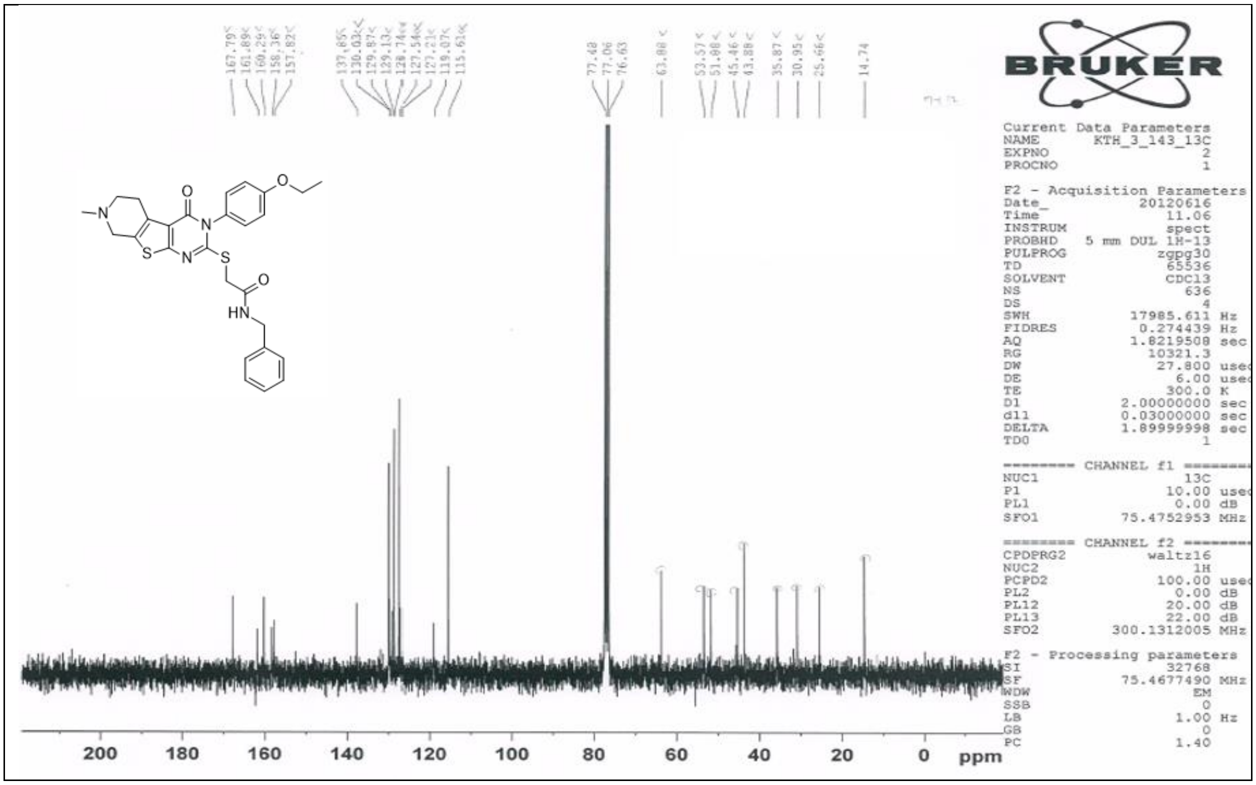

Figure S84. ^{1}H NMR Spectrum of **50** in $CDCl_3$

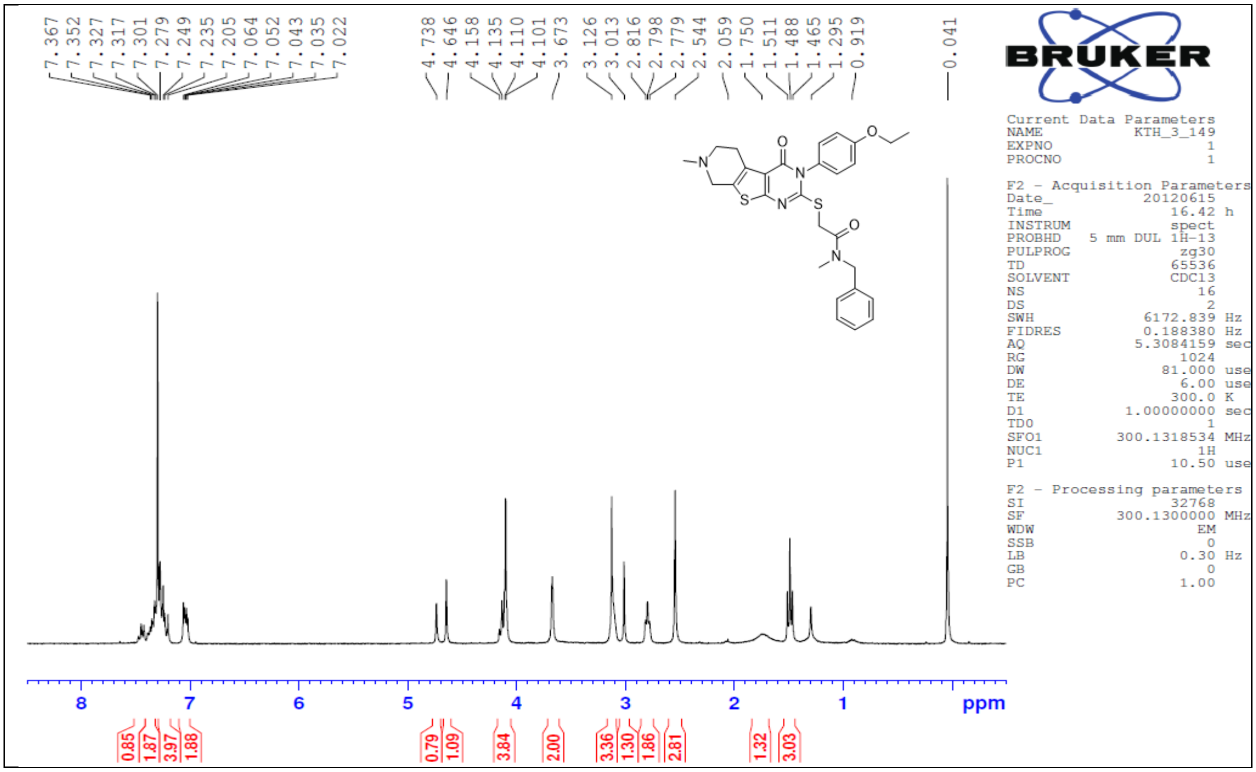

Figure S85. ^{13}C NMR Spectrum of **50** in $CDCl_3$

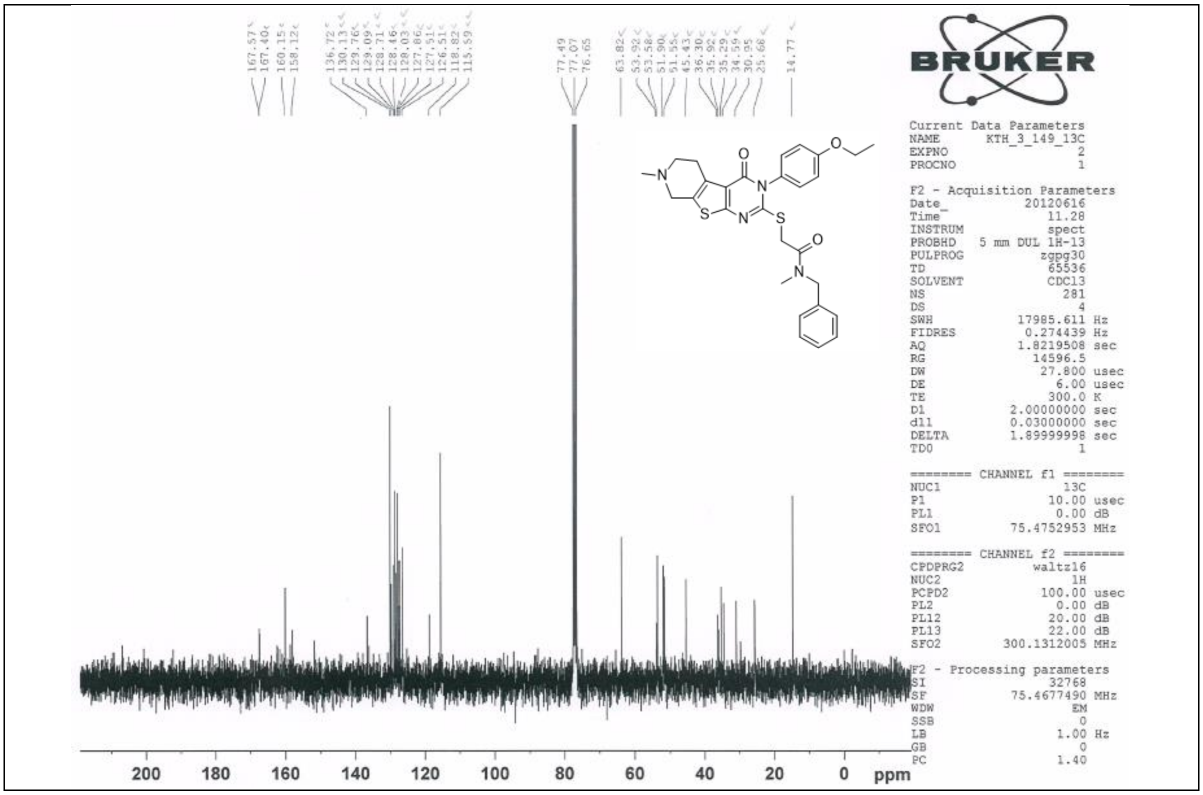

Figure S86. ^{1}H NMR Spectrum of **51** in $CDCl_3$

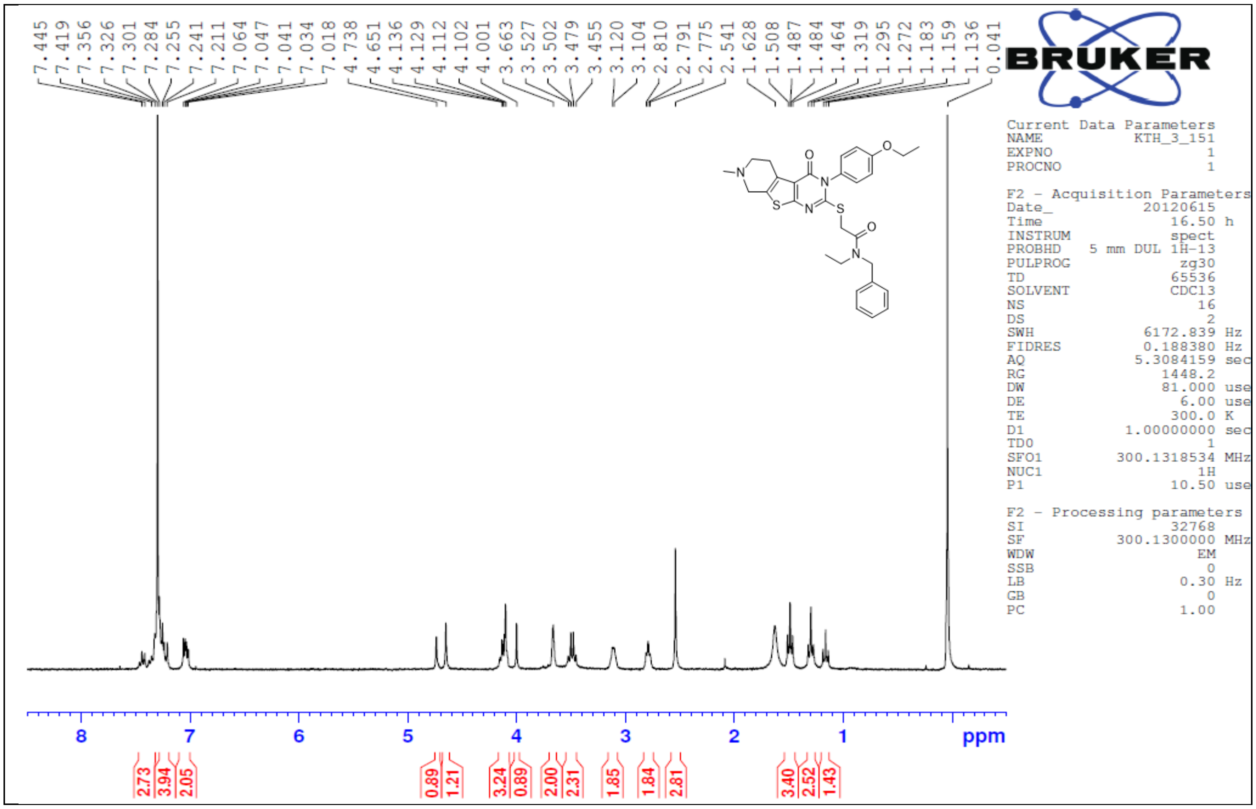

Figure S87. ^{13}C NMR Spectrum of **51** in $CDCl_3$

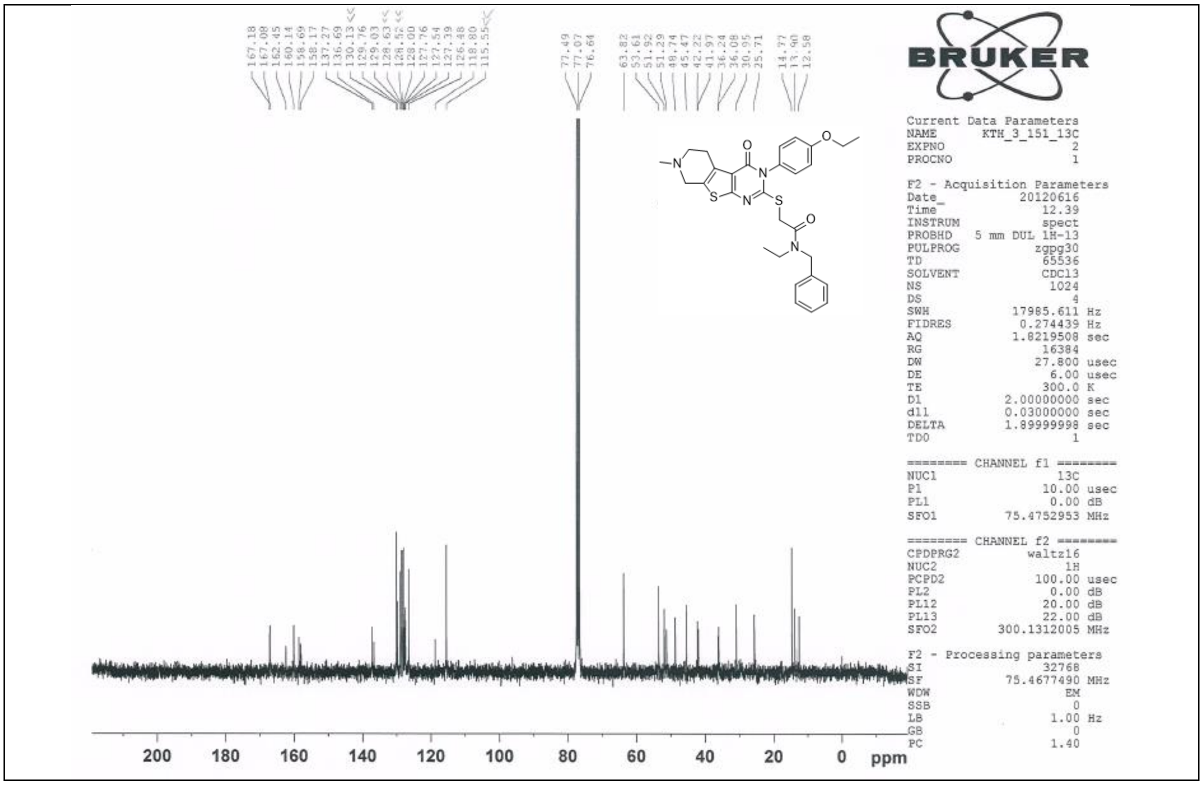

Figure S88. ^{1}H NMR Spectrum of **52** in $CDCl_3$

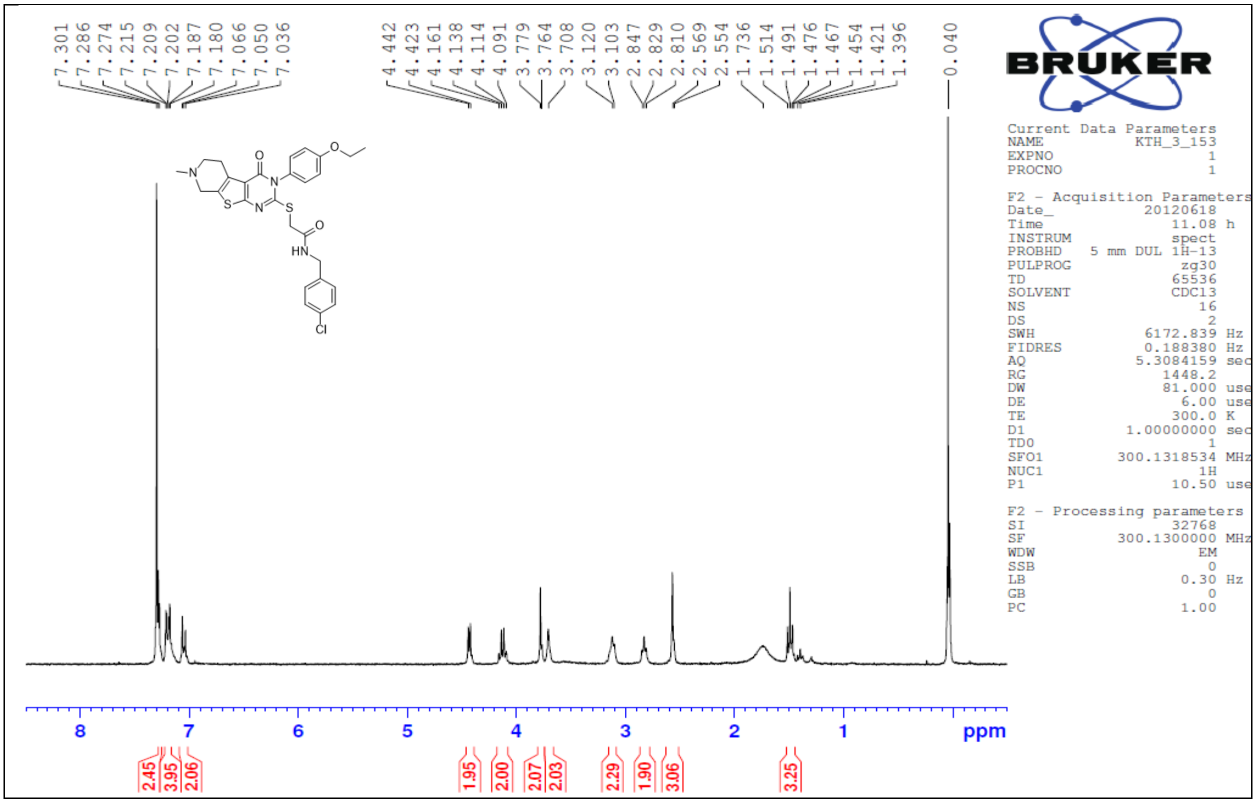

Figure S89. ^{13}C NMR Spectrum of **52** in $CDCl_3$

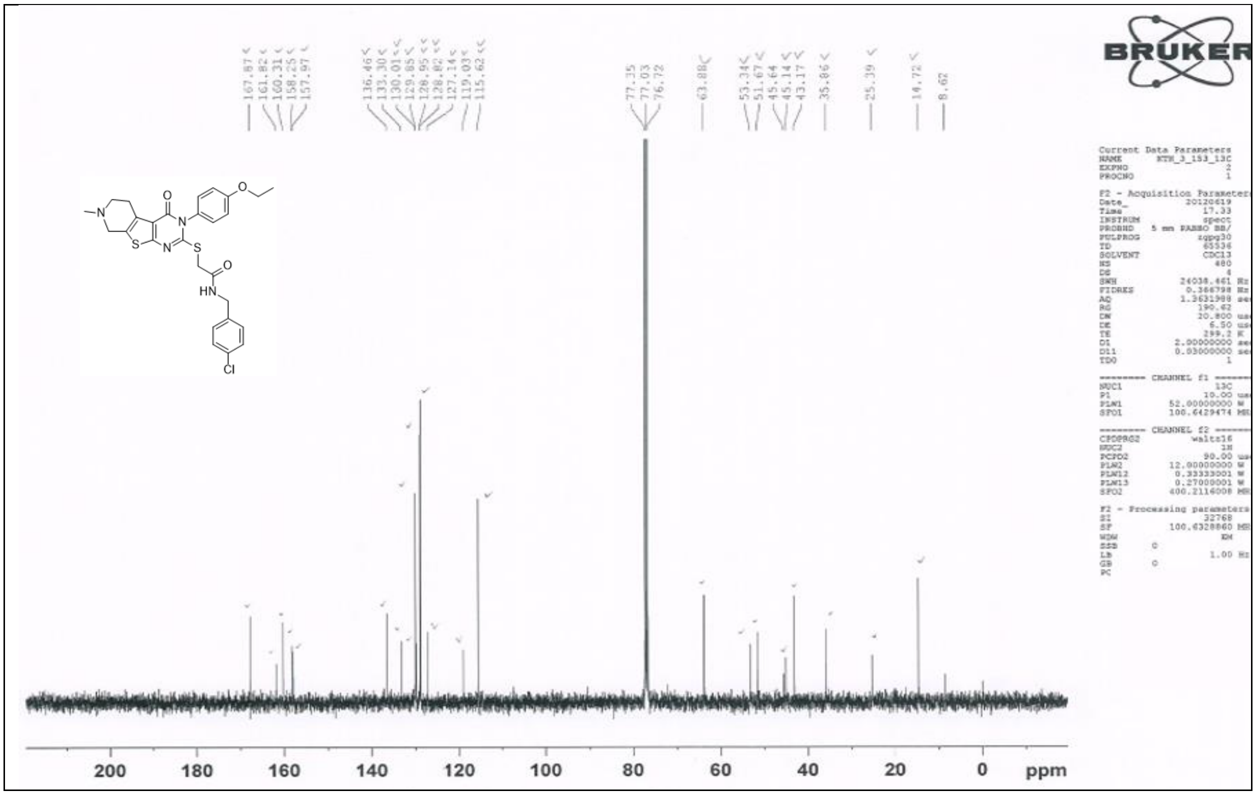

Figure S90. ^{1}H NMR Spectrum of **53** in $CDCl_3$

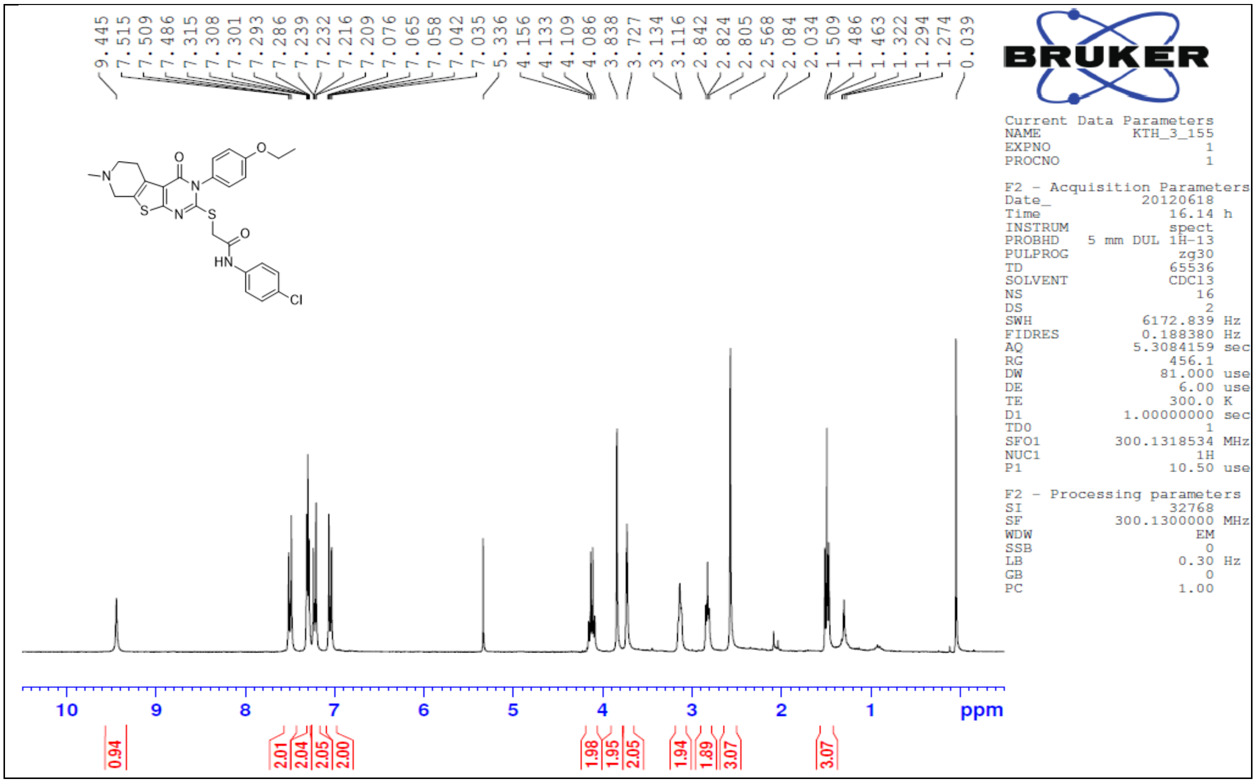

Figure S91. ^{13}C NMR Spectrum of **53** in $CDCl_3$

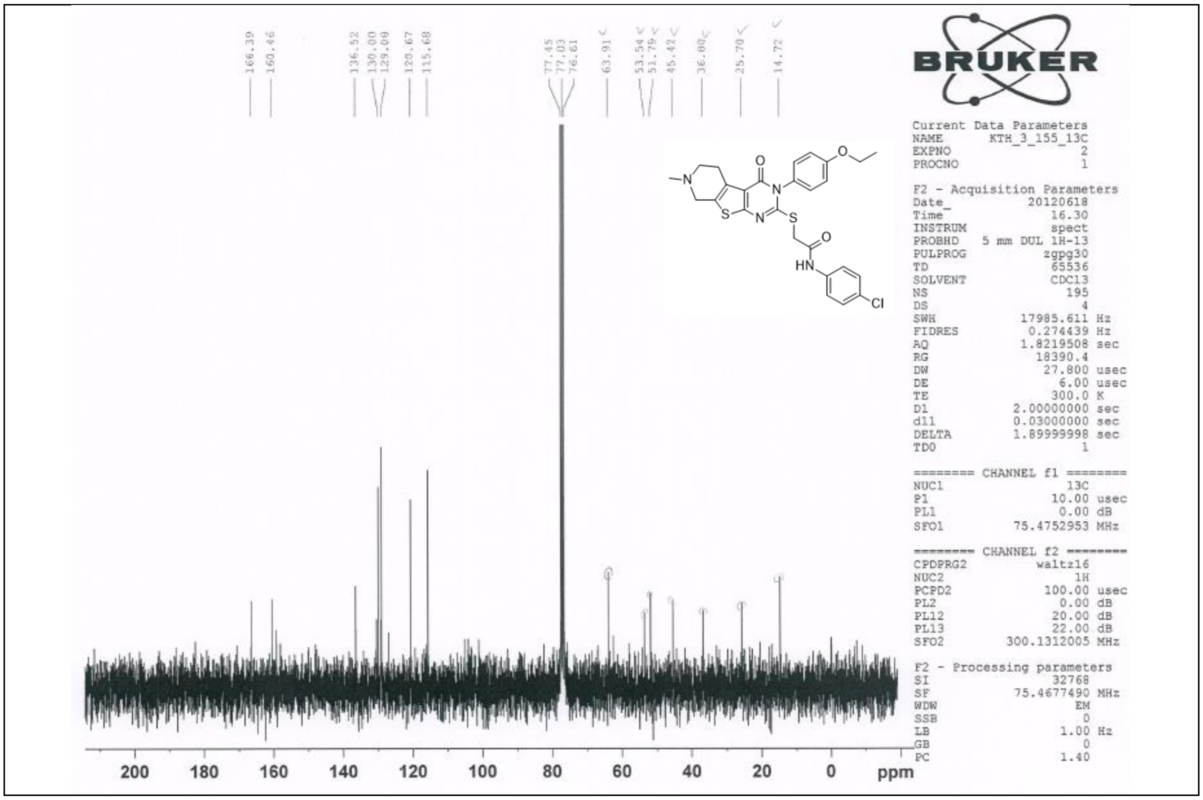

Figure S92. ^{1}H NMR Spectrum of **54** in $CDCl_3$

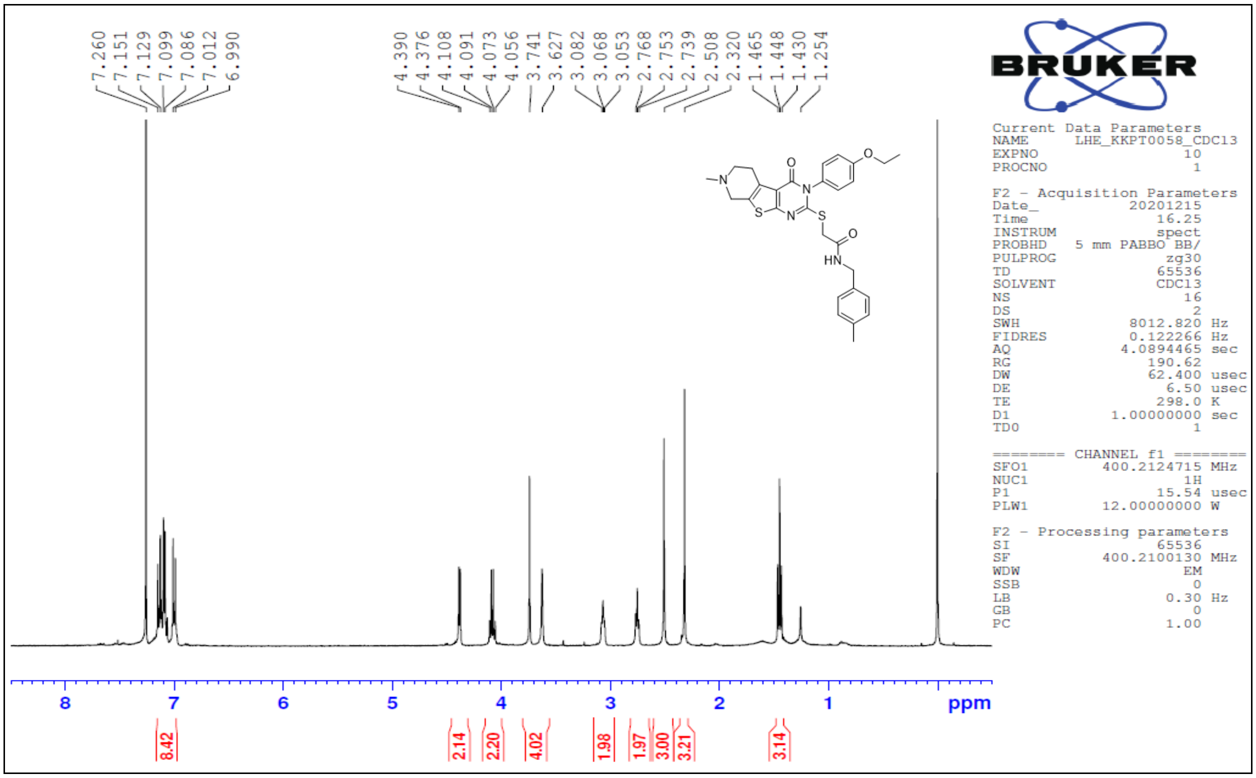

Figure S93. ^{13}C NMR Spectrum of **54** in $CDCl_3$

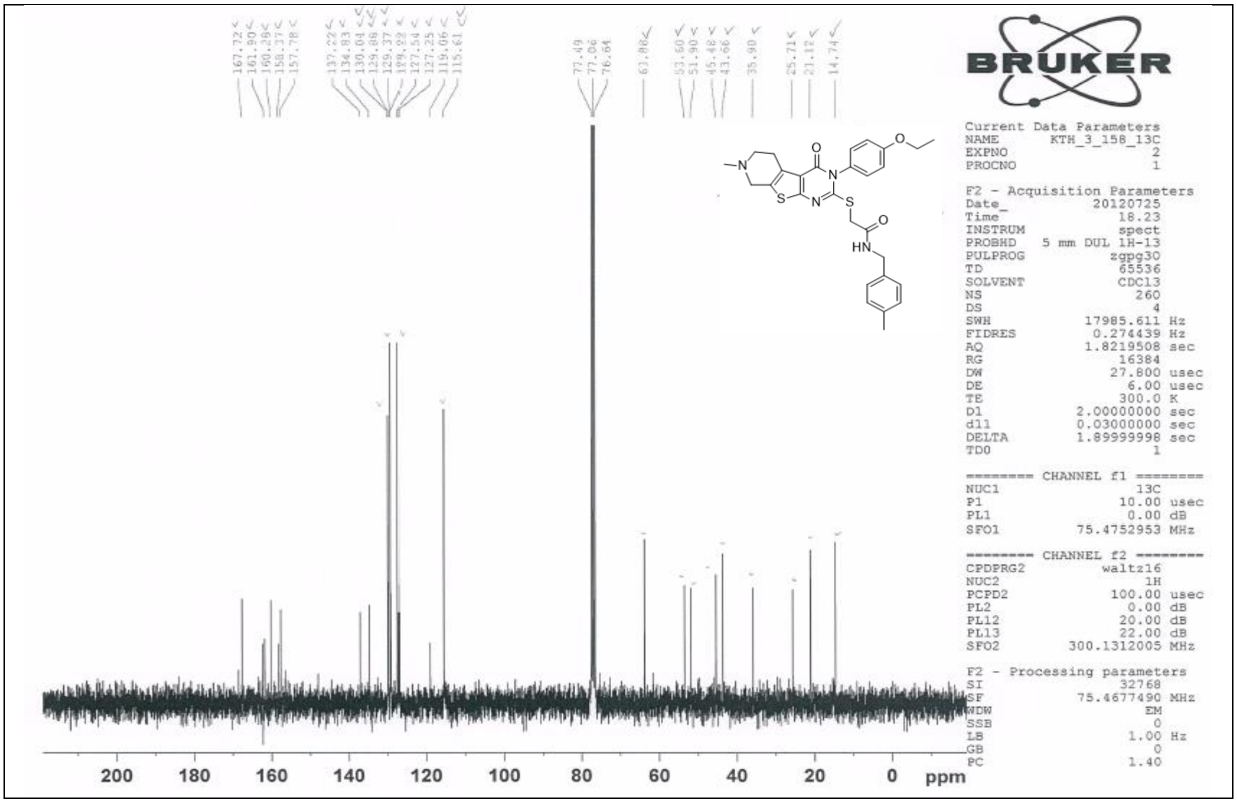

Figure S94. ^{1}H NMR Spectrum of **55** in $CDCl_3$

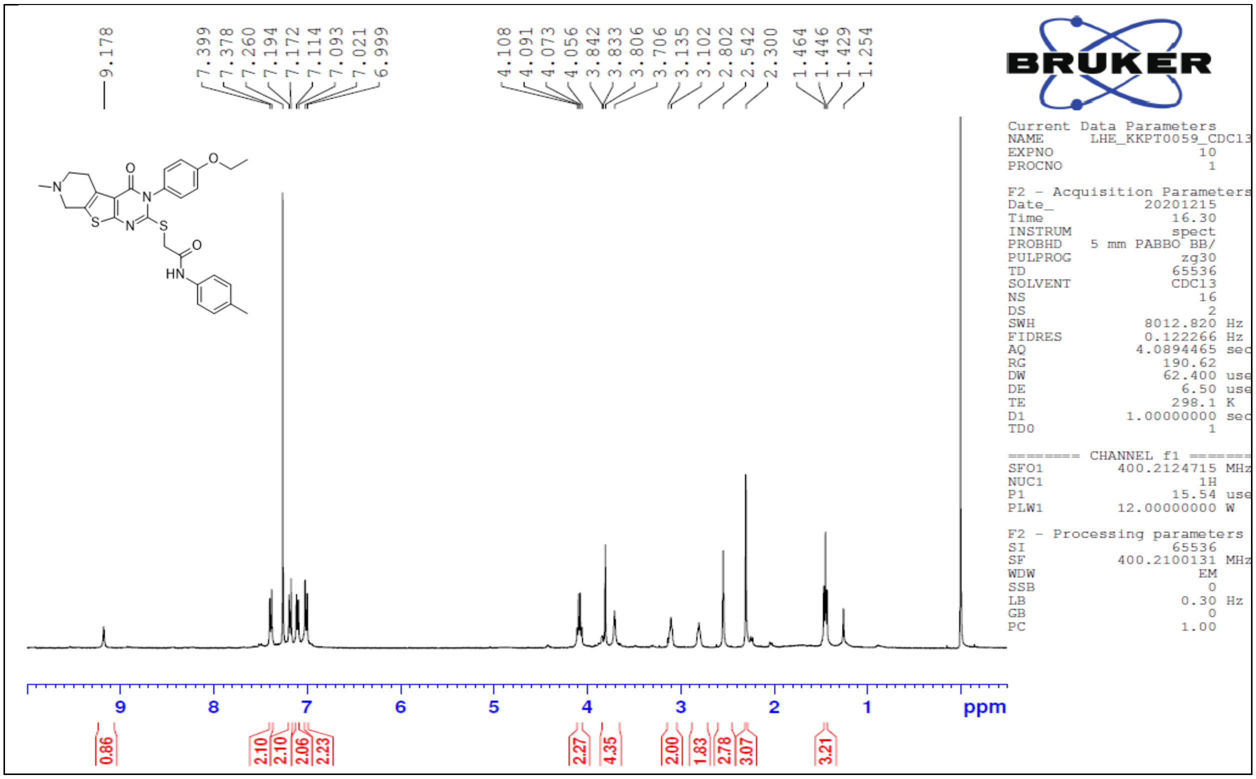

Figure S95. ^{13}C NMR Spectrum of **55** in $CDCl_3$

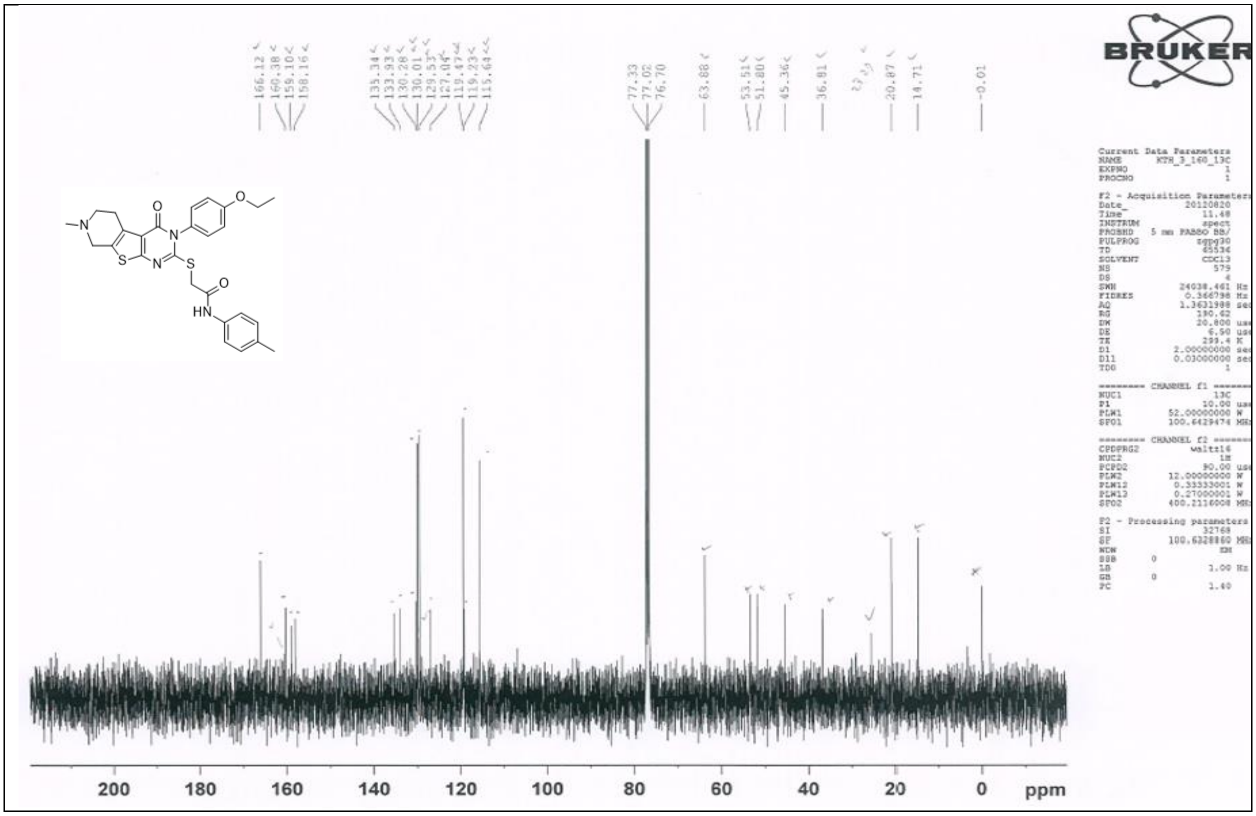

Figure S96. ^{1}H NMR Spectrum of **56** in $CDCl_3$

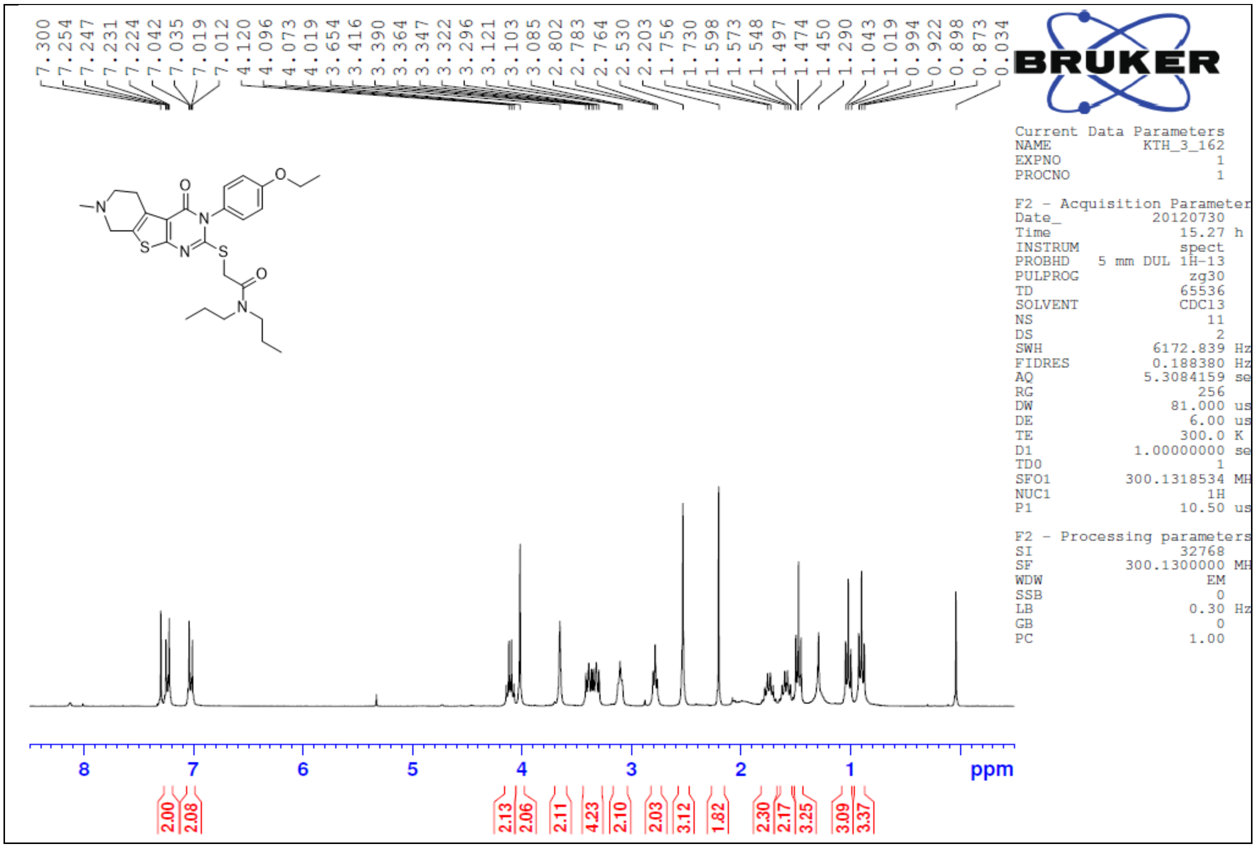

Figure S97. ^{13}C NMR Spectrum of **56** in $CDCl_3$

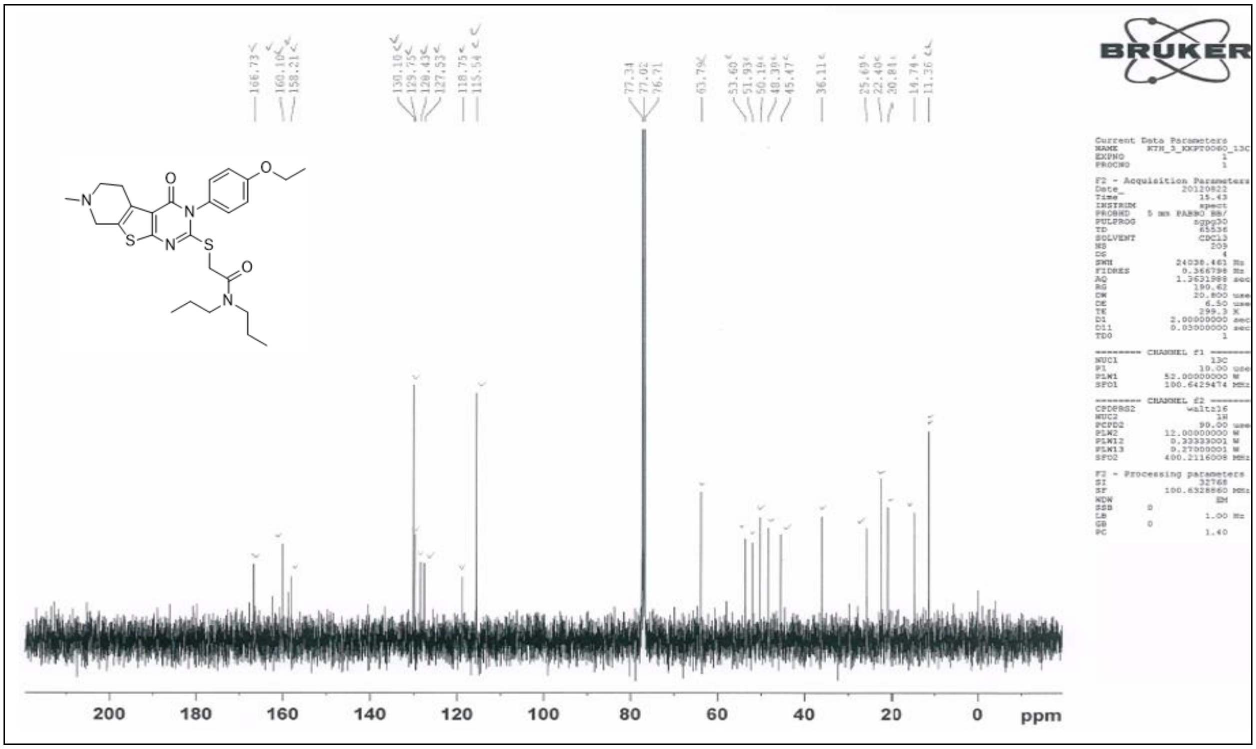

Figure S98. ^{1}H NMR Spectrum of **57** in $CDCl_3$

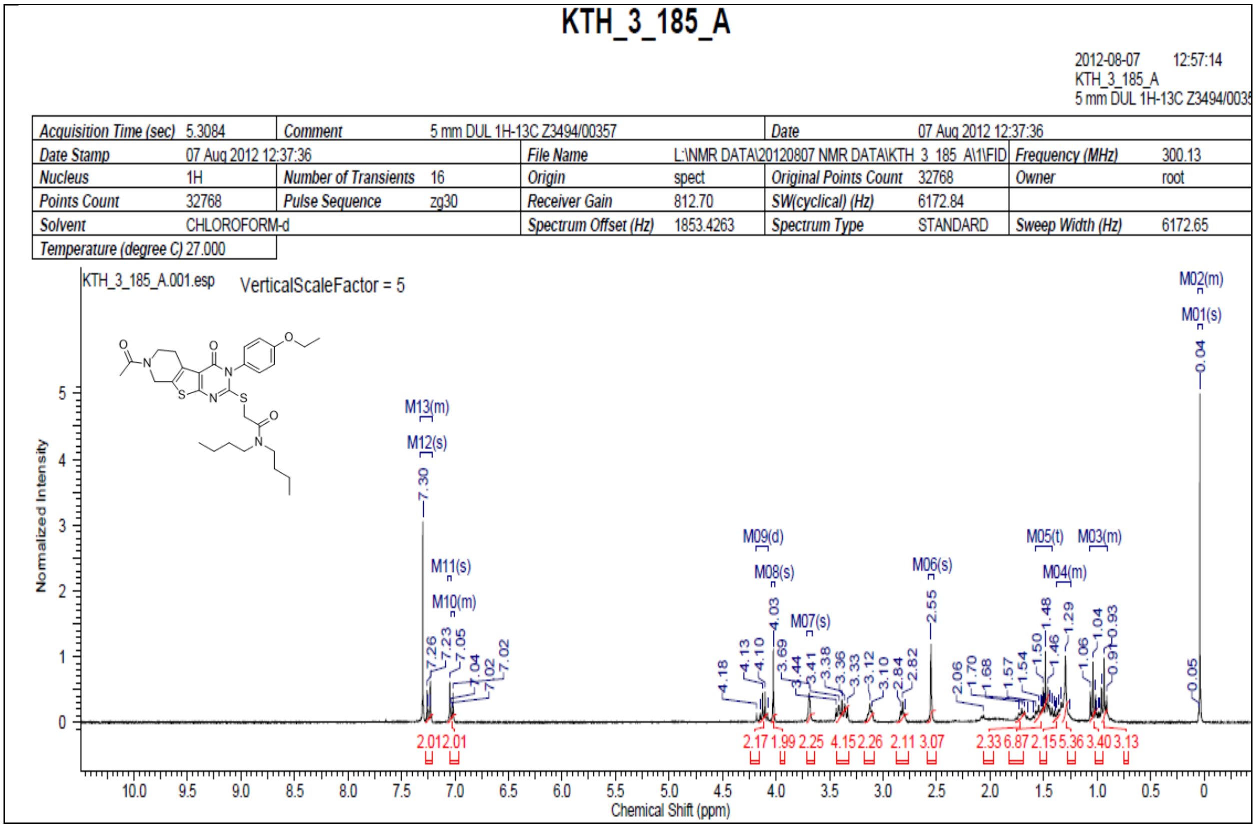

Figure S99. ^{13}C NMR Spectrum of **57** in $CDCl_3$

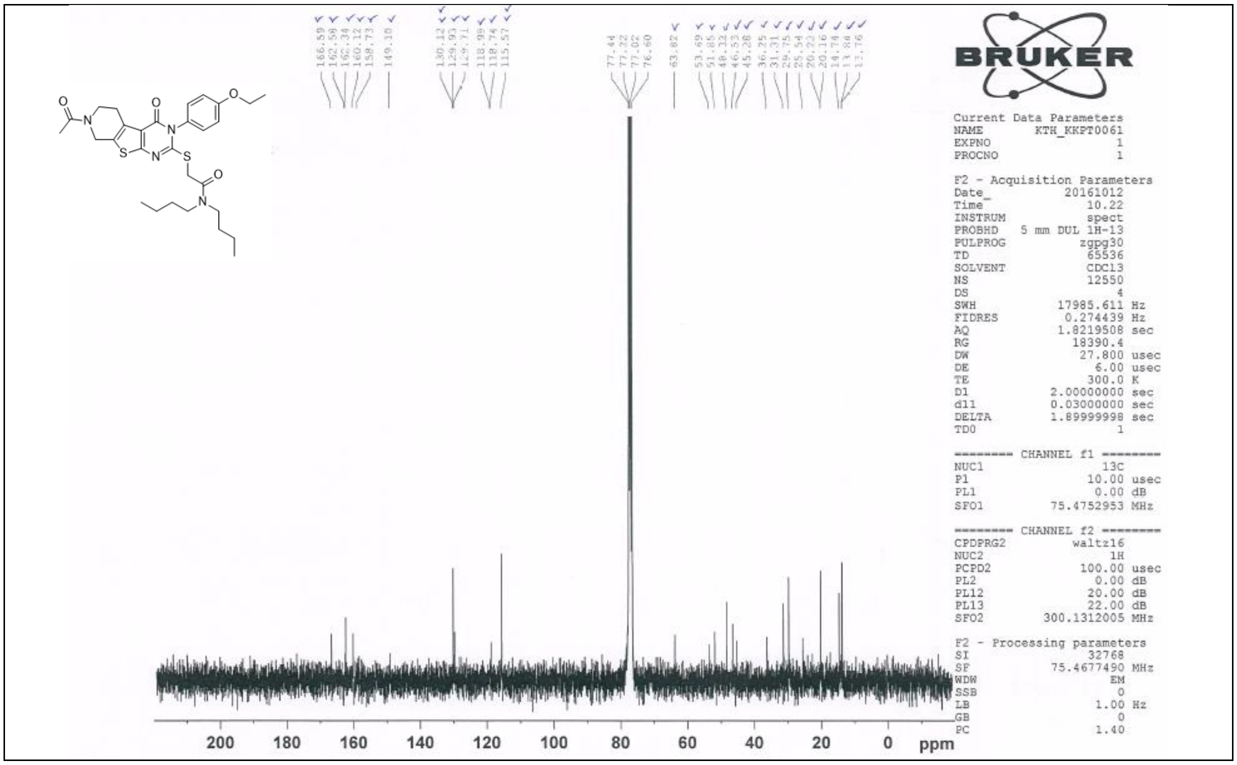

Figure S100. ^{1}H NMR Spectrum of **58** in $CDCl_3$

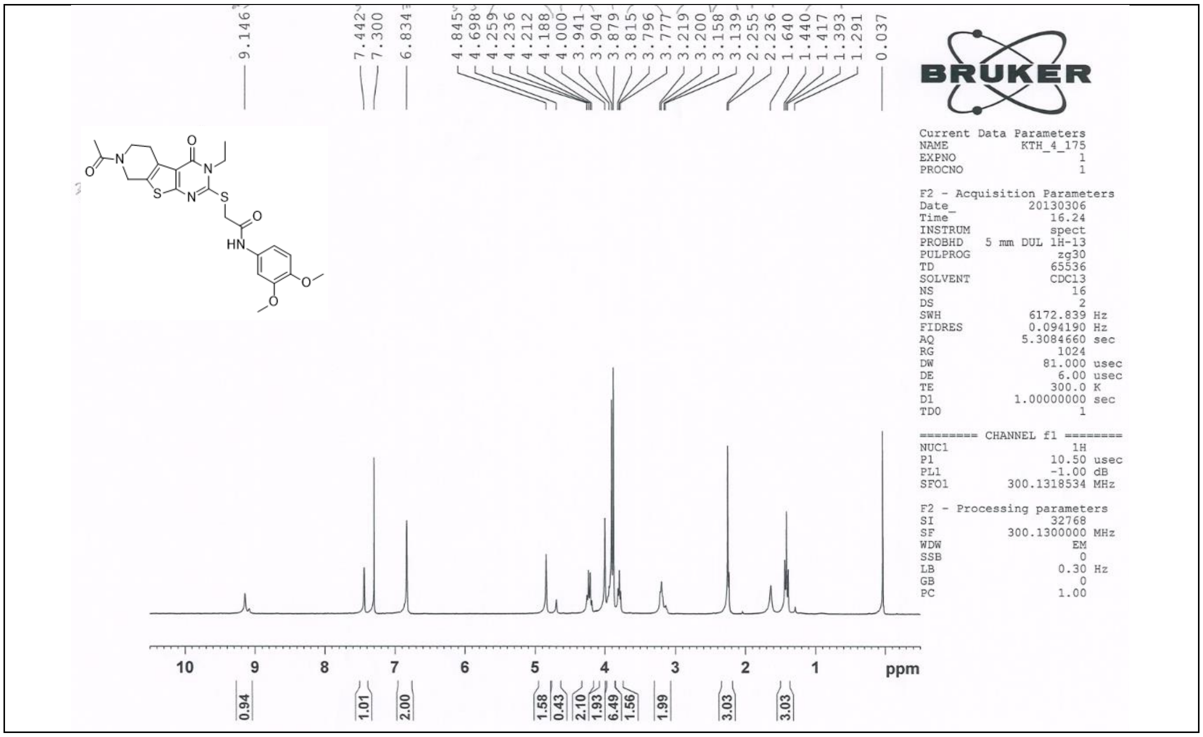

Figure S101. ^{13}C NMR Spectrum of **58** in $CDCl_3$

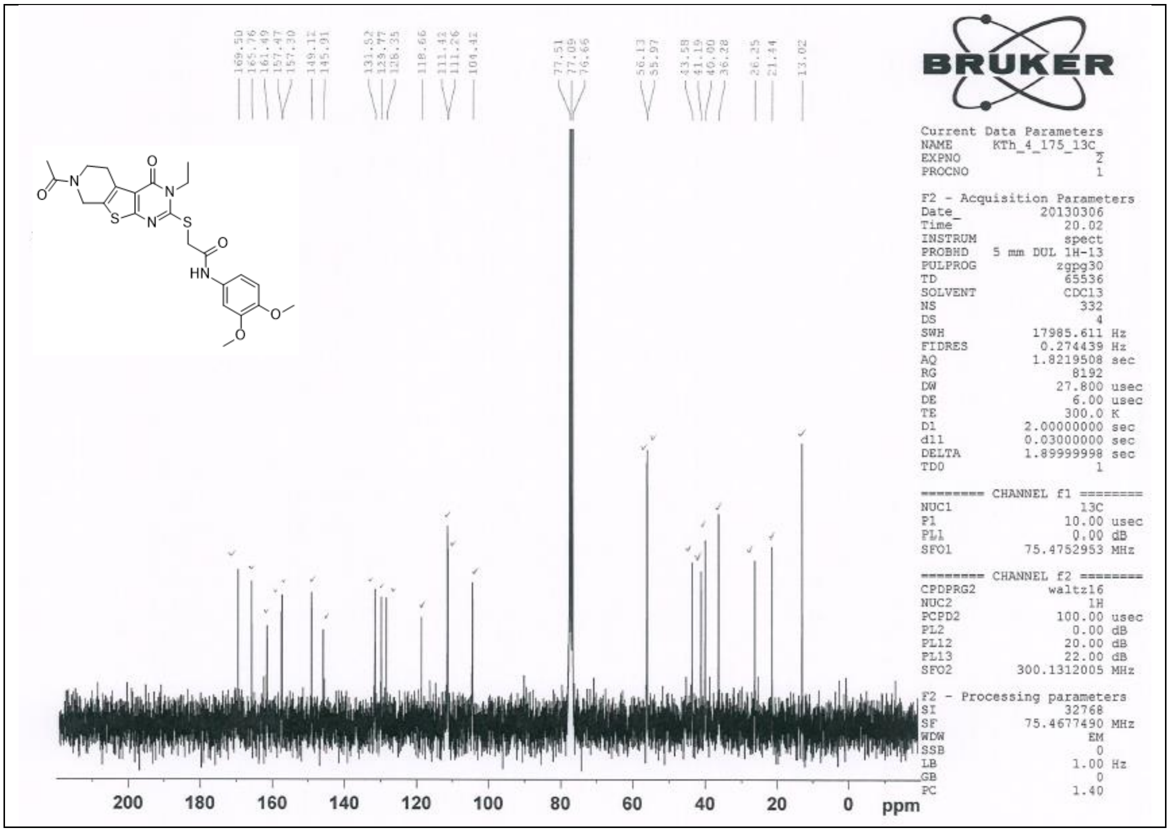

Figure S102. ^{1}H NMR Spectrum of **59** in $CDCl_3$

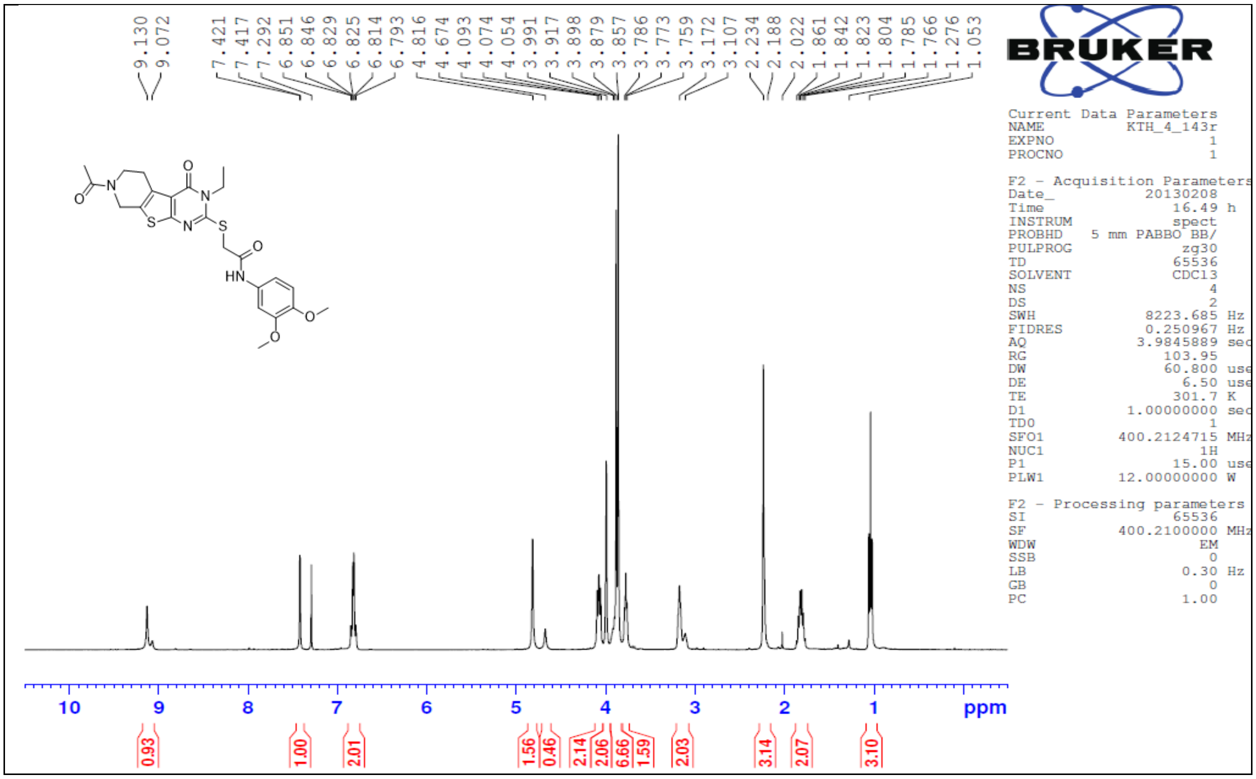

Figure S103. ^{13}C NMR Spectrum of **59** in $CDCl_3$

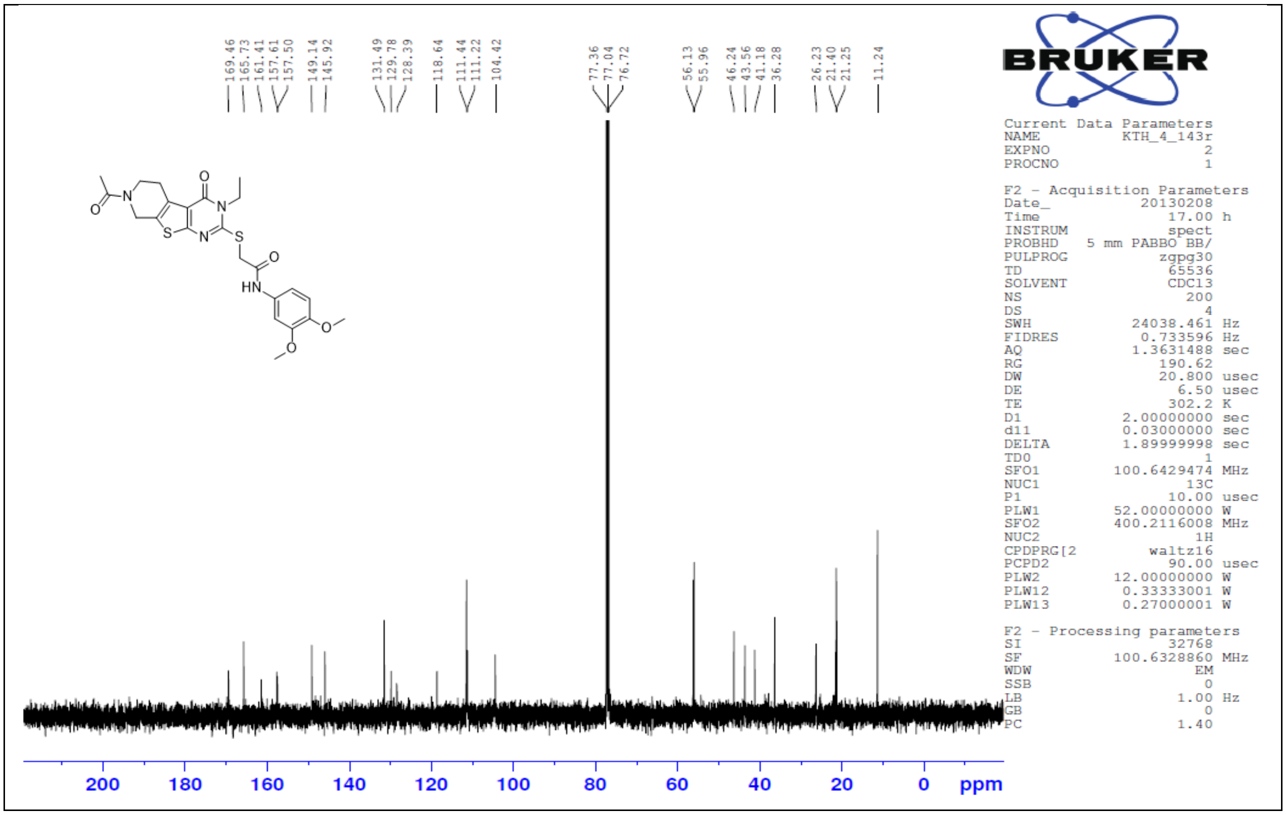

Figure S104. ^{1}H NMR Spectrum of **60** in $CDCl_3$

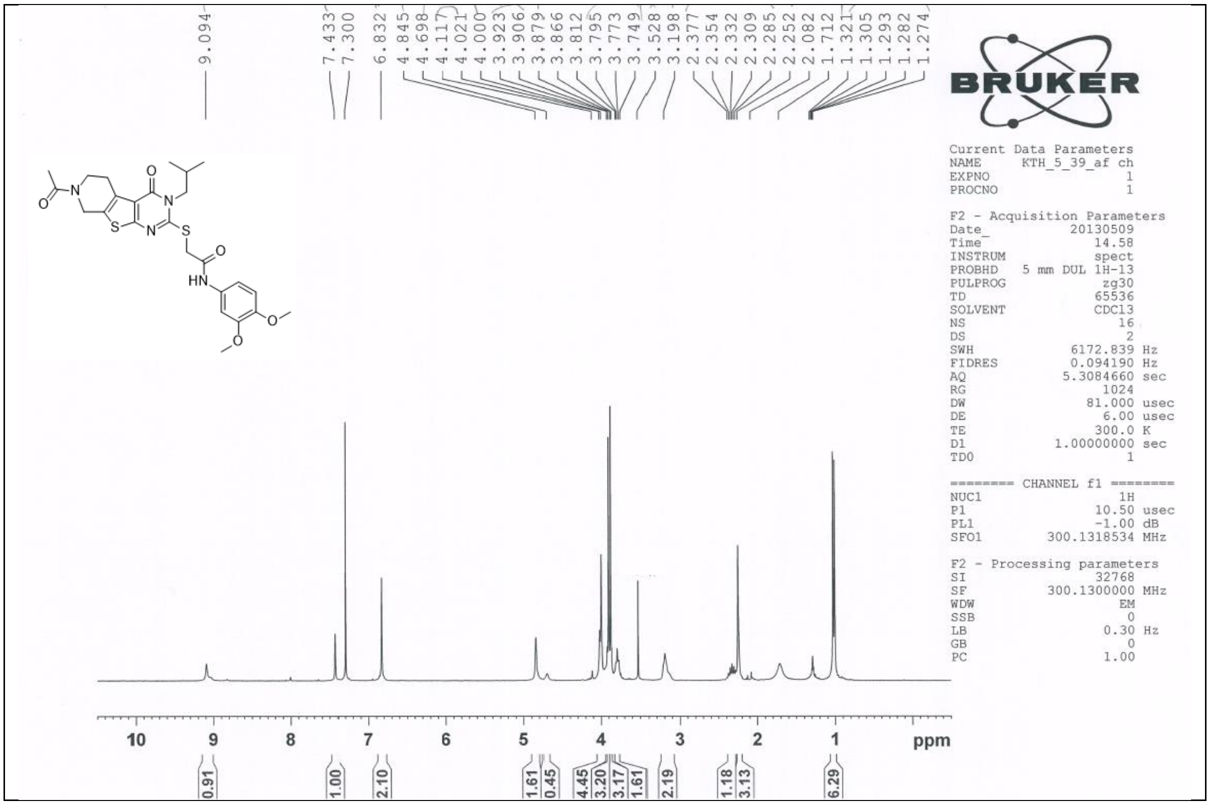

Figure S105. ^{13}C NMR Spectrum of **60** in $CDCl_3$

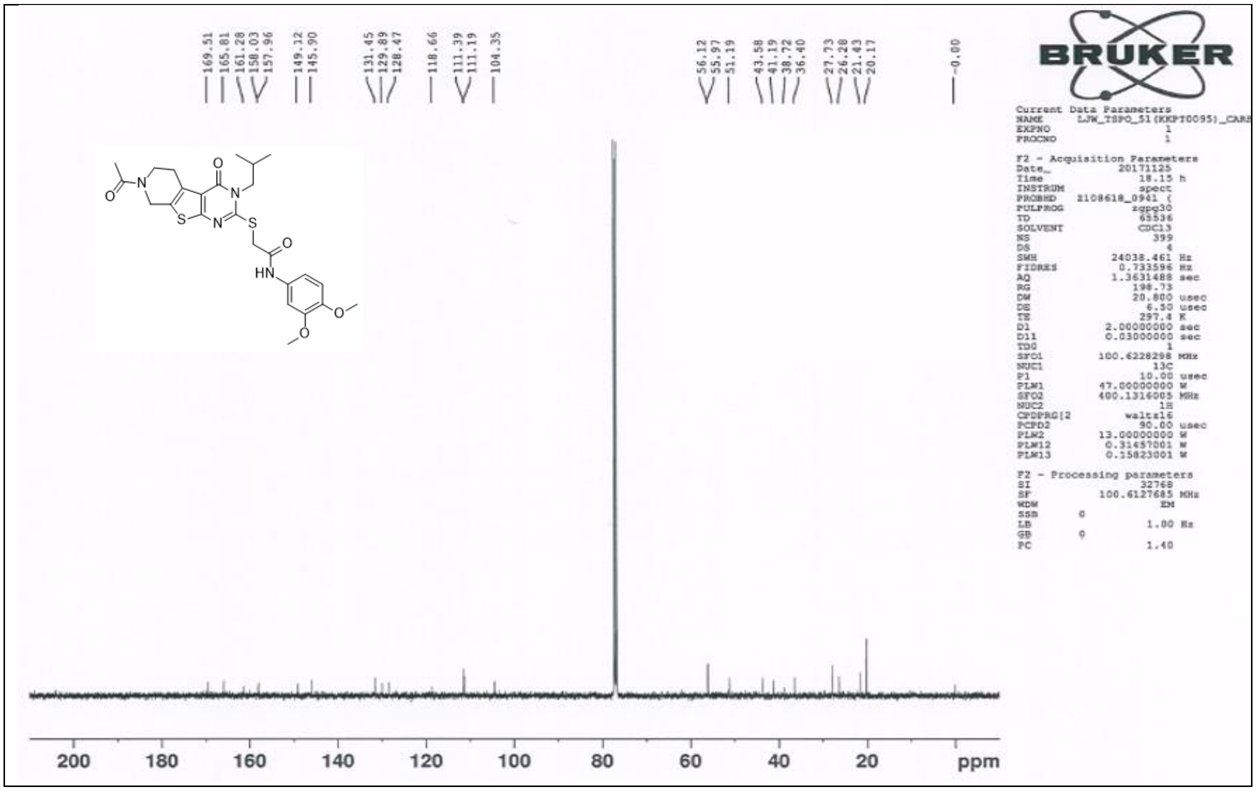

Figure S106. ^{1}H NMR Spectrum of **61** in $CDCl_3$

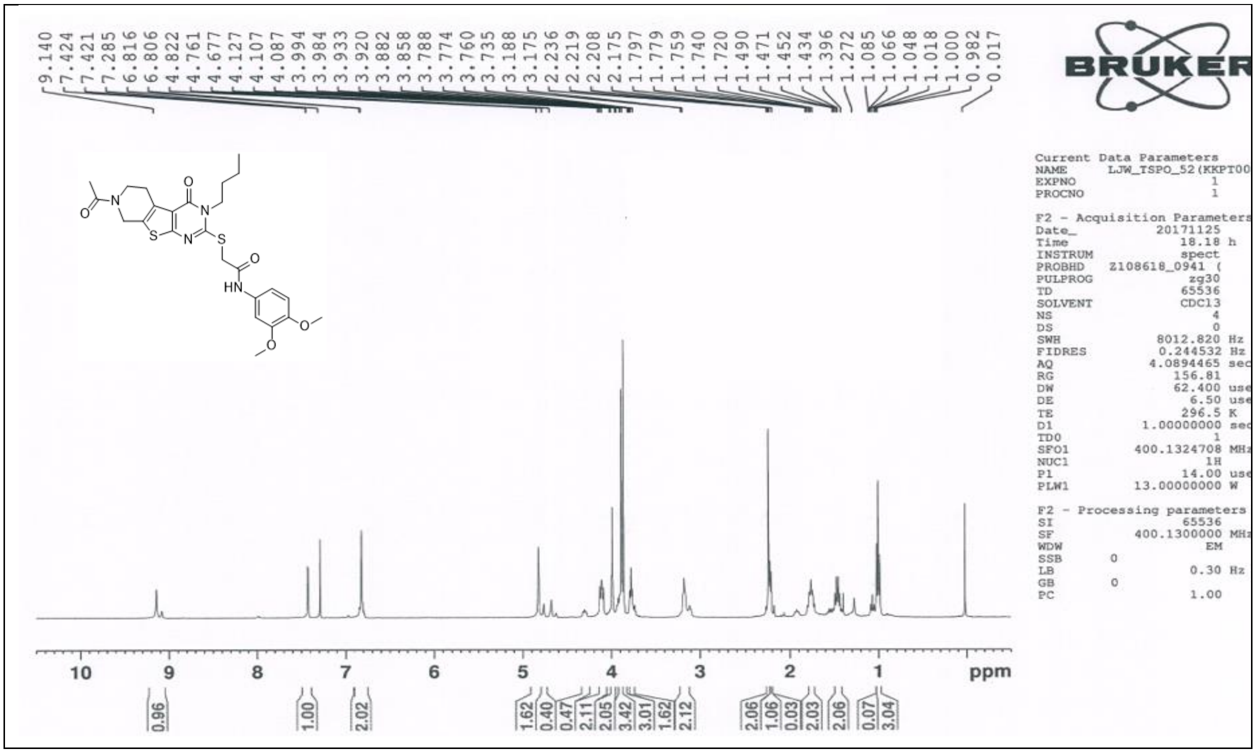

Figure S107. ^{13}C NMR Spectrum of **61** in $CDCl_3$

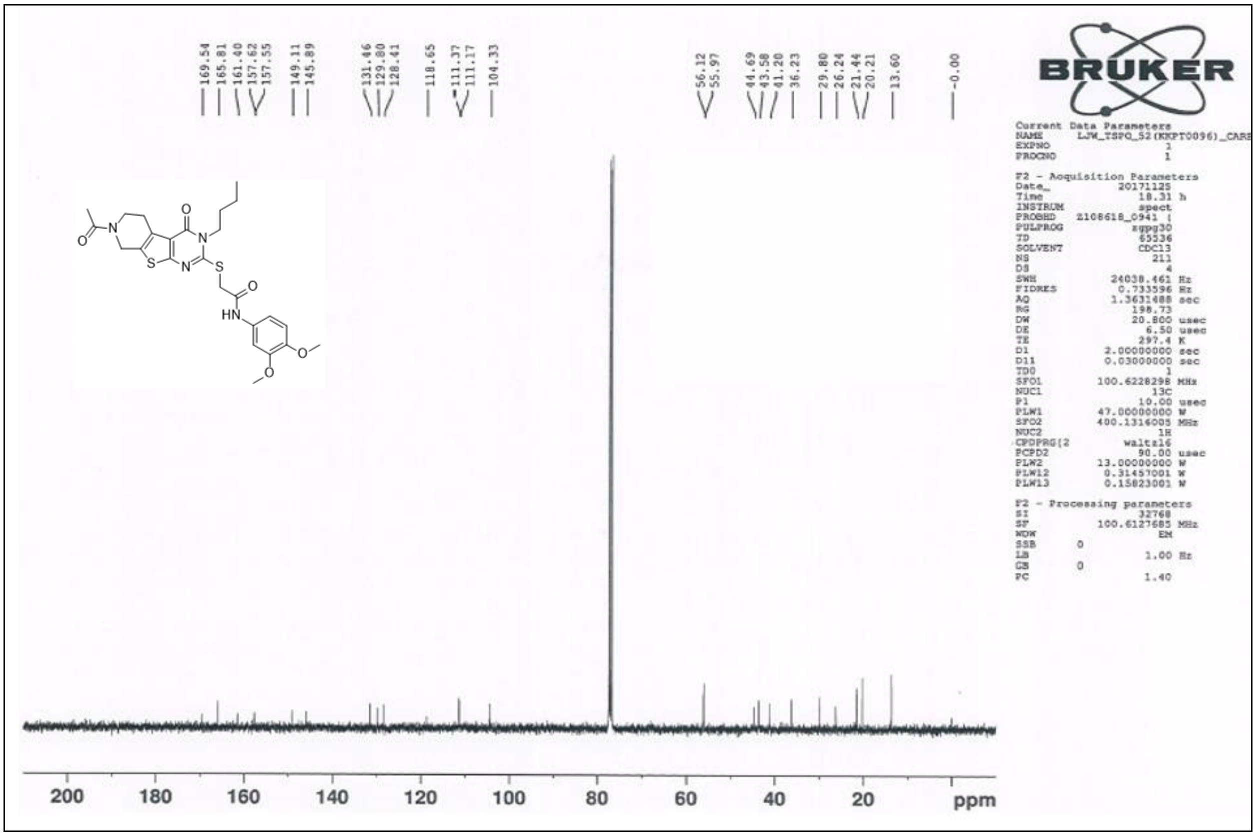

Figure S108. ^{1}H NMR Spectrum of **62** in $CDCl_3$

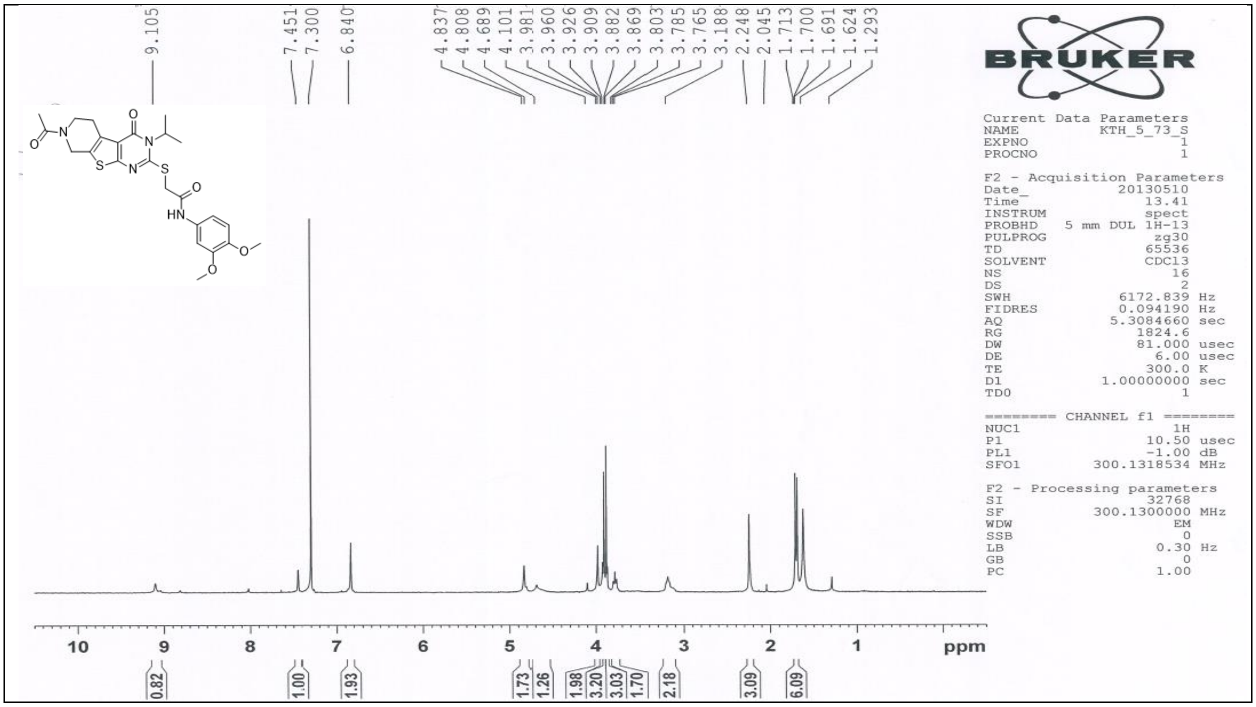

Figure S109. ^{13}C NMR Spectrum of **62** in $CDCl_3$

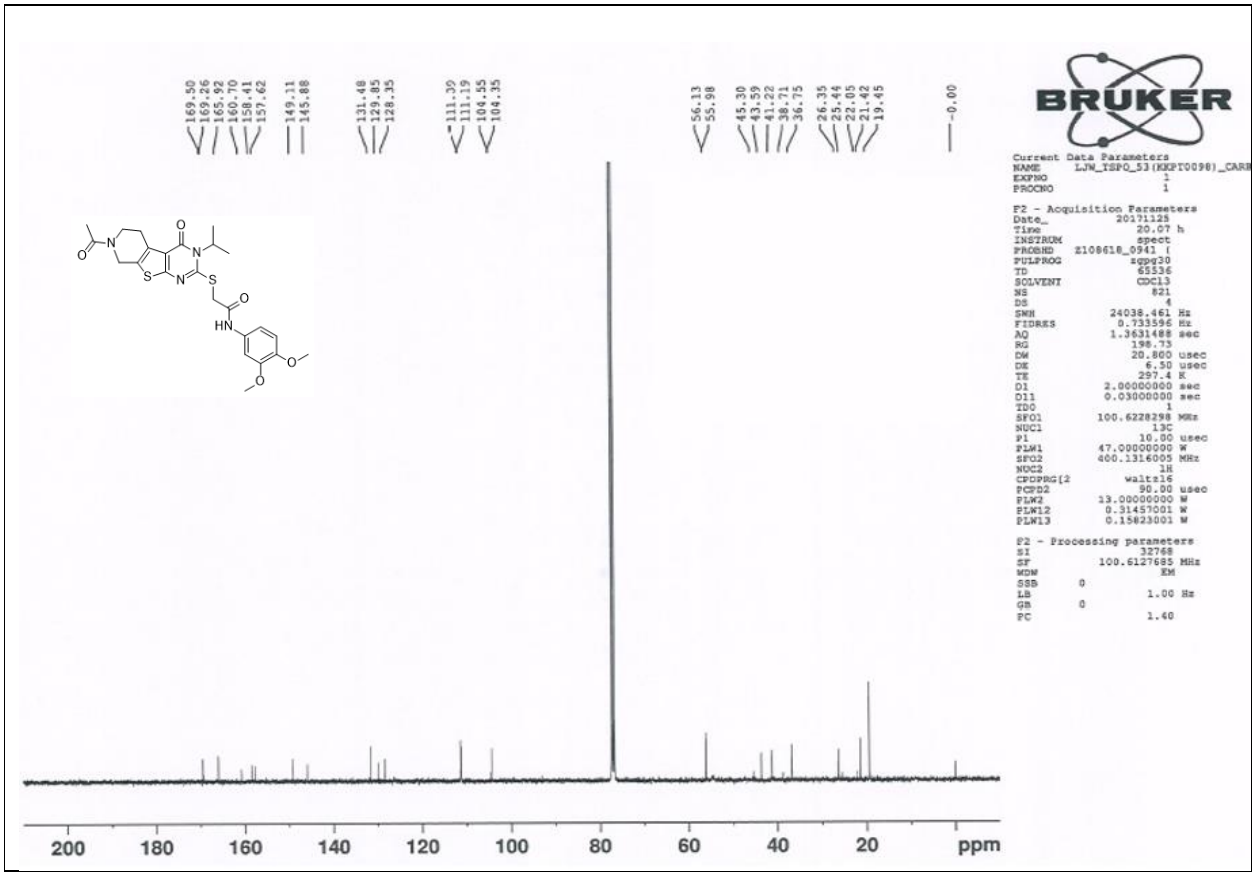

Chemical synthesis and characterization

All reactions were carried out under nitrogen atmosphere and commercially obtained reagents were used without further purification. Flash column chromatography were performed using silica gel 60 (Merck). ^{1}H NMR and ^{13}C NMR spectra were recorded on Bruker 500 spectrometer. Chemical shifts (δ) are reported in parts per million (ppm) values using tetramethylsilane (TMS) as the internal standard, and the coupling constants (J) are reported in hertz (Hz). Melting points were determined using an OptiMelt melting point apparatus (Stanford Research System, Inc.). All of the final compounds were found to have purity ≥95%. The purity of the samples was determined by analytical HPLC using a Waters E2695 system with SunFire C_{18} column (4.6 mm × 150 mm; 5 μm), with the gradient of H_2O/MeCN, 90/10 → 0/100 in 20 min, +3 min isocratic, flow rate of 1.0 mL/min, λ = 254 and 280 nm. High–resolution mass spectra (HRMS) were recorded on a LTQ Orbitrap (Thermo Electron Corporation) instrument.

Procedure for the synthesis of ethyl 6-acetyl-2-isothiocyanato-4,5,6,7-tetrahydrothieno[2,3-c]pyridine-3-carboxylate ***(23a).*** A mixture of ethyl 6-acetyl-2-amino-4,5,6,7-tetrahydrothieno[2,3-*c*]pyridine-3-carboxylate **22a** (2.00 g, 7.45 mmol, 1.0 equiv.) and 1,1'-thiocarbonyldipyridin-2(1*H*)-one (1.82 g, 7.83 mmol, 1.05 equiv.) in THF (74 mL) was heated under the reflux for 4 h. After cooling to room temperature, the reaction mixture was extracted with CH_2Cl_2 (3 × ~ 100 mL) and washed with H_2O (~ 100 mL). And the organic layer was dried over anhydrous $MgSO_4$, and concentrated in vacuo. The residue was purified by column chromatography (SiO_2, CH_2Cl_2/diethyl ether 4/1) to yield the title product **23a** (1.99 g, 86%) as a yellow solid; R_f = 0.30 (CH_2Cl_2/diethyl ether 4/1); ^{1}H NMR (400 MHz, $CDCl_3$, mixture of the rotamers) δ 4.71 (s, 1.5H), 4.58 (s, 0.5H), 4.40 (q, J = 6.0 Hz, 2H), 3.87 (t, J = 6.0 Hz, 0.5H), 3.73

(t, *J* = 6.0 Hz, 1.5H), 2.96-3.04 (m, 2H), 2.23 (s, 2H), 2.20 (s, 1H), 1.45 (t, *J* = 6.0 Hz, 3H); ^{13}C NMR (101 MHz, $CDCl_3$, mixture of the rotamers) δ 170.98, 169.26, 166.66, 150.89, 132.82, 111.67, 61.24, 61.06, 43.81, 43.54, 40.84, 40.70, 39.15, 27.21, 27.04, 26.31, 21.88, 21.40, 21.31, 14.43, 14.34, 14.27.

Ethyl 2-isothiocyanato-6-methyl-4,5,6,7-tetrahydrothieno[2,3-c]pyridine-3-carboxylate ***(23b).*** Following the same procedure for the synthesis of **23a**, a mixture of 2-amino-6-methyl-4,5,6,7-tetrahydrothieno[2,3-*c*]pyridine-3-carboxylate **22b** (210 mg, 0.87 mmol, 1.0 equiv.) and 1,1'-thiocarbonyldipyridin-2(1*H*)-one (203 mg, 0.87 mmol, 1.0 equiv.) in THF (9 mL) gave the title product **23b** (198 mg, 80%) as a yellow solid; R_f = 0.25 (CH_2Cl_2/MeOH 10/1); ^1H NMR (400 MHz, $CDCl_3$) δ 4.37 (q, *J* = 6.0 Hz, 2H), 3.52 (s, 2H), 2.95 (t, *J* = 6.0 Hz, 2H), 2.73 (t, *J* = 6.0 Hz, 2H), 2.49 (s, 3H), 1.43 (t, *J* = 6.0 Hz, 3H); ^{13}C NMR (101 MHz, DMSO-d_6) δ 161.82, 133.72, 133.02, 128.91, 126.00, 116.01, 60.87, 53.38, 51.98, 45.42, 26.70, 14.42.

Procedure for the synthesis of ethyl 6-acetyl-2-(3-(4-methoxyphenyl)thioureido)-4,5,6,7-tetrahydrothieno[2,3-c]pyridine-3-carboxylate ***(24aa).*** To a stirred solution of ethyl 6-acetyl-2-isothiocyanato-4,5,6,7-tetrahydrothieno[2,3-*c*]pyridine-3-carboxylate **23a** (1.98 g, 6.39 mmol, 1.0 equiv.) in EtOH (64 mL) was added 4-methoxyaniline (1.57 g, 12.78 mmol, 2.0 equiv.). After stirring at room temperature for 12 h, the precipitate was filtered off, washed on the filter with EtOH (~ 50 mL), then dried in vacuo to give the title product **24aa** as a white solid (2.56 g, 93%); R_f = 0.60 (*n*-hexane/EtOAc 1/1); ^1H NMR (400 MHz, $CDCl_3$, mixture of the rotamers) δ 11.93-12.02 (m, 1H), 7.96-8.00 (m, 1H), 7.26 (d, *J* = 9.0 Hz, 2H), 6.99 (d, *J* = 9.0 Hz, 2H), 4.68 (s, 1H), 4.55 (s, 1 H), 4.07-4.18 (m, 4H), 3.82 (t, *J* = 6.0 Hz, 1H), 3.66 (t, *J* = 6.0 Hz, 1H), 2.84-2.92 (m, 2H), 2.18 (s, 3H), 1.47 (t, *J* = 6.4 Hz, 3H), 1.25-1.30 (m, 3H); ^{13}C NMR (101 MHz, $CDCl_3$) δ

177.02, 169.13, 165.67, 158.73, 151.39, 151.30, 130.65, 127.92, 21.42, 14.72, 14.23, 14.14; LC/MS (ESI^+, MeCN/H_2O): *m/z*: calcd for $C_{20}H_{24}N_3O_4S_2$: 434.11 $[M+H]^+$; found: 434.1.

*Ethyl 6-acetyl-2-(3-(4-ethoxyphenyl)thioureido)-4,5,6,7-tetrahydrothieno[2,3-c]pyridine-3-carboxylate (**24ab**).* Following the same procedure for the synthesis of **24aa**, ethyl 6-acetyl-2-isothiocyanato-4,5,6,7-tetrahydrothieno[2,3-*c*]pyridine-3-carboxylate **23a** (1.82 g, 5.86 mmol, 1.0 equiv.) and 4-ethoxyaniline (1.50 mL, 11.86 mmol, 2.0 equiv.) in EtOH (59 mL) gave the title product **24ab** (2.37 g, 90%) as a white solid; R_f = 0.55 (*n*-hexane/EtOAc 1/1); ^1H NMR (400 MHz, $CDCl_3$, mixture of the rotamers) δ 12.03 (br s, 0.5H), 11.94 (br s, 0.5H), 7.79 (br s, 1H), 7.27 (d, *J* = 6.0 Hz, 2H), 7.01 (d, *J* = 6.0 Hz, 2H), 4.69 (s, 1H), 4.55 (s, 1H), 4.12-4.18 (m, 2H), 3.88 (s, 3H), 3.82 (t, *J* = 6.0 Hz, 1H), 3.66 (t, *J* = 6.0 Hz, 1H), 2.84-2.92 (m, 2H), 2.16-2.22 (m, 3H), 1.26-1.30 (m, 4H); ^{13}C NMR (101 MHz, $CDCl_3$) δ 177.08, 169.07, 165.81, 159.53, 151.27, 130.68, 128.75, 128.01, 127.97, 123.33, 121.67, 115.29, 60.70, 60.57, 55.61, 44.91, 43.83, 40.69, 39.20, 27.12, 26.22, 21.87, 21.43, 14.22, 14.14; LC/MS (ESI^+, MeCN/H_2O): *m/z*: calcd for $C_{21}H_{26}N_3O_4S_2$: 448.13 $[M+H]^+$; found: 448.1.

*Ethyl 6-acetyl-2-(3-ethylthioureido)-4,5,6,7-tetrahydrothieno[2,3-c]pyridine-3-carboxylate (**24c**).* Following the same procedure for the synthesis of **24aa**, ethyl 6-acetyl-2-isothiocyanato-4,5,6,7-tetrahydrothieno[2,3-*c*]pyridine-3-carboxylate **23a** (60 mg, 0.19 mmol, 1.0 equiv.) and ethanamine (2.0 M in THF, 145 μL, 0.29 mmol, 1.5 equiv.) in EtOH (2 mL) gave the title product **24c** (45 mg, 67%) as a white solid; R_f = 0.15 (DCM/MeOH 20/1); ^1H NMR (400 MHz, $CDCl_3$, mixture of the rotamers) δ 12.09 (br s, 1H), 6.29-6.34 (br m, 1H), 4.68 (s, 1H), 4.55 (s, 1H), 4.35 (q, *J* = 6.9 Hz, 2H), 3.84 (t, *J* = 6.0 Hz, 1H), 3.69 (t, *J* = 6.0 Hz, 1H), 3.58 (m, 2H), 2.90 -2.95 (m 2H), 2.21 (s, 1.5H), 2.18 (s, 1.5H), 1.37-1.43 (m, 6H); ^{13}C NMR (101 MHz, $CDCl_3$) δ 169.33, 169.09, 167.15, 167.08, 130.37, 128.40, 61.03, 60.90, 44.96, 43.86, 40.70, 39.23, 27.19, 26.28,

21.88, 21.44, 14.32, 14.23, 13.84; LC/MS (ESI$^+$, MeCN/H_2O): *m/z*: calcd for $C_{15}H_{22}N_3O_3S_2$: 356.10 [M+H]$^+$; found: 356.1.

*Ethyl 6-acetyl-2-(3-propylthioureido)-4,5,6,7-tetrahydrothieno[2,3-c]pyridine-3-carboxylate **(24d)***. Following the same procedure for the synthesis of **24aa**, ethyl 6-acetyl-2-isothiocyanato-4,5,6,7-tetrahydrothieno[2,3-*c*]pyridine-3-carboxylate **23a** (708 mg, 2.28 mmol, 1.0 equiv.) and propan-1-amine (0.28 mL, 3.42 mmol, 1.5 equiv.) in EtOH (22 mL) gave the title product **24d** (785 mg, 93%) as a white solid; R_f = 0.55 (DCM/MeOH 20/1); ^{1}H NMR (400 MHz, MeOD, mixture of the rotamers) δ 12.08 (br s, 1H), 6.50 (br s, 1H), 4.68 (s, 1H), 4.54 (s, 1H), 4.35 (q, *J* = 7.2 Hz, 2H), 3.83 (t, *J* = 6.0 Hz, 1H), 3.70 (t, *J* = 6.0 Hz, 1H), 3.37-3.48 (m, 2H), 2.96 (t, *J* = 6.0 Hz, 1H), 2.94 (t, *J* = 6.0 Hz, 1H), 2.21 (s, 1.5H), 2.18 (s, 1.5H), 1.72-1.75 (m, 2H), 1.37-1.39 (m, 3H), 1.05 (t, *J* = 7.2 Hz, 3H); ^{13}C NMR (101 MHz, $CDCl_3$) δ 169.42, 169.14, 60.99, 60.86, 44.94, 43.87, 40.71, 39.26, 27.18, 26.26, 21.98, 21.90, 21.45, 14.32, 14.22, 11.42; LC/MS (ESI$^+$, MeCN/H_2O): *m/z*: calcd for $C_{16}H_{24}N_3O_3S_2$: 370.12 [M+H]$^+$; found: 370.0.

*Ethyl 6-acetyl-2-(3-isobutylthioureido)-4,5,6,7-tetrahydrothieno[2,3-c]pyridine-3-carboxylate **(24e)***. Following the same procedure for the synthesis of **24aa**, ethyl 6-acetyl-2-isothiocyanato-4,5,6,7-tetrahydrothieno[2,3-*c*]pyridine-3-carboxylate **23a** (100 mg, 0.32 mmol, 1.0 equiv.) and 2-methylpropan-1-amine (48 μL, 0.48 mmol, 1.5 equiv.) in EtOH (3 mL) gave the title product **24e** (108 mg, 87%) as a white solid; R_f = 0.60 (DCM/MeOH 20/1); ^{1}H NMR (400 MHz, $CDCl_3$, mixture of the rotamers) δ 12.10 (br s, 1H), 6.43-6.50 (br m, 1H), 4.68 (s, 1H), 4.55 (s, 1H), 4.34 (q, *J* = 6.9 Hz, 2H), 3.84 (t, *J* = 6.0 Hz, 1H), 3.69 (t, *J* = 6.0 Hz, 1H), 3.33 (m, 2H), 2.95 (t, *J* = 6.0 Hz, 1H), 2.90 (t, *J* = 6.0 Hz, 1H), 2.21 (s, 1.5H), 2.18 (s, 1.5H), 2.01 (sextet, *J* = 6.9 Hz, 1H), 1.40 (q, *J* = 7.2 Hz, 3H), 1.04 (d, *J* = 5.6 Hz, 6H); ^{13}C NMR (101 MHz, $CDCl_3$) δ 169.36, 169.10, 166.99, 152.95, 130.83, 128.39, 60.99, 60.86, 44.94, 43.86, 40.70, 39.25, 28.06, 27.19, 26.27,

21.90, 21.45, 20.22, 14.32, 14.23; LC/MS (ESI$^+$, MeCN/H_2O): *m/z*: calcd for $C_{17}H_{25}N_3O_3S_2$: 384.13 $[M+H]^+$; found: 384.1.

*Ethyl 6-acetyl-2-(3-butylthioureido)-4,5,6,7-tetrahydrothieno[2,3-c]pyridine-3-carboxylate (**24f**).* Following the same procedure for the synthesis of **24aa**, ethyl 6-acetyl-2-isothiocyanato-4,5,6,7-tetrahydrothieno[2,3-*c*]pyridine-3-carboxylate **23a** (100 mg, 0.32 mmol, 1.0 equiv.) and butan-1-amine (47 µL, 0.48 mmol, 1.5 equiv.) in EtOH (3 mL) gave the title product **24f** (70 mg, 57%) as a white solid; R_f = 0.55 (DCM/MeOH 20/1); ^{1}H NMR (400 MHz, $CDCl_3$, mixture of the rotamers) δ 12.08 (br s, 1H), 6.45 (br m, 1H), 4.68 (s, 1H), 4.55 (s, 1H), 4.35 (q, *J* = 6.0 Hz, 2H), 3.84 (t, *J* = 6.0 Hz, 1H), 3.69 (t, *J* = 6.0 Hz, 1H), 3.52 (m, 2H), 2.89-2.95 (m, 2H), 2.21 (s, 1.5H), 2.18 (s, 1.5H), 1.67-1.71 (m, 2H), 1.37-1.50 (m, 5H), 1.00 (t, *J* = 6.0 Hz, 3H); ^{13}C NMR (101 MHz, $CDCl_3$) δ 177.11, 169.38, 169.12, 167.05, 152.74, 130.32, 128.36, 60.98, 60.85, 44.94, 43.87, 40.72, 39.26, 30.64, 27.19, 26.27, 21.88, 21.43, 20.06, 14.32, 14.22, 13.68; LC/MS (ESI$^+$, MeCN/H_2O): *m/z*: calcd for $C_{17}H_{25}N_3O_3S_2$: 384.13 $[M+H]^+$; found: 384.1.

*Ethyl 6-acetyl-2-(3-isopropylthioureido)-4,5,6,7-tetrahydrothieno[2,3-c]pyridine-3-carboxylate (**24g**).* Following the same procedure for the synthesis of **24aa**, ethyl 6-acetyl-2-isothiocyanato-4,5,6,7-tetrahydrothieno[2,3-*c*]pyridine-3-carboxylate **23a** (100 mg, 0.32 mmol, 1.0 equiv.) and propan-2-amine (42 µL, 0.48 mmol, 1.5 equiv.) in EtOH (3 mL) gave the title product **24g** (115 mg, 99%) as a white solid; R_f = 0.70 (DCM/MeOH 20/1); ^{1}H NMR (400 MHz, $CDCl_3$, mixture of the rotamers) δ 12.00 (br s, 1H), 6.35-6.44 (br m, 1H), 4.66 (s, 1H), 4.52 (s, 1H), 4.29-4.36 (m, 2H), 4.23 (s, 1H), 3.85 (t, *J* = 6.0 Hz, 1H), 3.67 (t, *J* = 6.0 Hz, 1H), 2.92 (t, *J* = 6.0 Hz, 1H), 2.86 (t, *J* = 6.0 Hz, 1H), 2.19 (s, 1.5H), 2.16 (s, 1.5H), 1.31-1.41 (m, 9H); ^{13}C NMR (101 MHz, $CDCl_3$) δ 176.07, 169.41, 169.13, 167.01, 152.80, 152.60, 130.28, 128.32, 60.98, 60.83, 46.44, 46.38,

44.94, 43.88, 40.71, 39.28, 27.19, 26.26, 22.32, 22.29, 21.89, 21.44, 14.31, 14.21; LC/MS (ESI$^+$, MeCN/H_2O): *m/z*: calcd for $C_{16}H_{24}N_3O_3S_2$: 370.12 $[M+H]^+$; found: 370.1.

*Procedure for the synthesis of 7-acetyl-3-(4-methoxyphenyl)-2-sulfanylidene-1,5,6,8-tetrahydropyrido[2,3]thieno[2,4-b]pyrimidin-4-one (**25aa**).* A solution of ethyl 6-acetyl-2-(3-(4-methoxyphenyl)thioureido)-4,5,6,7-tetrahydrothieno[2,3-*c*]pyridine-3-carboxylate **24aa** (2.56 g, 5.92 mmol) in the 1:3 mixture (59 mL) of 10% NaOH and MeOH was heated under the reflux for 3 h, and cooled to room temperature. The reaction mixture was acidified with 2 N HCl to pH ~ 3, and then the precipitated was filtered off and washed with H_2O to give the title product **25aa** as a white solid (2.13 g, 93%); R_f = 0.40 (CH_2Cl_2/MeOH 10/1); ^1H NMR (400 MHz, DMSO-d_6, mixture of the rotamers) δ 13.70 (s, 1H), 7.11 (d, *J* = 8.9 Hz, 2H), 6.98 (d, *J* = 8.9 Hz, 2H), 4.68 (s, 0.7H), 4.63 (s, 1.3H), 4.07 (q, *J* = 7.0 Hz, 2H), 3.72-3.67 (m, 2H), 2.89 (m, 1.3H), 2.78 (m, 0.7H), 2.11 (s, 2H), 2.08 (s, 1H), 1.37 (t, *J* = 7.0 Hz, 3H); ^{13}C NMR (75 MHz, DMSO-d_6) *δ* 175.56, 169.24, 158.49, 157.46, 150.42, 132.06, 130.38, 125.58, 116.09, 115.00, 63.67, 43.10, 26.16, 22.41, 21.66, 15.14..

*7-Acetyl-3-(4-ethoxyphenyl)-2-sulfanylidene-1,5,6,8-tetrahydropyrido[2,3]thieno[2,4-b] pyrimidin-4-one (**25ab**).* Following the same procedure for the synthesis of **25aa**, ethyl 6-acetyl-2-(3-(4-ethoxyphenyl)thioureido)-4,5,6,7-tetrahydrothieno[2,3-*c*]pyridine-3-carboxylate **24ab** (2.37 g, 5.29 mmol) in the 1:3 mixture (50 mL) of 10% NaOH and MeOH gave the title product **25ab** (2.07 g, 97%) as a white solid; R_f = 0.10 (*n*-hexane/EtOAc 1/1); ^1H NMR (400 MHz, DMSO-d_6, mixture of the rotamers) *δ* 13.65 (br s, 1H), 7.13 (d, J = 8.8 Hz, 2H), 7.00 (d, J = 8.8 Hz, 2H), 4.68 (s, 0.7H), 4.63 (s, 1.3H), 3.81 (s, 3H), 3.72-3.67 (m, 2H), 2.90 (m, 1.3H), 2.78 (m, 0.7H), 2.11 (s, 2H), 2.08 (s, 1H) ; ^{13}C NMR (75 MHz, DMSO-d_6) *δ* 175.63, 169.24, 160.99, 159.22, 157.47, 150.63, 132.24, 130.40, 125.55, 116.11, 114.62, 55.75, 43.12, 26.54, 22.18, 21.64.

*3-(4-Ethoxyphenyl)-7-methyl-2-sulfanylidene-1,5,6,8-tetrahydropyrido[2,3]thieno[2,4-b]pyrimidin-4-one (**25b**)*. Following the same procedure for the synthesis of **25aa**, 2-(3-(4-ethoxyphenyl)thioureido)-6-methyl-4,5,6,7-tetrahydrothieno[2,3-*c*]pyridine-3-carboxylate **24b** (130 mg, 0.31 mmol) in the 1:3 mixture (3.1 mL) of 10% NaOH and MeOH gave the title product **25b** (114 mg, 98%) as a yellow solid; R_f = 0.10 (CH_2Cl_2/MeOH: 10/1); ^{1}H NMR (400 MHz, DMSO) δ 13.78 (br s, 1H), 10.73 (br s, 1H), 7.12 (d, J = 8.8 Hz, 2H), 6.98 (d, J = 8.8 Hz, 2H), 4.37-4.52 (m, 2H), 4.07 (q, J = 6.0 Hz, 2H), 3.38-3.63 (m, 2H), 3.11 (s, 2H), 2.93 (s, 3H), 1.36 (t, J = 6.0 Hz, 3H); ^{13}C NMR (75 MHz, DMSO-d_6) δ 175.88, 158.57, 157.43, 151.29, 131.86, 130.36, 128.79, 120.07, 115.47, 115.06, 63.70, 50.66, 49.98, 42.15, 22.70, 15.16.

*7-Acetyl-3-ethyl-2-thioxo-2,3,5,6,7,8-hexahydropyrido[4',3':4,5]thieno[2,3-d]pyrimidin-4(1H)-one (**25c**)*. Following the same procedure for the synthesis of **25aa**, the title product **25c** (36 mg, 94%) was produced as a white solid; R_f = 0.30 (CH_2Cl_2/MeOH: 10/1); ^{1}H NMR (300 MHz, $CDCl_3$, mixture of the rotamers) δ 13.59 (s, 1H) 4.64-4.60 (m, 2H), 4.41-4.36 (m, 2H), 3.69 (t, J = 6.4 Hz, 2H), 2.95-2.83 (m, 2H), 2.11-2.06 (m, 3H), 1.24-1.81 (m, 3H). ^{13}C NMR (75 MHz, DMSO-d_6) δ 173.77, 169.23, 156.64, 149.95, 130.16, 125.68, 115.70, 43.11, 26.22, 25.25, 22.23, 21.67, 12.23.

*7-Acetyl-3-propyl-2-thioxo-2,3,5,6,7,8-hexahydropyrido[4',3':4,5]thieno[2,3-d]pyrimi-din-4(1H)-one (**25d**)*. Following the same procedure for the synthesis of **25aa**, the title product **25d** (671 mg, 98%) was produced as a white solid; R_f = 0.20 (CH_2Cl_2/MeOH: 10/1); ^{1}H NMR (400 MHz, $CDCl_3$, mixture of the rotamers) δ 11.09 (br s, 1H), 4.78 (s, 1.6H), 4.76 (s, 2H), 4.4.42 (t, J = 7.8 Hz, 2H), 3.75-3.78 (m, 2H), 3.08-3.13 (m, 2H), 2.27 (s, 2H), 2.21 (s, 1H), 1.79-1.85 (m, 2H), 1.02 (t, J = 6.9 Hz, 3H); ^{13}C NMR (75 MHz, DMSO-d_6) δ 173.53, 168.70, 156.32, 149.40, 129.67, 125.20, 124.81, 115.13, 46.74, 44.27, 42.60, 37.73, 25.71, 24.74, 21.71, 21.15, 19.47, 11.08.

*7-Acetyl-3-isobutyl-2-thioxo-2,3,5,6,7,8-hexahydropyrido[4',3':4,5]thieno[2,3-d]pyrimi-din-4(1H)-one (**25e**)*. Following the same procedure for the synthesis of **25aa**, the title product **25e** (86 mg, 91%) was produced as a white solid; R_f = 0.20 (CH_2Cl_2/MeOH: 10/1); ^{1}H NMR (300 MHz, $CDCl_3$, mixture of the rotamers) δ 12.50 (br s, 1H), 4.79 (s, 1.6H), 4.63 (s, 0.4H), 4.35 (d, J = 7.2 Hz, 2H), 3.93 (t, J = 6.0 Hz, 0.4H), 3.78 (t, J = 6.0 Hz, 1.6H), 3.09-3.16 (m, 2H), 2.45-2.51 (m, 1H), 2.31 (s, 2H), 2.25 (s, 1H), 1.43 (d, J = 8.7 Hz, 6H). ^{13}C NMR (75 MHz, $CDCl_3$) δ 174.95, 170.32, 157.36, 149.16, 130.34, 124.84, 116.05, 52.60, 43.50, 41.02, 26.53, 26.00, 22.06, 21.55, 20.15.

*7-Acetyl-3-butyl-2-thioxo-2,3,5,6,7,8-hexahydropyrido[4',3':4,5]thieno[2,3-d]pyrimidin-4(1H)-one (**25f**)*. Following the same procedure for the synthesis of **25aa**, the title product **25f** (57 mg, 93%) was produced as a white solid; R_f = 0.20 (CH_2Cl_2/MeOH: 10/1); ^{1}H NMR (300 MHz, $CDCl_3$, mixture of the rotamers) δ 12.03 (br s, 1H), 4.79 (s, 1.6H), 4.62 (s, 0.4H), 4.47 (s, 2H), 3.93 (t, J = 6.0 Hz, 0.4H), 3.78 (t, J = 6.0 Hz, 1.6H), 3.08-3.15 (m, 2H), 2.33 (s, 2H), 2.23 (s, 1H), 1.68-1.78 (m, 4H), 1.41-1.49 (m, 2H), 1.02 (t, J = 6.9 Hz, 3H). ^{13}C NMR (75 MHz, $CDCl_3$) δ 174.39, 170.20, 156.88, 148.98, 130.33, 124.85, 116.16, 46.51, 43.47, 40.95, 28.63, 25.98, 21.53, 20.24, 13.78.

*7-Aetyl-3-isopropyl-2-thioxo-2,3,5,6,7,8-hexahydropyrido[4',3':4,5]thieno[2,3-d]pyrimi-din-4(1H)-one (**25g**)*. Following the same procedure for the synthesis of **25aa**, the title product **25g** (63 mg, 63%) was produced as a white solid; R_f = 0.20 (CH_2Cl_2/MeOH: 10/1); ^{1}H NMR (300 MHz, $CDCl_3$, mixture of the rotamers) δ 13.53 (br s, 1H), 5.98-6.02 (m, 1H), 4.64 (s, 0.7H), 4.60 (s, 1.3H), 3.67-3.72 (m, 2H), 2.82-2.94 (m, 2H), 2.11 (s, 2H), 2.07 (s, 1H), 1.48 (d, J = 6.8 Hz, 6H). ^{13}C NMR (75 MHz, DMSO-d_6) δ 174.98, 169.22, 157.38, 149.34, 130.58, 130.18, 125.70, 125.28, 44.78, 4314, 38.26, 26.30, 25.34, 22.22, 21.66, 19.05.

*N-(2,4-Difluorophenyl)-2-[[7-ethanoyl-3-(4-methoxyphenyl)-4-oxidanylidene-6,8-dihydro-5H-pyrido[2,3]thieno[2,4-b]pyrimidin-2-yl]sulfanyl]ethanamide (**14 (VH062)**)*. To a stirred solution of 7-acetyl-3-(4-methoxyphenyl)-2-sulfanylidene-1,5,6,8-tetrahydropyrido[2,3] thieno[2,4-*b*]pyrimidin-4-one **25b** (600 mg, 1.55 mmol) and 2-bromo-*N*-(2,4-difluorophenyl) acetamide (350 mg, 1.70 mmol) in MeCN was added Et_3N (0.435 mL, 3.10 mmol) dropwise. The reaction was heated under the reflux for 4 h, and then cooled to room temperature. The reaction mixture was extracted with EtOAc (3 × ~ 50 mL) and washed with H_2O (~ 30 mL). The organic layer was dried over anhydrous $MgSO_4$, and concentrated in vacuo. The residue was purified by column chromatography (SiO_2, CH_2Cl_2/MeOH: 10/1) to yield the title product (752 mg, 87%) as a white solid; R_f = 0.60 (CH_2Cl_2/MeOH: 10/1); ^{1}H NMR (400 MHz, $CDCl_3$, mixture of the rotamers) δ 9.28 (br s, 1H), 8.28-8.33 (m, 1H), 7.23 (dd, J = 6.8, 2.0 Hz, 2H), 7.07 (dd, J = 6.8, 2.0 Hz, 2H), 6.85-6.91 (m, 2H), 4.87 (s, 1.5H), 4.73 (s, 0.5H), 3.89-3.94 (m, 5.5H), 3.78 (t, J = 5.6 Hz, 1.5H), 3.09-3.16 (m, 2H), 2.24 (s, 3H); ^{13}C NMR (75 MHz, $CDCl_3$, mixture of the rotamers) δ 169.34, 168.01 (d, ^1J = 230.25 Hz), 161.05, 160.69 (d, ^1J = 215.25 Hz), 159.00, 158.18, 154.04, 150.77, 131.67, 130.04, 129.63, 129.04, 127.10, 127.05, 122.86 (d, ^3J = 6.75 Hz), 122.77, 122.29 (d, ^3J = 6.00 Hz), 119.00, 115.29, 111.24 (d, ^2J = 21.75 Hz), 103.56 (t, ^2J = 24.75 Hz), 55.65, 45.31, 43.63, 41.19, 38.77, 36.75, 29.73, 26.11, 25.23, 22.13, 21.50; HRMS (ESI$^+$): *m/z*: calcd for $C_{26}H_{23}F_2N_4O_4S_2$ 557.1051 $[M + H]^+$; found: 557.1123.

*N-(3,4-Difluorophenyl)-2-[[7-ethanoyl-3-(4-methoxyphenyl)-4-oxidanylidene-6,8-dihydro-5H-pyrido[2,3]thieno[2,4-b]pyrimidin-2-yl]sulfanyl]ethanamide (**27**)*. Following the same procedure for the synthesis of **14**, 7-acetyl-3-(4-methoxyphenyl)-2-thioxo-2,3,5,6,7,8-hexahydropyrido[4',3':4,5]thieno[2,3-*d*]pyrimidin-4(1*H*)-one **25aa** (70 mg, 0.18 mmol, 1.0 equiv.), 2-bromo-*N*-(3,4-difluorophenyl)acetamide (41 mg, 0.20 mmol, 1.1 equiv.), and Et_3N (0.051 mL,

0.36 mmol, 2.0 equiv.) gave the title product (90 mg, 90%) as a white solid; R_f = 0.65 (CH_2Cl_2/MeOH 10/1); ^{1}H NMR (300 MHz, CDCl3, mixture of the rotamers) δ 9.34 (s, 1H), 7.69-7.63 (m, 1H), 7.20 (d, J = 8.8, 2H), 7.10-7.03 (m, 4H), 4.85-4.71 (m, 2H), 3.87 (s, 3H), 3.81 (s, 3H), 3.76 (t, *J* = 5.6 Hz, 2H), 3.12-3.06 (m, 2H), 2.21 (s, 3H); ^{13}C NMR (75 MHz, CDCl3, mixture of the rotamers) δ 169.53, 169.39, 166.23, 161.67, 161.14, 159.66, 158.01, 151.85, 134.42, 134.34, 132.27, 130.23, 129.97, 128.63, 127.01, 120.30, 119.10, 117.42, 117.18, 115.33, 115.02, 114.95, 114.90, 109.32, 105.13, 59.63, 45.87, 45.34, 43.59, 41.19, 38.78, 36.80, 26.19, 15.30, 22.07, 21.48; HRMS (ESI$^+$): *m/z*: calcd for $C_{26}H_{23}F_2N_4O_4S_2$ 557.1123 [M + H]$^+$; found: 557.1123.

*2-((7-Acetyl-3-(4-ethoxyphenyl)-4-oxo-3,4,5,6,7,8-hexahydropyrido[4',3':4,5]thieno[2,3-d]pyrimidin-2-yl)thio)-N-(2-(piperidin-1-yl)ethyl)acetamide (**28**).* Following the same procedure for the synthesis of **14**, 7-acetyl-3-(4-ethoxyphenyl)-2-thioxo-2,3,5,6,7,8-hexahydropyrido [4',3':4,5]thieno[2,3-*d*]pyrimidin-4(1*H*)-one **25ab** (50 mg, 0.12 mmol, 1.0 equiv.), 2-bromo-*N*-(2-(piperidin-1-yl)ethyl)acetamide (34 mg, 0.13 mmol, 1.1 equiv.), and Et_3N (0.034 mL, 0.24 mmol, 2.0 equiv.) gave the title product (48 mg, 70%) as a white solid; R_f = 0.40 (CH_2Cl_2/MeOH 20/1); ^{1}H NMR (300 MHz, $CDCl_3$, mixture of the rotamers) δ 7.22 (d, *J* = 9.0 Hz, 2H), 7.05 (d, *J* = 9.0 Hz, 2H), 4.82 (s, 1.5H), 4.69 (s, 0.5H), 4.11 (q, *J* = 6.9 Hz, 2H), 3.91 (t, *J* = 5.7 Hz, 0.5H), 3.78 (s, 2H), 3.74 (t, *J* = 5.7 Hz, 1.5H), 3.08-3.13 (m, 2H), 2.50 (t, *J* = 6.0 Hz, 2H), 2.41 (m, 4H), 2.24 (s, 3H), 1.48 (t, *J* = 6.9 Hz, 3H), 1.28 (m, 2H), 1.25 (m, 2H), 0.88-0.91 (m, 4H); ^{13}C NMR (75 MHz, $CDCl_3$, mixture of the rotamers) δ 169.50, 167.67, 162.73, 160.31, 158.51, 158.27, 130.05 (2C), 129.60, 128.36, 127.19, 118.67, 115.64 (2C), 63.88, 57.03, 54.24, 43.65, 41.24, 36.08, 29.70, 26.11, 25.09, 23.78, 22.69, 22.04, 21.45, 14.73; HRMS (ESI$^+$): *m/z*: calcd for $C_{28}H_{36}N_5O_4S_2$ 570.2130 [M + H]$^+$; found: 570.2194.

*2-((7-Acetyl-3-(4-ethoxyphenyl)-4-oxo-3,4,5,6,7,8-hexahydropyrido[4',3':4,5]thieno[2,3-d]pyrimidin-2-yl)thio)-N-(3-(pyrrolidin-1-yl)propyl)acetamide (**29**).* Following the same procedure for the synthesis of **14**, 7-acetyl-3-(4-ethoxyphenyl)-2-thioxo-2,3,5,6,7,8-hexahydropyrido[4',3':4,5]thieno[2,3-*d*]pyrimidin-4(1*H*)-one **25ab** (50 mg, 0.12 mmol, 1.0 equiv.), 2-bromo-*N*-(3-(pyrrolidin-1-yl)propyl)acetamide (34 mg, 0.13 mmol, 1.1 equiv.), and Et_3N (0.034 mL, 0.24 mmol, 2.0 equiv.) gave the title product (44 mg, 68%) as a white solid; R_f = 0.40 (CH_2Cl_2/MeOH 20/1); ^1H NMR (300 MHz, $CDCl_3$, mixture of the rotamers) δ 7.85 (br t, J = 5.7 Hz, 1H), 7.22 (d, J = 9.0 Hz, 2H), 7.05 (d, J = 9.0 Hz, 2H), 4.83 (s, 1.5H), 4.68 (s, 0.5H), 4.12 (q, J = 6.9 Hz, 2H), 3.77 (t, J = 6.0 Hz, 0.5H), 3.39 (t, J = 6.0 Hz, 1.5H), 3.35 (s, 2H), 3.14-3.16 (m, 2H), 3.12 (m, 2H), 2.55-2.61 (m, 6H), 2.24 (s, 3H), 1.73-1.80 (m, 6H), 1.49 (t, J = 6.9 Hz, 3H); ^{13}C NMR (75 MHz, $CDCl_3$, mixture of the rotamers) δ 169.55, 167.64, 162.64, 160.28, 158.55, 158.48, 130.11 (2C), 129.71, 128.17, 127.19, 118.71, 115.61 (2C), 63.87, 53.76, 53.43, 43.65, 41.23, 38.81, 37.95, 36.32, 29.69, 26.53, 23.37, 22.05, 21.44, 14.74; HRMS (ESI$^+$): *m/z*: calcd for $C_{28}H_{36}N_5O_4S_2$ 570.2130 $[M + H]^+$; found: 570.2194.

*2-((7-Acetyl-3-(4-ethoxyphenyl)-4-oxo-3,4,5,6,7,8-hexahydropyrido[4',3':4,5]thieno[2,3-d]pyrimidin-2-yl)thio)-N-(1-methylpiperidin-4-yl)acetamide (**30**).* Following the same procedure for the synthesis of **14**, 7-acetyl-3-(4-ethoxyphenyl)-2-thioxo-2,3,5,6,7,8-hexahydropyrido[4',3':4,5]thieno[2,3-*d*]pyrimidin-4(1*H*)-one **25ab** (50 mg, 0.13 mmol, 1.0 equiv.), 2-bromo-*N*-(1-methylpiperidin-4-yl)acetamide (26 mg, 0.14 mmol, 1.1 equiv.), and Et_3N (35 μL, 0.26 mmol, 2.0 equiv.) in MeCN (1.3 mL) gave the title product (26 mg, 38%) as a white solid; R_f = 0.50 (CH_2Cl_2/MeOH 10/1); ^1H NMR (300 MHz, $CDCl_3$, mixture of the rotamers) δ 7.20 (d, J = 8.7 Hz, 2H), 7.06 (d, J = 8.7 Hz, 2H), 6.73 (br d, J = 7.2 Hz, 1H), 4.84 (s, 1.5H), 4.70 (s, 0.5H), 4.12 (q, J = 6.9 Hz, 2H), 3.92 (t, J = 5.4 Hz, 0.5H), 3.75-3.79 (m, 2.5H), 3.69 (s, 2H), 3.09-

3.15 (m, 2H), 2.75-2.78 (m, 2H), 2.24-2.29 (m, 2H), 2.16 (s, 3H), 2.13 (s, 3H), 2.06 (t, J = 10.8 Hz, 2H), 1.93-1.97 (m, 2H), 1.86 (m, 2H), 1.49 (t, J = 7.2 Hz, 3H); ^{13}C NMR (75 MHz, $CDCl_3$, mixture of the rotamers) δ 169.48, 167.25, 162.18, 160.37, 158.87, 129.95 (2C), 129.81, 128.58, 127.02, 118.77, 115.69 (2C), 63.90 (2C), 54.18, 46.15 (2C), 43.61, 41.19, 36.10, 31.95 (2C), 26.14, 21.48, 14.74; HRMS (ESI$^+$): *m/z*: calcd for $C_{27}H_{34}N_5O_4S_2$ 556.2047 [M + H]$^+$; found: 556.2049.

*2-((7-Acetyl-3-(4-ethoxyphenyl)-4-oxo-3,4,5,6,7,8-hexahydropyrido[4',3':4,5]thieno[2,3-d]pyrimidin-2-yl)thio)-N-benzylacetamide (**31**).*Following the same procedure for the synthesis of **14**, 7-acetyl-3-(4-ethoxyphenyl)-2-thioxo-2,3,5,6,7,8-hexahydropyrido[4',3':4,5]thieno[2,3-*d*]pyri-midin-4(1*H*)-one **25ab** (50 mg, 0.13 mmol, 1.0 equiv.), *N*-benzyl-2-bromoacetamide (26 mg, 0.14 mmol, 1.1 equiv.), and Et_3N (35 μL, 0.26 mmol, 2.0 equiv.) in MeCN (1.3 mL) gave the title product (59 mg, 88%) as a white solid; R_f = 0.30 (CH_2Cl_2/MeOH 10/1); ^{1}H NMR (300 MHz, $CDCl_3$, mixture of the rotamers) δ 7.28-7.34 (m, 5H), 7.18 (d, J = 8.7 Hz, 2H), 7.01-7.07 (m, 3H), 4.82 (s, 1.5H), 4.69 (s, 0.5H), 4.47 (d, J = 6.6 Hz, 2H), 4.12 (q, J = 7.2 Hz, 2H), 3.92 (t, J = 5.7 Hz, 0.5H), 3.82 (s, 2H), 3.77 (t, J = 5.7 Hz, 1.5H), 3.11-3.15 (m, 2H), 2.25 (s, 2.25H), 2.22 (s, 0.75H), 1.49 (t, J = 7.2 Hz, 3H); ^{13}C NMR (75 MHz, $CDCl_3$, mixture of the rotamers) δ 169.53, 167.62, 162.37, 160.37, 158.52, 158.35, 137.83, 129.97 (2C), 129.63, 128.75 (2C), 128.57, 127.68, 127.59 (2C), 127.04, 118.74, 115.67 (2C), 63.91, 43.94, 43.59, 41.20, 36.04, 26.10, 21.51, 14.76; HRMS (ESI$^+$): *m/z*: calcd for $C_{28}H_{29}N_4O_4S_2$ 549.1625 [M + H]$^+$; found: 549.1627.

*2-((7-Acetyl-3-(4-ethoxyphenyl)-4-oxo-3,4,5,6,7,8-hexahydropyrido[4',3':4,5]thieno[2,3-d]pyrimidin-2-yl)thio)-N-benzyl-N-methylacetamide (**32**).* Following the same procedure for the synthesis of **14**, 7-acetyl-3-(4-ethoxyphenyl)-2-thioxo-2,3,5,6,7,8-hexahydropyrido[4',3':4,5] thieno[2,3-*d*]pyrimidin-4(1*H*)-one **25ab** (50 mg, 0.13 mmol, 1.0 equiv.), *N*-benzyl-2-bromo-*N*-methylacetamide (27 mg, 0.14 mmol, 1.1 equiv.), and Et_3N (35 μL, 0.26 mmol, 2.0 equiv.) in

MeCN (1.3 mL) gave the title product (68 mg, 97%) as a white solid; R_f = 0.70 (CH_2Cl_2/MeOH 20/1); ^{1}H NMR (300 MHz, $CDCl_3$, mixture of the rotamers) δ 7.32-7.46 (m, 3H), 7.20-7.28 (m, 4H), 7.03-7.07 (m, 2H), 4.83 (s, 1.3H), 4.73 (s, 0.7H), 4.65 (s, 2H), 4.15 (q, J = 7.8 Hz, 2H), 4.11 (s, 3H), 3.95 (t, J = 5.7 Hz, 0.7H), 3.77 (t, J = 5.7 Hz, 1.3H), 3.04-3.13 (m, 4H), 3.02 (s, 2H), 2.99 (s, 1H), 1.49 (t, J = 6.9 Hz, 3H); ^{13}C NMR (75 MHz, $CDCl_3$, mixture of the rotamers) δ 169.60, 169.35, 167.41, 167.26, 166.74, 162.99, 162.88, 160.23, 158.78, 158.63, 136.66, 136.09, 130.06 (2C), 129.54, 129.11, 128.73, 128.04 (2C), 128.01, 127.94, 127.65, 127.57, 127.35, 127.30, 126.50, 126.04, 118.52, 115.65 (2C), 63.85, 53.89, 53.69, 51.56, 51.34, 45.32, 43.66, 41.45, 41.16, 36.41, 36.07, 35.29, 35.07, 34.62, 34.40, 26.11, 22.06, 21.48, 14.78; HRMS (ESI$^+$): *m/z*: calcd for $C_{29}H_{31}N_4O_4S_2$ 563.1781 [M + H]$^+$; found: 563.1781.

*2-((7-Acetyl-3-(4-ethoxyphenyl)-4-oxo-3,4,5,6,7,8-hexahydropyrido[4',3':4,5]thieno[2,3-d]pyrimidin-2-yl)thio)-N-benzyl-N-ethylacetamide (**33**).* Following the same procedure for the synthesis of **14**, 7-acetyl-3-(4-ethoxyphenyl)-2-thioxo-2,3,5,6,7,8-hexahydropyrido[4',3':4,5] thieno[2,3-*d*]pyrimidin-4(1*H*)-one **25ab** (40 mg, 0.10 mmol, 1.0 equiv.), *N*-benzyl-2-bromo-*N*-methylacetamide (26 mg, 0.11 mmol, 1.1 equiv.), and Et_3N (30 µL, 0.20 mmol, 2.0 equiv.) in MeCN (1.0 mL) gave the title product (49 mg, 83%) as a white solid; R_f = 0.50 (CH_2Cl_2/MeOH 20/1); ^{1}H NMR (300 MHz, $CDCl_3$, mixture of the rotamers) δ 7.36-7.42 (m, 1H), 7.21-7.32 (m, 6H), 7.02-0.07 (m, 2H), 4.82 (s, 1.3H), 4.73 (s, 0.7H), 4.63-4.68 (m, 2H), 3.99-4.13 (m, 4H), 3.91 (t, J = 5.2 Hz, 0.7H), 3.75 (t, J = 5.2 Hz, 1.3H), 3.48 (q, J = 6.3 Hz, 2H), 3.10-3.14 (m, 2H), 2.23 (s, 3H), 1.48 (t, J = 6.3 Hz, 3H), 1.30 (t, J = 6.9 Hz, 2H), 1.18 (t, J = 6.9 Hz, 1H); ^{13}C NMR (75 MHz, $CDCl_3$, mixture of the rotamers) δ 169.52, 169.25, 167.02, 166.90, 162.97, 162.89, 160.24, 158.86, 158.71, 158.63, 137.22, 136.61, 131.63, 130.05 (2C), 129.54, 128.64, 128.02, 127.96, 127.81, 127.45, 127.37, 126.46, 125.97, 118.52, 115.66 (2C), 63.85, 51.30, 48.77, 45.32, 43.67,

42.21, 42.03, 41.25, 38.83, 36.38, 36.20, 26.12, 25.27, 22.04, 21.47, 14.76, 13.89, 12.59; HRMS (ESI$^+$): *m/z*: calcd for $C_{30}H_{33}N_4O_4S_2$ 577.1938 [M + H]$^+$; found: 577.1937.

*2-((7-Acetyl-3-(4-ethoxyphenyl)-4-oxo-3,4,5,6,7,8-hexahydropyrido[4',3':4,5]thieno[2,3-d]pyrimidin-2-yl)thio)-N-(4-chlorobenzyl)acetamide (**34**).* Following the same procedure for the synthesis of **14**, 7-acetyl-3-(4-ethoxyphenyl)-2-thioxo-2,3,5,6,7,8-hexahydropyrido[4',3':4,5] thieno[2,3-*d*]pyrimidin-4(1*H*)-one **25ab** (40 mg, 0.10 mmol, 1.0 equiv.), 2-bromo-*N*-(4-chlorobenzyl)acetamide (25 mg, 0.11 mmol, 1.1 equiv.), and Et_3N (30 µL, 0.20 mmol, 2.0 equiv.) in MeCN (1.0 mL) gave the title product (58 mg, 97%) as a white solid; R_f = 0.25 (CH_2Cl_2/MeOH 20/1); ^{1}H NMR (400 MHz, $CDCl_3$, mixture of the rotamers) δ 7.20-7.25 (m, 3H), 7.16-7.18 (m, 4H), 7.03 (dd, *J* = 8.8 Hz, 2.7 Hz, 4.8 Hz, 2H), 4.78 (s, 1.4H), 4.66 (s, 0.6H), 4.39 (d, *J* = 5.6 Hz, 2H), 4.10 (q, *J* = 6.8 Hz, 2H), 3.88 (t, *J* = 5.6 Hz, 0.6H), 3.76 (s, 2H), 3.74 (t, *J* = 5.6 Hz, 1.4H), 3.03-3.10 (m, 2H), 2.20 (s, 3H), 1.47 (t, *J* = 6.8 Hz, 3H); ^{13}C NMR (75 MHz, $CDCl_3$, mixture of the rotamers) δ 169.53, 167.72, 162.25, 160.40, 158.48, 158.26, 136.50, 133.33, 129.96 (2C), 129.71, 128.99 (2C), 128.80 (2C), 128.59, 127.01, 118.75, 115.69 (2C), 63.92, 45.30, 43.58, 43.20, 41.19, 36.00, 26.10, 25.23, 22.05, 21.45, 14.73; HRMS (ESI$^+$): *m/z*: calcd for $C_{28}H_{28}ClN_4O_4S_2$ 583.1235 [M + H]$^+$; found: 583.1226.

*2-((7-Acetyl-3-(4-ethoxyphenyl)-4-oxo-3,4,5,6,7,8-hexahydropyrido[4',3':4,5]thieno [2,3-d]pyrimidin-2-yl)thio)-N-(4-chlorophenyl)acetamide (**35**).* Following the same procedure for the synthesis of **14**, 7-acetyl-3-(4-ethoxyphenyl)-2-thioxo-2,3,5,6,7,8-hexahydropyrido[4',3':4,5] thieno[2,3-*d*]pyrimidin-4(1*H*)-one **25ab** (40 mg, 0.10 mmol, 1.0 equiv.), 2-bromo-*N*-(4-chlorophenyl)acetamide (23 mg, 0.11 mmol, 1.1 equiv.), and Et_3N (30 µL, 0.20 mmol, 2.0 equiv.) in MeCN (1.0 mL) gave the title product (43 mg, 73%) as a white solid; R_f = 0.35 (CH_2Cl_2/MeOH 20/1); ^{1}H NMR (300 MHz, $CDCl_3$, mixture of the rotamers) δ 9.26 (s, 1H), 7.46 (d, *J* = 8.7 Hz,

2H), 7.29-7.26 (m, 2H), 7.18 (d, J = 7.6 Hz, 2H), 7.02 (d, J = 8.7 Hz, 2H), 4.85-4.71 (m, 2H), 4.09 (q, J = 6.9 Hz, 2H), 3.94–3.72 (m, 4H), 3.12-3.07 (m, 2H), 2.21 (s, 3H), 1.45 (t, J = 6.9 Hz, 3H).; ^{13}C NMR (75 MHz, $CDCl_3$, mixture of the rotamers) δ 169.60, 166.23, 161.89, 160.48, 159.54, 158.13, 136.50, 130.10, 129.94 (2C), 129.23, 129.05 (2C), 128.60, 126.82, 120.75 (2C), 118.97, 115.71 (2C), 63.93, 43.60, 41.20, 38.83, 37.02, 26.17, 22.14, 21.48, 14.74; HRMS (ESI$^+$): *m/z*: calcd for $C_{27}H_{26}ClN_4O_4S_2$ 569.1079 [M + H]$^+$; found: 569.1071.

*2-((7-Acetyl-3-(4-ethoxyphenyl)-4-oxo-3,4,5,6,7,8-hexahydropyrido[4',3':4,5]thieno [2,3-d]pyrimidin-2-yl)thio)-N-(4-methylbenzyl)acetamide (**36**).* Following the same procedure for the synthesis of **14**, 7-acetyl-3-(4-ethoxyphenyl)-2-thioxo-2,3,5,6,7,8-hexahydropyrido[4',3':4,5] thieno[2,3-*d*]pyrimidin-4(1*H*)-one **25ab** (40 mg, 0.10 mmol, 1.0 equiv.), 2-bromo-*N*-(4-methylbenzyl)acetamide (28 mg, 0.11 mmol, 1.1 equiv.), and Et_3N (30 µL, 0.20 mmol, 2.0 equiv.) in MeCN (1.0 mL) gave the title product (51 mg, 88%) as a white solid; R_f = 0.35 (CH_2Cl_2/MeOH 20/1); ^{1}H NMR (300 MHz, $CDCl_3$, mixture of the rotamers) δ 7.14-7.19 (m, 6H), 7.06 (d, J = 9.0 Hz, 2H), 6.88 (br t, J = 6.0 Hz, 1H), 4.83 (s, 1.5H), 4.69 (s, 0.5H), 4.43 (d, J = 6.0 Hz, 2H), 4.13 (q, J = 6.0 Hz, 2H), 3.88 (t, J = 6.0 Hz, 0.5H), 3.78 (s, 2H), 3.77 (t, J = 6.0 Hz, 1.5H), 3.09-3.14 (m, 2H), 2.36 (s, 3H), 2.25 (s, 3H), 1.49 (t, J = 6.0 Hz, 3H); ^{13}C NMR (75 MHz, $CDCl_3$, mixture of the rotamers) δ 169.52, 167.54, 162.33, 160.37, 158.51, 158.36, 137.30, 134.78, 129.96 (2C), 129.62, 129.40 (2C), 128.57, 127.60 (2C), 127.05, 118.74, 115.68 (2C), 63.91, 43.72, 43.61, 41.21, 36.02, 26.11, 21.50, 21.15, 14.75; HRMS (ESI$^+$): *m/z*: calcd for $C_{29}H_{31}N_4O_4S_2$ 563.1781 [M + H]$^+$; found: 563.1782.

*2-((7-Acetyl-3-(4-ethoxyphenyl)-4-oxo-3,4,5,6,7,8-hexahydropyrido[4',3':4,5]thieno [2,3-d]pyrimidin-2-yl)thio)-N,N-dimethylacetamide (**37**).* Following the same procedure for the synthesis of **14**, 7-acetyl-3-(4-ethoxyphenyl)-2-thioxo-2,3,5,6,7,8-hexahydropyrido[4',3':4,5]

thieno[2,3-*d*]pyrimidin-4(1*H*)-one **25ab** (40 mg, 0.10 mmol, 1.0 equiv.), 2-bromo-*N*,*N*-dimethylacetamide (19 mg, 0.11 mmol, 1.1 equiv.), and Et_3N (30 µL, 0.20 mmol, 2.0 equiv.) in MeCN (1.0 mL) gave the title product (46 mg, 93%) as a white solid; R_f = 0.20 (CH_2Cl_2/MeOH 20/1); ^{1}H NMR (300 MHz, $CDCl_3$, mixture of the rotamers) δ 7.19 (d, *J* = 8.9 Hz, 2H), 7.00 (d, *J* = 8.8 Hz, 2H), 4.79–4.65 (m, 2H), 4.19–3.97 (m, 4H), 3.91–3.69 (m, 2H), 3.18 (s, 2H), 3.13–3.08 (m, 2H), 2.98 (s, 3H), 2.20 (s, 3H), 1.45 (t, *J* = 7.0 Hz, 3H).; ^{13}C NMR (75 MHz, $CDCl_3$, mixture of the rotamers) δ 169.51, 167.12, 162.95, 160.21, 158.76, 158.62, 130.02 (2C), 129.54, 127.92, 127.31, 118.49, 115.63 (2C), 63.83, 60.39, 45.32, 43.65, 41.22, 38.80, 37.84, 36.16, 36.03, 26.11, 25.25, 21.48, 21.06, 14.76, 14.21; HRMS (ESI$^+$): *m/z*: calcd for $C_{23}H_{27}N_4O_4S_2$ 487.1468 [M + H]$^+$; found: 487.1462.

*2-((7-Acetyl-3-(4-ethoxyphenyl)-4-oxo-3,4,5,6,7,8-hexahydropyrido[4',3':4,5]thieno [2,3-d]pyrimidin-2-yl)thio)-N,N-diethylacetamide (**38**).* Following the same procedure for the synthesis of **14**, 7-acetyl-3-(4-ethoxyphenyl)-2-thioxo-2,3,5,6,7,8-hexahydropyrido[4',3':4,5]thieno[2,3-*d*]pyrimidin-4(1*H*)-one **25ab** (40 mg, 0.10 mmol, 1.0 equiv.), 2-bromo-*N*,*N*-diethylacetamide (22 mg, 0.11 mmol, 1.1 equiv.), and Et_3N (30 µL, 0.20 mmol, 2.0 equiv.) in MeCN (1.0 mL) gave the title product (40 mg, 75%) as a white solid; R_f = 0.25 (CH_2Cl_2/MeOH 20/1); ^{1}H NMR (300 MHz, $CDCl_3$, mixture of the rotamers) δ 7.23 (d, *J* = 9.0 Hz, 2H), 7.03 (d, *J* = 9.0 Hz, 2H), 4.80 (s, 1.6H), 4.66 (s, 0.4H), 4.10 (q, *J* = 6.0 Hz, 2H), 4.01 (s, 2H), 3.85 (t, *J* = 6.0 Hz, 0.4H), 3.74 (t, *J* = 6.0 Hz, 1.6H), 3.48 (q, *J* = 6.0 Hz, 2H), 3.41 (q, *J* = 6.0 Hz, 2H), 3.06-3.12 (m, 2H), 2.22 (s, 3H), 1.47 (t, *J* = 6.0 Hz, 3H), 1.32 (t, *J* = 6.0 Hz, 3H), 1.14 (t, *J* = 6.0 Hz, 3H); ^{13}C NMR (75 MHz, $CDCl_3$, mixture of the rotamers) δ 169.62, 166.16, 165.69, 163.67, 162.96, 160.50, 160.21, 158.93, 158.64, 130.03 (2C), 129.86, 129.52, 127.85, 127.35, 115.83, 115.63 (2C), 63.83, 43.68, 42.72, 41.26,

41.10, 40.63, 36.58, 36.26, 27.15, 26.10, 22.92, 21.41, 14.74, 14.47, 12.98; HRMS (ESI$^+$): *m/z*: calcd for $C_{25}H_{31}N_4O_4S_2$ 515.1781 [M + H]$^+$; found: 515.1775.

*2-((7-Acetyl-3-(4-ethoxyphenyl)-4-oxo-3,4,5,6,7,8-hexahydropyrido[4',3':4,5]thieno [2,3-d]pyrimidin-2-yl)thio)-N,N-dipropylacetamide (**39**).* Following the same procedure for the synthesis of **14**, 7-acetyl-3-(4-ethoxyphenyl)-2-thioxo-2,3,5,6,7,8-hexahydropyrido[4',3':4,5] thieno[2,3-*d*]pyrimidin-4(1*H*)-one **25ab** (40 mg, 0.10 mmol, 1.0 equiv.), 2-bromo-*N*,*N*-dipropylacetamide (25 mg, 0.11 mmol, 1.1 equiv.), and Et_3N (30 µL, 0.20 mmol, 2.0 equiv.) in MeCN (1.0 mL) gave the title product (39 mg, 70%) as a white solid; R_f = 0.30 (CH_2Cl_2/MeOH 20/1); ^{1}H NMR (300 MHz, $CDCl_3$, mixture of the rotamers) δ 7.28 (d, *J* = 9.0 Hz, 2H), 7.05 (d, *J* = 9.0 Hz, 2H), 4.82 (s, 1.5H), 4.69 (s, 0.5H), 4.12 (q, *J* = 6.0 Hz, 2H), 4.03 (s, 2H), 3.85 (t, *J* = 6.0 Hz, 0.5H), 3.76 (t, *J* = 6.0 Hz, 1.5H), 3.31-3.43 (m, 4H), 3.14-3.16 (m, 2H), 2.24 (s, 2.25H), 2.21 (s, 0.75H), 1.61-1.74 (m, 4H), 1.49 (t, *J* = 6.0 Hz, 3H), 1.03 (t, *J* = 6.0 Hz, 3H), 0.88 (t, *J* = 6.0 Hz, 3H); ^{13}C NMR (75 MHz, $CDCl_3$, mixture of the rotamers) δ169.49, 169.21, 166.41, 162.96, 160.19, 158.93, 158.66, 130.03, 129.551, 127.87, 127.37, 118.45, 115.62, 63.82, 48.30, 46.54, 43.66, 41.22, 36.38, 31.29, 29.74, 26.11, 21.48, 20.21, 20.16, 14.76, 13.89, 13.84.; HRMS (ESI$^+$): *m/z*: calcd for $C_{27}H_{35}N_4O_4S_2$ 543.2094 [M + H]$^+$; found: 543.2088.

*2-((7-Acetyl-3-(4-ethoxyphenyl)-4-oxo-3,4,5,6,7,8-hexahydropyrido[4',3':4,5]thieno [2,3-d]pyrimidin-2-yl)thio)-N,N-dibutylacetamide (**40**).* Following the same procedure for the synthesis of **14**, 7-acetyl-3-(4-ethoxyphenyl)-2-thioxo-2,3,5,6,7,8-hexahydropyrido[4',3':4,5]thieno[2,3-*d*]pyrimidin-4(1*H*)-one **25ab** (40 mg, 0.10 mmol, 1.0 equiv.), 2-bromo-*N*,*N*-dibutylacetamide (28 mg, 0.11 mmol, 1.1 equiv.), and Et_3N (30 µL, 0.20 mmol, 2.0 equiv.) in MeCN (1.0 mL) gave the title product (46 mg, 78%) as a white solid; R_f = 0.35 (CH_2Cl_2/MeOH 20/1); ^{1}H NMR (300 MHz, $CDCl_3$, mixture of the rotamers) δ 7.23 (d, *J* = 9.0 Hz, 2H), 7.03 (d, *J* = 9.0 Hz, 2H), 4.81 (s, 1.4H),

4.67 (s, 0.6H), 4.13 (q, J = 6.0 Hz, 2H), 4.02 (s, 2H), 3.85 (t, J = 6.0 Hz, 0.6H), 3.75 (t, J = 6.0 Hz, 1.4H), 3.32-3.43 (m, 4H), 2.93-3.12 (m, 2H), 2.22 (s, 3H), 1.68-1.75 (m, 2H), 1.42-1.56 (m,, 6H), 1.30 (t, J = 6.0 Hz, 3H), 1.03 (t, J = 6.0 Hz, 3H), 0.92 (t, J = 6.0 Hz, 3H); ^{13}C NMR (75 MHz, $CDCl_3$, mixture of the rotamers) δ 169.49, 169.21, 166.41, 162.96, 160.19, 158.93, 158.66, 130.03 (2C), 129.51, 127.87, 127.37, 118.45, 115.62 (2C), 63.82, 48.30, 46.54, 43.66, 41.22, 36.38, 31.29, 29.74, 26.11, 21.48, 20.21, 20.16, 14.76, 13.89, 13.84; HRMS (ESI$^+$): *m/z*: calcd for $C_{29}H_{39}N_4O_4S_2$ 571.2407 [M + H]$^+$; found: 571.2399.

*2-((7-Acetyl-3-(4-ethoxyphenyl)-4-oxo-3,4,5,6,7,8-hexahydropyrido[4',3':4,5]thieno [2,3-d]pyrimidin-2-yl)thio)-N-propylacetamide (**41**).* Following the same procedure for the synthesis of **14**, 7-acetyl-3-(4-ethoxyphenyl)-2-thioxo-2,3,5,6,7,8-hexahydropyrido[4',3':4,5]thieno[2,3-*d*]pyrimidin-4(1*H*)-one **25ab** (40 mg, 0.10 mmol, 1.0 equiv.), 2-bromo-*N*-propylacetamide (20 mg, 0.11 mmol, 1.1 equiv.), and Et_3N (30 μL, 0.20 mmol, 2.0 equiv.) in MeCN (1.0 mL) gave the title product (50 mg, 96%) as a white solid; R_f = 0.30 (CH_2Cl_2/MeOH 20/1); ^{1}H NMR (300 MHz, $CDCl_3$, mixture of the rotamers) δ 7.22 (d, J = 9.0 Hz, 2H), 7.06 (d, J = 9.0 Hz, 2H), 6.75 (br t, J = 6.0 Hz, 1H), 4.85 (s, 1.4H), 4.71 (s, 0.6H), 4.13 (q, J = 6.0 Hz, 2H), 3.93 (t, J = 6.0 Hz, 0.6H), 3.78 (t, J = 6.0 Hz, 1.4H), 3.74 (s, 2H), 3.24-3.31 (q, J = 6.0 Hz, 2H), 3.15-3.22 (m, 2H), 2.24 (s, 3H), 1.52-1.63 (m, 2H), 1.49 (t, J = 6.0 Hz, 3H), 0.94 (t, J = 6.0 Hz, 3H); ^{13}C NMR (75 MHz, $CDCl_3$, mixture of the rotamers) δ 169.56, 169.35, 167.74, 162.42, 160.33, 158.96, 158.83, 158.39, 131.65, 129.97 (2C), 129.72, 128.41, 127.04, 126.54, 118.85, 118.70, 115.65 (2C), 63.89, 45.32, 43.61, 41.56, 41.19, 38.79, 36.10, 29.35, 26.12, 25.25, 22.65, 22.09, 21.47, 14.74, 11.43, 11.29; HRMS (ESI$^+$): *m/z*: calcd for $C_{24}H_{29}N_4O_4S_2$ 501.1630 [M + H]$^+$; found: 501.1618.

*2-((7-Acetyl-3-(4-ethoxyphenyl)-4-oxo-3,4,5,6,7,8-hexahydropyrido[4',3':4,5]thieno [2,3-d]pyrimidin-2-yl)thio)-N-isopropylacetamide (**42**).* Following the same procedure for the synthesis

of **14**, 7-acetyl-3-(4-ethoxyphenyl)-2-thioxo-2,3,5,6,7,8-hexahydropyrido[4',3':4,5]thieno[2,3-*d*]pyrimidin-4(1*H*)-one **25ab** (40 mg, 0.10 mmol, 1.0 equiv.), 2-bromo-*N*-isopropylacetamide (20 mg, 0.11 mmol, 1.1 equiv.), and Et_3N (30 μL, 0.20 mmol, 2.0 equiv.) in MeCN (1.0 mL) gave the title product (42 mg, 81%) as a white solid; R_f = 0.30 (CH_2Cl_2/MeOH 20/1); ^{1}H NMR (300 MHz, $CDCl_3$, mixture of the rotamers) δ 7.18 (d, J = 8.9 Hz, 2H), 7.02 (d, J = 8.9 Hz, 2H), 6.54 (d, J = 7.9 Hz, 1H), 4.88–4.58 (m, 3H), 4.14–3.99 (m, 3H), 3.93–3.59 (m, 4H), 3.18–3.00 (m, 2H), 2.20 (s, 3H), 1.45 (t, J = 7.0 Hz, 3H), 1.14 (d, J = 6.6 Hz, 6H).; ^{13}C NMR (75 MHz, $CDCl_3$, mixture of the rotamers) δ 169.47, 169.25, 166.77, 162.31, 160.32, 158.91, 158.37, 158.18, 129.97 (2C), 129.76, 128.40, 127.09, 126.48, 118.71, 115.65 (2C), 63.89, 45.31, 43.61, 41.81, 41.18, 38.78, 36.37, 26.15, 25.27, 22.64, 22.08, 21.46, 14.74; HRMS (ESI$^+$): *m/z*: calcd for $C_{24}H_{29}N_4O_4S_2$ 501.1625 $[M + H]^+$; found: 501.1627.

*2-((7-Acetyl-3-(4-ethoxyphenyl)-4-oxo-3,4,5,6,7,8-hexahydropyrido[4',3':4,5]thieno [2,3-d]pyrimidin-2-yl)thio)-N-butylacetamide (**43**)*. Following the same procedure for the synthesis of **14**, 7-acetyl-3-(4-ethoxyphenyl)-2-thioxo-2,3,5,6,7,8-hexahydropyrido[4',3':4,5]thieno[2,3-*d*]pyrimidin-4(1*H*)-one **25ab** (40 mg, 0.10 mmol, 1.0 equiv.), 2-bromo-*N*-butylacetamide (22 mg, 0.11 mmol, 1.1 equiv.), and Et_3N (30 μL, 0.20 mmol, 2.0 equiv.) in MeCN (1.0 mL) gave the title product (53 mg, 98%) as a white solid; R_f = 0.30 (CH_2Cl_2/MeOH 20/1); ^{1}H NMR (300 MHz, $CDCl_3$, mixture of the rotamers) δ 7.22 (d, J = 9.0 Hz, 2H), 7.07 (d, J = 9.0 Hz, 2H), 6.72 (br t, J = 6.0 Hz, 1H), 4.85 (s, 1.5H), 4.71 (t, 0.5H), 4.13 (q, J = 6.0 Hz, 2H), 3.93 (t, J = 6.0 Hz, 0.5H), 3.78 (t, J = 6.0 Hz, 1.5H), 3.74 (s, 2H), 3.30 (q, J = 6.0 Hz, 2H), 3.09-3.15 (m, 2H), 2.24 (s, 3H), 1.47-1.52 (m, 5H), 1.34-1.39 (m, 2H), 0.93 (t, J = 6.0 Hz, 3H); ^{13}C NMR (75 MHz, $CDCl_3$, mixture of the rotamers) δ 169.57, 167.72, 162.41, 160.35, 158.85, 158.39, 131.72, 129.96 (2C), 129.74, 128.46, 127.04, 118.74, 115.66 (2C), 63.90, 45.32, 43.61, 42.71, 41.21, 39.56, 38.81, 36.05, 31.36,

26.13, 21.46, 20.05, 14.73, 13.72; HRMS (ESI+): *m/z*: calcd for $C_{25}H_{31}N_4O_4S_2$ 515.1781 $[M + H]^+$; found: 515.1783.

*7-Acetyl-3-(4-ethoxyphenyl)-2-((2-oxo-2-(4-phenylpiperazin-1-yl)ethyl)thio)-5,6,7,8-tetrahydropyrido[4',3':4,5]thieno[2,3-d]pyrimidin-4(3H)-one (**44**).* Following the same procedure for the synthesis of **14**, 7-acetyl-3-(4-ethoxyphenyl)-2-thioxo-2,3,5,6,7,8-hexahydropyrido [4',3':4,5]thieno[2,3-*d*]pyrimidin-4(1*H*)-one **25ab** (30 mg, 0.08 mmol, 1.0 equiv.), 2-bromo-1-(4-phenylpiperazin-1-yl)ethan-1-one (22 mg, 0.09 mmol, 1.1 equiv.), and Et_3N (16 μL, 0.12 mmol, 1.5 equiv.) in MeCN (1.0 mL) gave the title product (41 mg, 88%) as a white solid; R_f = 0.25 (EtOAc/*n*-Hexane 8/1); ^{1}H NMR (300 MHz, $CDCl_3$, mixture of the rotamers) δ 7.37 (t, *J* = 8.1 Hz, 2H), 7.23 (d, *J* = 8.7 Hz, 2H), 7.04-7.07 (m, 5H), 4.81 (s, 1.5H), 4.67 (s, 0.5H), 4.12 (q, *J* = 6.9 Hz, 2H), 4.09 (s, 2H), 3.89-3.91 (m, 4.5H), 3.76 (t, *J* = 6.0 Hz, 1.5H), 3.34 (m, 2H), 3.25 (m, 2H), 3.08-3.16 (m, 2H), 2.24 (s, 2.25H), 2.27 (s, 0.75H), 1.49 (t, *J* = 6.9 Hz, 3H); ^{13}C NMR (101 MHz, $CDCl_3$, mixture of the rotamers) δ 169.49, 169.25, 165.72, 160.26, 158.57, 150.78, 130.02 (2C), 129.59, 129.32 (2C), 128.04, 127.23, 127.18, 126.07, 120.74, 118.57, 116.74 (2C), 116.70, 115.67 (2C), 63.85, 61.87, 49.79, 49.35, 46.24, 45.29, 43.63, 42.29, 41.21, 38.79, 35.82, 35.70, 31.66, 26.12, 25.26, 22.06, 21.47, 14.76, 13.92; HRMS (ESI+): *m/z*: calcd for $C_{31}H_{34}N_5O_4S_2$ 604.2047 $[M + H]^+$; found: 604.2048.

*7-Acetyl-2-((2-(4-benzylpiperazin-1-yl)-2-oxoethyl)thio)-3-(4-ethoxyphenyl)-5,6,7,8-tetrahydropyrido[4',3':4,5]thieno[2,3-d]pyrimidin-4(3H)-one (**45**).* Following the same procedure for the synthesis of **14**, 7-acetyl-3-(4-ethoxyphenyl)-2-thioxo-2,3,5,6,7,8-hexahydropyrido [4',3':4,5]thieno[2,3-*d*]pyrimidin-4(1*H*)-one **25ab** (30 mg, 0.08 mmol, 1.0 equiv.), 1-(4-benzylpiperazin-1-yl)-2-bromoethan-1-one (23 mg, 0.09 mmol, 1.1 equiv.), and Et_3N (16 μL, 0.12 mmol, 1.5 equiv.) in MeCN (1.0 mL) gave the title product (32 mg, 67%) as a white solid; R_f =

0.30 (CH_2Cl_2/MeOH 20/1); ^{1}H NMR (300 MHz, $CDCl_3$, mixture of the rotamers) δ 7.30-7.50 (m, 5H), 7.23 (d, J = 9.0 Hz, 2H), 7.05 (d, J = 9.0 Hz, 2H), 4.83 (s, 1.5H), 4.69 (s, 0.5H), 4.12 (q, J = 6.0 Hz, 2H), 4.05 (s, 2H), 3.80 (t, J = 6.0 Hz, 0.5H), 3.75 (t, J = 6.0 Hz, 1.5H), 3.62-3.69 (m, 4H), 3.61 (s, 2H), 2.90-3.15 (m, 2H), 2.57 (m, 2H), 2.49 (m, 2H), 2.24 (s, 2.25H), 2.21 (s, 0.75H), 1.49 (t, J = 6.0 Hz, 3H); ^{13}C NMR (101 MHz, $CDCl_3$, mixture of the rotamers) δ 169.50, 165.47, 162.90, 160.22, 158.65, 158.58, 137.17, 131.60, 130.00 (2C), 129.54, 129.20 (2C), 128.41 (2C), 127.44, 127.25, 126.01, 118.52, 115.64 (2C), 63.83, 62.75, 53.02, 52.51, 46.19, 45.79, 45.31, 43.64, 42.30, 41.22, 38.78, 35.94, 31.58, 26.11, 22.25, 22.65, 22.06, 21.47, 14.75, 14.13, 8.62; HRMS (ESI$^+$): *m/z*: calcd for $C_{32}H_{36}N_5O_4S_2$ 618.2203 $[M + H]^+$; found: 618.2205.

*7-Acetyl-3-(4-ethoxyphenyl)-2-((2-oxo-2-(4-phenylpiperidin-1-yl)ethyl)thio)-5,6,7,8-tetrahydropyrido[4',3':4,5]thieno[2,3-d]pyrimidin-4(3H)-one (**46**).*Following the same procedure for the synthesis of **14**, 7-acetyl-3-(4-ethoxyphenyl)-2-thioxo-2,3,5,6,7,8-hexahydropyrido[4',3':4,5]thieno[2,3-*d*]pyrimidin-4(1*H*)-one **25ab** (30 mg, 0.08 mmol, 1.0 equiv.), 2-bromo-1-(4-phenylpiperidin-1-yl)ethan-1-one (22 mg, 0.09 mmol, 1.1 equiv.), and Et_3N (16 μL, 0.12 mmol, 1.5 equiv.) in MeCN (1.0 mL) gave the title product (39 mg, 84%) as a white solid; R_f = 0.15 (EtOAc/*n*-Hexane 4/1); ^{1}H NMR (300 MHz, $CDCl_3$, mixture of the rotamers) δ 7.37 (t, J = 6.9 Hz, 2H), 7.22-7.28 (m, 5H), 7.05 (d, J = 9.0 Hz, 2H), 4.68-4.82 (m, 2.5H), 4.22 (s, 0.5H), 4.11-4.17 (m, 5H), 4.09 (t, J = 6.0 Hz, 0.5H), 3.76 (t, J = 6.0 Hz, 1.5H), 3.29 (m, 1H), 3.14-3.16 (m, 2H), 2.73-2.82 (m, 2H), 2.24 (s, 3H), 1.71-1.76 (m, 2H), 1.47-1.65 (m, 2H), 1.31 (t, J = 6.0 Hz, 3H); ^{13}C NMR (101 MHz, $CDCl_3$, mixture of the rotamers) δ 169.54, 165.36, 162.96, 160.23, 158.75, 158.64, 144.87, 130.03, 129.56, 128.65 (2C), 127.94, 126.75, 126.72, 126.67, 126.00, 118.53, 115.66 (2C), 63.84, 47.02, 45.88, 45.30, 43.65, 43.17, 42.58, 41.21, 36.43, 33.90,

32.74, 26.12, 25.26, 22.06, 21.49, 14.77; HRMS (ESI$^+$): *m/z*: calcd for $C_{32}H_{35}N_4O_4S_2$ 603.2094 $[M + H]^+$; found: 603.2097.

*7-Acetyl-2-((2-(4-benzylpiperidin-1-yl)-2-oxoethyl)thio)-3-(4-ethoxyphenyl)-5,6,7,8-tetrahydropyrido[4',3':4,5]thieno[2,3-d]pyrimidin-4(3H)-one (**47**).* Following the same procedure for the synthesis of **14**, 7-acetyl-3-(4-ethoxyphenyl)-2-thioxo-2,3,5,6,7,8-hexahydropyrido [4',3':4,5]thieno[2,3-*d*]pyrimidin-4(1*H*)-one **25ab** (30 mg, 0.08 mmol, 1.0 equiv.), 1-(4-benzylpiperidin-1-yl)-2-bromoethan-1-one (23 mg, 0.09 mmol, 1.1 equiv.), and Et_3N (16 μL, 0.12 mmol, 1.5 equiv.) in MeCN (1.0 mL) gave the title product (44 mg, 91%) as a white solid; R_f = 0.15 (EtOAc/*n*-Hexane 4/1); ^{1}H NMR (300 MHz, $CDCl_3$, mixture of the rotamers) δ 7.31-7.36 (m, 2H), 7.17-7.28 (m, 5H), 7.04 (d, J = 9.0 Hz, 2H), 5.34 (s, 1.5H), 4.83 (s, 0.5H), 4.63 (m, 1H), 4.10-4.15 (m, 3H), 4.08 (s, 2H), 3.98 (t, J = 6.0 Hz, 0.5H), 3.76 (t, J = 6.0 Hz, 1.5H), 3.10-3.14 (m, 2H), 2.60-2.64 (m, 3H), 2.24 (s, 3H), 1.46-1.51 (m, 3H), 1.28 (t, J = 6.0 Hz, 3H); ^{13}C NMR (101 MHz, $CDCl_3$, mixture of the rotamers) δ 169.51, 169.25, 165.17, 162.96, 160.20, 158.83, 158.61, 139.77, 131.58, 130.03 (2C), 130.00 (2C), 129.54, 129.09, 128.35, 127.88, 127.31, 127.24, 126.13, 125.95, 118.49, 115.63 (2C), 63.82, 46.61, 45.30, 42.85, 42.78, 41.22, 38.13, 36.38, 32.58, 31.68, 26.11, 25.26, 22.05, 21.46, 14.76; HRMS (ESI$^+$): *m/z*: calcd for $C_{33}H_{37}N_4O_4S_2$ 617.2251 $[M + H]^+$; found: 617.2254.

*N-(3,4-Dimethoxyphenyl)-2-((3-(4-ethoxyphenyl)-7-methyl-4-oxo-3,4,5,6,7,8-hexahydropyrido[4',3':4,5]thieno[2,3-d]pyrimidin-2-yl)thio)acetamide (**48**).* Following the same procedure for the synthesis of **14**, 3-(4-ethoxyphenyl)-7-methyl-2-thioxo-2,3,5,6,7,8-hexahydropyrido[4',3':4,5]thieno[2,3-*d*]pyrimidin-4(1*H*)-one **25b** (30 mg, 0.08 mmol, 1.0 equiv.), 2-bromo-*N*-(3,4-dimethoxyphenyl)acetamide (29 mg, 0.10 mmol, 1.3 equiv.), and Et_3N (17 μL, 0.12 mmol, 1.5 equiv.) in MeCN (0.7 mL) gave the title product (15 mg, 33%) as a yellowish solid;

R_f = 0.45 (CH_2Cl_2/MeOH 10/1); ^{1}H NMR (300 MHz, $CDCl_3$) δ 9.27 (br s, 1H), 7.40 (d, J = 2.4 Hz, 1H), 7.23 (dd, J = 6.9 Hz, 2.1 Hz, 2H), 7.05 (dd, J = 6.9 Hz, 2.1Hz, 2H), 6.92 (dd, J = 8.4 Hz, 2.4 Hz, 1H), 6.83 (d, J = 8.4 Hz, 1H), 4.14 (q, J = 6.9 Hz, 2H), 3.91 (s, 3H), 3.89 (s, 3H), 3.84 (s, 2H), 3.70 (s, 2H), 3.12 (t, J = 5.4 Hz, 2H), 2.81 (t, J = 5.4 Hz, 2H), 2.56 (s, 3H), 1.49 (t, J = 6.9 Hz, 3H); ^{13}C NMR (101 MHz, $CDCl_3$) δ 166.03, 161.32, 160.40, 159.18, 158.12, 149.11, 145.77, 131.65, 130.43, 130.00 (2C), 129.11, 127.02, 119.32, 115.64 (2C), 111.42, 111.113, 104.28, 63.89, 56.15, 55.95, 53.56, 51.81, 45.46, 29.70, 25.72, 14.71; HRMS (ESI$^+$): *m/z*: calcd for $C_{28}H_{31}N_4O_5S_2$ 567.1730 [M + H]$^+$; found: 567.1732.

*N-Benzyl-2-((3-(4-ethoxyphenyl)-7-methyl-4-oxo-3,4,5,6,7,8-hexahydropyrido[4',3':4,5] thieno[2,3-d]pyrimidin-2-yl)thio)acetamide (**49**).* Following the same procedure for the synthesis of **14**, 3-(4-ethoxyphenyl)-7-methyl-2-thioxo-2,3,5,6,7,8-hexahydropyrido[4',3':4,5]thieno[2,3-*d*]pyrimidin-4(1*H*)-one **25b** (20 mg, 0.05 mmol, 1.0 equiv.), *N*-benzyl-2-bromoacetamide (13 mg, 0.07 mmol, 1.3 equiv.), and Et_3N (12 μL, 0.08 mmol, 1.5 equiv.) in MeCN (0.5 mL) gave the title product (21 mg, 75%) as a yellowish solid; R_f = 0.10 (EtOAc/*n*-Hexane 2/1); ^{1}H NMR (300 MHz, $CDCl_3$) δ 7.25-7.34 (m, 5H), 7.18 (dd, J = 6.9 Hz, 2.1 Hz, 2H), 7.03-7.08 (m, 3H), 4.47 (d, J = 5.7 Hz, 2H), 4.13 (q, J = 6.9 Hz, 2H), 3.80 (s, 2H), 3.67 (s, 2H), 3.10 (t, J = 5.7 Hz, 2H), 2.80 (t, J = 5.7 Hz, 2H), 2.55 (s, 3H), 1.49 (t, J = 6.9 Hz, 3H); ^{13}C NMR (101 MHz, $CDCl_3$) δ 167.79, 161.89, 160.29, 158.36, 157.82, 137.85, 130.03 (2C), 129.87, 129.13, 128.74 (2C), 128.38, 127.54 (2C), 127.21, 119.07, 115.61 (2C), 63.88, 53.57, 51.88, 43.88, 35.87, 30.95, 25.66, 14.74; HRMS (ESI$^+$): *m/z*: calcd for $C_{27}H_{29}N_4O_3S_2$ 521.1676 [M + H]$^+$; found: 521.1668.

*N-Benzyl-2-((3-(4-ethoxyphenyl)-7-methyl-4-oxo-3,4,5,6,7,8-hexahydropyrido[4',3':4,5] thieno[2,3-d]pyrimidin-2-yl)thio)-N-methylacetamide (**50**).* Following the same procedure for the synthesis of **14**, 3-(4-ethoxyphenyl)-7-methyl-2-thioxo-2,3,5,6,7,8-hexahydropyrido[4',3':4,5]

thieno[2,3-*d*]pyrimidin-4(1*H*)-one **25b** (20 mg, 0.05 mmol, 1.0 equiv.), *N*-benzyl-2-bromo-*N*-methylacetamide (14 mg, 0.07 mmol, 1.3 equiv.), and Et_3N (12 μL, 0.08 mmol, 1.5 equiv.) in MeCN (0.5 mL) gave the title product (24 mg, 85%) as a yellowish solid; R_f = 0.15 (EtOAc/*n*-Hexane 2/1); ^{1}H NMR (300 MHz, $CDCl_3$, mixture of the rotamers) *δ* 7.21-7.45 (m, 7H), 7.02-7.06 (m, 2H), 4.74 (s, 0.9H), 4.65 (s, 1.1H), 4.12 (q, *J* = 6.9 Hz, 2H), 4.10 (s, 2H), 3.67 (s, 2H), 3.01-3.13 (m, 5H), 2.80 (t, *J* = 6.9 Hz, 2H), 2.54 (s, 3H), 1.49 (t, *J* = 6.9 Hz, 3H); ^{13}C NMR (101 MHz, $CDCl_3$, mixture of the rotamers) *δ* 167.57, 167.40, 160.15, 158.12, 136.72, 130.13 (2C), 129.76, 129.09, 128.71 (2C), 128.46, 128.03 (2C), 127.86, 127.51, 126.51, 118.82, 115.59 (2C), 63.82, 53.92, 53.58, 51.90, 45.43, 36.30, 35.92, 35.29, 34.59, 25.68, 14.77; HRMS (ESI$^+$): *m/z*: calcd for $C_{28}H_{31}N_4O_3S_2$ 535.1832 [M + H]$^+$; found: 535.1823.

*N-Benzyl-2-((3-(4-ethoxyphenyl)-7-methyl-4-oxo-3,4,5,6,7,8-hexahydropyrido[4',3':4,5] thieno[2,3-d]pyrimidin-2-yl)thio)-N-ethylacetamide (**51**).* Following the same procedure for the synthesis of **14**, 3-(4-ethoxyphenyl)-7-methyl-2-thioxo-2,3,5,6,7,8-hexahydropyrido[4',3':4,5] thieno[2,3-*d*]pyrimidin-4(1*H*)-one **25b** (20 mg, 0.05 mmol, 1.0 equiv.), *N*-benzyl-2-bromo-*N*-ethylacetamide (15 mg, 0.07 mmol, 1.3 equiv.), and Et_3N (12 μL, 0.08 mmol, 1.5 equiv.) in MeCN (0.5 mL) gave the title product (23 mg, 83%) as a yellowish solid; R_f = 0.20 (EtOAc/*n*-Hexane 2/1); ^{1}H NMR (300 MHz, $CDCl_3$, mixture of the rotamers) *δ* 7.21-7.44 (m, 7H), 7.02-7.06 (m, 2H), 4.74 (s, 0.9H), 4.65 (s, 1.1H), 4.10-4.14 (m, 3.1H), 4.00 (s, 0.9H), 3.66 (s, 2H), 3.49 (q, *J* = 6.6 Hz, 2H), 3.10-3.12 (m, 2H), 2.79 (t, *J* = 5.7 Hz, 2H), 2.54 (s, 3H), 1.49 (t, *J* = 6.6 Hz, 3H), 1.29 (t, *J* = 7.5 Hz, 1.6H), 1.60 (t, *J* = 7.5 Hz, 1.4H); ^{13}C NMR (101 MHz, $CDCl_3$, mixture of the rotamers) *δ* 167.18, 167.08, 162.45, 160.14, 158.69, 158.17, 137.27, 136.69, 130.13 (2C), 129.76, 129.03, 128.63 (2C), 128.52 (2C), 128.00, 127.76, 127.54, 127.39, 126.48, 118.80, 115.55 (2C),

63.82, 53.61, 51.92, 51.29, 48.74, 45.47, 42.22, 41.97, 36.24, 36.08, 30.95, 25.71, 14.77, 13.90, 12.58; HRMS (ESI$^+$): *m/z*: calcd for $C_{29}H_{33}N_4O_3S_2$ 549.1989 [M + H]$^+$; found: 549.1980.

*N-(4-Chlorobenzyl)-2-((3-(4-ethoxyphenyl)-7-methyl-4-oxo-3,4,5,6,7,8-hexahydropyrido[4',3':4,5]thieno[2,3-d]pyrimidin-2-yl)thio)acetamide (**52**)*. Following the same procedure for the synthesis of **14**, 3-(4-ethoxyphenyl)-7-methyl-2-thioxo-2,3,5,6,7,8-hexahydropyrido[4',3':4,5]thieno[2,3-*d*]pyrimidin-4(1*H*)-one **25b** (20 mg, 0.05 mmol, 1.0 equiv.), 2-bromo-*N*-(4-chlorobenzyl)acetamide (15 mg, 0.07 mmol, 1.3 equiv.), and Et_3N (12 μL, 0.08 mmol, 1.5 equiv.) in MeCN (0.5 mL) gave the title product (19 mg, 65%) as a yellowish solid; R_f = 0.25 (CH_2Cl_2/MeOH 30/1); ^{1}H NMR (300 MHz, $CDCl_3$) δ 7.25 (dd, J = 9.0 Hz, 2.7 Hz, 2H), 7.18-7.21 (m, 5H), 7.04 (dd, J = 9.0 Hz, 4.2 Hz, 2H), 4.43 (d, J = 5.7 Hz, 2H), 4.12 (q, J = 6.9 Hz, 2H), 3.78 (s, 2H), 3.71 (s, 2H), 3.11 (t, J = 5.7 Hz, 2H), 2.83 (t, J = 5.7 Hz, 2H), 2.57 (s, 3H), 1.48 (t, J = 6.9 Hz, 3H); ^{13}C NMR (101 MHz, $CDCl_3$) δ 167.87, 161.82, 160.31, 158.25, 157.97, 136.46, 133.30, 130.01 (2C), 129.85, 128.95 (2C), 128.90, 128.82 (2C), 127.14, 119.03, 115.62 (2C), 63.88, 53.34, 51.67, 45.14, 43.17, 35.86, 25.39, 14.72; HRMS (ESI$^+$): *m/z*: calcd for $C_{27}H_{28}ClN_4O_3S_2$ 555.1286 [M + H]$^+$; found: 555.1286.

*N-(4-Chlorophenyl)-2-((3-(4-ethoxyphenyl)-7-methyl-4-oxo-3,4,5,6,7,8-hexahydropyrido[4',3':4,5]thieno[2,3-d]pyrimidin-2-yl)thio)acetamide (**53**)*. Following the same procedure for the synthesis of **14**, 3-(4-ethoxyphenyl)-7-methyl-2-thioxo-2,3,5,6,7,8-hexahydropyrido[4',3':4,5]thieno[2,3-*d*]pyrimidin-4(1*H*)-one **25b** (20 mg, 0.05 mmol, 1.0 equiv.), 2-bromo-*N*-(4-chlorophenyl)acetamide (14 mg, 0.07 mmol, 1.3 equiv.), and Et_3N (12 μL, 0.08 mmol, 1.5 equiv.) in MeCN (0.5 mL) gave the title product (11 mg, 40%) as a yellowish solid; R_f = 0.50 (CH_2Cl_2/MeOH 20/1); ^{1}H NMR (300 MHz, $CDCl_3$) δ 9.45 (br s, 1H), 7.50 (dd, J = 8.7 Hz, 1.8 Hz, 2H), 7.30 (dd, J = 8.7 Hz, 1.8 Hz, 2H), 7.22 (dd, J = 6.9 Hz, 2.1 Hz, 2H), 7.05 (dd, J = 6.9

Hz, 2.1 Hz, 2H), 4.12 (q, *J* = 6.9 Hz, 2H), 3.84 (s, 2H), 3.73 (s, 2H), 3.12 (t, *J* = 6.9 Hz, 2H), 2.82 (t, *J* = 6.9 Hz, 2H), 2.57 (s, 3H), 1.49 (t, *J* = 6.9 Hz, 3H); ^{13}C NMR (75 MHz, $CDCl_3$) δ 167.35, 166.39, 160.46, 160.05, 158.95, 136.52, 131.25, 130.00 (2C), 129.87, 129.29, 129.08 (2C), 126.05, 120.67 (2C), 118.65, 115.68 (2C), 63.91, 53.54, 51.79, 45.42, 36.80, 25.70, 14.72; HRMS (ESI^+): *m/z*: calcd for $C_{26}H_{26}ClN_4O_3S_2$ 541.1129 $[M + H]^+$; found: 541.1120.

*2-((3-(4-Ethoxyphenyl)-7-methyl-4-oxo-3,4,5,6,7,8-hexahydropyrido[4',3':4,5]thieno [2,3-d]pyrimidin-2-yl)thio)-N-(4-methylbenzyl)acetamide (**54**).*Following the same procedure for the synthesis of **14**, 3-(4-ethoxyphenyl)-7-methyl-2-thioxo-2,3,5,6,7,8-hexahydropyrido[4',3':4,5] thieno[2,3-*d*]pyrimidin-4(1*H*)-one **25b** (20 mg, 0.05 mmol, 1.0 equiv.), 2-bromo-*N*-(4-methylbenzyl)acetamide (17 mg, 0.07 mmol, 1.3 equiv.), and Et_3N (12 μL, 0.08 mmol, 1.5 equiv.) in MeCN (0.5 mL) gave the title product (20 mg, 47%) as a yellowish solid; R_f = 0.20 (CH_2Cl_2/MeOH 10/1); ^{1}H NMR (300 MHz, $CDCl_3$) δ 7.10-7.19 (m, 7H), 7.05 (d, *J* = 9.0 Hz, 2H), 4.42 (d, *J* = 6.0 Hz, 2H), 4.12 (q, *J* = 6.0 Hz, 2H), 3.78 (s, 2H), 3.67 (s, 2H), 3.11 (t, *J* = 6.0 Hz, 2H), 2.79 (t, *J* = 6.0 Hz, 2H), 2.55 (s, 3H), 2.36 (s, 3H), 1.49 (t, *J* = 6.0 Hz, 3H); ^{13}C NMR (75 MHz, $CDCl_3$) δ 167.72, 161.90, 160.28, 158.37, 157.78, 137.22, 134.83, 130.04 (2C), 129.88 (2C), 129.37 (2C), 129.22, 127.54, 127.25, 119.06, 115.61 (2C), 63.88, 53.60, 51.90, 45.48, 43.66, 35.90, 25.71, 21.12, 14.74; HRMS (ESI^+): *m/z*: calcd for $C_{28}H_{31}N_4O_3S_2$ 535.1832 $[M + H]^+$; found: 535.1824.

*2-((3-(4-Ethoxyphenyl)-7-methyl-4-oxo-3,4,5,6,7,8-hexahydropyrido[4',3':4,5]thieno [2,3-d]pyrimidin-2-yl)thio)-N-(p-tolyl)acetamide (**55**).* Following the same procedure for the synthesis of **14**, 3-(4-ethoxyphenyl)-7-methyl-2-thioxo-2,3,5,6,7,8-hexahydropyrido[4',3':4,5]thieno[2,3-*d*]pyrimidin-4(1*H*)-one **25b** (20 mg, 0.05 mmol, 1.0 equiv.), 2-bromo-*N*-(p-tolyl)acetamide (19 mg, 0.07 mmol, 1.3 equiv.), and Et_3N (12 μL, 0.08 mmol, 1.5 equiv.) in MeCN (0.5 mL) gave the

title product (20 mg, 49%) as a yellowish solid; R_f = 0.25 (CH_2Cl_2/MeOH 10/1); ^{1}H NMR (300 MHz, $CDCl_3$) δ 9.18 (s, 1H), 7.39 (d, J = 8.2 Hz, 2H), 7.18 (d, J = 8.9 Hz, 2H), 7.10 (d, J = 8.0 Hz, 2H), 7.01 (d, J = 8.9 Hz, 2H), 4.08 (q, J = 6.9 Hz, 2H), 3.84-3.71 (m, 4H), 3.10 (s, 2H), 2.80 (s, 2H), 2.54 (s, 3H), 2.30 (s, 3H), 1.45 (t, J = 6.9 Hz, 3H).; ^{13}C NMR (75 MHz, $CDCl_3$) δ 166.12, 160.38, 160.27, 159.10, 158.16, 135.34, 133.93, 130.28, 130.01 (2C), 129.87, 129.53 (2C), 127.04, 119.47 (2C), 119.23, 115.64 (2C), 63.88, 53.51, 51.80, 45.36, 36.81, 23.27, 20.87, 14.71; HRMS (ESI$^+$): *m/z*: calcd for $C_{27}H_{29}N_4O_3S_2$ 521.1676 [M + H]$^+$; found: 521.1669.

*2-((3-(4-Ethoxyphenyl)-7-methyl-4-oxo-3,4,5,6,7,8-hexahydropyrido[4',3':4,5]thieno [2,3-d]pyrimidin-2-yl)thio)-N,N-dipropylacetamide (**56**).* Following the same procedure for the synthesis of **14**, 3-(4-ethoxyphenyl)-7-methyl-2-thioxo-2,3,5,6,7,8-hexahydropyrido [4',3':4,5]thieno[2,3-*d*]pyrimidin-4(1*H*)-one **25b** (20 mg, 0.05 mmol, 1.0 equiv.), 2-bromo-*N*,*N*-dipropylacetamide (18 mg, 0.07 mmol, 1.3 equiv.), and Et_3N (12 µL, 0.08 mmol, 1.5 equiv.) in MeCN (0.5 mL) gave the title product (14 mg, 41%) as a yellowish solid; R_f = 0.60 (CH_2Cl_2/MeOH 10/1); ^{1}H NMR (300 MHz, $CDCl_3$) δ 7.24 (dd, J = 6.9 Hz, 2.1 Hz, 2H), 7.02 (dd, J = 6.9 Hz, 2.1 Hz, 2H), 4.08 (q, J = 6.9 Hz, 2H), 4.02 (s, 2H), 3.65 (s, 2H), 3.30-3.41 (m, 4H), 3.10 (t, J = 5.7 Hz, 2H), 2.78 (t, J = 5.7 Hz, 2H), 2.53 (s, 3H), 1.74 (sextet, J = 7.5 Hz, 2H), 1.57 (sextet, J = 7.5 Hz, 2H), 1.47 (t, J = 6.9 Hz, 3H), 1.02 (t, J = 7.5 Hz, 3H), 0.90 (t, J = 7.5 Hz, 3H); ^{13}C NMR (101 MHz, $CDCl_3$) δ 166.73, 163.35, 160.10, 159.78, 158.21, 130.10 (2C), 129.75, 128.43, 127.53, 118.75, 115.54 (2C), 63.79, 53.60, 51.93, 50.19, 48.39, 45.47, 36.11, 25.69, 22.40, 20.84, 14.74, 11.36 (2C); HRMS (ESI$^+$): *m/z*: calcd for $C_{26}H_{35}N_4O_3S_2$ 515.2145 [M + H]$^+$; found: 515.2138.

*N,N-Dibutyl-2-((3-(4-ethoxyphenyl)-7-methyl-4-oxo-3,4,5,6,7,8-hexahydropyrido [4',3':4,5]thieno[2,3-d]pyrimidin-2-yl)thio)acetamide (**57**).* Following the same procedure for the synthesis of **14**, 3-(4-ethoxyphenyl)-7-methyl-2-thioxo-2,3,5,6,7,8-hexahydropyrido

[4',3':4,5]thieno[2,3-*d*]pyrimidin-4(1*H*)-one **25b** (25 mg, 0.07 mmol, 1.0 equiv.), 2-bromo-*N*,*N*-butylacetamide (20 mg, 0.08 mmol, 1.3 equiv.), and Et_3N (14 μL, 0.10 mmol, 1.5 equiv.) in MeCN (0.7 mL) gave the title product (12 mg, 33%) as a yellowish solid; R_f = 0.70 (CH_2Cl_2/MeOH 10/1); ^{1}H NMR (300 MHz, $CDCl_3$) δ 7.24 (d, *J* = 9.0 Hz, 2H), 7.03 (d, *J* = 9.0 Hz, 2H), 4.12 (q, *J* = 6.0 Hz, 2H), 4.03 (s, 2H), 3.69 (s, 2H), 3.33-3.44 (m, 4H), 3.12 (t, *J* = 6.0 Hz, 2H), 2.84 (t, *J* = 6.0 Hz, 2H), 2.55 (s, 3H), 2.06 (m, 2H), 1.66-1.72 (m, 2H), 1.46-1.57 (m, 7H), 1.30-1.35 (m, 2H), 1.04 (t, *J* = 6.0 Hz, 3H), 0.93 (t, *J* = 6.0 Hz, 3H); ^{13}C NMR (75 MHz, $CDCl_3$) δ 166.59, 162.58, 162.34, 160.12, 158.73, 149.10, 130.12 (2C), 129.93, 129.71, 118.99, 118.74, 115.57 (2C), 63.82, 53.69, 51.85, 48.32, 46.53, 45.28, 36.25, 31.31, 29.75, 25.54, 20.22, 20.16, 14.74, 13.84, 13.76; HRMS (ESI$^+$): *m/z*: calcd for $C_{28}H_{39}N_4O_3S_2$ 543.2458 [M + H]$^+$; found: 543.2449.

*2-((7-Acetyl-3-ethyl-4-oxo-3,4,5,6,7,8-hexahydropyrido[4',3':4,5]thieno[2,3-d]pyrimi-din-2-yl)thio)-N-(3,4-dimethoxyphenyl)acetamide (**58**).* Following the same procedure for the synthesis of **14**, 7-acetyl-3-ethyl-2-thioxo-2,3,5,6,7,8-hexahydropyrido[4',3':4,5]thieno[2,3-*d*]pyrimidin-4(1*H*)-one **25c** (30 mg, 0.10 mmol, 1.0 equiv.) and 2-bromo-*N*-(3,4-dimethoxyphenyl)acetamide (32 mg, 0.12 mmol, 1.2 equiv.), and Et_3N (20 μL, 0.15 mmol, 1.5 equiv.) in MeCN (0.9 mL) gave the title product (41 mg, 85%) as a yellowish solid; R_f = 0.20 (CH_2Cl_2/MeOH 10/1); ^{1}H NMR (300 MHz, $CDCl_3$, mixture of the rotamers) δ 9.15 (br s, 1H), 7.44 (s, 1H), 6.83 (m, 2H), 4.85 (s, 1.6H), 4.70 (s, 0.4H), 4.22 (q, *J* = 6.9 Hz, 2H), 4.00 (s, 2H), 3.90-3.94 (m, 3.4H), 3.88 (s, 3H), 3.80 (t, *J* = 6.0 Hz, 1.6H), 3.21 (t, *J* = 6.0 Hz, 1.6H), 3.15 (t, *J* = 6.0 Hz, 0.4H), 2.25 (s, 2H), 2.24 (s, 1H), 1.42 (t, *J* = 6.9 Hz, 3H); ^{13}C NMR (75 MHz, $CDCl_3$, mixture of the rotamers) δ 169.50, 165.76, 161.49, 157.47, 157.30, 149.12, 145.91, 131.52, 129.77, 128.35, 118.66, 111.42, 111.26, 104.42, 56.13, 55.97, 43.58, 41.19, 40.00, 36.28, 26.25, 21.44, 13.02; HRMS (ESI$^+$): *m/z*: calcd for $C_{23}H_{27}N_4O_5S_2$ 503.1417 [M + H]$^+$; found: 503.1419.

*2-((7-Acetyl-4-oxo-3-propyl-3,4,5,6,7,8-hexahydropyrido[4',3':4,5]thieno[2,3-d]pyrimi-din-2-yl)thio)-N-(3,4-dimethoxyphenyl)acetamide (**59**).* Following the same procedure for the synthesis of **14**, 7-acetyl-3-propyl-2-thioxo-2,3,5,6,7,8-hexahydropyrido[4',3':4,5]thieno[2,3-*d*]pyrimidin-4(1*H*)-one **25d** (15 mg, 0.05 mmol, 1.0 equiv.) and 2-bromo-*N*-(3,4-dimethoxyphenyl)acetamide (15 mg, 0.06 mmol, 1.2 equiv.), and Et_3N (10 μL, 0.07 mmol, 1.5 equiv.) in MeCN (0.5 mL) gave the title product (21 mg, 89%) as a yellowish solid; R_f = 0.20 (CH_2Cl_2/MeOH 10/1); ^{1}H NMR (400 MHz, $CDCl_3$, mixture of the rotamers) δ 9.13 (br s, 1H), 7.42 (s, 1H), 6.79-6.85 (m, 2H), 4.82 (s, 1.6H), 4.67 (s, 0.4H), 4.17 (t, J = 8.0 Hz, 2H), 3.99 (s, 2H), 3.88-3.92 (m, 3.4H), 3.86 (s, 3H), 3.77 (t, J = 6.0 Hz, 1.6H), 3.11-3.17 (m, 2H), 2.23 (s, 2H), 2.19 (s, 1H), 1.77-1.86 (m, 2H), 1.03 (t, J = 8.0 Hz, 3H); ^{13}C NMR (101 MHz, $CDCl_3$, mixture of the rotamers) δ 169.46, 165.73, 161.41, 157.61, 157.50, 149.14, 145.92, 131.49, 129.78, 128.39, 118.64, 111.44, 111.22, 104.42, 56.13, 55.96, 46.24, 43.56, 41.18, 36.28, 26.23, 21.40, 21.25, 11.24; HRMS (ESI^+): *m/z*: calcd for $C_{24}H_{29}N_4O_5S_2$ 517.1574 $[M + H]^+$; found: 517.1567.

*2-((7-Acetyl-3-isobutyl-4-oxo-3,4,5,6,7,8-hexahydropyrido[4',3':4,5]thieno[2,3-d]pyri-midin-2-yl)thio)-N-(3,4-dimethoxyphenyl)acetamide (**60**).* Following the same procedure for the synthesis of **14**, 7-acetyl-3-isobutyl-2-thioxo-2,3,5,6,7,8-hexahydropyrido[4',3':4,5]thieno[2,3-d]pyrimidin-4(1H)-one **25e** (40 mg, 0.12 mmol, 1.0 equiv.) and 2-bromo-*N*-(3,4-dimethoxyphenyl)acetamide (41 mg, 0.14 mmol, 1.2 equiv.), and Et_3N (25 μL, 0.18 mmol, 1.5 equiv.) in MeCN (1.2 mL) gave the title product (51 mg, 81%) as a yellowish solid; R_f = 0.20 (CH_2Cl_2/MeOH 10/1); ^{1}H NMR (400 MHz, $CDCl_3$, mixture of the rotamers) δ 9.09 (br s, 1H), 7.43 (s, 1H), 6.83 (m, 2H), 4.85 (s, 1.6H), 4.70 (s, 0.4H), 4.01 (d, J = 6.3 Hz, 2H), 4.00 (s, 2H), 3.91-3.92 (m, 3.4H), 3.88 (s, 3H), 3.77 (t, J = 6.0 Hz, 1.6H), 3.20 (m, 2H), 2.28-2.35 (m, 1H), 2.25 (s, 3H), 1.29 (dd, J = 6.9 Hz, 3.6 Hz, 6H); ^{13}C NMR (75 MHz, $CDCl_3$, mixture of the rotamers) δ 169.51, 165.81, 161.28, 158.03, 157.96,

149.12, 145.90, 131.45, 129.89, 128.47, 118.66, 111.39, 111.19, 104.35, 56.12, 55.97, 51.19, 43.58, 41.19, 38.72, 36.40, 27.73, 26.28, 21.43, 20.17; HRMS (ESI$^+$): *m/z*: calcd for $C_{25}H_{31}N_4O_5S_2$ 531.1730 [M + H]$^+$; found: 531.1724.

*2-((7-Acetyl-3-butyl-4-oxo-3,4,5,6,7,8-hexahydropyrido[4',3':4,5]thieno[2,3-d]pyrimi-din-2-yl)thio)-N-(3,4-dimethoxyphenyl)acetamide (**61**).* Following the same procedure for the synthesis of **14**, 7-acetyl-3-butyl-2-thioxo-2,3,5,6,7,8-hexahydropyrido[4',3':4,5]thieno[2,3-d]pyrimidin-4(1H)-one **25f** (40 mg, 0.12 mmol, 1.0 equiv.) and 2-bromo-*N*-(3,4-dimethoxyphenyl)acetamide (41 mg, 0.14 mmol, 1.2 equiv.), and Et_3N (25 μL, 0.18 mmol, 1.5 equiv.) in MeCN (1.2 mL) gave the title product (32 mg, 50%) as a yellowish solid; R_f = 0.25 (CH_2Cl_2/MeOH 10/1); ^{1}H NMR (400 MHz, $CDCl_3$, mixture of the rotamers) δ 9.19 (br s, 1H), 7.44 (s, 1H), 6.79-6.83 (m, 2H), 4.83 (s, 1.6H), 4.78 (s, 0.4H), 3.97-4.16 (m, 2H), 3.94 (s, 2H), 3.83-3.87 (m, 3.4H), 3.81 (s, 3H), 3.78 (t, *J* = 6.0 Hz, 1.6H), 3.17 (m, 2H), 2.25 (s, 2H), 2.23 (s, 1H), 1.72-1.79 (m, 2H), 1.41-1.51 (m, 2H), 1.01 (t, *J* = 7.2 Hz, 3H); ^{13}C NMR (75 MHz, $CDCl_3$, mixture of the rotamers) δ 169.54, 165.81, 161.40, 157.62, 157.55, 149.11, 145.89, 131.46, 129.80, 128.41, 118.65, 111.37, 111.17, 104.33, 56.12, 55.97, 44.69, 43.58, 41.20, 36.23, 29.80, 26.24, 21.44, 20.21, 13.60; HRMS (ESI$^+$): *m/z*: calcd for $C_{25}H_{31}N_4O_5S_2$ 531.1730 [M + H]$^+$; found: 531.1721.

*2-((7-Acetyl-3-isopropyl-4-oxo-3,4,5,6,7,8-hexahydropyrido[4',3':4,5]thieno[2,3-d]pyrimidin-2-yl)thio)-N-(3,4-dimethoxyphenyl)acetamide (**62**).* Following the same procedure for the synthesis of **14**, 7-acetyl-3-isopropyl-2-thioxo-2,3,5,6,7,8-hexahydropyrido[4',3':4,5]thieno[2,3-d]pyrimidin-4(1H)-one **25g** (40 mg, 0.12 mmol, 1.0 equiv.) and 2-bromo-*N*-(3,4-dimethoxyphenyl)acetamide (41 mg, 0.14 mmol, 1.2 equiv.), and Et_3N (25 μL, 0.18 mmol, 1.5 equiv.) in MeCN (1.2 mL) gave the title product (56 mg, 90%) as a yellowish solid; R_f = 0.25 (CH_2Cl_2/MeOH 10/1); ^{1}H NMR (300 MHz, $CDCl_3$, mixture of the rotamers) δ 9.11 (br s, 1H),

7.45 (s, 1H), 6.84 (m, 2H), 4.84 (s, 1.7H), 4.62-6.70 (m, 1.3H), 3.98 (s, 2H), 3.88-3.93 (m, 3.3H), 3.87 (s, 3H), 3.79 (t, J = 6.0 Hz, 1.7H), 3.19 (m, 2H), 2.25 (s, 3H), 1.70 (dd, J = 6.9 Hz, 2.9 Hz, 6H); ^{13}C NMR (75 MHz, $CDCl_3$, mixture of the rotamers) δ 169.50, 165.88, 160.81, 158.43, 157.48, 149.11, 145.89, 131.56, 129.78, 119.86, 111.46, 111.33, 104.52, 56.14, 55.96, 45.31, 43.59, 41.22, 38.73, 36.92, 26.33, 22.03, 21.41, 19.45; HRMS (ESI$^+$): *m/z*: calcd for $C_{24}H_{29}N_4O_5S_2$ 517.1574 $[M + H]^+$; found: 517.1567.
